# Supplementary material for: Rapid and scalable photocatalytic C(sp2)–C(sp3) Suzuki−Miyaura cross-coupling of aryl bromides with alkyl boranes
Source: Nat Commun. 2024 May 13;15:4028. doi: 10.1038/s41467-024-48212-5 (PMC11091139; doi:10.1038/s41467-024-48212-5)
Supplement: Supplementary file 1 — Supplementary Information [file 41467_2024_48212_MOESM1_ESM.pdf]

Supplementary Information

Rapid and Scalable Photocatalytic C(sp^2)–C(sp^3) Suzuki−Miyaura Cross-Coupling of Aryl Bromides with Alkyl Boranes

Ting Wan,[1,2‡] Luca Capaldo,[1,3‡] Jonas Djossou,[1] Angela Staffa,[1,4] Felix J. de Zwart,[5] Bas de Bruin,[5] Timothy Noël[1*]

[1] *Flow Chemistry Group, van 't Hoff Institute for Molecular Sciences (HIMS), University of Amsterdam, 1098 XH Amsterdam, The Netherlands;*

[2] *The Research Center of Chiral Drugs, Innovation Research Institute of Traditional Chinese Medicine, Shanghai University of Traditional Chinese Medicine, Shanghai 201203, China.*

[3] *SynCat Lab, Department of Chemistry, Life Sciences and Environmental Sustainability, University of Parma, 43124 Parma, Italy.*

[4] *Merck Healthcare KGaA, Frankfurter Str. 250, 64293 Darmstadt, Germany*

[5] *Homogeneous, Supramolecular and Bioinspired Catalysis Group (HomKat), van't Hoff Institute for Molecular Sciences (HIMS), Universiteit van Amsterdam (UvA), 1098 XH Amsterdam, The Netherlands;*

‡ These authors contributed equally to the work

.

1. Supplementary Methods S4

1.1 General information S4

1.2 Reactor design S5

1.2.1 UFO reactor S5

1.2.2 Flow reactor (UFlow) S7

1.2.3 3D-printed setup to check light intensity (vide infra) S7

1.3 Charts of starting materials S10

1.3.1 Olefins S10

1.3.2 Aryl bromides S10

1.3.3 Starting materials S11

1.4 Optimization of reaction conditions S13

*1.4.1 Optimization of conditions for reaction with triethyl borane (**1a**) S13*

1.4.2 Optimization of reaction conditions with 9-borabicyclo[3.3.1]nonane (9-BBN) ..S16

1.4.3 Adjustment of reaction conditions with 9-borabicyclo[3.3.1]nonane (9-BBN) for continuous-flow S18

*1.4.4 Adjustment of reaction conditions with triethyl borane (**1a**) for continuous-flow.S20*

1.5 General procedures (GPs) for preparative experiments S21

1.5.1 GP2: batch conditions with commercial available alkyl borane S21

1.5.2 GP3 (Method A): batch conditions – via hydroboration of olefins with THF·BH$_3$S21

1.5.3 GP4 (Method B): batch conditions - via hydroboration of olefins with 9-BBN S21

1.5.4 GP5: continuous flow conditions with 9-BBN, blue light (456 nm) S22

*1.5.5 Scale-up for the synthesis of compound **44** in continuous-flow S23*

2. Supplementary Discussion S26

2.1 Mechanistic investigation S26

2.1.1 Preliminary evaluation of reaction profile S26

2.1.2 Kinetics S27

2.1.3 Hammett plot S32

2.1.4 Quantum Yield measurement S33

2.1.5 Chemical quenching experiments S34

2.1.6 Electron Paramagnetic Resonance experiments S38

2.1.7 Experiments with the Nickel complex *S39*
2.1.8 Experiments with Ni(COD)$_2$ *S41*
2.1.9 Ligand effect *S43*
2.1.10 Complexation studies *S44*
2.1.11 Decomposition of the PC *S46*
2.1.12 Redox potential of PCs *S50*
2.1.13 Emission quenching experiments with 2,6-lutidine. *S51*
2.1.14 Supplementary discussion on the mechanistic scenario. *S51*
2.3 Characterization data S53
2.4 NMR spectra S69
3. Supplementary References S122

1. Supplementary Methods

1.1 General information

Reagents and consumables. All reagents and solvents were bought from Sigma Aldrich, TCI, Flurochem, VWR International and Biosolv and used as received. Disposable syringes were purchased from Laboratory Glass Specialist. Syringe pumps were purchased from Chemix Inc. model Fusion 200 Touch. All capillary tubing, microfluidic fittings and Back Pressure Regulator (BPR) were purchased from IDEX Health & Science. Product isolation was performed manually, using silica (P60, SILICYCLE), or automatically, by a Biotage® Isolation Four, with Biotage® SNAP KP-Sil 20 or 50 g flash chromatography cartridges. TLC analysis was performed using Silica on aluminum foils TLC plates (F254, SILICYCLE) with visualization under ultraviolet light (254 nm and 365 nm) or appropriate TLC staining (potassium permanganate or cerium ammonium molybdate).

NMR spectroscopy. ^{1}H (400 MHz or 300 MHz), ^{13}C (101 MHz or 75 MHz), ^{31}P (121 MHz) and ^{11}B (128 MHz or 96 MHz) spectra were recorded unless stated otherwise on ambient temperature using a Bruker AV400 or a Bruker AV300. ^{1}H NMR spectra are reported in parts per million (ppm) downfield relative to $CDCl_3$ (7.26 ppm) or CD_2Cl_2 (5.32 ppm) and all ^{13}C NMR spectra are reported in ppm relative to $CDCl_3$ (77.2 ppm) or CD_2Cl_2 (53.8 ppm) unless stated otherwise. The multiplicities of signals are designated by the following abbreviations: s (singlet), d (doublet), t (triplet), q (quartet), m (multiplet), dd (doublet of doublets), dt (doublet of triplets), td (triplet of doublets), tt (triplets of triplets), ddd (doublet of doublet of doublets), qd (quartet of doublet). Coupling constants (*J*) are reported in hertz (Hz). NMR data was processed using the MestReNova 14 software package. Known products were characterized by comparing to the corresponding ^{1}H NMR, ^{13}C NMR, and ^{11}B NMR with those available in the literature.

Melting point. Melting points were measured using a Büchi Melting Point M-565 apparatus.

Mass spectrometry. High resolution mass spectra (HRMS) were collected on an AccuTOF LC, JMS-T100LP Mass spectrometer (JEOL, Japen).

UV-Vis spectroscopy. UV-Vis spectra were recorded with a single-beam Duetta ExSpec equipped with a Xe arc lamp (250-1000 nm) and a CCD camera as a detector. Measurements were performed in a quartz cuvette (optical path: 1 cm). For quantum yield measurements, a double beam spectrophotometer Shimadzu UV2700 equipped with a deuterium lamp (190-350 nm), a halogen lamp (330-900 nm) and a photomultiplier (Hamamatsu R928).

1.2 Reactor design

1.2.1 UFO reactor

For all batch experiments a homemade, 3D-printed reactor was adopted. The reactor was designed to fit reaction vials and to be equipped with a Kessil lamp PR160L series (λ_{em} = 390 or 456 nm). The reactor was designed in Adobe Inventor 2021 with 4 different parts. The lid (100 mm × 12 mm) is designed to host up to 8 reactions vials and holds the Kessil lamp in the center (**Supplementary Figure 1**A); a fan (SUNON DCLüfter 24 V; 50x50x15 Vapo RoHS) is mounted on the bottom of the reactor for cooling. The box is designed with holes to allow the air flow to escape the reactor and keep the temperature stable around 30−33 °C (**Supplementary Figure 1**B), as measured by an external thermometer. A reflector is situated underneath the lamp and reflects the photons inside the box to have homogeneous light distribution (**Supplementary Figure 1**C). Finally, the stirring plate adapter (**Supplementary Figure 1**D) was added to fix the system on a stirring plate and provide homogeneous stirring (**Supplementary Figure 1**E). It also spaces the reflector from the plate to ensure a continuous air flow from the top to the bottom of the system. All the inside surfaces were covered with reflective tape. An overview of the assembled reactor is shown in **Supplementary Figure 2**.

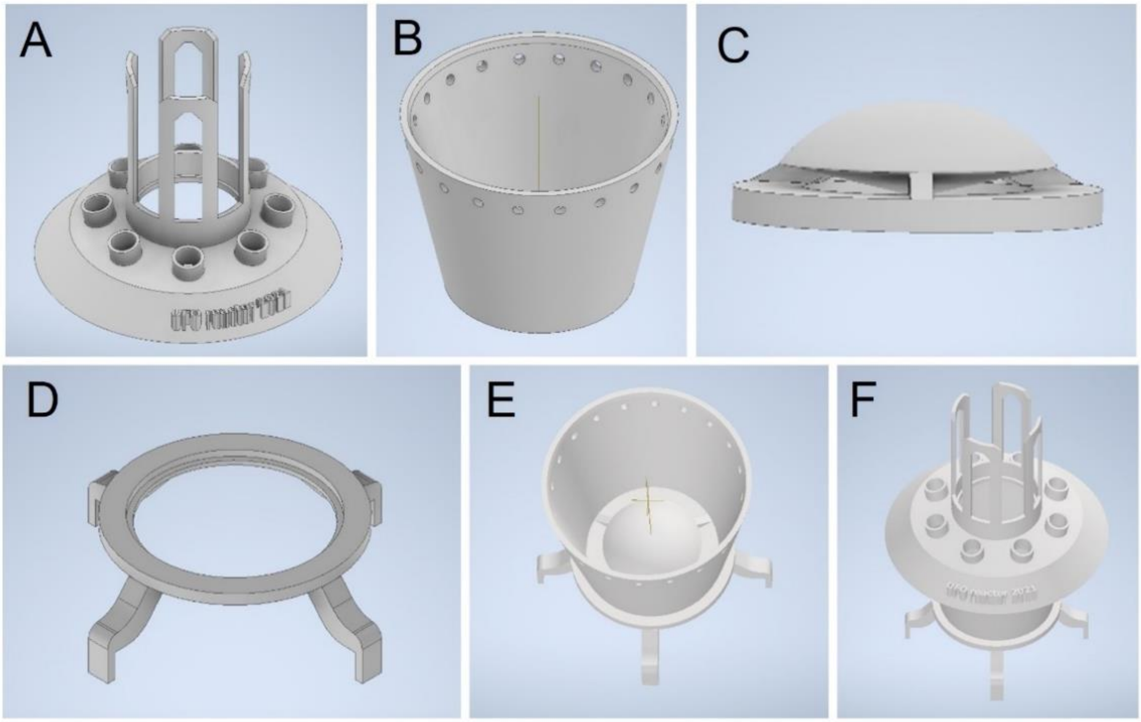

Supplementary Figure 1: Overview of the 3D-printed reactor. A) lid designed to host up to 8 reactions vials and hold the Kessil lamp in the center; B) body of the reactor; C) light reflector: it is coated with reflective tape; D) adapter for stirring plate; E) inside of the reactor; G) overall reactor.

Copyright: "Photoinduced Halogen-Atom Transfer by N-Heterocyclic Carbene-Ligated Boryl Radicals for C(sp3)–C(sp3) Bond Formation" by our group is licensed under CC BY 4.0.

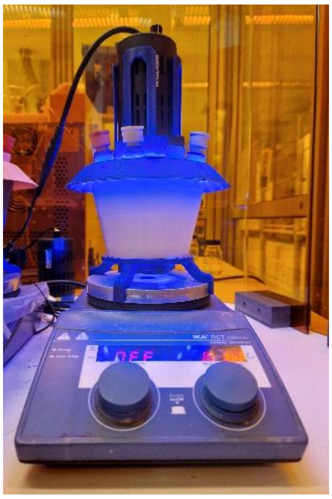

Supplementary Figure 2: Assembled reactor. Picture of the assembled reactor equipped with a Kessil lamp (λ_{em} = 456 nm). Copyright: "Photoinduced Halogen-Atom Transfer by N-Heterocyclic Carbene-Ligated Boryl Radicals for C(sp3)–C(sp3) Bond Formation" by our group is licensed under CC BY 4.0.

For kinetic experiments, the UFO kinetics reactor was used: the only variation compared to the classic UFO reactor is the lid. In particular, the new lid hosts 4 reaction vials (instead of 8) and holds the Kessil lamp in the center. This design was made to ensure that, with a proper alignment of Kessil lamps of the PR160L series (equipped with the linear reflector), the 4 vials experienced the same photonic flux (**Supplementary Figure 3**). This was found to be crucial to avoid reproducibility issues.

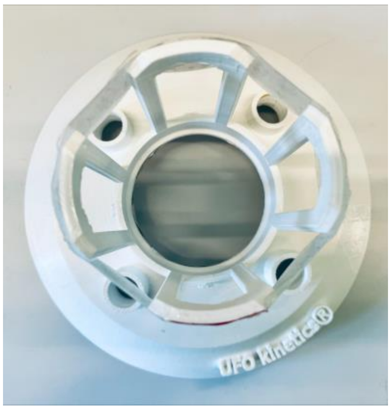

Supplementary Figure 3: Picture of the lid for the UFO kinetics reactor.

1.2.2 Flow reactor (UFlow)

For all flow experiments a homemade, 3D-printed reactor was adopted (**Supplementary Figure 4**, left). The reactor consists of a lid to host the Kessil lamp and a support around which a PFA tubing (0.8 mm inner diameter) is coiled (**Supplementary Figure 4**, middle); a fan (SUNON DCLüfter 24 V; 50x50x15 Vapo RoHS) is mounted on the bottom of the reactor for cooling. The coil is inserted in a cylindrical plastic body **Supplementary Figure 4**, right) with holes to allow the air flow to escape the reactor and keep the temperature stable around 30−33 °C. Additional details on the reactor will be published elsewhere.

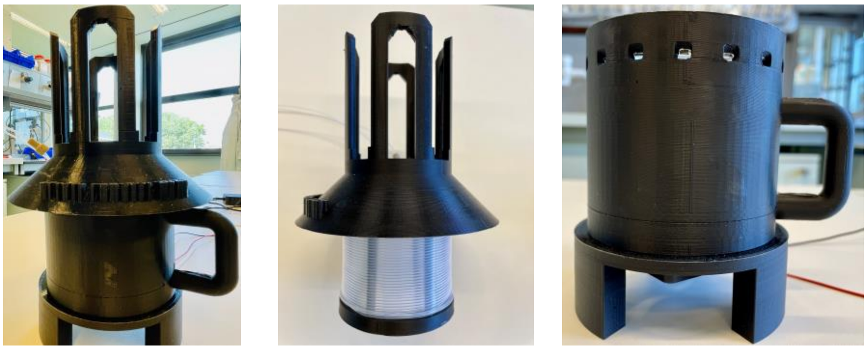

Supplementary Figure 4: **Overview of the 3D-printed flow reactor. Left**: overall reactor; **middle**: inside of the reactor (flow loop) and holder for the Kessil lamp in the center; **right**: main body, internally coated with reflective tape. A fan is mounted on the bottom to keep the temperature stable around 30−33 °C.

1.2.3 3D-printed setup to check light intensity (vide infra)

The reactor was designed in Adobe Inventor 2021. The setup consists of two sections tagged below as Part A and Part B. Part A is the socket for the Kessil lamp (PR160L series) used in this work. The cavity has a conical shape so that the lamp can slide in and firmly secure itself, being in a specific position ensuring reproducibility. This guarantees a consistent distance between the lamp and the light meter. Part B is the socket for the light meter, positioned in such a way that the sensor fits the window. Part A and B clip together thanks to embedded magnets (**Supplementary Figure 5**). The entire setup was printed in PLA filament with an Ultimaker Model S3.

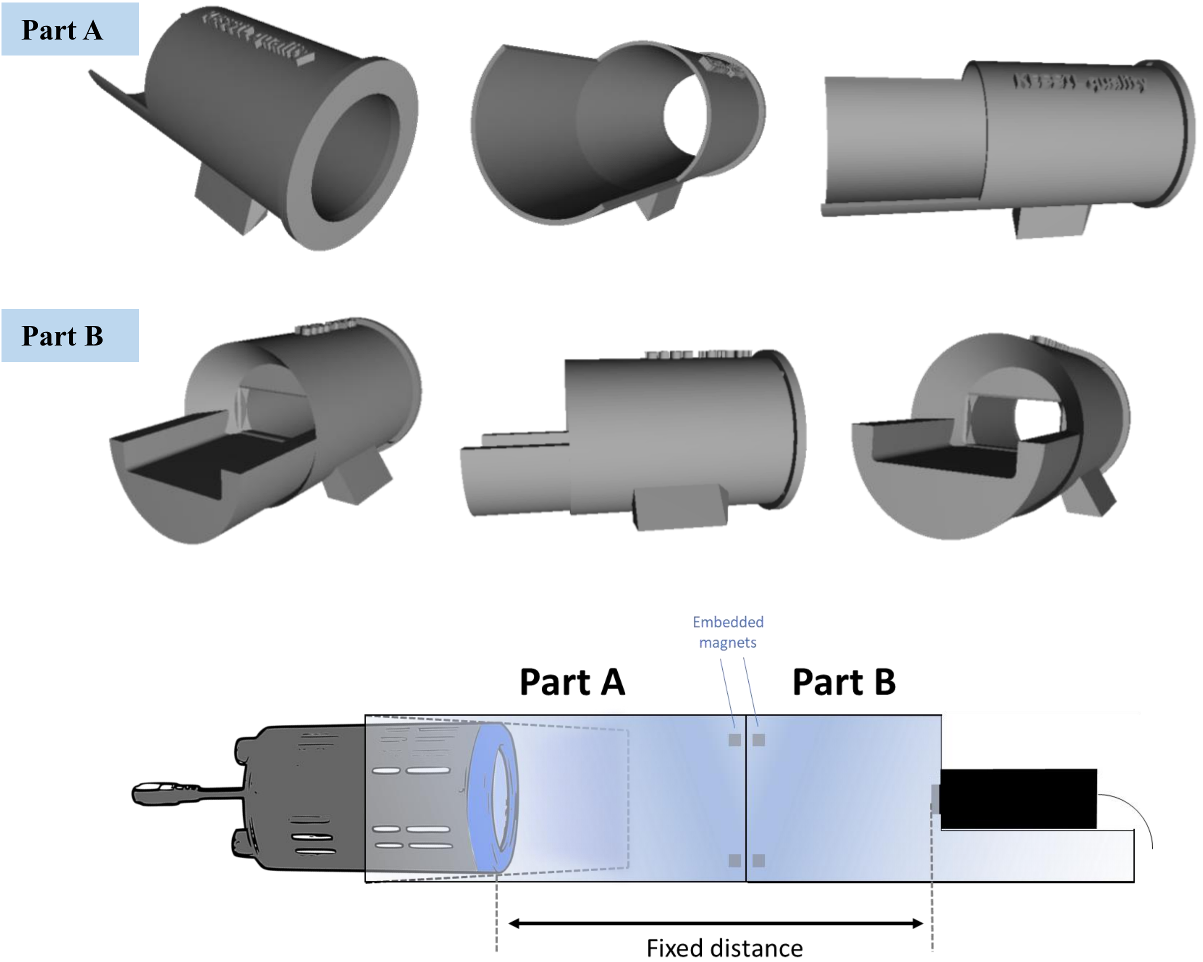

Supplementary Figure 5: **3D-printed setup for light intensity control**.

Part B of the reactor shown above was replaced with Part C. Part C is the socket for the cuvette. Part A and C clip together thanks to embedded magnets, similarly to what shown above for Part A and B (**Supplementary Figure 6**). The entire setup was printed in PLA filament with an Ultimaker Model S3.

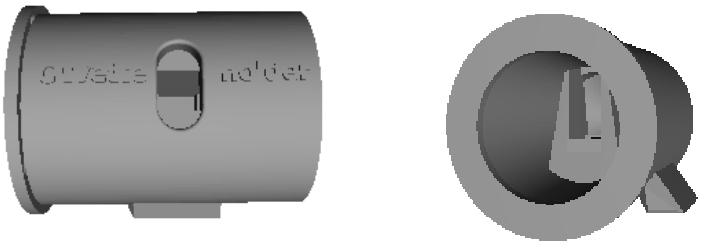

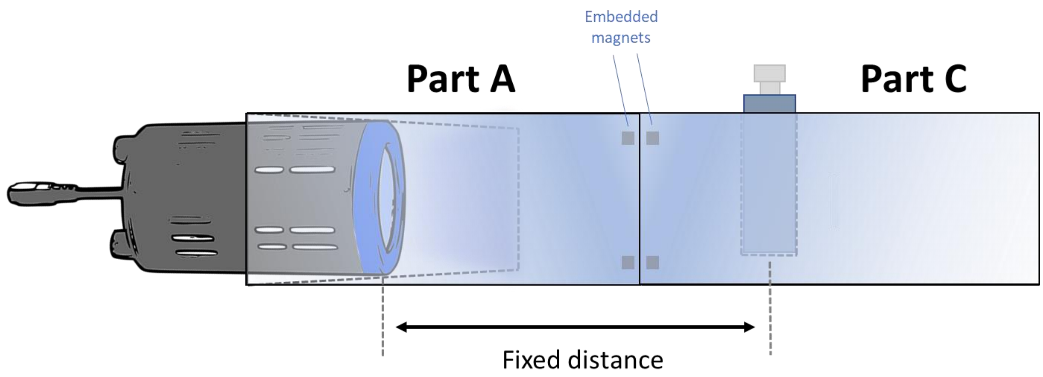

Supplementary Figure 6: **3D-printed setup for irradiation in cuvette** (vide infra).

1.3 Charts of starting materials

1.3.1 Olefins

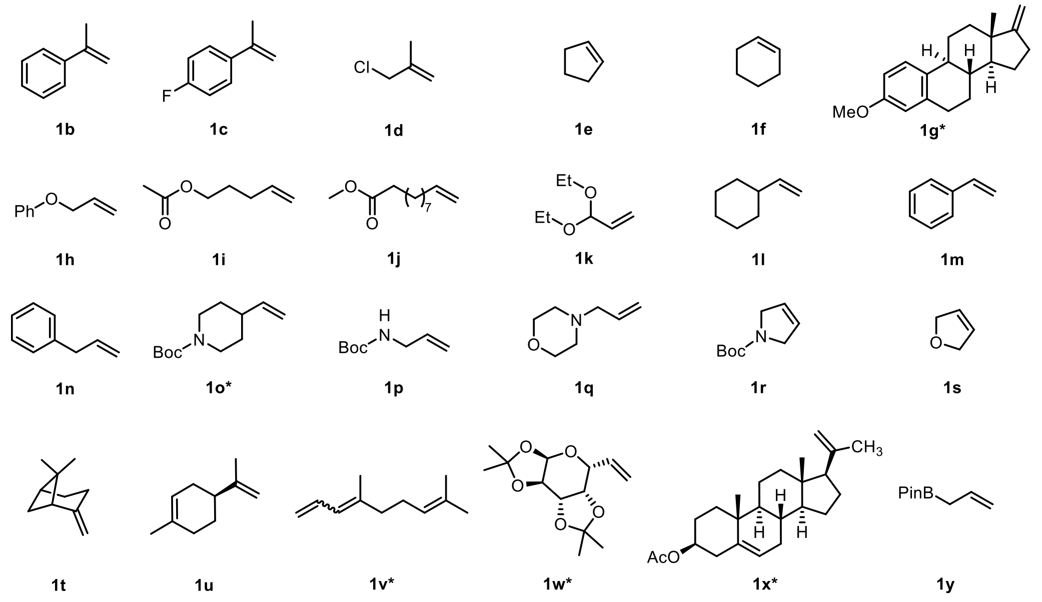

Supplementary Figure 7: **Olefins used as starting materials for hydroboration.**

1.3.2 Aryl bromides

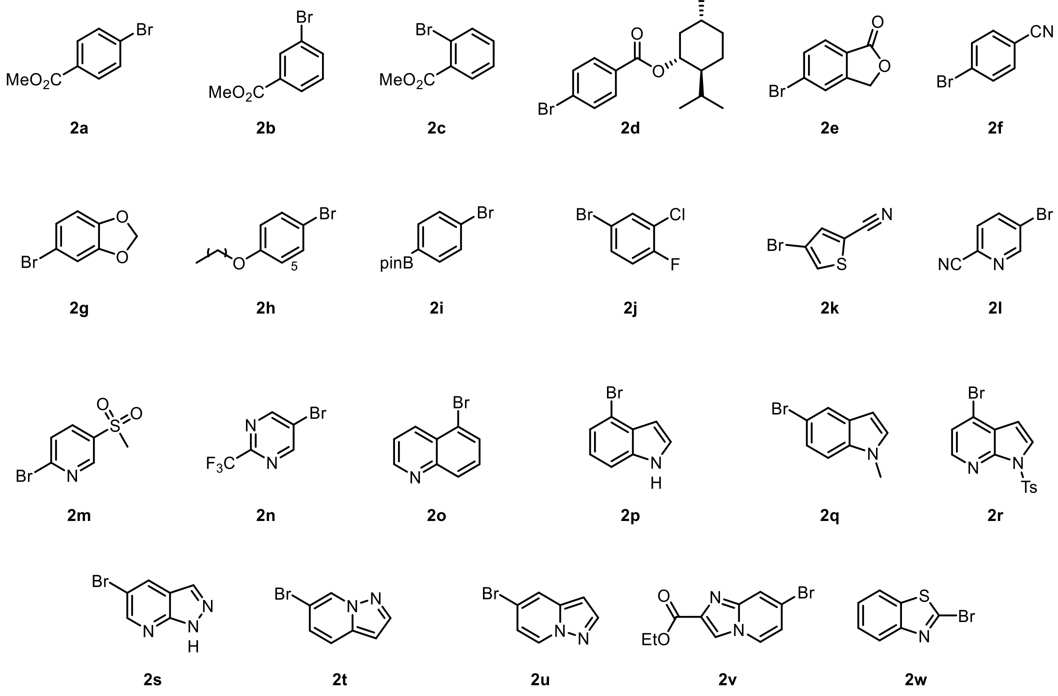

Supplementary Figure 8: **Aryl bromides used as aryl counterparts in this work.**

1.3.3 Starting materials

Synthesis of olefin:

General procedure 1: to a flame-dried, round-bottomed flask was added Methyltriphenylphosphonium iodide (1.5 equiv, 6.1 g, 15 mmol) and potassium *tert*-butoxide (1.5 equiv, 1.7 g, 15 mmol). anhydrous THF (30 mL) under N_2. Then the solution of aldehyde or ketone (10 mmol in 10 mL THF) was dropwise. The reaction mixture was allowed to stir at rt for 60 min and was then diluted with diethyl ether. The solution was filtered over a pad of silica gel and the filtrate was concentrated in vacuo. The crude product was purified by column chromatography on SiO_2 to afford the title compound. All spectral data were in accordance with the literature.

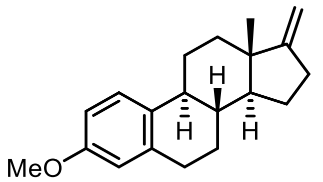

(8S,9S,13S,14S)-3-Methoxy-13-methyl-17-methylene-7,8,9,11,12, 13,14,15,16,17-decahydro-6H-cyclopenta[a]phenanthrene (1g). Compound **1g** was synthesized following a procedure reported in the literature.[1]

^{1}H NMR (300 MHz, $CDCl_3$) δ 7.24 (dd, J_1 = 9 Hz, J_2 = 1 Hz, 1H), 6.73 (dd, J_1 = 9 Hz, J_2 = 3 Hz, 1H), 6.65 (d, J = 3 Hz, 1H), 4.69 – 4.68 (m, 2H), 3.79 (s, 3H), 3.00 – 2.78 (m, 2H), 2.64 – 2.47 (m, 1H), 2.47 – 2.16 (m, 3H), 2.01 – 1.91 (m, 2H), 1.88 – 1.76 (m, 1H), 1.67 – 1.18 (m, 6H), 0.83 (s, 3H). ^{13}C NMR (75 MHz, $CDCl_3$) δ 161.9, 157.6, 138.2, 133.0, 126.5, 113.9, 111.6, 101.0, 55.3, 53.6, 44.5, 44.2, 38.9, 35.9, 30.0, 29.6, 27.7, 26.8, 24.0, 18.7. Spectroscopic data are in accordance with the literature.[1]

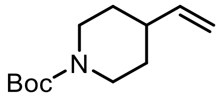

***tert*-Butyl 4-vinylpiperidine-1-carboxylate (1o).** Compound **1o** was synthesized following the GP1.

^{1}H NMR (300 MHz, $CDCl_3$) δ 5.77 (ddd, J_1 = 17 Hz, J_2 = 10 Hz, J_3 = 6 Hz, 1H), 5.07 – 4.90 (m, 2H), 4.08 (d, J = 13 Hz, 2H), 2.73 (t, J = 13 Hz, 2H), 2.19 – 2.01 (m, 1H), 1.73 – 1.62 (m, 2H), 1.45 (s, 9H), 1.27 (qd, J_1 = 12 Hz, J_2 = 4 Hz, 2H). ^{13}C NMR (75 MHz, $CDCl_3$) δ 155.0, 142.8, 113.1, 79.4, 43.9, 39.9, 31.5, 28.6. Spectroscopic data are in accordance with the literature.[2]

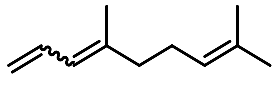

4,8-Dimethylnona-1,3,7-triene (1v). Compound **1v** was synthesized following the GP1.

^{1}H NMR (300 MHz, $CDCl_3$) δ 6.58 (dtd, *J* = 17, 11, 4 Hz, 1H), 5.92 – 5.80 (m, 1H), 5.18 – 5.01 (m, 2H), 4.97 (td, *J* = 10, 2 Hz, 1H), 2.25 – 2.00 (m, 4H), 1.81 – 1.75 (m, 3H), 1.69 (s, 2H), 1.61 (s, 2H). ^{13}C NMR (75 MHz, $CDCl_3$) δ 139.9, 139.7, 133.6, 133.3, 132.1, 131.9, 126.5, 125.6, 124.1, 124.0, 114.7, 114.5, 40.0, 32.6, 27.0, 26.7, 25.8, 23.9, 17.8, 17.8, 16.8. Spectroscopic data are in accordance with the literature.[3]

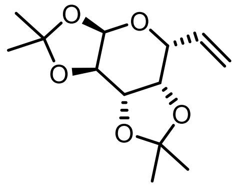

(3aR,5R,5aS,8aS,8bR)-2,2,7,7-Tetramethyl-5-vinyltetrahydro-5H-bis([1,3]dioxolo)[4,5-b:4',5'-d]pyran (1w). Compound **1w** was synthesized following a procedure reported in the literature.[4]

^{1}H NMR (300 MHz, $CDCl_3$) δ 6.01 – 5.85 (m, 1H), 5.58 (d, *J* = 5 Hz, 1H), 5.43 – 5.23 (m, 2H), 4.62 (dd, J_1 = 8 Hz, J_2 = 2 Hz, 1H), 4.35 – 4.26 (m, 2H), 4.23 (dd, J_1 = 8 Hz, J_2 = 2 Hz, 1H), 1.54 (s, 3H), 1.47 (s, 3H), 1.34 (s, 6H). ^{13}C NMR (75 MHz, $CDCl_3$) δ 134.0, 117.5, 109.4, 108.6, 96.6, 73.6, 71.0, 70.6, 69.1, 26.3, 26.1, 25.1, 24.5. Spectroscopic data are in accordance with the literature.[4]

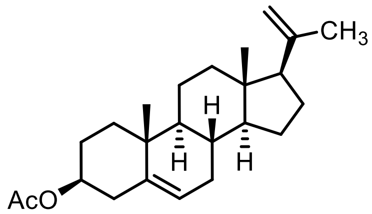

(3S,8S,9S,10R,13S,14S,17R)-10,13-Dimethyl-17-(prop-1-en-2-yl)-2,3,4,7,8,9,10,11,12,13,14,15,16,17-tetradecahydro-1H-cyclopenta[a]phenanthren-3-yl acetate (1x). Compound **1x** was synthesized following a procedure reported in the literature.[1]

^{1}H NMR (300 MHz, $CDCl_3$) δ 5.42 – 5.34 (m, 1H), 4.88 – 4.82 (m, 1H), 4.74 – 4.68 (m, 1H), 4.67 – 4.53 (m, 1H), 2.38 – 2.27 (m, 2H), 2.03 (s, 3H), 2.07 – 1.94 (m, 2H), 1.91 – 1.36 (m, 11H), 1.76 (s, 3H), 1.29 – 1.07 (m, 4H), 1.02 (s, 3H), 1.02 – 0.93 (m, 1H), 0.59 (s, 3H). ^{13}C NMR (75 MHz, $CDCl_3$) δ 170.7, 145.8, 139.8, 122.7, 110.8, 74.1, 57.4, 56.6, 50.3, 43.2, 38.8, 38.3, 37.2, 36.8, 32.3, 32.0, 27.9, 25.5, 24.8, 24.4, 21.6, 21.2, 19.5, 12.8. Spectroscopic data are in accordance with the literature.[1]

1.4 Optimization of reaction conditions

*1.4.1 Optimization of conditions for reaction with triethyl borane (**1a**)*

The optimization of the reaction conditions was carried out by studying the addition of triethyl borane (**1a**) onto methyl 4-bromobenzoate (**2a**) to give methyl 4-ethylbenzoate (**3**) on a 0.1 mmol scale (see Supplementary Supplementary Table 1-6).

In a 7 mL vial equipped with a screw cap **2a** (0.1 mmol), base (*n* equiv.), nickel salt (*n* equiv.), ligand (*n* equiv.), and the chosen photocatalyst (*n* mol%) were dissolved in 1.0 mL of the chosen solvent. The mixture was sonicated for 10 minutes to obtain a bright green solution and then bubbled with N_2 (1 min); hence, **1a** (0.1 mL of a 1.0 M solution in THF, 1 equiv.) was added. The mixture was irradiated for the indicated time with a 40 W Kessil lamp (λ = 456 nm, full intensity) for the required time in the UFO reactor (see **Supplementary Figure 2**). After irradiation, the solvent was removed, the crude was suspended in EtOAc and filtered through a short silica plug in a Pasteur pipette to remove solids. The solvent was removed and the sample was analyzed via ^{1}H-NMR ($CDCl_3$, CH_2Br_2 as external standard).

Screening of photocatalysts

Supplementary Table 1: Screening of photocatalysts.

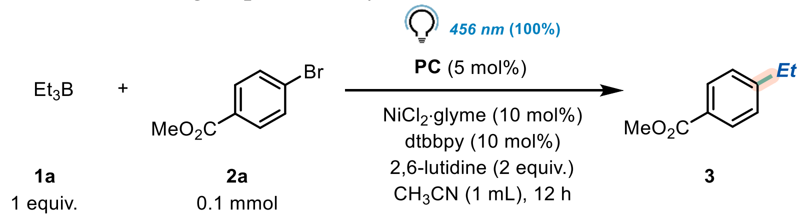

Entry	Photocatalyst	Yield (%)[a]
1	**PC1**	23
2	**PC2**	82
3	**PC3**	93
4	**PC4**	87

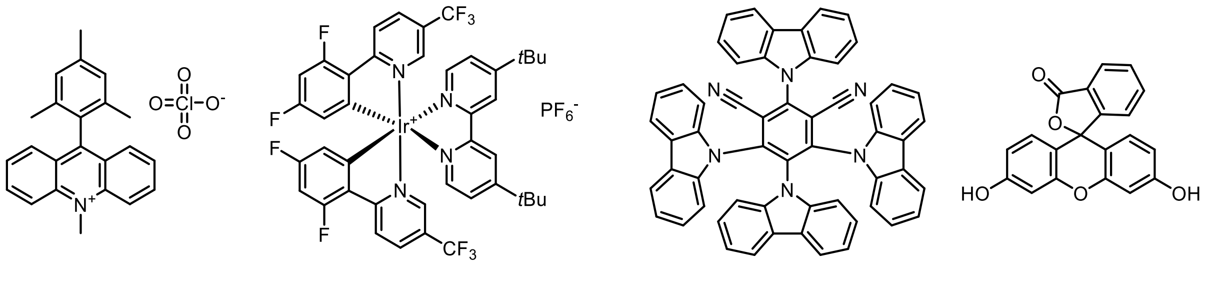

PC1: Mes-AcrClO$_4$ **PC2**: (Ir[dF(CF$_3$)ppy]$_2$(dtbpy))PF$_6$ **PC3**: 4CzIPN **PC4**: Fluorescein

[a] Yields determined by ^{1}H-NMR, CH_2Br_2 as external standard.

Screening of solvents

Supplementary Table 2: Screening of solvents.

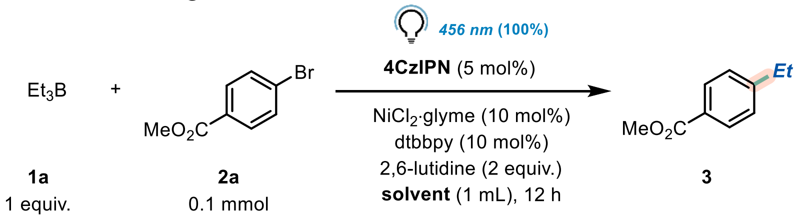

Entry	Solvent	Yield (%)[a]
1	CH_3CN	93
2	CH_3OH	86
3	CH_2Cl_2	63
4	THF	80
5	EtOAc	88
6	CH_3CN/DMSO (8:2)	60
7	CH_3CN/H_2O (10 equiv.)	85
8	Anhydrous CH_3CN	83

[a] Yields determined by ^{1}H-NMR, CH_2Br_2 as external standard.

Screening of bases

Supplementary Table 3: Screening of base.

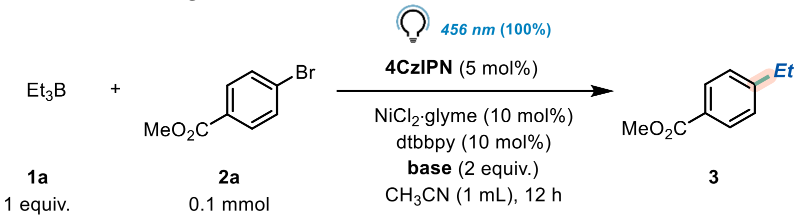

Entry	Base	Yield (%)[a]
1	Pyridine	30
2	Et_3N	23
3	DMAP	63
4	Cs_2CO_3	83
5	K_3PO_4	85
6	2,6-lutidine	93
7	2,6-lutidine (1.2 equiv.)	81

[a] Yields determined by ^{1}H-NMR, CH_2Br_2 as external standard.

Screening of nickel source and ligands

Supplementary Table 4: Screening of nickel source and ligand.

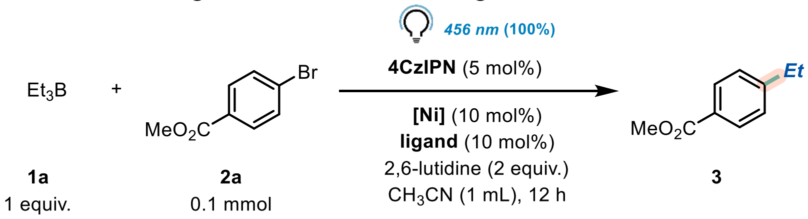

Entry	Ni^{II}	Yield (%)[a]
1	$NiBr_2$ glyme, **L1**	90
2	$Ni(acac)_2$, **L1**	93

3	$NiCl_2$·glyme, **L1**	93
4	$NiCl_2$·glyme, **L3**	76

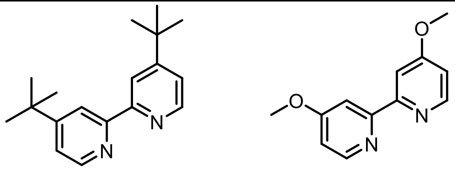

L1 **L3**

a Yields determined by NMR, CH_2Br_2 as external standard.

Screening of reaction time and catalyst loading

Supplementary Table 5: Screening of catalyst loading and reaction time.

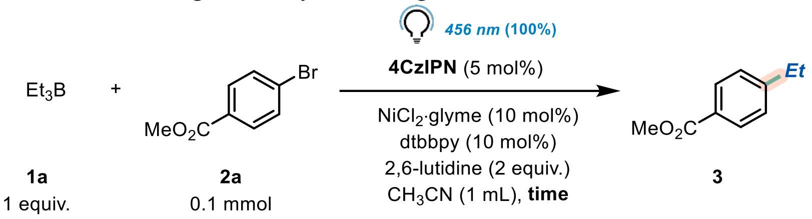

Entry	Variation from conditions	Yield (%)[a]
1	3 h	92
2	**4CzIPN** (2 mol%), $NiCl_2$·glyme and dtbbpy (5 mol%), 3 h	82

a Yields determined by ^{1}H-NMR, CH_2Br_2 as external standard.

Control experiments

Supplementary Table 6: Control experiments.

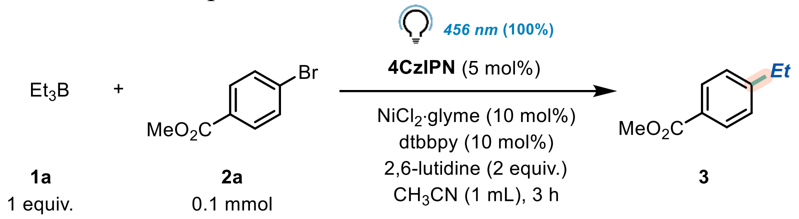

Entry	Variation from conditions	Yield (%)[a]
1	Without **PC3**	n.d.
2	Without ligand	6
3	Without base	8
4	Without $NiCl_2$·glyme and dtbbpy	n.d.
5	Without light	n.d.
6	Without light, under air	n.d.
7	No light and heating 80 °C	7
8	Under Air	39%
9	0.33 equiv. **1a**	33%
10	$BuB(OH)_2$ instead of **1a**	n.d.

a Yields determined by ^{1}H-NMR, CH_2Br_2 as external standard.

1.4.2 Optimization of reaction conditions with 9-borabicyclo[3.3.1]nonane (9-BBN)

The optimization of the reaction conditions was carried out by studying the coupling of alkyl-9-BBN **S1** with methyl 4-bromobenzoate (**2a**) to give methyl 4-(3-phenoxypropyl)benzoate (**32**) on a 0.1 mmol scale (see Supplementary Supplementary Table 7-10).

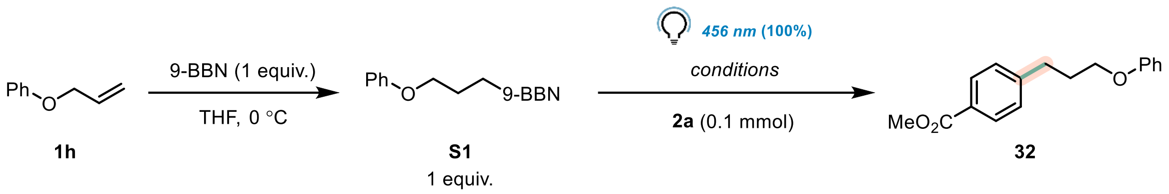

<u>*Synthesis of* ***S1***</u>

S1 was prepared via hydroboration according to a procedure reported in the literature.[5,6] In particular, 2 mL of 9-BBN solution in THF (0.5 M, 1.0 mmol, 1.0 equiv.) were added to a flame-dried Schlenk flask equipped with a stirring bar at 0 °C (ice bath). The olefin (1.0 mmol, 1.0 equiv.) was added neat dropwise. After addition, the ice bath was removed and the reaction was stirred at room temperature for 1 hour. The reaction was monitored via ^{1}H-NMR and it was considered finished when conversion of the olefin was found higher than 95%.

In a 7 mL vial equipped with a screw cap, **2a** (0.1 mmol), base (*n* equiv.), nickel salt (*n* equiv.), ligand (*n* equiv.), and 4CzIPN (*n* mol%) were dissolved in 1.0 mL of the chosen solvent. The mixture was sonicated for 10 minutes to obtain a bright green solution and then bubbled with N_2 (1 min); hence, the desired aliquot of **S1** (as a solution in THF) was added. The mixture was irradiated for the indicated time with a 40 W Kessil lamp (λ = 456 nm, full intensity) for the required time in the UFO reactor (see **Supplementary Figure 2**). After irradiation, the solvent was removed, the crude was suspended in EtOAc and filtered through a short silica plug in a Pasteur pipette to remove solids. The solvent was removed and the sample was analyzed via ^{1}H-NMR ($CDCl_3$, CH_2Br_2 as external standard).

Screening of different bases

Supplementary Table 7: Screening of different base

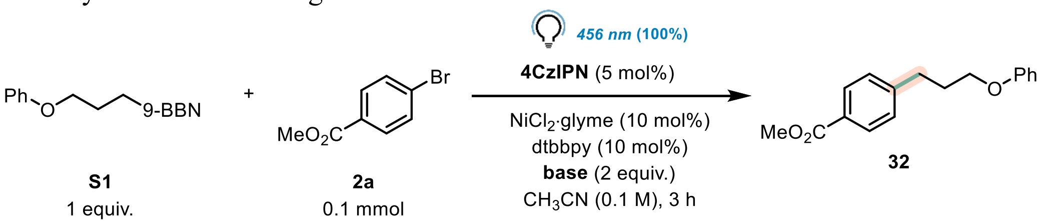

Entry	Base	Yield (%)[a]
1	2,6-lutidine	5
2	K_3PO_4	8

3	pyridine	13
4	DMAP	30
5	2-picoline	5

[a] Yields determined by ^{1}H-NMR, CH_2Br_2 as external standard.

Screening of different solvent

Supplementary Table 8: Screening of different solvent

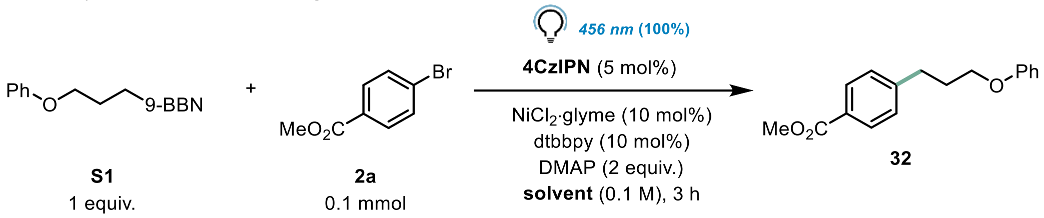

Entry	Solvent	Yield (%)[a]
1	THF	n.d.
2	Dioxane	n.d.
3	CH_2Cl_2	5
4	CH_3OH	31
5	CH_3CN	30

[a] Yields determined by ^{1}H-NMR, CH_2Br_2 as external standard.

Screening stochiometric ratio

Supplementary Table 9: Screening stochiometric ratio

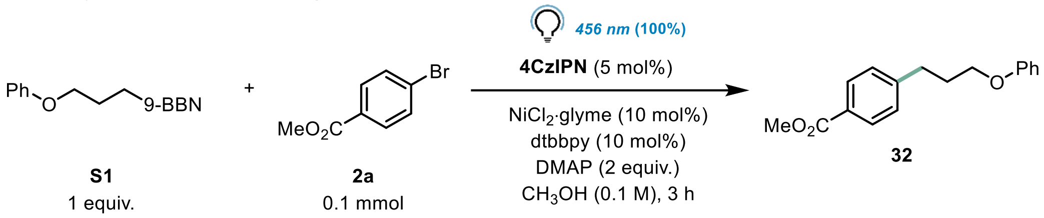

Entry	**S1**	Yield (%)[a]
1	1.0 equiv.	31
2	1.5 equiv.	43
3	2.0 equiv.	63
4	2.5 equiv.	60

[a] Yields determined by ^{1}H-NMR, CH_2Br_2 as external standard.

Control experiments

Supplementary Table 10: Control experiments

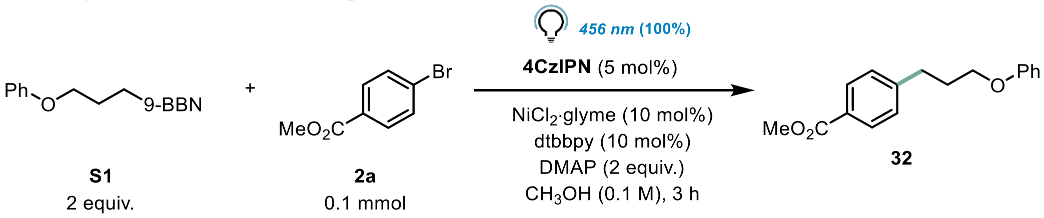

Entry	Variation from conditions	Yield (%)[a]
1	Without **PC3**	n.d.
2	Without base	n.d.
3	Without $NiCl_2$·glyme and dtbbpy	n.d.

4	Without dtbbpy	44
5	No light and heating 80 ºC	n.d.

[a] Yields determined by ^{1}H-NMR, CH_2Br_2 as external standard.

1.4.3 Adjustment of reaction conditions with 9-borabicyclo[3.3.1]nonane (9-BBN) for continuous-flow

Optimization of hydroboration:

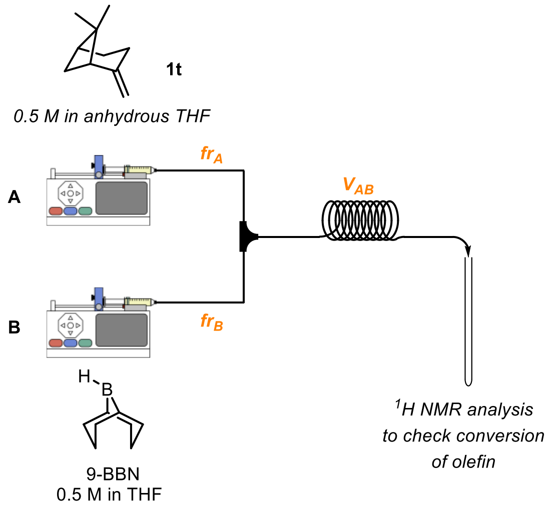

Supplementary Figure9: **Setup used for the optimization of the hydroboration step in flow**.

Solution A: (-)-β-Pinene (0.5 M in anhydrous THF).

Solution B: 9-Borabicyclo[3.3.1]nonane solution (0.5 M in THF).

Solutions **A** and **B** were taken up with plastic syringes and mounted on two syringe pumps. The syringes were connected to PFA capillary tubing (0.8 mm inner diameter) and the liquid feeds were pumped: the two streams were connected via a PEEK T-mixer and flowed through a PFA tubing. Different residence times were screened to ensure complete conversion of **1t**: in detail, the crude was directly collected in NMR tubes and checked the conversion by ^{1}H NMR. Results are shown in Supplementary Table 11.

Supplementary Table 11: Optimization in flow.

Entry	fr_A/mL·min^{-1}	fr_B/mL·min^{-1}	V_{AB}/mL	t_R/min	Conv. **1t** (%)
1	0.04	0.04	0.8	10	Trace
2	0.0133	0.0133	0.8	30	79
3	0.021	0.021	2.5	60	99

[a] Conversion determined by ^{1}H-NMR, trichloroethene as external standard.

Optimization of photoreaction in flow:

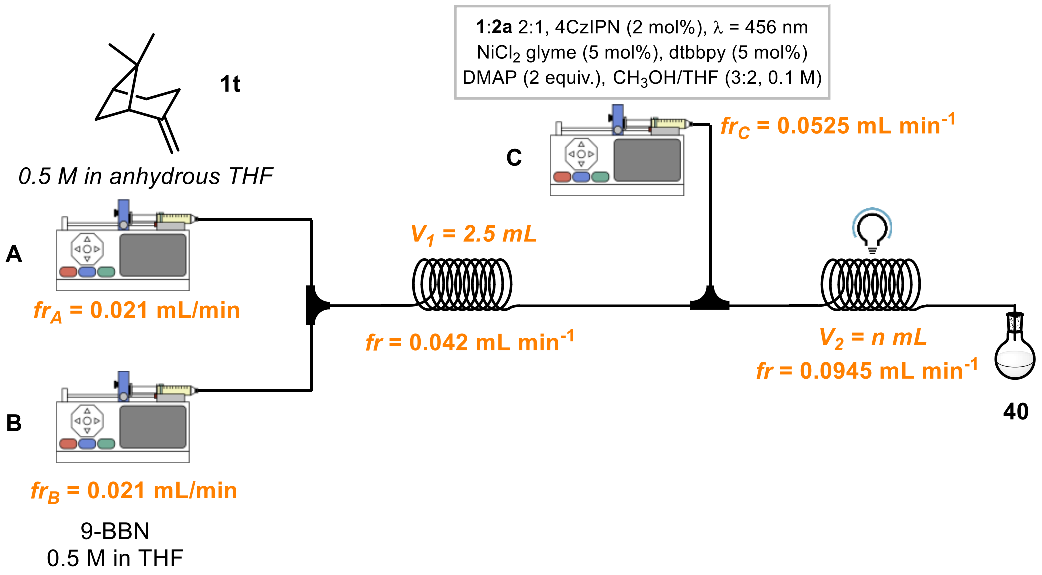

Supplementary Figure10: **Setup used for the optimization of the telescoped reaction in flow**.

Solution A: (-)-β-Pinene (0.5 M in anhydrous THF);

Solution B: 9-Borabicyclo[3.3.1]nonane solution (0.5 M in THF);

Solution C: **2a** (0.1 mmol, 21.5 mg), $NiCl_2$·glyme (n mol%) and dtbbpy (n mol), **PC3** (n mol%) and 4-dimethylaminopyridine (2 equiv.) were dissolved in CH_3OH/THF (0.1 M, 3:2). The reaction solution was degassed with N_2 (1 min).

Solutions **A** and **B** were taken up with plastic syringes and mounted on two syringe pumps. The syringes were connected to PFA capillary tubing (0.8 mm inner diameter) and the liquid feeds were pumped: the two streams were connected via a PEEK T-mixer and flowed through a PFA coil at room temperature (V = 2.5 mL, f_{A+B} = 0.042 mL·min^{-1}, t_R = 60 min). The outflow of this reactor was merged with another containing solution C: the combined feeds were then flowed through the UFlow (**Supplementary Figure 4**) equipped with a 4.5 mL PFA tubing (0.8 mm internal diameter) and a Kessil lamp (λ = 456 nm, 40 W). When the syringe was fully empty, neat CH_3CN was again loaded into a syringe and injected to collect all product at the end of the reactor in a flask. Afterwards, the solvent was removed, the crude was suspended in EtOAc and filtered through a short silica plug in a Pasteur pipette to remove solids. The solvent was removed and the sample was analyzed via ^{1}H-NMR ($CDCl_3$, CH_2Br_2 as external standard). Results are shown in Supplementary Supplementary Table12.

Supplementary Table12: Screening the condition of photoreaction in flow.

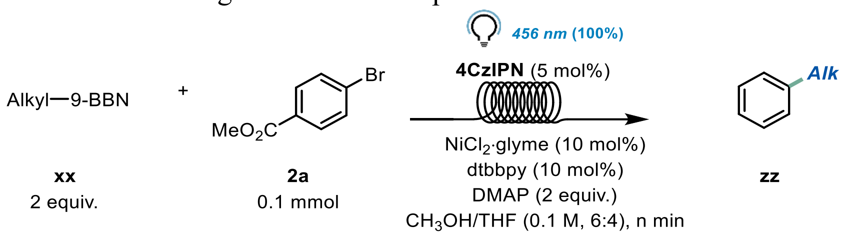

Entry	Variation from conditions	V_2/mL	t_R/min	Yield **40** (%)
1	none	1.9	20	70
2	none	4.5	48	77
3	2 mol% **PC3**, 5 mol% $NiCl_2$·glyme, dtbbpy	4.5	48	86

*1.4.4 Adjustment of reaction conditions with triethyl borane (**1a**) for continuous-flow*

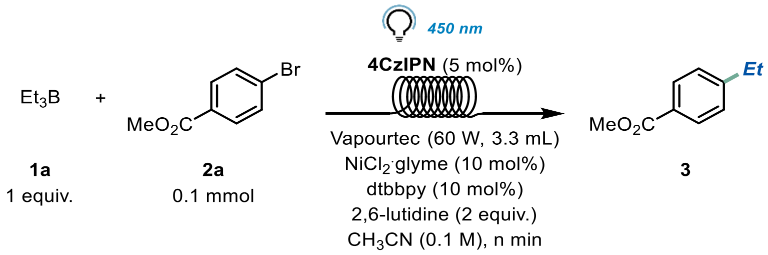

Entry	Variation from conditions	t_R/min	Yield **3** (%)
1	none	20	76
2	none	10	70
3	2 mol% **PC3**, 5 mol% $NiCl_2$·glyme, dtbbpy	10	86

1.5 General procedures (GPs) for preparative experiments

1.5.1 GP2: batch conditions with commercial available alkyl borane

A CH_3CN (0.1 M) solution containing the aryl bromide **2** (0.5 mmol), $NiCl_2$·glyme and dtbbpy (10 mol%), **PC3** (5 mol%) and 2,6-lutidine (2 equiv.) was prepared in a 7 mL vial equipped with a screw cap and a stirring bar. The solution sonicated until fully dissolved and then was bubbled with N_2 (5 min). Triethylborane (0.5 M in Hexane) **1a** (1 mL, 1.0 equiv.) was added. Then the solution was irradiated by adopting the setup shown in **Supplementary Figure 2** equipped with a 40 W Kessil lamp (λ = 456 nm, full intensity) for 3 h. The solutions were collected, solvent was removed under reduced pressure and the crude was purified via column chromatography on silica gel to provide the expected product.

1.5.2 GP3 (Method A): batch conditions – via hydroboration of olefins with THF·BH$_3$

Step 1: Hydroboration. 1.1 mL of BH_3 solution in THF (1 M, 1.1 mmol) was added to a flame-dried Schlenk flask equipped with a stirring bar at 0 °C (ice bath). The olefin (3.0 mmol) was added neat dropwise. After addition, the ice bath was removed and the reaction was stirred at room temperature for 1 hour. The reaction was monitored via ^{1}H-NMR without any purification. (In case of solid olefins, the substrate was added to a flame-dried Schlenk flask under a nitrogen atmosphere and dissolved using the minimum amount of anhydrous THF. Hence, THF-BH_3 was added dropwise at ^{0}C, after which the cold bath was removed. The solution was left stirring for 1 h at room temperature.)

Step 2: Photoreaction solution. A CH_3CN (0.1 M) solution containing the aryl bromide **2** (0.5 mmol), $NiCl_2$·glyme and dtbbpy (10 mol%), **PC3** (5 mol%) and 2,6-lutidine (2 equiv.) was prepared in a 7 mL vial equipped with a screw cap and a stirring bar. The solution was sonicated until fully homogeneous and then bubbled with N_2 (5 min). Alkyl borane from Step 1 (in THF) **1** (1 equiv.) was added. Then the solution was irradiated by adopting the setup shown in **Supplementary Figure 2** equipped with a 40 W Kessil lamp (λ = 456 nm, full intensity) for 3 h. The solvent was removed under reduced pressure and the crude was purified via column chromatography on silica gel to provide the expected product.

1.5.3 GP4 (Method B): batch conditions - via hydroboration of olefins with 9-BBN

Step 1: Hydroboration. 2 mL of 9-BBN solution in THF (0.5 M, 1.0 mmol, 1.0 equiv.) were added to a flame-dried Schlenk flask equipped with a stirring bar at 0 °C (ice bath). The olefin (1.0 mmol, 1.0 equiv.) was added neat dropwise. After addition, the ice bath was removed and

the reaction was stirred at room temperature for 1 hour. The reaction was monitored via ^{1}H-NMR without any purification. (In case of solid olefins, the substrate was added to a flame-dried Schlenk flask under a nitrogen atmosphere and dissolved using the minimum amount of anhydrous THF. Hence, 9-BBN was added dropwise at ^{0}C, after which the cold bath was removed. The solution was left stirring for 1 h at room temperature.)

Step 2: Photoreaction solution. A CH_3CN (0.1 M) solution containing the aryl bromide **2** (0.5 mmol), $NiCl_2$·glyme and dtbbpy (10 mol%), **PC3** (5 mol%) and 4-dimethylaminopyridine (2 equiv.) was prepared in a 7 mL vial equipped with a screw cap and a stirring bar. The solution sonicated until fully dissolved and then was bubbled with N_2 (5 min). Alkyl borane from Step 1 (in THF) **1** (2 equiv.) was added. Then the solution was irradiated by adopting the setup shown in **Supplementary Figure 2** equipped with a 40 W Kessil lamp (λ = 456 nm, full intensity) for 3 h. The solvent was removed under reduced pressure and the crude was purified via column chromatography on silica gel to provide the expected product.

1.5.4 GP5: continuous flow conditions with 9-BBN, blue light (456 nm)

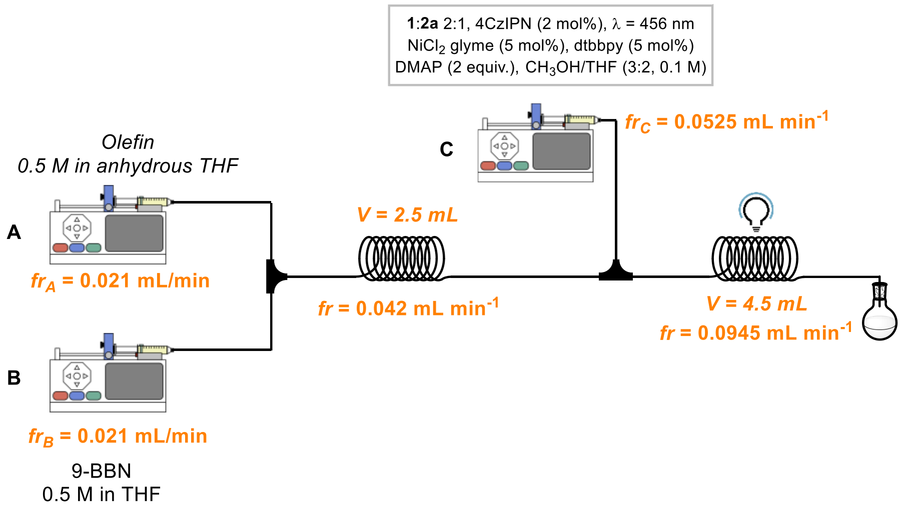

Supplementary Figure11: **Sketch of the setup used for GP5**.

Solution A: Olefin (0.5 M in anhydrous THF);

Solution B: 9-Borabicyclo[3.3.1]nonane solution (0.5 M in THF);

Solution C: **2a** (1.0 mmol, 215 mg), $NiCl_2$·glyme (5 mol%, 0.05 mmol, 11 mg) and dtbbpy (5 mol%, 0.05 mmol, 13.4 mg), **PC3** (2 mol%, 0.02 mmol, 15.8 mg) and 4-dimethylaminopyridine (2 equiv., 2 mmol, 244 mg) was prepared in a 10 mL volumetric flask, then dissolved in CH_3OH/THF (6:4). The reaction solution was degassed with N_2 (5 min).

Solutions **A** and **B** were taken up with plastic syringes and mounted on two syringe pumps set at 0.021 mL/min. **A** and **B** were mixed by means of a T-mixer and flowed through a 2.5 mL (or 3.8 mL) reactor (PFA capillary tubing, 0.8 mm inner diameter) at 0.042 mL/min (corresponding to 60 or 90 minutes residence time). Next, solution **C** was combined (flow rate: 0.0525 mL/min) via plastic syringe before entering an UFlow reactor equipped with a 4.5 mL coil (total flow rate: 0.0945 mL/min, corresponding to 48 minutes residence time). When the syringe was fully empty, again CH_3CN was loaded into a syringe and injected to collect all product at the end of the reactor in a flask. The crude was concentrated under vacuum and extracted with EtOAc/H_2O. The combined organic layers were washed with brine, dried with $MgSO_4$, filter and evaporated. The crude was purified via column chromatography on silica gel (Pentane : Ethyl Acetate) to afford the product.

*1.5.5 Scale-up for the synthesis of compound **44** in continuous-flow*

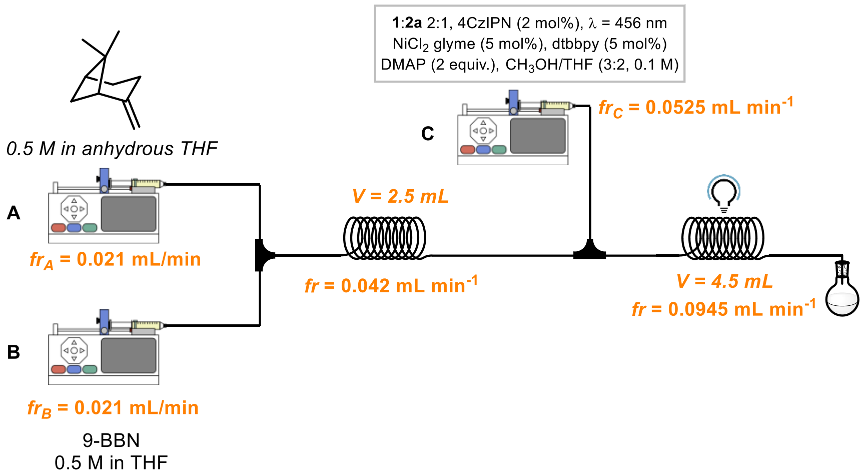

Supplementary Figure 12: **Sketch of the setup used for the scale-up of compound 44**.

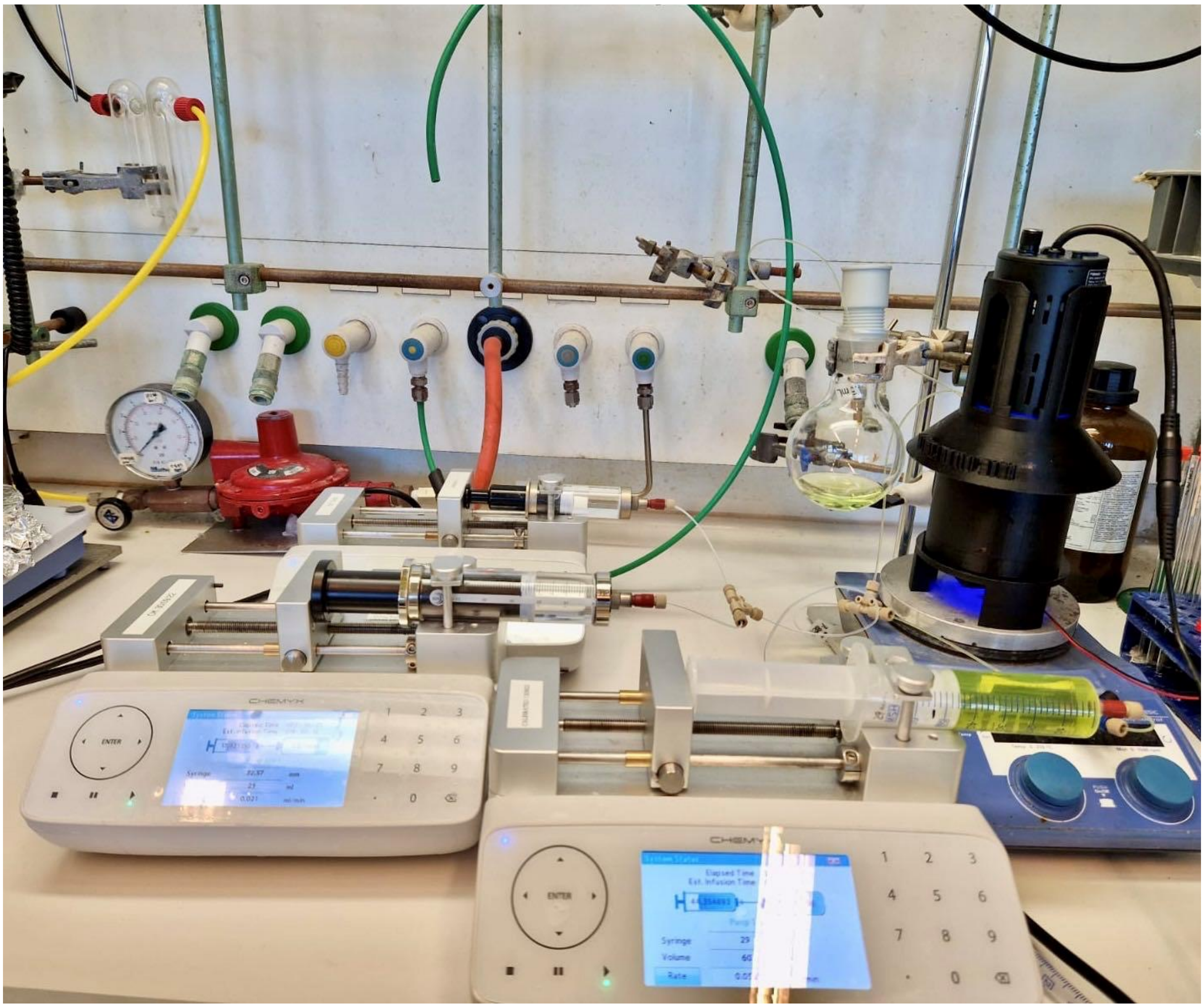

Supplementary Figure 13: **Picture of the setup used for the scale-up of compound 44**.

Solution A: (−)-β-Pinene (0.5 M in anhydrous THF);

Solution B: 9-Borabicyclo[3.3.1]nonane solution (0.5 M in THF);

Solution C: **2a** (25 mmol, 5.4 g), $NiCl_2$·glyme (5 mol%, 1.3 mmol, 0.27 g) and dtbbpy (5 mol%, 1.3 mmol, 0.34 g), **PC** (2 mol%, 0.5 mmol, 0.39 g) and 4-dimethylaminopyridine (2 equiv., 50 mmol, 6.1 g) was prepared in a 250 mL volumetric flask, then dissolved in CH_3OH/THF (6:4). The reaction solution be transferred to a Schlenk flask then freeze-pump-thaw degassing.

The solution **A** and **B** was taken up with SGE gas tight syringes and mounted on a syringe pump. The syringe was connected to a 2.5 mL reactor (PFA capillary tubing, 0.8 mm inner diameter). The liquid feed was pumped into the flow reactor at 0.021 mL/min (corresponding to 60 minutes residence time). The loop was connected with Uflow reactor. After 60 min, solution **C** was pumped via plastic syringe into 4.5 mL Uflow reactor at 0.0525 mL/min (the total flow rate: 0.0945 mL/min, corresponding to 48 minutes residence time). When the syringe was fully empty, again CH_3CN was loaded into a syringe and injected to collect all product at

the end of the reactor in a flask. Uflow (λ = 456 nm, 40 W, **Supplementary Figure 4**) was used. The solutions were collected, then removed solvent, diluted with H_2O and EtOAc. The layers were separated and the aqueous layer was extracted with EtOAc (×2). The combined organic layers was washed with brine, dried with $MgSO_4$, filter and evaporated. The crude was purified via column chromatography on silica gel (Pentane : Ethyl Acetate 95:5) to afford the product as colourless oil **44** (5.3 g, 78%).

2. Supplementary Discussion

2.1 Mechanistic investigation

2.1.1 Preliminary evaluation of reaction profile

Before conducting a kinetic study of the model reaction between **1a** and **2a** via initial rates determination, the reaction was performed under optimized conditions (Supplementary Supplementary Table 5, entry 1) and monitored in time.

Thus, in a 5 mL volumetric flask, a solution of **2a** (0.1 M), $NiCl_2$·glyme (10 mol%), dtbbpy (10 mol%), and 4CzIPN (5 mol%) was prepared in some CH_3CN. The mixture was sonicated for 10 minutes to obtain a bright green solution; next, 2,6-lutidine (2 equiv.) was added and the solution was diluted to volume with CH_3CN. The solution was transferred to four 7 mL-vials equipped with a stirring bar and a screw cap (1 mL each), the solutions were bubbled with N_2 (1 min), and then **1a** (1.0 M in hexanes, 0.1 mL each, 1 equiv.) was added with a syringe through the septum. The solutions were irradiated with a 40 W Kessil lamp (λ = 456 nm, full intensity) in the UFO kinetics reactor (**Supplementary Figure 3**) for the 0, 10, 30, 60 and 120 min and worked up as described in the reaction optimization section.

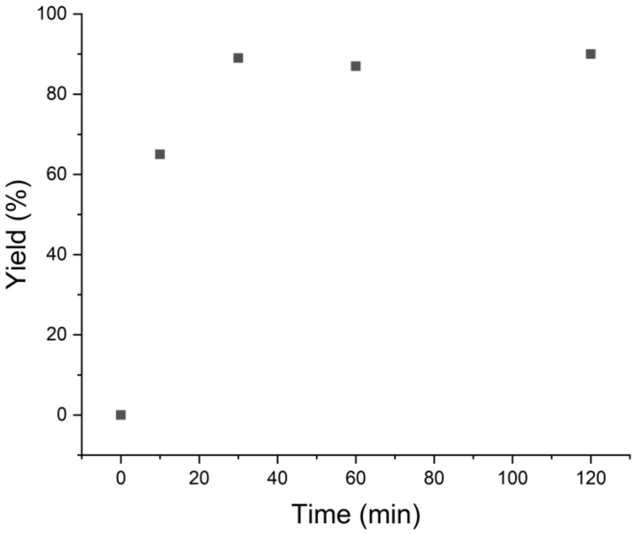

Supplementary Figure 14: Kinetic profile. Time profile of the reaction between **1a** with **2a** under optimized conditions.

Given the results shown in **Supplementary Figure 14**, in order to keep conversion below 25%, we performed our initial rates experiments in the first 4 minutes of irradiation.

2.1.2 Kinetics

With the optimized reaction condition in hand (Supplementary Supplementary Table 5, entry 1), the kinetics of the reaction were monitored by ^{1}H-NMR with CH_2Br_2 as external standard. Each reaction was monitored to 0-25% conversion.

*Initial rates evaluation by varying the concentration of **2a***: In a 5 mL volumetric flask, **2a** (75 mM, 100 mM, 125 mM or 150 mM), $NiCl_2$·glyme (10 mol%), dtbbpy (10 mol%), and 4CzIPN (5 mol%) were dissolved in some CH_3CN. The mixture was sonicated for 10 minutes to obtain a bright green solution; next, 2,6-lutidine (2 equiv.) was added and the solution was diluted to volume with CH_3CN. The solution was transferred to four 7 mL-vials equipped with a stirring bar and a screw cap (1 mL each), the solutions were bubbled with N_2 (1 min), and then **1a** (1.0 M in hexanes, 0.1 mL each, 1 equiv.) was added with a syringe through the septum. The final concentrations of **2a** are 68 mM, 91 mM, 114 mM or 136 mM, respectively. The solutions were irradiated with a 40 W Kessil lamp (λ = 456 nm, full intensity) in the UFO kinetics reactor for the indicated time and worked up as described in the reaction optimization section.

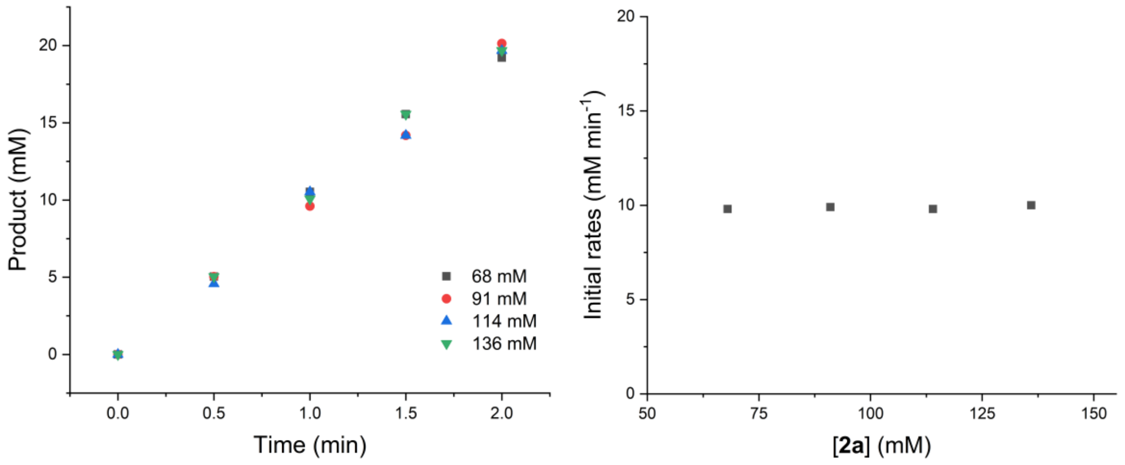

Supplementary Figure 15: **Initial rates at different concentrations of 2a**.

*Initial rates evaluation by varying the concentration of **1a***: In a 25 mL volumetric flask, **2a** (0.12 M), $NiCl_2$·glyme (10 mol%), dtbbpy (10 mol%), and 4CzIPN (5 mol%) were dissolved in some CH_3CN. The mixture was sonicated for 10 minutes to obtain a bright green solution; next, 2,6-lutidine (2 equiv.) was added and the solution was diluted to volume with CH_3CN. Aliquots of this solution were transferred to four 7 mL-vials equipped with a stirring bar and a screw cap (833 µL each, corresponding to 0.1 mmol of **2a**), the solutions were bubbled with N_2 (1 min), and then **1a** (1.0 M in hexanes; 75 µL, 100 µL, 125 µL or 150 µL) was added with a Hamilton syringe through the septum. Finally, neat degassed CH_3CN was added to reach a

total volume for each reaction of 1.1 mL. The final concentrations of **1a** are therefore 68 mM, 91 mM, 114 mM or 136 mM. The solutions were irradiated with a 40 W Kessil lamp (λ = 456 nm, full intensity) in the UFO kinetics reactor for the indicated time and worked up as described in the optimization of reaction conditions.

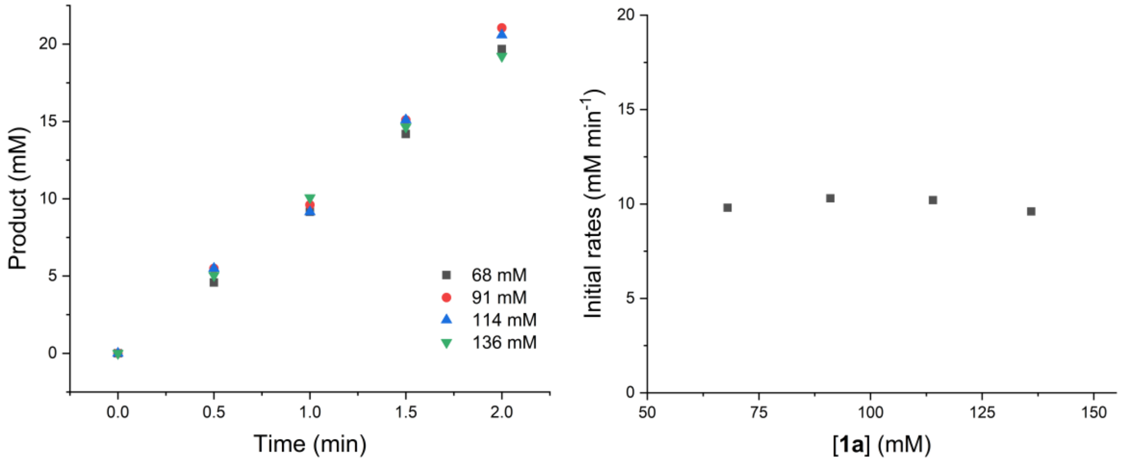

Supplementary Figure 16: **Initial rates at different concentrations of 1a**.

Initial rates evaluation by varying the concentration of 2,6-lutidine: In a 5 mL volumetric flask, **2a** (0.1 M), $NiCl_2$·glyme (10 mol%), dtbbpy (10 mol%), and 4CzIPN (5 mol%) were dissolved in some CH_3CN. The mixture was sonicated for 10 minutes to obtain a bright green solution; next, 2,6-lutidine (175 mM, 200 mM, 225 mM or 250 mM) was added and the solution was diluted to volume with CH_3CN. This solution was added to four 7 mL-vials equipped with a stirring bar and a screw cap (1 mL each), the solutions were bubbled with N_2 (1 min), and then **1a** (1.0 M in hexanes; 0.1 mL each, 1 equiv.) was added with a syringe through the septum. The final concentrations of 2,6-lutidine are therefore 159 mM, 182 mM, 204 mM or 227 mM. The solutions were irradiated with a 40 W Kessil lamp (λ = 456 nm, full intensity) in the UFO kinetics reactor for the indicated time and worked up as described in the optimization of reaction conditions.

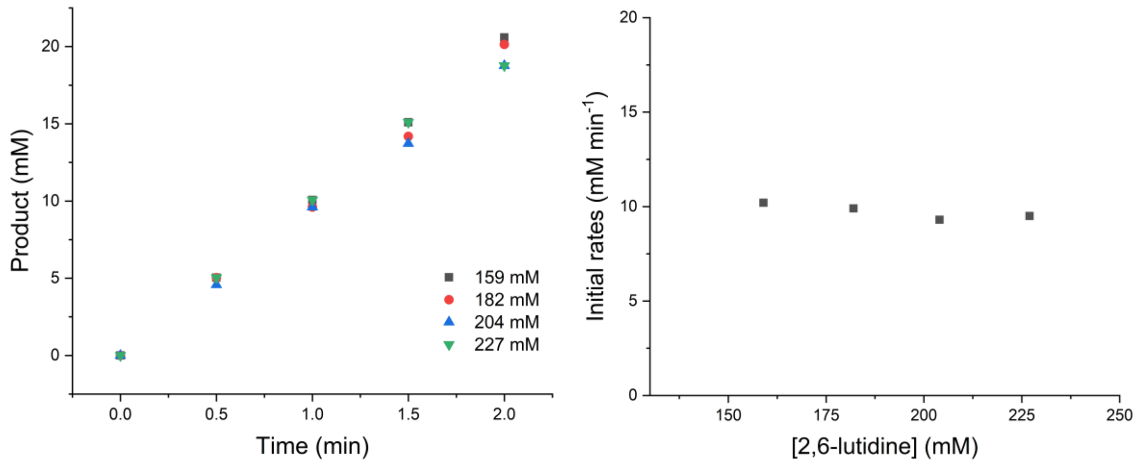

Supplementary Figure 17: **Initial rates at different concentrations of 2,6-lutidine**.

Initial rates evaluation by varying the concentration of 4CzIPN: In a 5 mL volumetric flask, **2a** (0.1 M), $NiCl_2$·glyme (10 mol%), dtbbpy (10 mol%), and 4CzIPN (1 mM, 2.5 mM, 5 mM or 7.5 mM) were dissolved in some CH_3CN. The mixture was sonicated for 10 minutes to obtain a bright green solution; next, 2,6-lutidine (2 equiv.) was added and the solution was diluted to volume with CH_3CN. This solution was transferred to four 7 mL-vials equipped with a stirring bar and a screw cap (1 mL each), the solutions were bubbled with N_2 (1 min), and then **1a** (1.0 M in hexanes; 0.1 mL each, 1 equiv.) was added with a syringe through the septum. The final concentrations of 4CzIPN are therefore 0.9 mM, 2.3 mM, 4.5 mM or 6.8 mM. The solutions were irradiated with a 40 W Kessil lamp (λ = 456 nm, full intensity) in the UFO kinetics reactor for the indicated time and worked up as described in the optimization of reaction conditions.

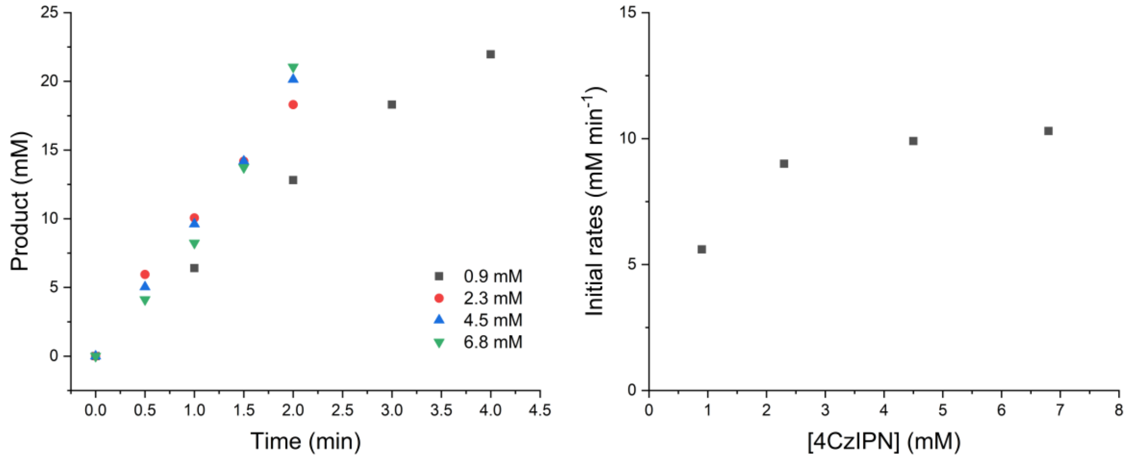

Supplementary Figure 18: **Initial rates at different concentrations of 4CzIPN**.

Initial rates evaluation by varying the light intensity: In a 5 mL volumetric flask, **2a** (0.1 M), $NiCl_2$·glyme (10 mol%), dtbbpy (10 mol%), and 4CzIPN (5 mol%) were dissolved in some CH_3CN. The mixture was sonicated for 10 minutes to obtain a bright green solution; next, 2,6-lutidine (2 equiv.) was added and the solution was diluted to volume with CH_3CN. This solution was transferred to four 7 mL-vials equipped with a stirring bar and a screw cap (1 mL each), the solutions were bubbled with N_2 (1 min), and then **1a** (1.0 M in hexanes; 0.1 mL each, 1 equiv.) was added with a syringe through the septum. The solutions were irradiated with a 40 W Kessil lamp (λ = 456 nm, 25%, 50%, 75% and 100% intensity) in the UFO kinetics reactor for the indicated time and worked up as described in the optimization of reaction conditions. The linear variation in light intensity was verified with a light meter (Extech SP505 Zonne-energiemeter 0 – 3999 $W{\cdot}m^{-2}$) with the 3D-setup shown in **Supplementary Figure 5**.

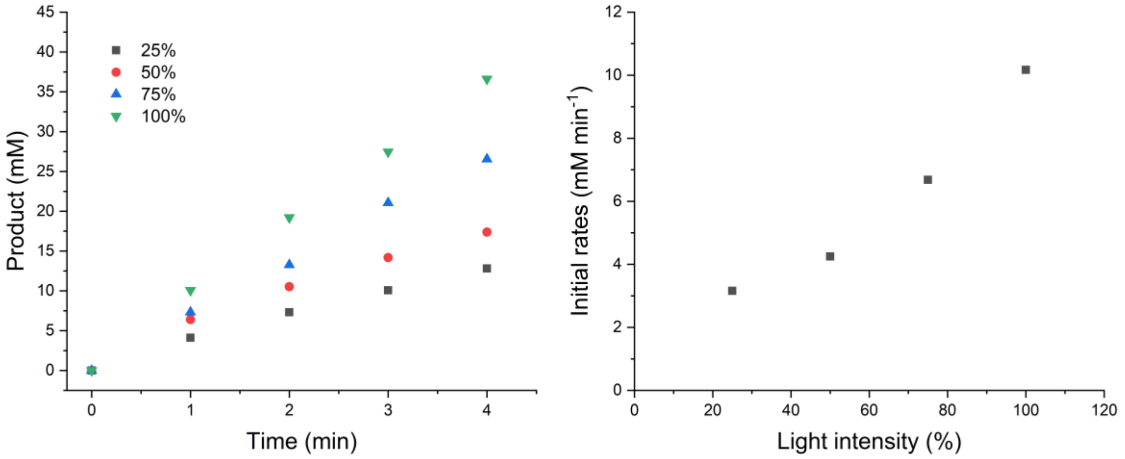

Supplementary Figure 19: **Initial rates at different light intensity.**

Initial rates evaluation by varying the concentration of $NiCl_2$·glyme: In a 5 mL volumetric flask, **2a** (0.1 M), $NiCl_2$·glyme (2 mM, 5 mM, 10 mM or 15 mM), dtbbpy (equimolar to nickel chloride), and 4CzIPN (5 mol%) was prepared in some CH_3CN. The mixture was sonicated for 10 minutes to obtain a bright green solution; next, 2,6-lutidine (2 equiv.) was added and the solution was diluted to volume with CH_3CN. This solution was transferred to four 7 mL-vials equipped with a stirring bar and a screw cap (1 mL each), the solutions were bubbled with N_2 (1 min), and then **1a** (1.0 M in hexanes; 0.1 mL each, 1 equiv.) was added with a syringe through the septum. The final concentrations of the nickel complex are therefore 1.8 mM, 4.5 mM, 9.1 mM or 13.6 mM. The solutions were irradiated with a 40 W Kessil lamp (λ = 456 nm, full intensity) in the UFO kinetics reactor for the indicated time and worked up as described in the optimization of reaction conditions.

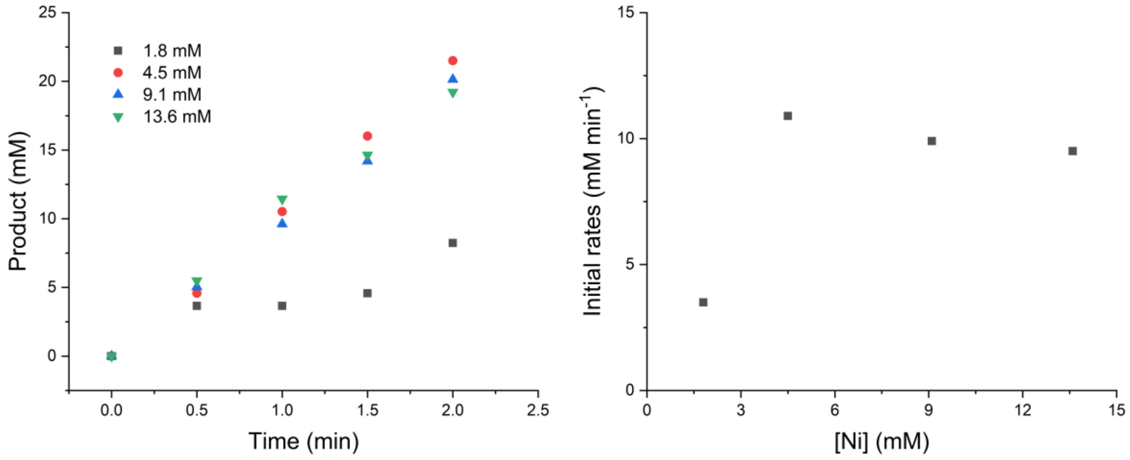

Supplementary Figure 20: **Initial rates at different concentrations of nickel**.

Taken altogether, under optimized conditions, the reaction shows zeroth order in aryl bromides, 2,6-lutidine and alkyl borane; moreover, we understood that the reaction runs in a photon-limited regime.

2.1.3 Hammett plot

A Hammett plot was also built to investigate the impact of the substituent on the aryl bromide on the course of the reaction. Hammett values were taken from the literature.[7]

In a 5 mL volumetric flask, the chosen aryl bromide (0.1 M), $NiCl_2$·glyme (10 mol%), dtbbpy (10 mol%), and 4CzIPN (5 mol%) were dissolved in some CH_3CN. The mixture was sonicated for 10 minutes to obtain a bright green solution; next, 2,6-lutidine (2 equiv.) was added and the solution was diluted to volume with CH_3CN. This solution was transferred to four 7 mL-vials equipped with a stirring bar and a screw cap (1 mL each), the solutions were bubbled with N_2 (1 min), and then **1a** (1.0 M in hexanes; 0.1 mL each, 1 equiv.) was added with a syringe through the septum. The solutions were irradiated with a 40 W Kessil lamp (λ = 456 nm, 100% intensity) in the UFO kinetics reactor for the indicated time and worked up as described in the optimization of reaction conditions. Each reaction was monitored to 0-20% conversion.

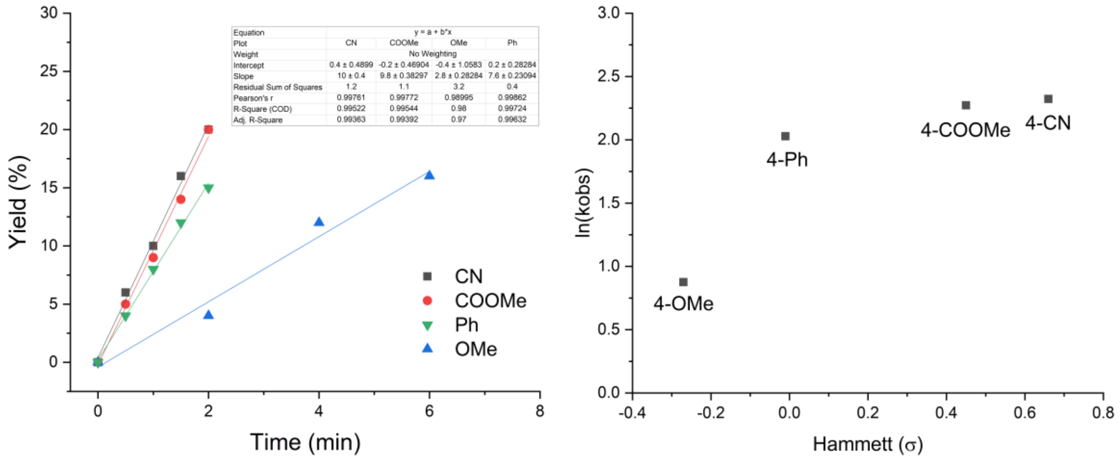

Supplementary Figure 21: **Initial rates with different aryl bromides**.

These results show that the reaction proceeds at similar rates when oxidative additions proceeds smoothly, i.e. with electron-withdrawing substituents on the aromatic ring. This is in accordance with a photon-limited regime, where the rate of the reaction solely depends on the amounts of photon absorbed. On the contrary, when electron-donating substituents are used, the reaction slows down, meaning the rate of the reaction is now limited by one of the steps of the nickel-catalytic cycle, most likely oxidative addition as reported in the case of traditional B-alkyl SM coupling.[8]

2.1.4 Quantum Yield measurement

Determination of light intensity at 456 nm: The procedure reported by Yoon et al. was followed.[9] In particular, a 0.15 M solution of ferrioxalate was prepared by dissolving 2.21 g of potassium ferrioxalate hydrate in 30 mL of 0.05 M H_2SO_4. A buffered solution of phenanthroline was prepared by dissolving 50 mg of phenanthroline and 11.25 g of sodium acetate in 50 mL of 0.5 M H_2SO_4. Both solutions were stored in the dark. To determine the photon flux of the spectrophotometer, 2.0 mL of the ferrioxalate solution were placed in a cuvette and irradiated for the time indicated below at λ = 456 nm with an emission slit width at 10.0 nm. The experiment was performed twice with two different irradiation times (Samples A and B). After irradiation, 0.35 mL of the phenanthroline solution was added to the cuvette. The solution was then allowed to rest for 1 h to allow the ferrous ions to completely coordinate to the phenanthroline. The absorbance of the solution was measured at 510 nm. A non-irradiated sample was also prepared and the absorbance at 510 nm measured.

Consumption of ferrioxalate was < 10%. By using the values for the quantum yield and fraction available for λ = 464 nm: Φ = 0.98 and f = 0.93, an average photon flux of $1.12 \cdot 10^{-8}$ E s^{-1} could be estimated.[10]

Supplementary Table 13: Measurements for the determination of the photon flux

Sample	Irradiation time, s	$[Fe^{2+}]$, M	mmol Fe^{2+}	mol Fe^{2+}	Photon flux (E/s)
**A**	10	$4.58 \cdot 10^{-5}$	$1.08 \cdot 10^{-4}$	$1.08 \cdot 10^{-7}$	$1.16 \cdot 10^{-8}$
**B**	30	$1.27 \cdot 10^{-4}$	$3.00 \cdot 10^{-4}$	$3.00 \cdot 10^{-7}$	$1.07 \cdot 10^{-8}$
				Average	***$1.12 \cdot 10^{-8}$***

Determination of the quantum yield: In a 5 mL volumetric flask, **2a** (0.1 M), $NiCl_2$·glyme (10 mol%), dtbbpy (10 mol%), and 4CzIPN (5 mol%) were dissolved in some CH_3CN. The mixture was sonicated for 10 minutes to obtain a bright green solution; next, 2,6-lutidine (2 equiv.) was added and the solution was diluted to volume with CH_3CN. 2 mL of this solution were transferred to a fluorescence cuvette equipped with a stirring bar and a septum. The solution was bubbled with N_2 (2 min), and then **1a** (1.0 M in hexanes; 1 equiv.) was added with a syringe through the septum. The solution was irradiated for 2 h (7200 s) at λ = 456 nm with an emission slit width at 10.0 nm. The reaction was processed as described above for reaction optimization. Product **3** was obtained in 11% ^{1}H-NMR yield ($2.2 \cdot 10^{-5}$ mol). In 7200s, $8.06 \cdot 10^{-5}$ E were emitted by the light source. Quantum yield was estimated to be 27%.

This value shows that, whether a dark cycle Ni^I/Ni^{III} cycle is operating, it is quite inefficient.

2.1.5 Chemical quenching experiments

Chemical quenching experiments with BHT

In a 7-mL vial equipped with a screw cap **2a** (0.1 mmol), $NiCl_2$·glyme (10 mol%), dtbbpy (10 mol%), and 4CzIPN (5 mol%) were added and dissolved in CH_3CN (1 mL). BHT (0.5 mmol, 5 equiv. 110 mg) was added and the mixture was sonicated for 10 minutes to obtain a bright green solution. The solution was bubbled with N_2 (1 min); hence, **1a** (0.1 mL of a 1.0 M solution in THF, 1 equiv.) was added. The mixture was irradiated for the indicated time with a 40 W Kessil lamp (λ = 456 nm, full intensity) for the required time in the UFO reactor (see **Supplementary Figure 2**). After irradiation, the solvent was removed, the crude was suspended in EtOAc and filtered through a short silica plug in a Pasteur pipette to remove solids. The solvent was removed and the sample was analyzed via ^{1}H-NMR ($CDCl_3$, CH_2Br_2 as external standard).

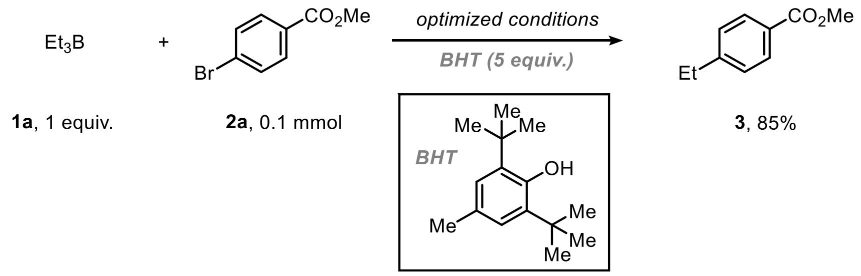

Supplementary Figure 22: **Chemical quenching experiments with BHT**.

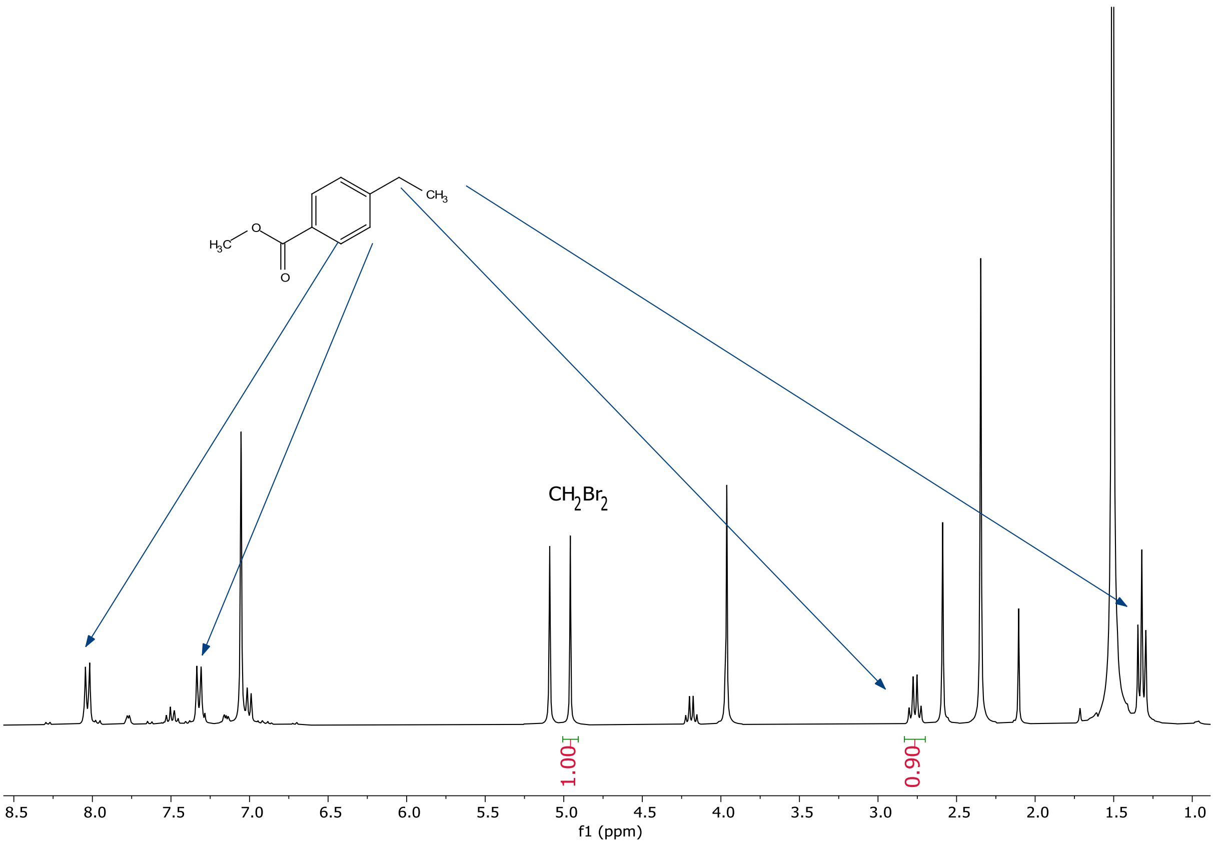

Supplementary Figure 23: **Chemical quenching experiments with BHT.** ^{1}H NMR spectrum of the crude (5 equiv. of BHT, $CDCl_3$, CH_2Br_2 as external standard).

Similarly, other control chemical quenching experiments were performed (see **Supplementary Figure 24**).

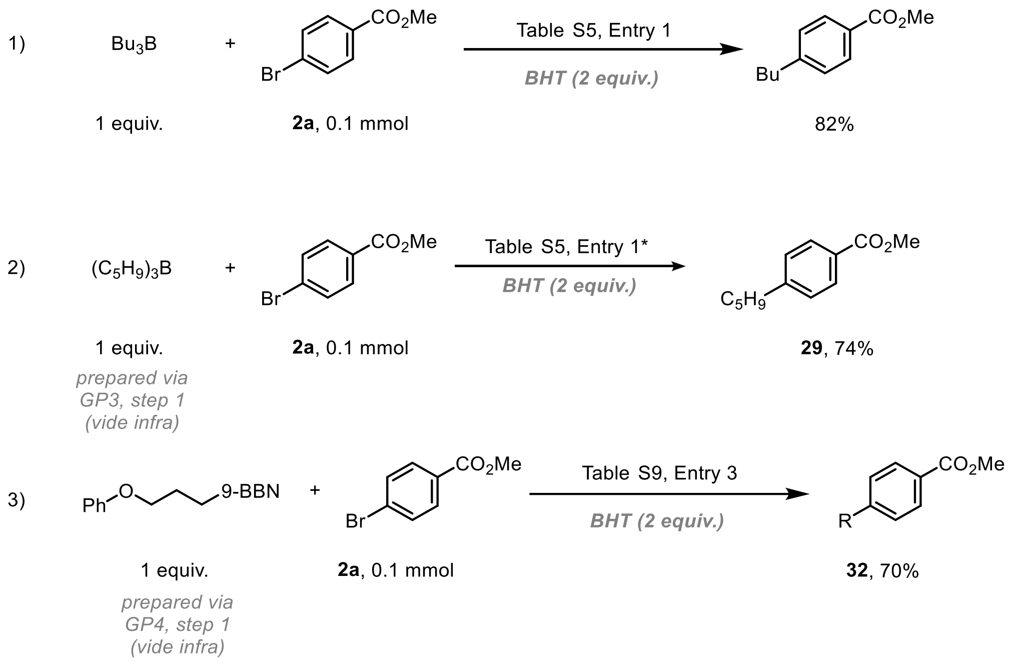

Supplementary Figure 24: Quenching experiments. *solvent: THF

In no case did the BHT inhibit the reaction.

Chemical quenching experiments with TEMPO

In a 7-mL vial equipped with a screw cap **2a** (0.1 mmol), $NiCl_2$·glyme (10 mol%), dtbbpy (10 mol%), and 4CzIPN (5 mol%) were added and dissolved in CH_3CN (1 mL). TEMPO (2 or 1 equiv.) was added. The solution was bubbled with N_2 (1 min); hence, **1a** (0.1 mL of a 1.0 M solution in THF, 1 equiv.) was added. The mixture was irradiated for 3 hours with a 40 W Kessil lamp (λ = 456 nm, full intensity) for the required time in the UFO reactor (see **Supplementary Figure 2**). After irradiation, the solvent was removed, the crude was suspended in EtOAc and filtered through a short silica plug in a Pasteur pipette to remove solids. The solvent was removed and the sample was analyzed via ^{1}H-NMR ($CDCl_3$, CH_2Br_2 as external standard). HRMS (ESI) m/z calcd for $C_{11}H_{24}NO^+$: $[M]^+$ 186.1858; found: 186.1861.

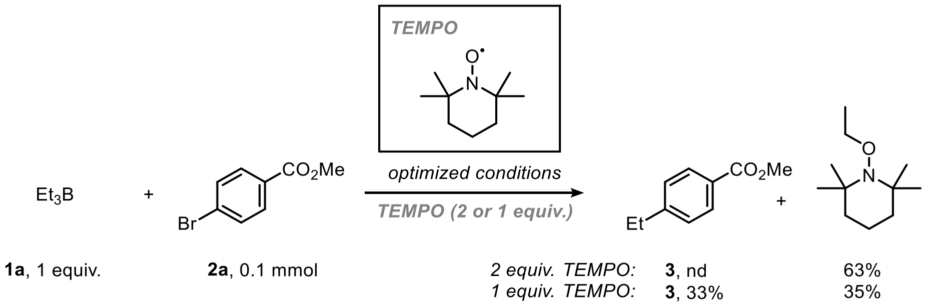

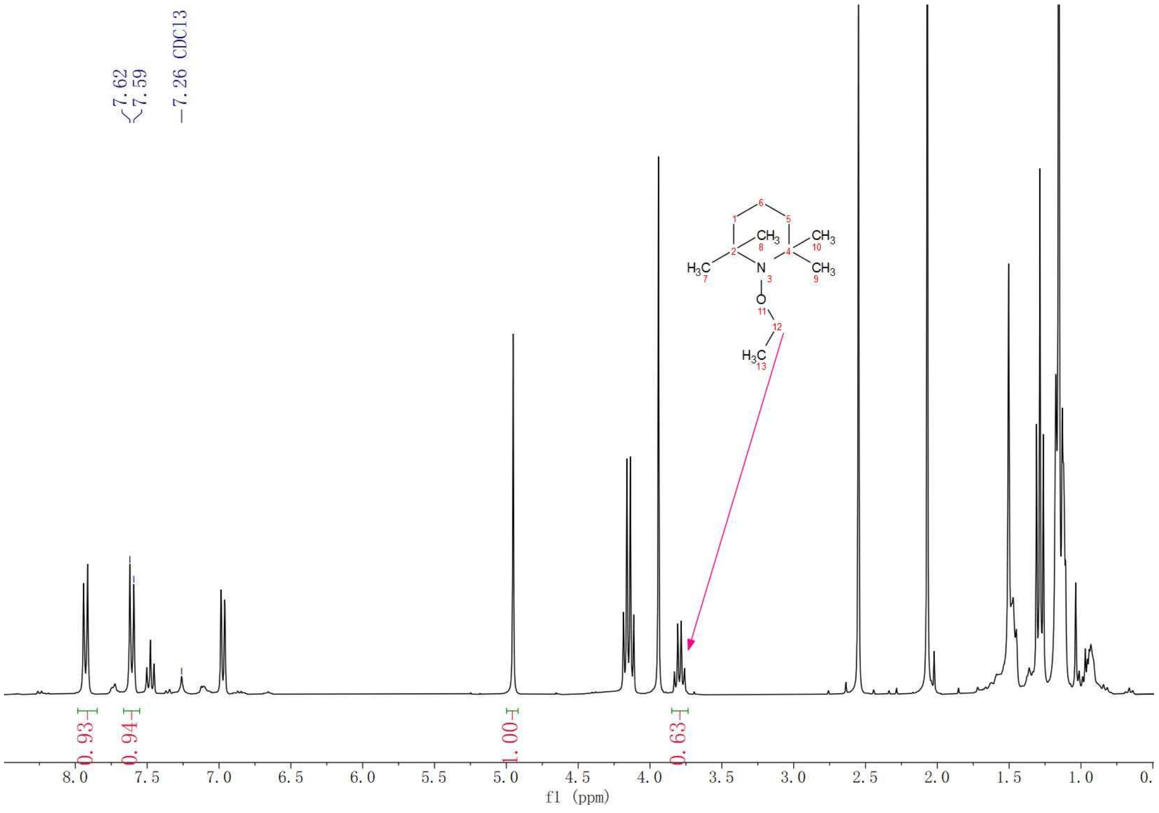

Supplementary Figure 25: Chemical quenching experiments with TEMPO. The ^{1}H NMR spectrum for quenching experiments, (2 equiv. of TEMPO, $CDCl_3$, CH_2Br_2 as external standard).

In a 7-mL vial equipped with a screw cap **2a** (0.1 mmol), $NiCl_2$·glyme (10 mol%), dtbbpy (10 mol%), and 4CzIPN (5 mol%) were added and dissolved in CH_3CN (1 mL). TEMPO (2 equiv.) was added. The solution was bubbled with N_2 (1 min); hence, **1a** (0.1 mL of a 1.0 M solution in THF, 1 equiv.) was added. The mixture was wrapped with foil and reacting for 3 hour. After reaction, the solvent was removed, the crude was suspended in EtOAc and filtered through a short silica plug in a Pasteur pipette to remove solids. The solvent was removed and the sample was analyzed via ^{1}H-NMR ($CDCl_3$). There was no TEMPO-Et in crude NMR and GC-MS.

2.1.6 Electron Paramagnetic Resonance experiments

To gain further insight into the mechanism we employed continuous wave (CW) X-band EPR at low temperature (10 K). As glassing solvent is required for high quality spectra, we first performed catalysis in a mixture of butyronitrile and acetonitrile 5:1 which provided a ^{1}H NMR yield similar to the optimized conditions (75 %). Having confirmed the similar operation of the reaction in this solvent system, we measured the EPR spectrum prior to irradiation which provided no signal at all. Thereafter, inside a J-Young EPR tube, the catalytic mixture was irradiated with 456 nm light (Kessil PR160L, 100% intensity) for 30 seconds and then frozen in liquid nitrogen while irradiating. The tube was transferred to the EPR which provided the following spectrum (**Supplementary Figure 26**, black trace). Simulation of the spectrum to two doublet components provided an isotropic organic radical with g_{iso}=2.002 and a nearly axial metal-centered radical with g_{xyz}=[2.265 2.23 2.047].[11] No resolved hyperfine coupling was observed, but the linewidth (8 mT peak-to-peak) indicated the presence of unresolved coupling, therefore the isotropic signal was assigned to the radical anion of 4CzIPN.[12] The *g*-values for the axial signal closely match those found for other paramagnetic Ni species in literature.[13-16]

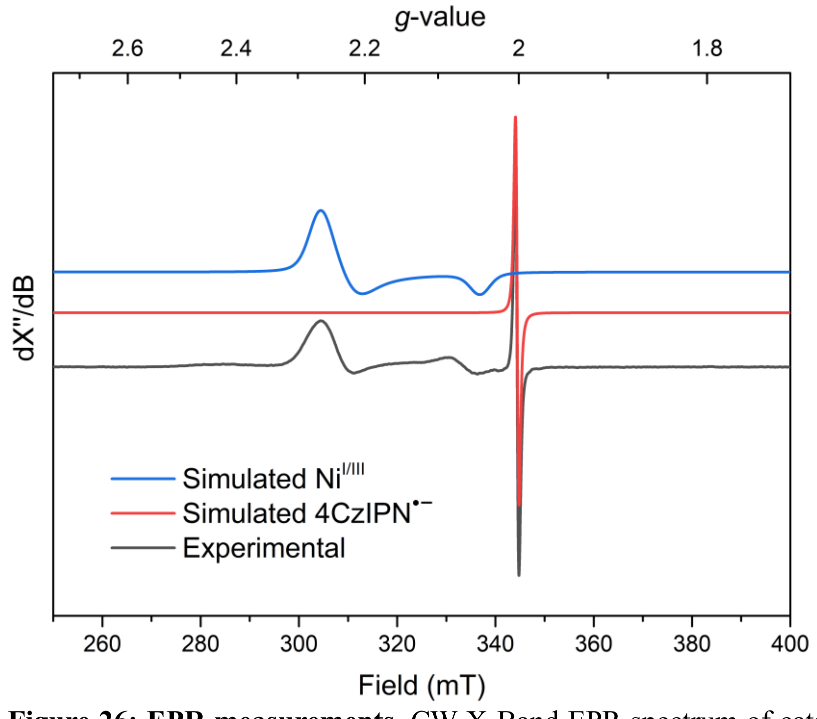

Supplementary Figure 26: EPR measurements. CW X-Band EPR spectrum of catalytic mixture in 5:1 PrCN:CH_3CN at 10 K, after 30 s irradiation with blue light (Mw Freq= 9.6503 GHz). Experimental spectrum (black), simulated spectrum for a paramagnetic Ni species (blue, S=1/2, g=[2.265 2.23 2.047]), simulated spectrum 4CzIPN radical (red, S=1/2, g_{iso}=2.002).

2.1.7 Experiments with the Nickel complex

Note: Dry solvents were either purchased by commercial suppliers or dried via standard procedures and subsequently degassed *via* the freeze-pump-thaw method.

6.7.1 Synthesis of aryl Ni^{II} complex

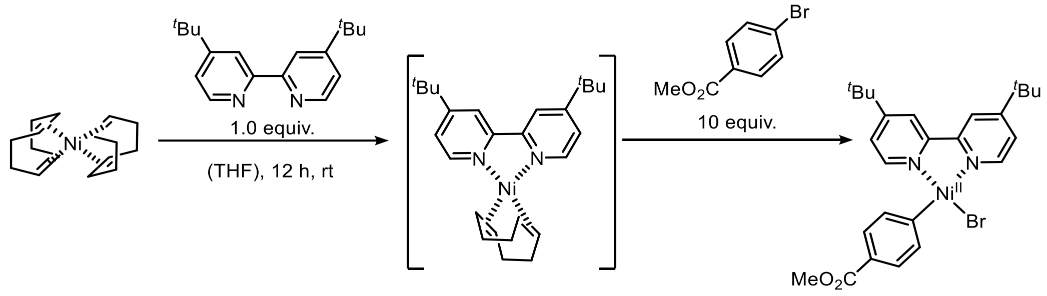

The title complex was prepared as described in the literature.[17]

6.7.2 Stoichiometric experiments with Ni^{II} complex

A flame-dried 5 mL Schlenk-flask equipped with a PTFE coated stirring bar was charged with the aryl Ni^{II} complex (27 mg, 50 µmol, 1.0 equiv.) under N_2 counterflow and cycled three times. The complex was dissolved in 0.5 mL CH_3CN, afterwards 2,6-lutidine (12 µL, 100 µmol, 2.0 equiv.) and Et_3B (1.0 M in hexane, 50 µL, 100 µmol, 1.0 equiv.) were added. The Schlenk-flask was wrapped in aluminum foil and the reaction was stirred for the indicated time. The solvent was removed *in vacuo*, the crude was suspended in EtOAc and filtered through a short silica plug in a Pasteur pipette to remove solids. The solvent was removed *in vacuo* and the sample was analyzed via ^{1}H-NMR ($CDCl_3$, CH_2Br_2 as external standard).

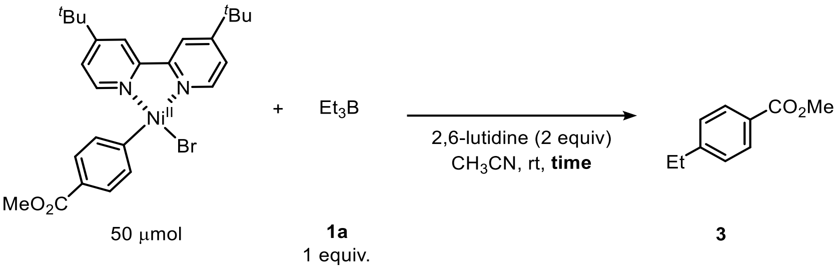

Time (h)	^{1}H- NMR Yield (%)
0.5	19
1.5	29
3	42

6.7.3 *Catalytic experiments with Ni^{II} complex*

A flame-dried 5 mL Schlenk-flask equipped with a PTFE coated stirring bar was charged with the aryl Ni^{II} complex (2.7 mg, 5 µmol, 10 mol%) and **2a** (0.05 mmol) under N_2 counterflow and cycled three times. Under N_2, 0.5 mL CH_3CN were added in the Schlenk-flask. Afterwards 2,6-lutidine (12 µL, 100 µmol, 2.0 equiv.) and Et_3B (1.0 M in hexane, 50 µL, 100 µmol, 1.0 equiv.) were added. The Schlenk-flask was wrapped in aluminum foil and the reaction was stirred for the indicated time. The solvent was removed *in vacuo*, the crude was suspended in EtOAc and filtered through a short silica plug in a Pasteur pipette to remove solids. The solvent was removed *in vacuo* and the sample was analyzed via ^{1}H-NMR ($CDCl_3$, CH_2Br_2 as external standard).

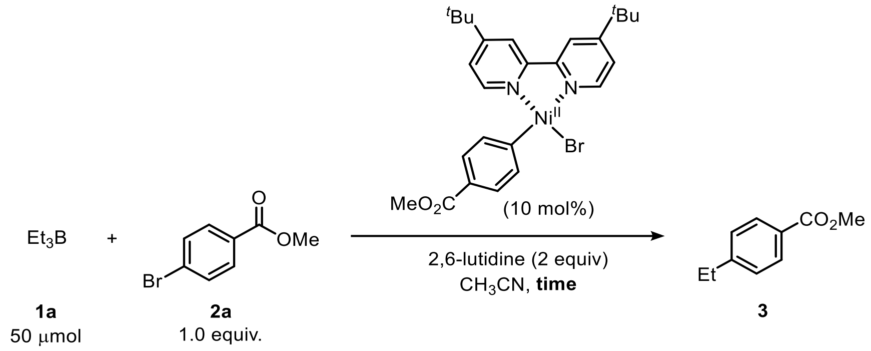

Time (h)	^{1}H- NMR Yield (%)
0.5	2
1.5	4
3	4

2.1.8 Experiments with Ni(COD)$_2$

Three control experiments were performed in order to understand the role of Ni^0 species in the reaction.

1. A flame-dried vial was taken to a glovebox where Ni(COD)$_2$ (10 mol%, 3 mg) and dtbbpy (10 mol%) was weighted. The vial was removed from the glovebox and 0.5 mL dry CH_3CN were added and the resulting mixture was kept stirring for 15 min at room temperature. In the meantime, in a flame-dried vial equipped with a septum, a solution of **2a** (0.1 mmol) and 2,6-lutidine (2 equiv.) was prepared in 0.5 mL dry CH_3CN. Next, the solution was added into the vial with Ni complex. **1a** (0.1 mL of a 1.0 M solution in hexane, 1 equiv.) was added. The mixture was stirred for 3 hours at room temperature. After reaction, the reaction was handled as described in Section 1.4 and analyzed via ^{1}H-NMR ($CDCl_3$, CH_2Br_2 as external standard).

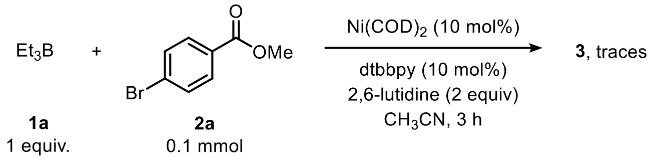

2. A flame-dried vial was taken to a glovebox where Ni(COD)$_2$ (10 mol%, 3 mg) and dtbbpy (10 mol%) was weighted. The vial was removed from the glovebox and 0.5 mL dry CH_3CN were added and the resulting mixture was kept stirring for 15 min at room temperature. In the meantime, in a flame-dried vial equipped with a septum, a solution of **2a** (0.1 mmol) and 2,6-lutidine (2 equiv.) was prepared in 0.5 mL dry CH_3CN. Next, the solution was added into the vial with Ni complex. **1a** (0.1 mL of a 1.0 M solution in hexane, 1 equiv.) was added. The mixture was irradiated for 3 hours with a Kessil lamp (456 nm, full intensity) in a UFO reactor. After reaction, the reaction was handled as described in Section 1.4 and analyzed via ^{1}H-NMR ($CDCl_3$, CH_2Br_2 as external standard).

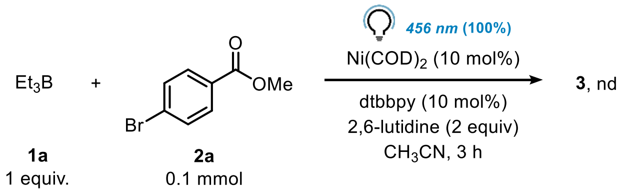

3. A flame-dried vial was taken to a glovebox where Ni(COD)$_2$ (10 mol%, 3 mg) and dtbbpy (10 mol%) was weighted. The vial was removed from the glovebox and 0.5 mL dry CH_3CN were added and the resulting mixture was kept stirring for 15 min at room

temperature. In the meantime, in a flame-dried vial equipped with a septum, a solution of **2a** (0.1 mmol), 2,6-lutidine (2 equiv.) and, 4CzIPN (5 mol%), was prepared in 0.5 mL dry CH_3CN. Next, the solution was added into the vial with Ni complex. **1a** (0.1 mL of a 1.0 M solution in hexane, 1 equiv.) was added. The mixture was irradiated for 3 hours with a Kessil lamp (456 nm, full intensity) in a UFO reactor. After reaction, the reaction was handles as described in Section 1.4 and analyzed via ^{1}H-NMR ($CDCl_3$, CH_2Br_2 as external standard).

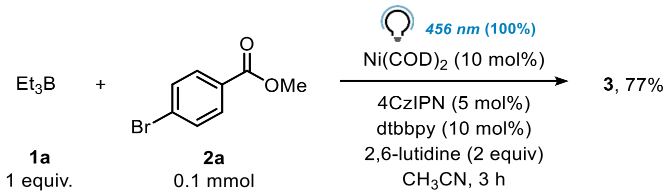

2.1.9 Ligand effect

Step 1: Hydroboration. 1.1 mL of BH_3 solution in THF (1 M, 1.1 mmol) was added to a flame-dried Schlenk flask equipped with a stirring bar at 0 °C (ice bath). Olefin **1n** (3.0 mmol) was added neat dropwise. After addition, the ice bath was removed and the reaction was stirred at room temperature for 1 hour. The reaction was monitored via ^{1}H-NMR without any purification.

Step 2: Photoreaction solution. A CH_3CN (0.1 M) solution containing the aryl bromide **2** (0.5 mmol), $NiCl_2$·glyme and **L1** (4,4′-di-*tert*-butyl-2,2′-dipyridyl) or **L2** (6,6′-dimethyl-2,2′-dipyridyl (10 mol%), **PC3** (5 mol%) and 2,6-Lutidine (2 equiv.) was prepared in a 7 mL vial equipped with a screw cap and a stirring bar. The solution sonicated until fully dissolved and then was bubbled with N_2 (5 min). Alkyl borane (in THF) **1** (1 equiv.) was added. Then the solution was irradiated by adopting the setup shown in **Supplementary Figure 2** equipped with a 40 W Kessil lamp (λ = 456 nm, full intensity) for 3 h (with **L1**) or 16 h (with **L2**). The solutions were collected, solvent was removed under reduced pressure and the crude was purified via column chromatography on silica gel to provide the expected product.

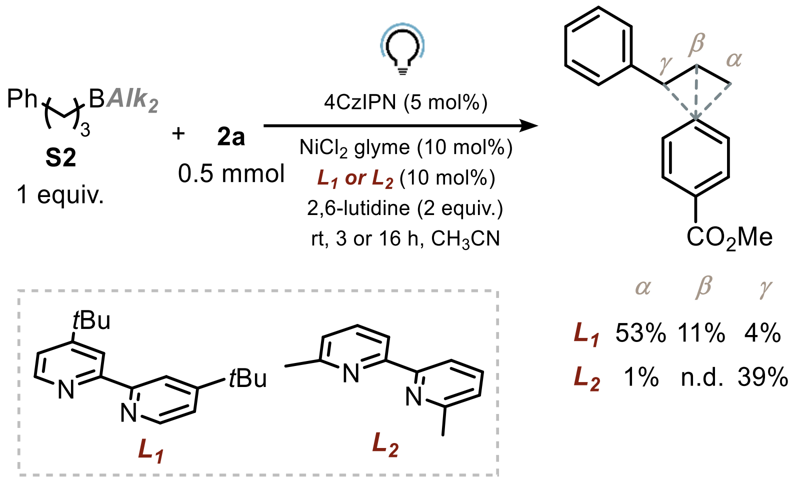

Supplementary Figure 27: **Effect of the nickel ligand on linear-branched selectivity**.

2.1.10 Complexation studies

In order to check for the complexation of trialkyl boranes by 2,6-lutidine, we decided to monitor the chemical shift of the heterocycle upon addition of Et_3B. As a control, we repeated the same experiment with other Lewis bases such as pyridine and 4-dimethylaminopyridine.

Thus, in a flame-dried NMR tube and under inert atmosphere, two solutions were prepared:

1. 2,6-lutidine (12 µL, 0.1 mmol) was dissolved in CD_3CN (500 µL);
2. 2,6-lutidine (12 µL, 0.1 mmol) and Et_3B (1.0 M solution in hexanes; 100 µL, 0.1 mmol) was dissolved in CD_3CN (400 µL).
3. pyridine (8 µL, 0.1 mmol) was dissolved in CD_3CN (500 µL);
4. pyridine (8 µL, 0.1 mmol) and Et_3B (1.0 M solution in hexanes; 100 µL, 0.1 mmol) was dissolved in CD_3CN (400 µL).
5. DMAP (12 mg, 0.1 mmol) was dissolved in CD_3CN (500 µL);
6. DMAP (12 mg, 0.1 mmol) and Et_3B (1.0 M solution in hexanes; 100 µL, 0.1 mmol) was dissolved in CD_3CN (400 µL).

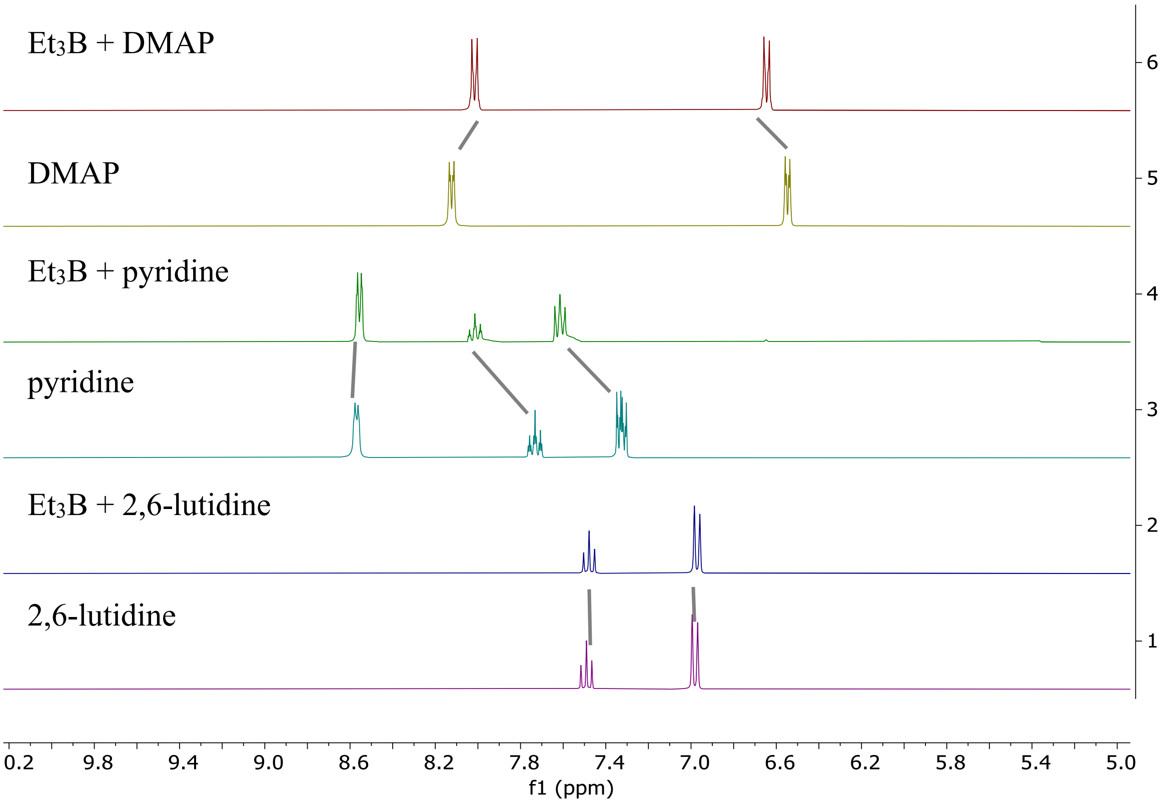

Supplementary Figure 28: **Complexation studies via ^{1}H NMR**.

Although the addition of Et_3B did not alter the chemical shifts of 2,6-lutidine, it caused considerable changes in the chemical shifts of pyridine and DMAP. This suggests that a quick formation of a Lewis acid-base pair occurred in the latter two cases, which is not the case for 2,6-lutidine.

We also monitored the complexation via ^{11}B-NMR, which confirmed that borane complexation occurred only with pyridine and DMAP, but not with 2,6-lutidine.

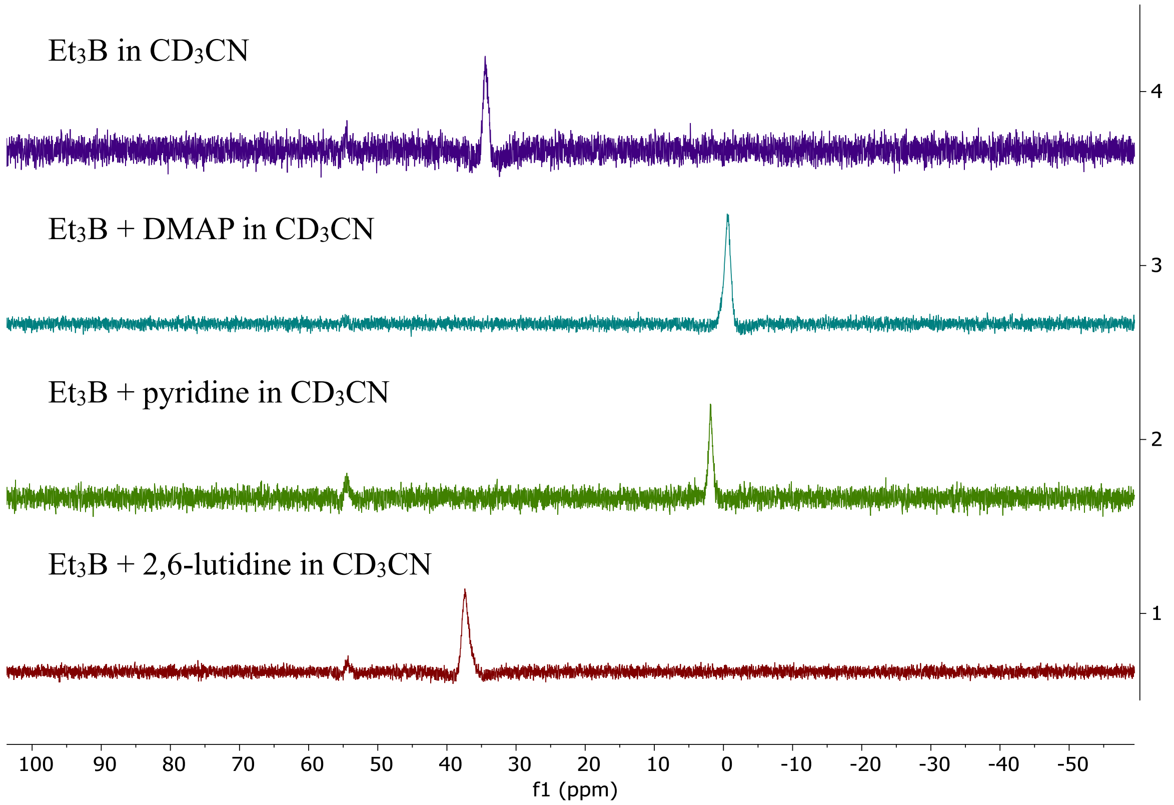

Supplementary Figure 29: **Complexation studies via ^{11}B NMR**.

2.1.11 Decomposition of the PC

4CzIPN was stable upon irradiation under optimized conditions; in fact, we managed to re-isolate it quantitively after column chromatography. However, in one of our control experiments, we noticed that when we omitted the nickel catalyst, fast decomposition occurred. We decided to get insights into this phenomenon by using UV-Vis spectroscopy.

All spectra displayed below were recorded in CH_3CN (solvent cutoff: 190 nm) in quartz cuvettes (optical path: 1 cm) with the Duetta instrument (see General Information). The monochromators slits were set as 5 nm and the spectra were recorded with steps of 1 nm with an integration time for the CCD camera of 0.1 s. Solutions were prepared and filtered with Teflon filters (0.45 µm) before analysis. Ni(dtbbpy)Cl_2 solutions were prepared by dissolving $NiCl_2$·glyme and dtbbpy (1:1) in CH_3CN in a volumetric flask and sonicating until homogeneous (~15 min).

First, an absorption spectra of 4CzIPN (11.4 µM in CH_3CN) and Ni(dtbbpy)Cl_2 (10 mM in CH_3CN) alone were taken, then an absorption spectrum of a N_2-bubbled (10 min) mixture of 4CzIPN (5 mM) and Ni(dtbbpy)Cl_2 (10 mM) in CH_3CN was recorded. The latter spectrum showed additive absorbances (that is the sum of the absorbance of the single components) and no hints of ground state interactions between the two species. By adopting the setup shown in **Supplementary Figure 6** equipped with a Kessil 456 nm (full intensity), the latter solution was irradiated for 1 minute: no changes in the absorption spectrum were observed. Control experiments showed that the photocatalyst is stable upon irradiation with all the other components in the reaction, except for **1a**.

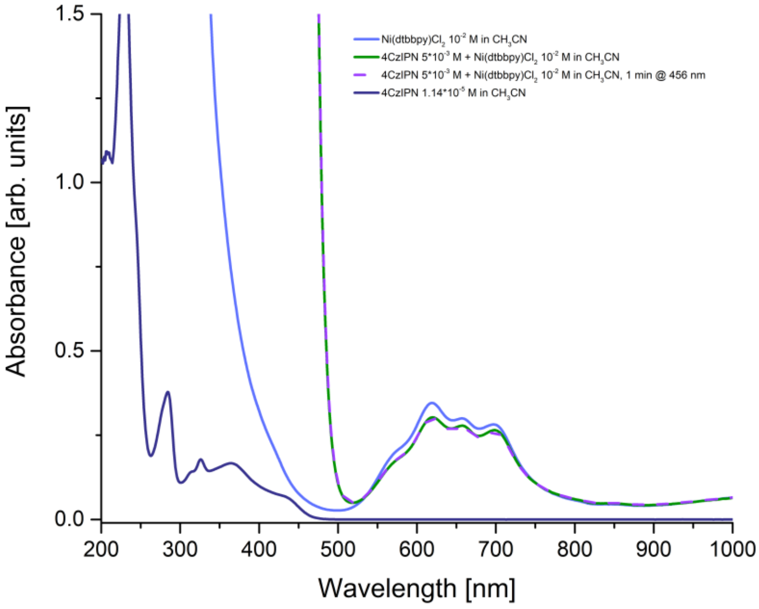

Supplementary Figure 30: UV-Vis analysis of the mixture of 4CzIPN and the nickel complex before and after irradiation.

In fact, when a mixture of 4CzIPN and **1a** (5 mM and 100 mM, respectively, i.e. operating conditions) was irradiated with the setup shown in **Supplementary Figure 6** equipped with a Kessil 456 nm (full intensity), fast decomposition of the photocatalyst occurred.

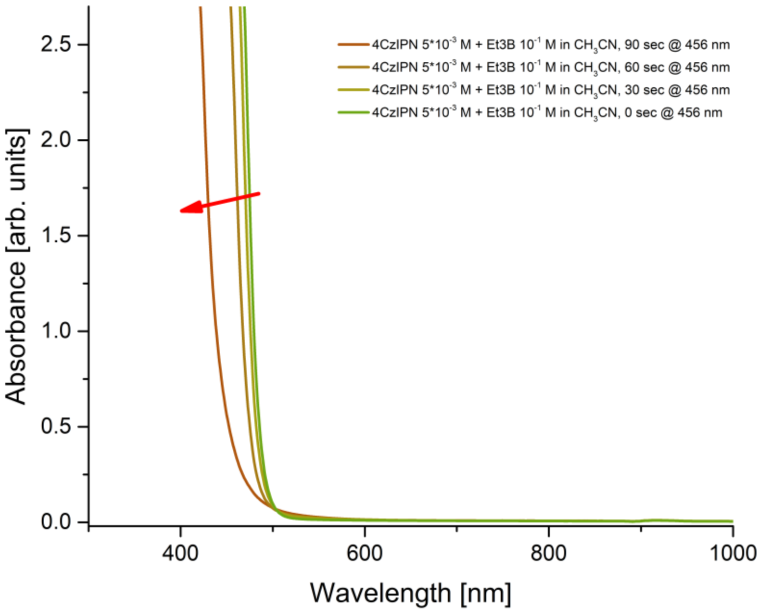

Supplementary Figure 31: UV-Vis analysis of a mixture of 4CzIPN and Et_3B before and after irradiation ([4CzIPN] = $5 \cdot 10^{-3}$ M, Et_3B: 20 equiv.).

Intrigued by this observation, an experiment under diluted conditions was performed to better appreciate the features of the bands. Thus, when a mixture of 4CzIPN and **1a** (100 µM and 2 mM, respectively) in CH_3CN was irradiated under similar conditions for 2 minutes, the bleaching of the photocatalyst was observed. No change of the chromophore was observed before irradiation.

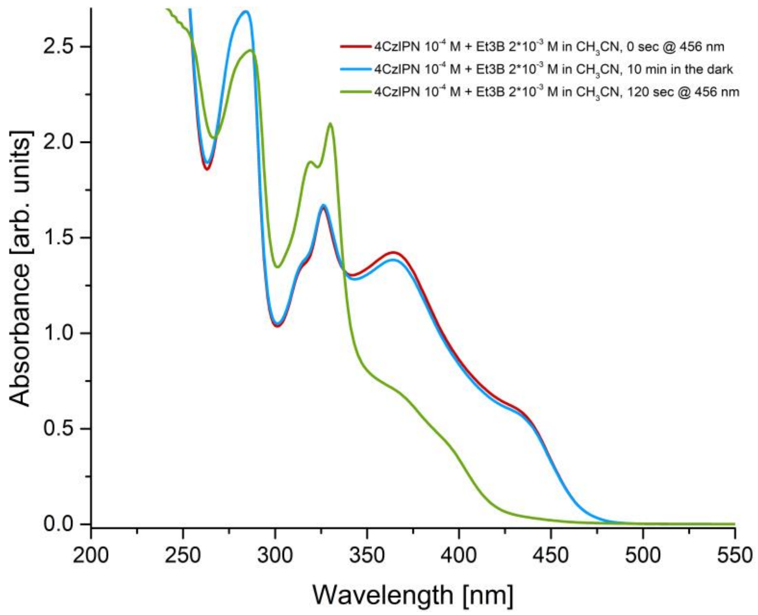

Supplementary Figure 32: UV-Vis analysis of a mixture of 4CzIPN and Et_3B before and after irradiation (dilute conditions: [4CzIPN] = 10^{-4} M, Et_3B: 20 equiv.).

HRMS analysis revealed that decyanative ethylation of the photocatalyst occurred upon irradiation. A similar photochemical transformation of the photocatalyst core was reported by Konig and co-workers and was explained via the formation of alkyl radicals upon reductive quenching of the very same photocatalyst.[18] This study demonstrates that 4CzIPN can produce alkyl radicals from alkyl boranes. Along this line, we monitored the fate of the photocatalyst in our model reaction conditions: the photocatalyst is quantitatively recovered after the reaction, indicating that the decomposition pathway described above is not present or occurs at a lesser extent. This can be explained by a very effective trapping of putative radicals by the nickel complex on the way to product **3** formation.

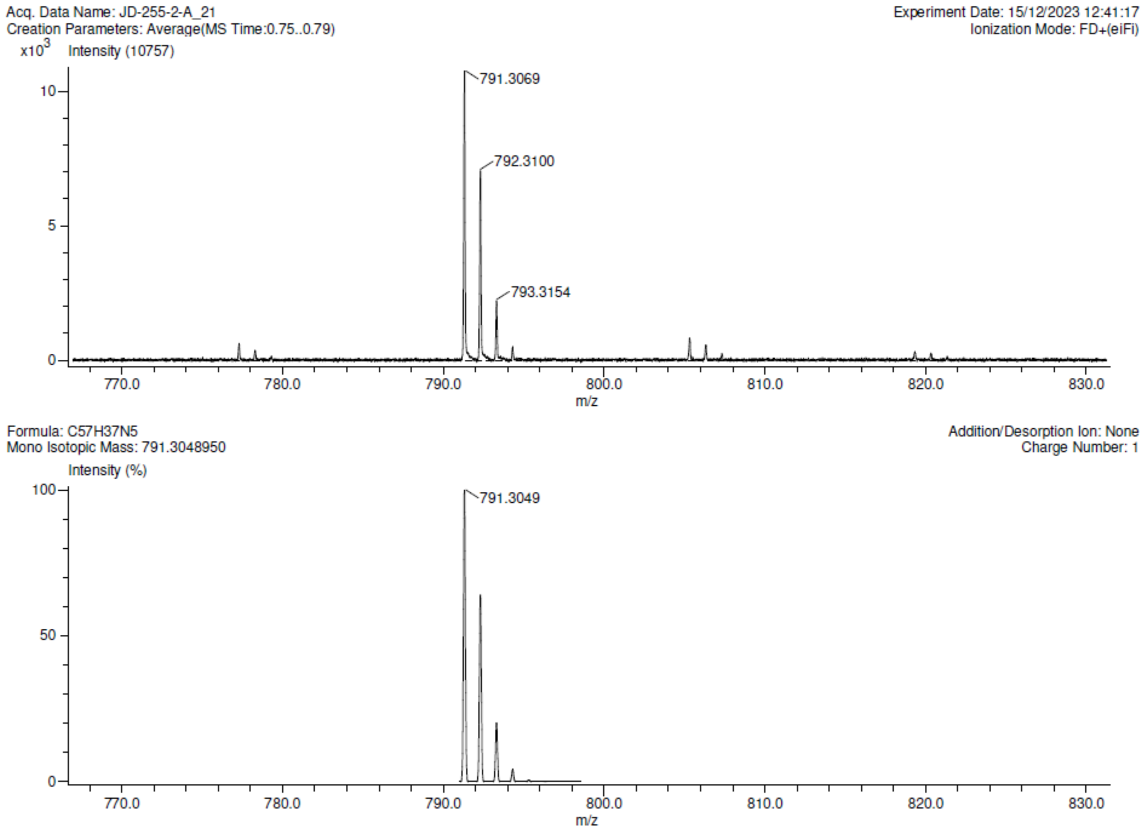

Supplementary Figure 33: HRMS analysis showing the occurrence of decyanative ethylation upon irradiation of a mixture of 4CzIPN and Et_3B.

2.1.12 Redox potential of PCs

In order to better understand the nature of interaction between **1a** and 4CzIPN, we performed the model reaction under optimized conditions with different photocatalyst owing different E(PC*/PC_{red}) and triplet energies to identify possible correlations. Results are summarized in the chart below and displayed in the graph below.

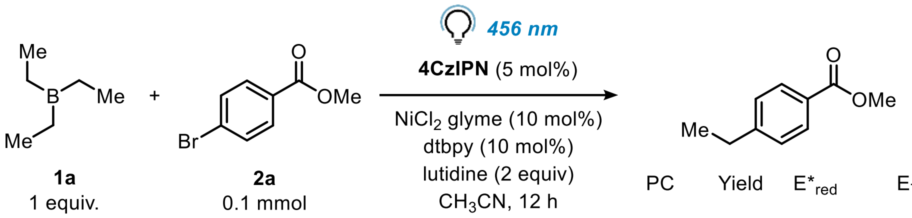

PC	Yield	E^*_{red}	E_T
**4CzIPN**	93%	1.35 V	60 kcal/mol
**MesAcr**	23%	2.06 V	45 kcal/mol
**Ir(df-CF$_3$)**	82%	1.21 V	62 kcal/mol
**Ir(ppy)$_3$**	21%	0.13 V	58 kcal/mol
**Ir(4,4'CF$_3$)**	13%	0.74 V	39 kcal/mol
**thioxanthone**	58%	1.18 V	65 kcal/mol

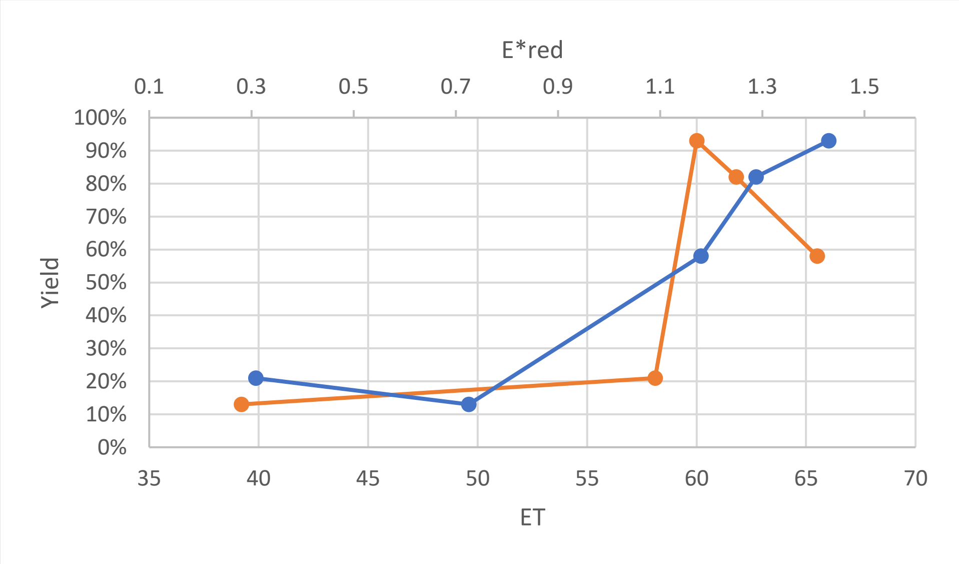

Supplementary Figure 34: Results for the model reaction obtained with different photocatalysts: correlation with triplet energy and redox potentials.

We found that a correlation can be observed with both parameters. Consequently, at present, we refrain from asserting confidence in categorizing the mechanism as either photoinduced electron transfer or triplet-triplet energy transfer. Our leading hypothesis is that the excited state of the photocatalyst is reductively quenched by triethyl borane to deliver alkyl radicals. Intriguingly, precedents of outer-sphere oxidation of alkylboranes to unveil alkyl radicals were proposed (Cu^{2+} as the oxidant[19]). This lends credibility to the proposed hypothesis and ongoing investigations in our laboratories appear to align with this premise. Subsequent findings will be disseminated in due course.

2.1.13 Emission quenching experiments with 2,6-lutidine.

To further prove that the photocatalyst is not quenched by 2,6-lutidine, we demonstrated that the luminescence of the photocatalyst was not quenched even in the presence of large excess of 2,6-lutidine. Thus, 2 mL of a $1.14 \cdot 10^{-5}$ M solution of 4CzIPN in CH_3CN was put in a quartz cuvette (1 cm) and N_2-bubbled for 10 minutes; the cuvette was sealed with a rubber septum. Then, an emission spectrum was taken with a Duetta spectrophotometer (ex@365 nm, bandwidth: 5 nm, integration time: 0.1s). Next, an excess of 2,6-lutidine was added (5 and 10 μL) and the emission spectrum was taken again. No changes in the emission intensity were observed (**Supplementary Figure 35**), which suggests that the base does not quench the photocatalyst.

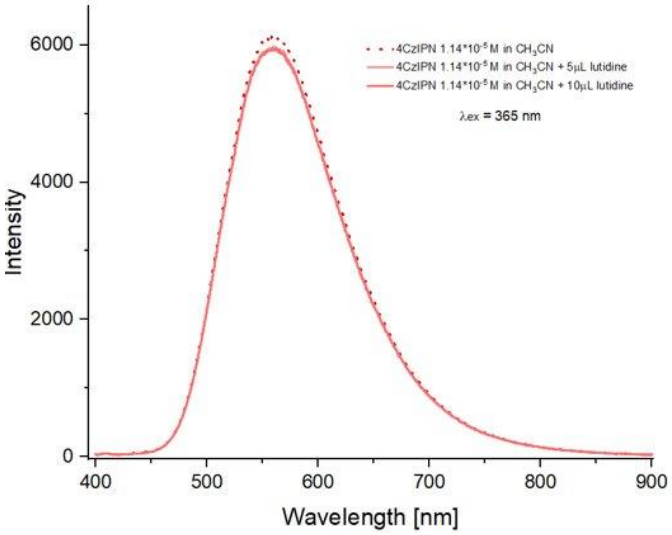

Supplementary Figure 35: Luminescence quenching studies with 2,6-lutidine. Experimental conditions: [4CzIPN]: $1.14 \cdot 10^{-5}$ M in CH_3CN (2 mL). Spectra reported: addition of 0, 5 and 10 uL of 2,6-lutidine. Solutions were degassed for 10 minutes before experiments. Path: 1 cm (2 mL of PC solution), ex@365 nm, bandwidth: 5 nm, int: 0.1s.

2.1.14 Supplementary discussion on the mechanistic scenario.

In the main text, we have proposed a scenario where an alkyl radical is generated by the photocatalyst via reductive quenching: this intermediate is very rapidly quenched by a low-valent Ni^0 species to generate a Ni^I intermediate. Ensuing oxidative addition, reductive elimination and reduction of the last-formed Ni^I species yields the product, thus closing both catalytic cycle.

An alternative scenario where the Ni^0 species first undergoes oxidative addition to form an Ar-Ni^{II}-Br complex can also be conceived; however, this hypothesis is in contrast with the experiments shown in Figure 4C. In detail, while transmetalation and reductive elimination smoothly proceed in the dark in a stoichiometric experiments (Figure 4C, experiment i), the

nickel catalytic cycle is not closed by an oxidative addition step (Figure 4C, experiment ii). Thus, our results indicate that the photocatalyst is needed to promote turnover of the nickel-catalytic cycle, as we proposed in the main text.

Another possible scenario is one where Ni^0 (**A**) undergoes oxidative addition onto aryl bromide to give an Ar-Ni^{II}-Br species (**B**). The latter species is involved in a base-aided transmetalation, just like in traditional SMC reactions, to give the desired Ar-Ni^{II}-Alk complex (**C**). This complex slowly undergoes reductive elimination to restore the Ni^0 species. This step could be promoted under photocatalytic conditions: the photocatalyst could promote an oxidatively induced reductive elimination from **D**[20] to yield the desired alkyl arene and a Ni^I species **E**[14,21,22]. The latter species is then reduced by the spent photocatalyst. It is important to note that this scenario, although intriguing, does not account for the formation of radicals observed in the control experiments with TEMPO and those showcasing decomposition of the photocatalyst. Moreover, experiments ii and iii shown in Figure 4C do not corroborate oxidative addition by Ni^0.

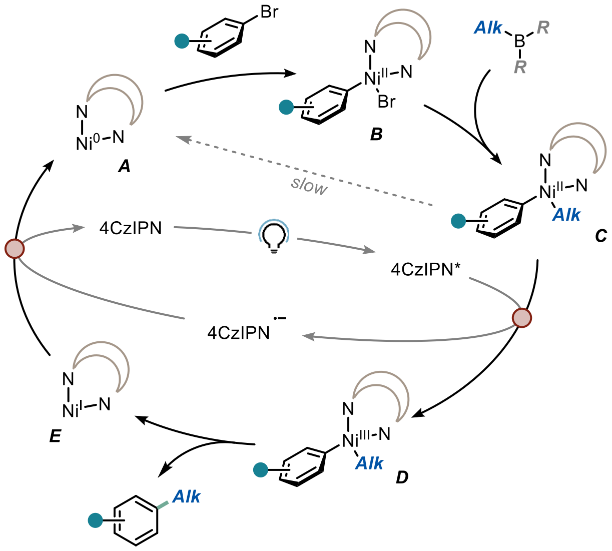

Supplementary Figure 36: Alternative mechanistic scenario.

2.3 Characterization data

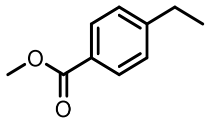

Methyl 4-ethylbenzoate (3). Prepared according to GP2. Purified by flash column chromatography on silica gel (Pentane: Dichloromethane 90:10 → 80:20) to afford the product as colourless oil (64 mg, 78%). ^{1}H NMR (300 MHz, $CDCl_3$) δ 8.04 – 7.93 (m, 2H), 7.33 – 7.22 (m, 2H), 3.92 (s, 3H), 2.72 (q, J = 8 Hz, 2H), 1.27 (t, J = 8 Hz, 3H). ^{13}C NMR (75 MHz, $CDCl_3$) δ 167.3, 149.8, 129.8 (2C), 128.0 (2C), 127.7, 52.0, 29.0, 15.3. Spectroscopic data are in accordance with the literature.[5]

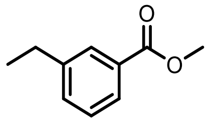

Methyl 3-ethylbenzoate (4). Prepared according to GP2. Purified by flash column chromatography on silica gel (Pentane: Dichloromethane 90:10 → 80:20) to afford the product as colourless oil (58 mg, 70%). ^{1}H NMR (400 MHz, $CDCl_3$) δ 7.89 (s, 1H), 7.87 – 7.82 (m, 1H), 7.41 – 7.32 (m, 2H), 3.91 (s, 3H), 2.70 (q, J = 8 Hz, 2H), 1.26 (t, J = 8 Hz, 3H). ^{13}C NMR (101 MHz, $CDCl_3$) δ 167.5, 144.6, 132.7, 130.3, 129.1, 128.5, 127.1, 52.2, 28.8, 15.6. HRMS (EI) m/z calcd for $C_{10}H_{12}O_2^+$: $[M]^+$ 164.0837; found: 164.0846.

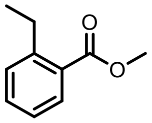

Methyl 2-ethylbenzoate (5). Prepared according to GP2. Purified by flash column chromatography on silica gel (Pentane: Ethyl Acetate 95:5) to afford the product as colourless oil (33 mg, 40%). ^{1}H NMR (300 MHz, $CDCl_3$) δ 7.88 (dd, J_1 = 8 Hz, J_2 = 2 Hz, 1H), 7.45 (td, J_1 = 8, J_2 = 2 Hz, 1H), 7.33 – 7.28 (m, 1H), 7.25 (dd, J_1 = 8, J_2 = 1 Hz, 1H), 3.92 (s, 3H), 3.01 (q, J = 8 Hz, 2H), 1.27 (t, J = 8 Hz, 3H). ^{13}C NMR (75 MHz, $CDCl_3$) δ 168.3, 146.1, 132.1, 130.6, 130.3, 129.5, 125.8, 52.0, 27.7, 16.0. Spectroscopic data are in accordance with the literature.[22]

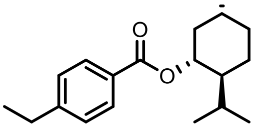

(1R,2S,5R)-2-isopropyl-5-methylcyclohexyl 4-ethylbenzoate (6). Prepared according to GP2. Purified by flash column chromatography on silica gel (Pentane: Ethyl Acetate 95:5) to

afford the product as colourless oil (120 mg, 83%). ^{1}H NMR (400 MHz, $CDCl_3$) δ 7.92 – 7.85 (m, 2H), 7.17 (d, *J* = 8 Hz, 2H), 4.84 (td, *J* = 11, 4 Hz, 1H), 2.61 (q, *J* = 8 Hz, 2H), 2.09 – 1.99 (m, 1H), 1.88 (pd, *J* = 7, 3 Hz, 1H), 1.68 – 1.60 (m, 2H), 1.53 – 1.40 (m, 2H), 1.16 (t, *J* = 8 Hz, 3H), 1.08 – 0.95 (m, 2H), 0.88 – 0.78 (m, 7H), 0.71 (d, *J* = 7 Hz, 3H). ^{13}C NMR (101 MHz, $CDCl_3$) δ 166.3, 149.6, 129.8 (2C), 128.5, 127.9 (2C), 74.7, 47.4, 41.1, 34.5, 31.6, 29.1, 26.6, 23.8, 22.2, 20.9, 16.7, 15.4. HRMS (FD) m/z calcd for $C_{19}H_{28}O_2^+$: $[M]^+$ 288.2089; found: 288.2091.

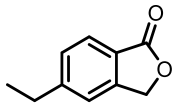

5-Ethylisobenzofuran-1(3H)-one (7). Prepared according to GP2. Purified by flash column chromatography on silica gel (Pentane: Ethyl Acetate 95:5 → 70:30) to afford the product as colourless oil (56 mg, 69%). ^{1}H NMR (300 MHz, $CDCl_3$) δ 7.77 (d, *J* = 8 Hz, 1H), 7.36 – 7.27 (m, 2H), 5.25 (s, 2H), 2.76 (q, *J* = 8 Hz, 2H), 1.26 (t, *J* = 7.6 Hz, 3H). ^{13}C NMR (75 MHz, $CDCl_3$) δ 171.2, 151.5, 147.2, 129.1, 125.4, 123.2, 121.2, 69.5, 29.3, 15.3. HRMS (FI) m/z calcd for $C_{10}H_{10}O_2^+$: $[M]^+$ 162.0681; found: 162.0674.

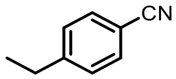

4-Ethylbenzonitrile (8). Prepared according to GP2. Purified by flash column chromatography on silica gel (Pentane: Ethyl Acetate 95:5) to afford the product as colourless oil (51 mg, 78%). ^{1}H NMR (300 MHz, $CDCl_3$) δ 7.60 – 7.54 (m, 2H), 7.34 – 7.26 (m, 2H), 2.72 (q, *J* = 8 Hz, 2H), 1.26 (t, *J* = 8 Hz, 3H). ^{13}C NMR (75 MHz, $CDCl_3$) δ 149.9, 132.2 (2C), 128.7 (2C), 119.2, 109.5, 29.1, 15.1. Spectroscopic data are in accordance with the literature.[23]

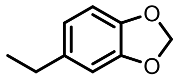

5-Ethylbenzo[d][1,3]dioxole (9). Prepared according to GP2. Purified by flash column chromatography on silica gel (100% Pentane) to afford the product as colourless oil (30 mg, 40%). ^{1}H NMR (300 MHz, $CDCl_3$) δ 6.78 – 6.69 (m, 2H), 6.68 – 6.62 (m, 1H), 5.92 (s, 2H), 2.58 (q, *J* = 8 Hz, 2H), 1.21 (t, *J* = 8 Hz, 3H). ^{13}C NMR (75 MHz, $CDCl_3$) δ 147.6, 145.5, 138.4, 120.5, 108.6, 108.2, 100.8, 28.8, 16.1. HRMS (FI) m/z calcd for $C_9H_{10}O_2^+$: $[M]^+$ 150.0681; found: 150.0686.

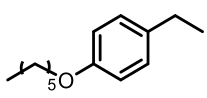

1-Ethyl-4-(hexyloxy)benzene (10). Prepared according to GP2. Purified by flash column chromatography on silica gel (100% Pentane) to afford the product as colourless oil (44 mg,

42%). ^{1}H NMR (400 MHz, $CDCl_3$) δ 7.13 (d, J = 8 Hz, 2H), 6.89 – 6.82 (m, 2H), 3.96 (t, J = 7 Hz, 2H), 2.62 (q, J = 8 Hz, 2H), 1.85 – 1.75 (m, 2H), 1.48 (tt, J_1 = 7 Hz, J_2 = 4 Hz, 2H), 1.43 – 1.33 (m, 4H), 1.25 (t, J = 8 Hz, 3H), 0.99 – 0.90 (m, 3H). ^{13}C NMR (101 MHz, $CDCl_3$) δ 157.3, 136.3, 128.8 (2C), 114.5 (2C), 68.2, 31.8, 29.5, 28.1, 25.9, 22.8, 16.0, 14.2. Spectroscopic data are in accordance with the literature.[24]

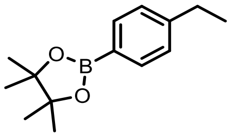

2-(4-Ethylphenyl)-4,4,5,5-tetramethyl-1,3,2-dioxaborolane (11). Prepared according to GP2. Purified by flash column chromatography on silica gel (Pentane: Ethyl Acetate 95:5) to afford the product as colourless oil (75 mg, 65%). ^{1}H NMR (300 MHz, $CDCl_3$) δ 7.85 – 7.73 (m, 2H), 7.31 – 7.22 (m, 2H), 2.71 (q, J = 8 Hz, 2H), 1.38 (s, 12H), 1.31 – 1.27 (m, 3H). ^{13}C NMR (75 MHz, $CDCl_3$) δ 147.8, 135.0 (2C), 127.5 (2C), 83.7 (2C), 29.2, 25.0 (4C), 15.59. The signal of the α-B-carbon was not observed. ^{11}B NMR (96 MHz, $CDCl_3$) δ 31.2. Spectroscopic data are in accordance with the literature.[25]

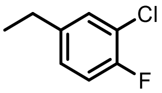

2-Chloro-4-ethyl-1-fluorobenzene (12). Prepared according to GP2, extracted with diethyl ether and removed solvent carefully (product is Volatile). CH_2Br_2 (87.5 mg, 35 uL, 1 equiv.) was added, NMR yield: 88%. Purified by flash column chromatography on silica gel (100% Pentane) to afford the product mixture with starting material as colourless oil. ^{1}H NMR (300 MHz, $CDCl_3$) δ 7.24 – 7.18 (m, 1H), 7.09 – 6.99 (m, 2H), 2.61 (q, J = 7.6 Hz, 2H), 1.22 (t, J = 7.6 Hz, 3H). ^{13}C NMR (75 MHz, $CDCl_3$) δ 156.5 (d, J = 246 Hz), 141.2 (d, J = 4 Hz), 129.9 (2C), 127.5 (d, J = 7 Hz), 116.4 (d, J = 21 Hz), 28.1, 15.6 (d, J = 1 Hz). ^{19}F NMR (282 MHz, $CDCl_3$) δ -120.52. HRMS (FI) m/z calcd for $C_8H_8ClF^+$: $[M]^+$ 158.0299; found: 158.0305.

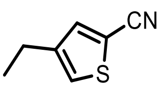

4-Ethylthiophene-2-carbonitrile (13). Prepared according to GP2. Purified by flash column chromatography on silica gel (Pentane: Dichloromethane 91:9) to afford the product as colourless oil (45 mg, 65%). ^{1}H NMR (300 MHz, $CDCl_3$) δ 7.47 – 7.43 (m, 1H), 7.22 – 7.17 (m, 1H), 2.66 (q, J = 8 Hz, 2H), 1.25 (t, J = 8 Hz, 3H). ^{13}C NMR (75 MHz, $CDCl_3$) δ 145.6, 138.1, 127.1, 114.7, 109.6, 23.3, 14.6. HRMS (FI) m/z calcd for $C_7H_7NS^+$: $[M]^+$ 137.0299; found: 137.0295.

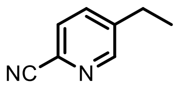

5-Ethylpicolinonitrile (14). Prepared according to GP2. Purified by flash column chromatography on silica gel (Pentane: Ethyl Acetate 80:20) to afford the product as light yellow oil (32 mg, 48%). ^{1}H NMR (300 MHz, $CDCl_3$) δ 8.55 (t, J = 1 Hz, 1H), 7.69 – 7.56 (m, 2H), 2.73 (q, J = 8 Hz, 2H), 1.28 (t, J = 8 Hz, 3H). ^{13}C NMR (75 MHz, $CDCl_3$) δ 151.2, 143.7, 136.1, 131.3, 128.3, 117.6, 26.4, 14.8. HRMS (FI) m/z calcd for $C_8H_8N_2^+$: [M]$^+$ 132.0687; found: 132.0681.

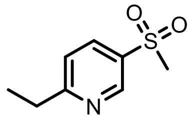

2-Ethyl-5-(methylsulfonyl)pyridine (15). Prepared according to GP2. Purified by flash column chromatography on silica gel (Pentane: Ethyl Acetate 75:25 → 50:50) to afford the product as light yellow oil (57 mg, 61%). ^{1}H NMR (300 MHz, $CDCl_3$) δ 8.99 (dd, J_1 = 3, J_2 = 1Hz, 1H), 8.08 (dd, J_1 = 8, J_2 = 2 Hz, 1H), 7.33 (d, J = 8 Hz, 1H), 3.06 (s, 3H), 2.89 (q, J = 8 Hz, 2H), 1.30 (t, J = 8 Hz, 3H). ^{13}C NMR (75 MHz, $CDCl_3$) δ 169.5, 148.2, 135.6, 134.3, 122.4, 45.0, 31.6, 13.5. HRMS (FI) m/z calcd for $C_8H_{11}NO_2S^+$: [M]$^+$ 185.0510; found: 185.0503.

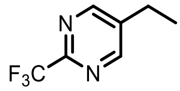

5-Ethyl-2-(trifluoromethyl)pyrimidine (16). Prepared according to GP2. Purified by flash column chromatography on silica gel (Pentane: Ethyl Acetate 91:9) to afford the product as colourless oil (61 mg, 69%). ^{1}H NMR (300 MHz, $CDCl_3$) δ 8.71 (s, 2H), 2.74 (q, J = 8 Hz, 2H), 1.31 (t, J = 8 Hz, 3H). ^{13}C NMR (75 MHz, $CDCl_3$) δ 157.3 (2C), 154.8 (q, J = 37 Hz), 139.2, 119.82 (q, J = 275 Hz), 23.6, 14.6. ^{19}F NMR (282 MHz, $CDCl_3$) δ -70.2. HRMS (FI) m/z calcd for $C_7H_7F_3N_2^+$: [M]$^+$ 176.0561; found: 176.0569.

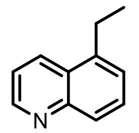

5-Ethylquinoline (17). Prepared according to GP2. Purified by flash column chromatography on silica gel (Pentane : Dichloromethane 50:50 → Pentane : Ethyl Acetate 90:10 → 70:30) to afford the product as deliquescent solid (55 mg, 70%). ^{1}H NMR (400 MHz, $CDCl_3$) δ 8.89 (dd, J_1 = 4 Hz, J_2 = 2 Hz, 1H), 8.35 (dd, J_1 = 9 Hz, J_2 = 2 Hz, 1H), 7.96 (d, J = 9 Hz, 1H), 7.62 (dd, J_1 = 9 Hz, J_2 = 7 Hz, 1H), 7.42 – 7.33 (m, 2H), 3.07 (q, J = 8 Hz, 2H), 1.35 (t, J = 8 Hz, 3H).

^{13}C NMR (101 MHz, $CDCl_3$) δ 149.9, 148.8, 140.7, 132.1, 129.3, 127.8, 126.9, 125.4, 120.7, 25.3, 15.2. Spectroscopic data are in accordance with the literature.[26]

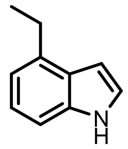

4-Ethyl-1H-indole (18). Prepared according to GP2. Purified by flash column chromatography on silica gel (Pentane: Ethyl Acetate 95:5) to afford the product as colourless oil (25 mg, 34%). ^{1}H NMR (300 MHz, $CDCl_3$) δ 8.06 (s, 1H), 7.35 – 7.16 (m, 3H), 7.09 – 7.02 (m, 1H), 6.73 – 6.63 (m, 1H), 3.05 (q, *J* = 8 Hz, 2H), 1.46 (t, *J* = 8 Hz, 3H). ^{13}C NMR (75 MHz, $CDCl_3$) δ 136.7, 135.8, 127.1, 123.6, 122.3, 118.2, 108.9, 101.0, 26.5, 14.9. HRMS (FI) m/z calcd for $C_{10}H_{11}N^+$: $[M]^+$ 145.0891; found: 145.0899.

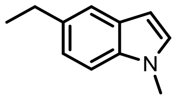

5-Ethyl-1-methyl-1H-indole (19). Prepared according to GP2, CH_3OH as solvent. Purified by flash column chromatography on silica gel (Pentane: Ethyl Acetate 98:2 → 95:5) to afford the product as yellow oil (45 mg, 62%). ^{1}H NMR (300 MHz, $CDCl_3$) δ 7.60 – 7.56 (m, 1H), 7.39 – 7.33 (m, 1H), 7.22 (dd, J_1 = 8 Hz, J_2 = 2 Hz, 1H), 7.13 – 7.09 (m, 1H), 6.55 (dd, J_1 = 3 Hz, J_2 = 1 Hz, 1H), 3.85 (s, 3H), 2.89 (q, *J* = 8 Hz, 2H), 1.43 (t, *J* = 8 Hz, 3H). ^{13}C NMR (75 MHz, $CDCl_3$) δ 135.4, 135.3, 128.9, 128.8, 122.2, 119.4, 109.1, 100.5, 32.9, 29.1, 16.7. HRMS (FI) m/z calcd for $C_{11}H_{13}N^+$: $[M]^+$ 159.1048; found: 159.1056.

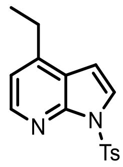

4-Ethyl-1-tosyl-1H-pyrrolo[2,3-b]pyridine (20). Prepared according to GP2. Purified by flash column chromatography on silica gel (Pentane: Ethyl Acetate 95:5 → 91:9) to afford the product as light yellow solid (130 mg, 87%), m.p. 95.8~101.2 °C. ^{1}H NMR (300 MHz, $CDCl_3$) δ 8.31 (d, *J* = 5 Hz, 1H), 8.09 – 7.99 (m, 2H), 7.66 (d, *J* = 4 Hz, 1H), 7.24 – 7.16 (m, 2H), 6.96 (d, *J* = 5 Hz, 1H), 6.60 (d, *J* = 4 Hz, 1H), 2.79 (q, *J* = 8 Hz, 2H), 2.30 (s, 3H), 1.24 (t, *J* = 8 Hz, 3H). ^{13}C NMR (75 MHz, $CDCl_3$) δ 147.2, 146.7, 145.2, 145.1, 135.5, 129.6 (2C), 128.0 (2C), 125.5, 122.1, 117.8, 103.6, 25.6, 21.6, 13.9. HRMS (ESI) m/z calcd for $C_{16}H_{17}N_2O_2S^+$: $[M+H]^+$ 301.1005; found: 301.1006.

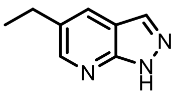

5-Ethyl-1H-pyrazolo[3,4-b]pyridine (21). Prepared according to GP2, THF as solvent. Purified by flash column chromatography on silica gel (Pentane: Ethyl Acetate 80:20 → 50:50) to afford the product as light yellow solid (30 mg, 41%), m.p. 87~90 ºC. ^{1}H NMR (300 MHz, $CDCl_3$) δ 13.41 (s, 1H), 8.53 (d, *J* = 2 Hz, 1H), 8.06 (s, 1H), 7.95 – 7.89 (m, 1H), 2.80 (q, *J* = 8 Hz, 2H), 1.31 (t, *J* = 8 Hz, 3H). ^{13}C NMR (75 MHz, $CDCl_3$) δ 151.0, 149.8, 133.3, 132.7, 128.6, 115.4, 26.2, 16.1. HRMS (FI) m/z calcd for $C_8H_9N_3^+$: $[M]^+$ 147.0796; found: 147.0800.

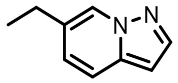

6-Ethylpyrazolo[1,5-a]pyridine (22). Prepared according to GP2. Purified by flash column chromatography on silica gel (Pentane: Ethyl Acetate 90:10) to afford the product as colourless oil (53 mg, 72%). ^{1}H NMR (300 MHz, $CDCl_3$) δ 8.29 – 8.23 (m, 1H), 7.87 (d, *J* = 2 Hz, 1H), 7.42 (dd, J_1 = 9 Hz, J_2 = 1 Hz, 1H), 6.95 (dd, J_1 = 9 Hz, J_2 = 2 Hz, 1H), 6.43 (dd, J_1 = 2 Hz, J_2 = 1 Hz, 1H), 2.62 (qd, J_1 = 8 Hz, J_2 = 1 Hz, 2H), 1.25 (t, *J* = 8 Hz, 3H). ^{13}C NMR (75 MHz, $CDCl_3$) δ 141.3, 138.8, 127.7, 125.8, 125.4, 117.6, 96.3, 25.7, 14.9. HRMS (EI) m/z calcd for $C_9H_{10}N_2^+$: $[M]^+$ 146.0844; found: 146.0847.

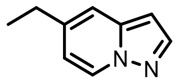

5-Ethylpyrazolo[1,5-a]pyridine (23). Prepared according to GP2. Purified by flash column chromatography on silica gel (Pentane: Ethyl Acetate 90:10) to afford the product as colourless oil (50 mg, 68%). ^{1}H NMR (400 MHz, $CDCl_3$) δ 8.36 (d, *J* = 7 Hz, 1H), 7.90 (d, *J* = 2 Hz, 1H), 7.31 – 7.26 (m, 1H), 6.58 (dd, J_1 = 7 Hz, J_2 = 2 Hz, 1H), 6.39 (d, *J* = 2 Hz, 1H), 2.65 (q, *J* = 8 Hz, 2H), 1.26 (t, *J* = 8 Hz, 3H). ^{13}C NMR (101 MHz, $CDCl_3$) δ 142.0, 140.4, 139.9, 128.0, 115.0, 113.3, 95.8, 28.3, 14.5. HRMS (EI) m/z calcd for $C_9H_{10}N_2^+$: $[M]^+$ 146.0844; found: 146.0846.

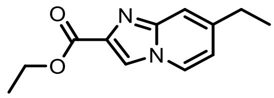

Ethyl 7-ethylimidazo[1,2-a]pyridine-2-carboxylate (24). Prepared according to GP2, CH_3CN/DMSO = 3:2 (5 mL). Purified by flash column chromatography on silica gel (Pentane : Ethyl Acetate 50:50) to afford the product as colourless solid (56 mg, 51%), m.p. 47~52 ºC. ^{1}H NMR (400 MHz, $CDCl_3$) δ 8.03 (d, *J* = 1 Hz, 1H), 7.96 (dd, J_1 = 7 Hz, J_2 = 1 Hz, 1H), 7.33 (dt, J_1 = 2 Hz, J_2 = 1 Hz, 1H), 6.64 (dd, J_1 = 7 Hz, J_2 = 2 Hz, 1H), 4.36 (q, *J* = 7 Hz, 2H), 2.60 (qd, J_1 = 8 Hz, J_2 = 1 Hz, 2H), 1.35 (t, *J* = 7 Hz, 3H), 1.19 (t, *J* = 8 Hz, 3H). ^{13}C NMR (101 MHz, $CDCl_3$) δ 163.4, 145.8, 143.1, 136.7, 125.4, 116.4, 115.6, 115.5, 60.9, 28.4, 14.4, 14.1. HRMS (EI) m/z calcd for $C_{12}H_{14}N_2O_2^+$: $[M]^+$ 218.1055; found: 218.1048.

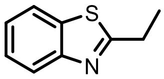

2-Ethylbenzo[d]thiazole (25). Prepared according to GP2. Purified by flash column chromatography on silica gel (Pentane: Ethyl Acetate 85:15) to afford the product as deliquescent solid (42 mg, 51%). ^{1}H NMR (400 MHz, $CDCl_3$) δ 7.97 (d, J = 8 Hz, 1H), 7.84 (d, J = 8 Hz, 1H), 7.47 – 7.41 (m, 1H), 7.34 (td, J_1 = 8 Hz, J_2 = 1 Hz, 1H), 3.15 (q, J = 8 Hz, 2H), 1.47 (t, J = 8 Hz, 3H). ^{13}C NMR (101 MHz, $CDCl_3$) δ 173.7, 153.4, 135.2, 126.0, 124.7, 122.63, 121.6, 27.9, 13.9. Spectroscopic data are in accordance with the literature.[22]

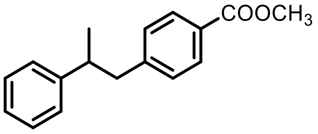

Methyl 4-(2-phenylpropyl)benzoate (26). Prepared according to GP3 (Method A). Purified by flash column chromatography on silica gel (Pentane: Dichloromethane 75:25 → 67:33) to afford the product as light yellow oil (98 mg, 77%). ^{1}H NMR (300 MHz, $CDCl_3$) δ 8.03 – 7.91 (m, 2H), 7.31 (tt, J_1 = 7 Hz, J_2 = 1 Hz, 2H), 7.25 – 7.11 (m, 5H), 3.91 (s, 3H), 3.14 – 2.96 (m, 2H), 2.89 (dd, J_1 = 13 Hz, J_2 = 7 Hz, 1H), 1.31 (d, J = 7 Hz, 3H). ^{13}C NMR (75 MHz, $CDCl_3$) δ 167.1, 146.3, 146.2, 129.5 (2C), 129.2 (2C), 128.4 (2C), 127.9, 127.0 (2C), 126.2, 51.9, 45.0, 41.7, 21.3. Spectroscopic data are in accordance with the literature.[27]

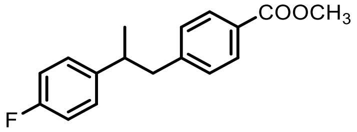

Methyl 4-(2-(4-fluorophenyl)propyl)benzoate (27). Prepared according to GP3 (Method A). Purified by flash column chromatography on silica gel (Pentane : Ethyl Acetate 95:5) to afford the product as colourless oil (71 mg, 52%). ^{1}H NMR (400 MHz, $CDCl_3$) δ 7.94 – 7.87 (m, 2H), 7.12 – 7.03 (m, 4H), 6.98 – 6.89 (m, 2H), 3.89 (s, 3H), 3.02 (h, J = 7 Hz, 1H), 2.93 – 2.81 (m, 2H), 1.26 (d, J = 7 Hz, 3H). ^{13}C NMR (101 MHz, $CDCl_3$) δ 167.2, 161.4 (d, J = 244 Hz), 146.1, 141.9 (d, J = 3 Hz), 129.6 (2C), 129.3 (2C), 128.44 (d, J = 8 Hz, 2C), 128.1, 115.16 (d, J = 21 Hz, 2C), 52.1, 45.3, 41.1, 21.6. ^{19}F NMR (376 MHz, $CDCl_3$) δ -117.1. HRMS (EI) m/z calcd for $C_{17}H_{17}FO_2^+$: $[M]^+$ 272.1213; found: 272.1215.

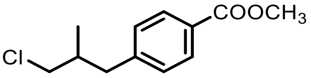

Methyl 4-(3-chloro-2-methylpropyl)benzoate (28). Prepared according to GP3 (Method A). Purified by flash column chromatography on silica gel (Pentane : Ethyl Acetate 99:1 → 95-5) to afford the product as colourless oil (53 mg, 47%). ^{1}H NMR (300 MHz, $CDCl_3$) δ 8.04 – 7.93

(m, 2H), 7.31 – 7.22 (m, 2H), 3.92 (s, 3H), 3.52 – 3.36 (m, 2H), 2.87 (dd, J_1 = 13 Hz, J_2 = 7 Hz, 1H), 2.59 (dd, J_1 = 13 Hz, J_2 = 7 Hz, 1H), 2.17 (qt, J_1 = 7 Hz, J_2 = 5 Hz, 1H), 1.03 (d, J = 7 Hz, 3H). ^{13}C NMR (75 MHz, $CDCl_3$) δ 167.1, 145.5, 129.8 (2C), 129.3 (2C), 128.3, 52.1, 50.2, 40.1, 37.4, 17.7. HRMS (FI) m/z calcd for $C_{12}H_{15}ClO_2^{+}$: $[M]^+$ 226.0761; found: 226.0757.

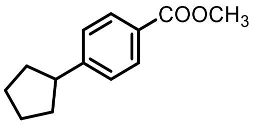

Methyl 4-cyclopentylbenzoate (29). Prepared according to GP3 (Method A), step 2: THF as solvent, 6 h. Purified by flash column chromatography on silica gel (Pentane : Ethyl Acetate 98:2) to afford the product as colourless oil (73 mg, 83%). ^{1}H NMR (300 MHz, $CDCl_3$) δ 7.99 – 7.91 (m, 2H), 7.33 – 7.26 (m, 2H), 3.89 (s, 3H), 3.13 – 2.95 (m, 1H), 2.18 – 1.99 (m, 2H), 1.89 – 1.51 (m, 6H). ^{13}C NMR (75 MHz, $CDCl_3$) δ 167.3, 152.3, 129.7 (2C), 127.7, 127.2 (2C), 52.0, 46.1, 34.6 (2C), 25.7 (2C). Spectroscopic data are in accordance with the literature.[28]

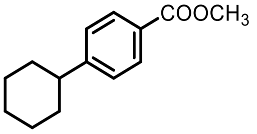

Methyl 4-cyclohexylbenzoate (30). Prepared according to GP3 (Method A), step 1: 13 h, step 2: 6 h. Purified by flash column chromatography on silica gel (Pentane : Ethyl Acetate 98:2) to afford the product as colourless oil (73 mg, 67%). ^{1}H NMR (300 MHz, $CDCl_3$) δ 8.04 – 7.93 (m, 2H), 7.32 – 7.23 (m, 2H), 3.91 (s, 3H), 2.67 – 2.48 (m, 1H), 1.97 – 1.70 (m, 5H), 1.52 – 1.15 (m, 5H). ^{13}C NMR (101 MHz, $CDCl_3$) δ 167.2, 153.5, 129.8 (2C), 127.8, 126.9 (2C), 52.0, 44.8, 34.2 (2C), 26.8 (2C), 26.1. Spectroscopic data are in accordance with the literature.[29]

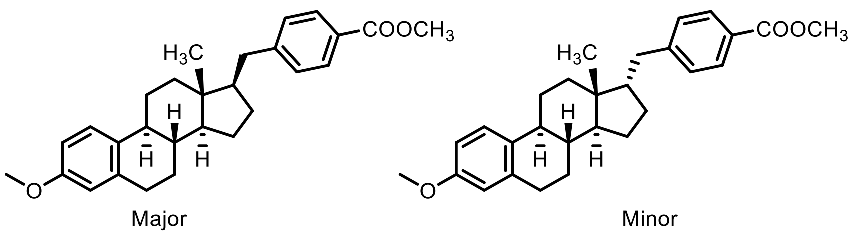

Methyl 4-(((8S,9S,13R,14S,17R)-3-methoxy-13-methyl-7,8,9,11,12,13,14,15,16,17-decahydro-6H-cyclopenta[a]phenanthren-17-yl)methyl)benzoate (31), methyl 4-(((8S,9S,13R,14S,17S)-3-methoxy-13-methyl-7,8,9,11,12,13,14,15,16,17-decahydro-6H-cyclopenta[a]phenanthren-17-yl)methyl)benzoate (31'). Prepared according to GP3 (Method A), 0.25 mmol scale of **2a**, THF as solvent. Purified by flash column chromatography on silica gel (Pentane : Ethyl Acetate 95:5) to afford the product as white solid (45 mg, 43%, dr 3:1, brsm: 73%). ^{1}H NMR (400 MHz, $CDCl_3$) δ 8.01 – 7.95 (m, 2H, mixture), 7.32 – 7.27

(m, 2H, mixture), 7.27 – 7.20 (m, 1H, mixture), 6.78 – 6.71 (m, 1H, mixture), 6.69 – 6.64 (m, 1H, mixture), 3.94 (s, 3H, mixture), 3.82 – 3.78 (m, 3H, mixture), 3.00 – 2.80 (m, 3H, mixture), 2.57 – 2.18 (m, 3H, mixture), 2.14 – 1.67 (m, 5H, mixture), 1.64 – 1.19 (m, 7H, mixture), 0.90 (s, 0.8H, minor), 0.77 (s, 2.2H, major). ^{13}C NMR (101 MHz, $CDCl_3$) δ 167.3, 157.6, 157.5, 148.3, 148.0, 138.2, 133.0, 132.9, 129.7, 129.6, 129.3, 128.9, 127.7, 126.5, 126.4, 113.9, 111.6, 111.5, 55.3, 54.8, 52.7, 52.1, 50.0, 49.1, 44.2, 43.9, 43.8, 42.9, 39.4, 39.0, 38.1, 37.9, 37.0, 34.4, 30.1, 30.0, 28.5, 28.4, 27.9, 27.1, 26.8, 26.6, 25.5, 24.2, 21.1, 12.8. HRMS (EI) m/z calcd for $C_{28}H_{34}O_3^+$: $[M]^+$ 418.2508; found: 418.2494.

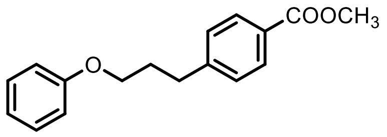

Methyl 4-(3-phenoxypropyl)benzoate (32). Prepared according to GP4 (Method B). Purified by flash column chromatography on silica gel (Pentane: Ethyl Acetate 95:5) to afford the product as white solid (80 mg, 59%), m.p.36~40 °C. ^{1}H NMR (400 MHz, $CDCl_3$) δ 7.89 – 7.83 (m, 2H), 7.22 – 7.12 (m, 4H), 6.84 (tt, J_1 = 7 Hz, J_2 = 1 Hz, 1H), 6.79 (dt, J_1 = 8 Hz, J_2 = 1 Hz, 2H), 3.84 (t, J = 6 Hz, 2H), 3.79 (s, 3H), 2.76 (dd, J_1 = 8 Hz, J_2 = 7 Hz, 2H), 2.06 – 1.95 (m, 2H). ^{13}C NMR (101 MHz, $CDCl_3$) δ 167.1, 159.0, 147.2, 129.8 (2C), 129.5 (2C), 128.6 (2C), 128.0, 120.8, 114.6 (2C), 66.6, 52.0, 32.3, 30.6. HRMS (FI) m/z calcd for $C_{17}H_{18}O_3^+$: $[M]^+$ 270.1256; found: 270.1265.

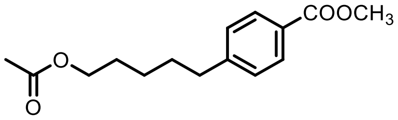

Methyl 4-(5-acetoxypentyl)benzoate (33). Prepared according to GP4 (Method B). Purified by flash column chromatography on silica gel (Pentane: Ethyl Acetate 70:30) to afford the product as colourless liquid (114 mg, 86%). ^{1}H NMR (300 MHz, $CDCl_3$) δ 8.00 – 7.90 (m, 2H), 7.25 – 7.20 (m, 2H), 4.05 (t, J = 7 Hz, 2H), 3.90 (s, 3H), 2.67 (t, J = 8 Hz, 2H), 2.03 (s, 3H), 1.74 – 1.60 (m, 4H), 1.45 – 1.34 (m, 2H). ^{13}C NMR (75 MHz, $CDCl_3$) δ 171.3, 167.3, 148.1, 129.8 (2C), 128.6 (2C), 127.9, 64.5, 52.1, 35.9, 30.8, 28.6, 25.7, 21.1. HRMS (FI) m/z calcd for $C_{15}H_{20}O_4^+$: $[M]^+$ 264.1362; found: 264.1362.

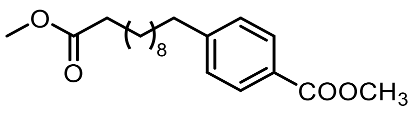

Methyl 4-(11-methoxy-11-oxoundecyl)benzoate (34). Prepared according to GP4 (Method B). Purified by flash column chromatography on silica gel (Pentane : Ethyl Acetate 98:2 → 95:5) to afford the product as colourless oil (120 mg, 72%), m.p. 40~42 °C. ^{1}H NMR (400 MHz,

$CDCl_3$) δ 7.98 – 7.90 (m, 2H), 7.23 (d, J = 8 Hz, 2H), 3.90 (s, 3H), 3.66 (s, 3H), 2.69 – 2.60 (m, 2H), 2.29 (t, J = 8 Hz, 2H), 1.67 – 1.55 (m, 4H), 1.28 (td, J_1 = 8 Hz, J_2 = 4 Hz, 12H). ^{13}C NMR (101 MHz, $CDCl_3$) δ 174.5, 167.4, 148.6, 129.8 (2C), 128.6 (2C), 127.7, 52.1, 51.6, 36.1, 34.2, 31.3, 29.6, 29.5, 29.5, 29.4 (2C), 29.3, 25.1. HRMS (FD) m/z calcd for $C_{20}H_{30}O_4^+$: $[M]^+$ 334.2144; found: 334.2154.

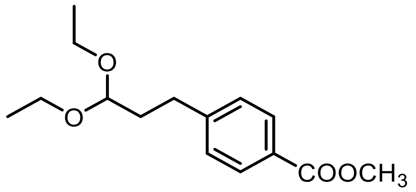

Methyl 4-(3,3-diethoxypropyl)benzoate (35). Prepared according to GP4 (Method B), step 1: 4 h. Purified by flash column chromatography on silica gel (Pentane : Ethyl Acetate 85:15) to afford the product as colourless oil (40 mg, 30%). ^1H NMR (300 MHz, CD_3CN) δ 7.95 – 7.87 (m, 2H), 7.37 – 7.29 (m, 2H), 4.44 (t, J = 6 Hz, 1H), 3.85 (s, 3H), 3.67 – 3.55 (m, 2H), 3.51 – 3.39 (m, 2H), 2.77 – 2.65 (m, 2H), 1.91 – 1.81 (m, 2H), 1.14 (t, J = 7 Hz, 6H). ^{13}C NMR (75 MHz, CD_3CN) δ 167.7, 148.9, 130.4 (2C), 129.6 (2C), 128.9, 103.0, 62.0 (2C), 52.5, 35.9, 31.6, 15.7 (2C). HRMS (EI) m/z calcd for $C_{15}H_{22}O_4^+$: $[M]^+$ 266.1518; found: 266.1520.

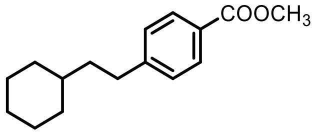

Methyl 4-(2-cyclohexylethyl)benzoate (36). Prepared according to GP4 (Method B). Purified by flash column chromatography on silica gel (Pentane : Ethyl Acetate 99:3) to afford the product as colourless oil (112 mg, 90%). ^1H NMR (300 MHz, $CDCl_3$) δ 7.99 – 7.89 (m, 2H), 7.29 – 7.18 (m, 2H), 3.89 (s, 3H), 2.71 – 2.60 (m, 2H), 1.82 – 1.58 (m, 5H), 1.56 – 1.45 (m, 2H), 1.33 – 1.06 (m, 4H), 1.02 – 0.81 (m, 2H). ^{13}C NMR (75 MHz, $CDCl_3$) δ 167.2, 148.9, 129.7 (2C), 128.4 (2C), 127.6, 52.0, 39.0, 37.4, 33.4, 33.3 (2C), 26.7, 26.4 (2C). HRMS (FI) m/z calcd for $C_{16}H_{22}O_2^+$: $[M]^+$ 246.1620; found: 246.1619.

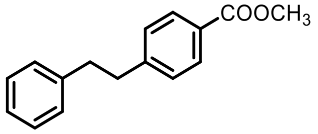

Methyl 4-phenethylbenzoate (37). Prepared according to GP4 (Method B). Purified by flash column chromatography on silica gel (Pentane : Ethyl Acetate 95:5) to afford the product as colourless oil (80 mg, 82%). Product **37** was also synthesized in 86% yield after isolation by adopting GP5 (3.8 mL reactor have been used for step 1, corresponding to 90 minutes residence time). ^1H NMR (300 MHz, $CDCl_3$) δ 8.03 – 7.93 (m, 2H), 7.36 – 7.14 (m, 7H), 3.93 (s, 3H),

3.06 – 2.92 (m, 4H). ^{13}C NMR (75 MHz, $CDCl_3$) δ 167.3, 147.3, 141.3, 129.8 (2C), 128.7 (2C), 128.6 (2C), 128.5 (2C), 128.1, 126.2, 52.1, 38.0, 37.6. Spectroscopic data are in accordance with the literature.[22]

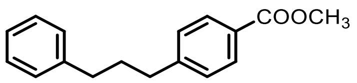

Methyl 4-(3-phenylpropyl)benzoate (38). Prepared according to GP4 (Method B). Purified by flash column chromatography on silica gel (Pentane: Ethyl Acetate 98:2) to afford the product as colourless oil (90 mg, 71%). ^{1}H NMR (300 MHz, $CDCl_3$) δ 8.01 – 7.90 (m, 2H), 7.35 – 7.14 (m, 7H), 3.91 (s, 3H), 2.68 (dt, J_1 = 15 Hz, J_2 = 8 Hz, 4H), 2.06 – 1.90 (m, 2H). ^{13}C NMR (75 MHz, $CDCl_3$) δ 167.3, 148.0, 142.1, 129.8 (2C), 128.6 (2C), 128.6 (2C), 128.5 (2C), 127.9, 126.0, 52.1, 35.6, 35.5, 32.7. Spectroscopic data are in accordance with the literature.[30]

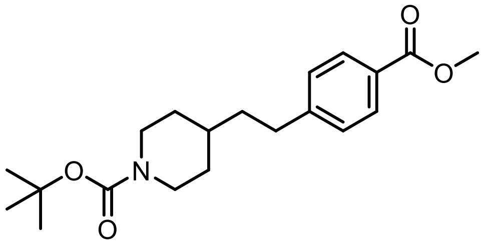

***tert*-Butyl 4-(4-(methoxycarbonyl)phenethyl)piperidine-1-carboxylate (39).** Prepared according to GP4 (Method B). Purified by flash column chromatography on silica gel (Pentane : Ethyl Acetate: 70:30, second column with reverse phase C18, H_2O:CH_3CN (0.1% Formic acid) 50:50 → 20:80) to afford the product as colourless oil (137 mg, 79%). Product **39** was also synthesized in 73% yield after isolation by adopting GP5 (2.5 mL reactor have been used for step 1, corresponding to 60 minutes residence time). ^{1}H NMR (300 MHz, $CDCl_3$) δ 7.96 – 7.86 (m, 2H), 7.24 – 7.14 (m, 2H), 4.14 – 3.97 (m, 2H), 3.85 (s, 3H), 2.74 – 2.51 (m, 4H), 1.66 (dd, J_1 = 14 Hz, J_2 = 3 Hz, 2H), 1.59 – 1.48 (m, 2H), 1.42 (s, 9H), 1.39 – 1.29 (m, 1H), 1.17 – 1.01 (m, 2H). ^{13}C NMR (75 MHz, $CDCl_3$) δ 167.1, 154.8, 148.1, 129.7 (2C), 128.3 (2C), 127.8, 79.2, 52.0, 44.0 (2C), 37.9, 35.5, 33.0, 32.1 (2C), 28.5 (3C). HRMS (FD) m/z calcd for $C_{20}H_{29}NO_4^+$: $[M]^+$ 347.2097; found: 347.2102.

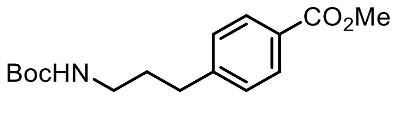

Methyl 4-(3-((*tert*-butoxycarbonyl)amino)propyl)benzoate (40). Prepared according to GP4 (Method B). Purified by flash column chromatography on silica gel (Pentane : Ethyl Acetate: 95:5) to afford the product as colourless oil (105 mg, 72%). ^{1}H NMR (300 MHz, $CDCl_3$) δ 7.99 – 7.91 (m, 2H), 7.26 – 7.21 (m, 2H), 4.55 (s, 1H), 3.90 (s, 3H), 3.15 (q, J= 7, 2H), 2.69 (dd, J_1

= 9 Hz, J_2 = 7 Hz, 2H), 1.88 – 1.78 (m, 2H), 1.44 (s, 9H). ^{13}C NMR (75 MHz, $CDCl_3$) δ 167.2, 156.1, 147.3, 129.9 (2C), 128.5 (2C), 128.1, 79.4, 52.1, 40.3, 33.3, 31.6, 28.5 (3C). Spectroscopic data are in accordance with the literature.[31]

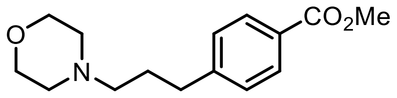

Methyl 4-(3-morpholinopropyl)benzoate (41). Prepared according to GP4 (Method B). Purified by flash column chromatography on silica gel (Dichloromethane : Methanol: 96:4) to afford the product as light yellow oil (66 mg, 50%). ^{1}H NMR (400 MHz, $CDCl_3$) δ = 7.97 – 7.88 (m, 2H), 7.25 – 7.17 (m, 2H), 3.87 (s, 3H), 3.72 – 3.65 (m, 4H), 2.71 – 2.63 (m, 2H), 2.41 (t, J_1 = 4 Hz. J_2 = 7 Hz, 4H), 2.37 – 2.28 (m, 2H), 1.87 – 1.75 (m, 2H). ^{13}C NMR (101 MHz, $CDCl_3$) δ 167.2, 147.6, 129.8 (2C), 128.5 (2C), 127.9, 67.0 (2C), 58.1, 53.7 (2C), 52.0, 33.6, 27.8. HRMS (ESI) m/z calcd for $C_{15}H_{22}NO_3^+$: $[M+H]^+$ 264.1594; found: 264.1601.

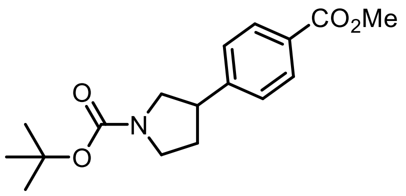

***tert*-Butyl 3-(4-(methoxycarbonyl)phenyl)pyrrolidine-1-carboxylate (42).** Prepared according to GP4 (Method B), step 1: reflux reaction for 1 hour. Purified by flash column chromatography on silica gel (Pentane : Ethyl Acetate: 70:30, second column with reverse phase C18, H_2O:CH_3CN (0.1% Formic acid) 50:50 → 20:80) to afford the product as colourless oil (91 mg, 60%). ^{1}H NMR (400 MHz, $CDCl_3$) δ 7.95 – 7.87 (m, 2H), 7.25 – 7.20 (m, 2H), 3.82 (s, 3H), 3.79 – 3.69 (m, 1H), 3.58 – 3.45 (m, 1H), 3.39 – 3.20 (m, 3H), 2.25 – 2.15 (m, 1H), 1.98 – 1.85 (m, 1H), 1.40 (s, 9H). ^{13}C NMR (101 MHz, $CDCl_3$) δ 166.9, 154.5, 146.9, 130.0 (2C), 128.8, 127.2 (2C), 79.4, 52.1, 52.0, 45.7, 43.9, 32.8, 28.6 (3C). HRMS (ESI) m/z calcd for $C_{17}H_{23}NO_4Na^+$: $[M+Na]^+$ 328.1519; found: 328.1526. Spectroscopic data are in accordance with the literature.[30]

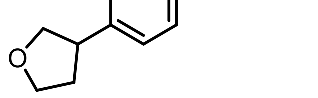

Methyl 4-(tetrahydrofuran-3-yl)benzoate (43). Prepared according to GP4 (Method B). Purified by flash column chromatography on silica gel (Pentane : Ethyl Acetate: 85:15) to afford the product as colourless oil (58 mg, 56%). ^{1}H NMR (300 MHz, $CDCl_3$) δ 8.02 – 7.93 (m, 2H), 7.37 – 7.28 (m, 2H), 4.18 – 4.10 (m, 1H), 4.07 (dt, J_1 = 8 Hz, J_2 = 4 Hz, 1H), 3.95 (t,

J = 8 Hz, 1H), 3.91 (s, 3H), 3.76 (dd, J_1 = 9 Hz, J_2 = 7 Hz, 1H), 3.46 (p, J = 8 Hz, 1H), 2.46 – 2.33 (m, 1H), 2.10 – 1.94 (m, 1H). ^{13}C NMR (75 MHz, $CDCl_3$) δ 167.1, 148.6, 130.1 (2C), 128.6, 127.4 (2C), 74.6, 68.6, 52.2, 45.1, 34.8. Spectroscopic data are in accordance with the literature.[32]

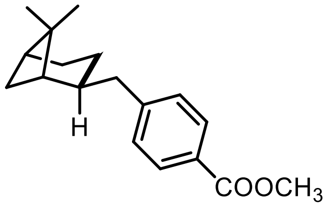

Methyl 4-(((1S,2R,5S)-6,6-dimethylbicyclo[3.1.1]heptan-2-yl)methyl)benzoate (44). Prepared according to GP4 (Method B). Purified by flash column chromatography on silica gel (Pentane : Ethyl Acetate 97:3) to afford the product as colourless oil (129 mg, 90%). Product **40** was also synthesized in 86% yield after isolation by adopting GP5 (2.5 mL reactor have been used for step 1, corresponding to 60 minutes residence time). ^{1}H NMR (400 MHz, $CDCl_3$) δ 7.97 – 7.91 (m, 2H), 7.20 (d, J = 8 Hz, 2H), 3.89 (s, 3H), 2.79 – 2.66 (m, 2H), 2.39 – 2.23 (m, 2H), 2.00 – 1.77 (m, 5H), 1.60 – 1.48 (m, 1H), 1.19 (s, 3H), 1.13 (s, 3H), 0.84 (d, J = 10 Hz, 1H). ^{13}C NMR (101 MHz, $CDCl_3$) δ 167.2, 147.6, 129.6 (2C), 129.1 (2C), 127.7, 52.0, 45.3, 43.5, 43.1, 41.5, 38.8, 33.8, 28.2, 26.4, 23.6, 22.3. HRMS (EI) m/z calcd for $C_{18}H_{24}O_2^+$: $[M]^+$ 272.1776; found: 272.1783.

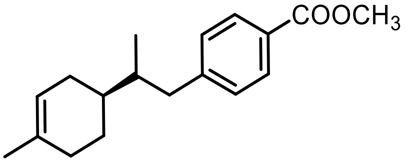

Methyl 4-((R)-2-((S)-4-methylcyclohex-3-en-1-yl)propyl)benzoate (45). Prepared according to GP4 (Method B). Purified by flash column chromatography on silica gel (Pentane : Ethyl Acetate 98:2 → 95:5) to afford the product as colourless oil (114 mg, 84%, dr 1:1). ^{1}H NMR (300 MHz, $CDCl_3$) δ 8.02 – 7.89 (m, 2H), 7.24 – 7.17 (m, 2H), 5.43 – 5.33 (m, 1H), 3.90 (s, 3H), 2.83 (dd, J_1 = 13 Hz, J_2 = 5 Hz, 1H), 2.37 (ddd, J_1 = 13 Hz, J_2 = 9 Hz, J_3 = 6 Hz, 1H), 2.10 – 1.58 (m, 9H), 1.53 – 1.21 (m, 2H), 0.79 (dd, J_1 = 7 Hz, J_2 = 2 Hz, 3H). ^{13}C NMR (75 MHz, $CDCl_3$) δ 167.3, 147.8, 147.8, 134.2, 134.2, 129.6, 129.3, 129.3, 127.8, 121.0, 120.9, 52.1, 41.0, 40.8, 39.7, 39.5, 38.3, 38.2, 31.0, 30.9, 29.7, 27.6, 27.4, 25.4, 23.6, 16.0, 15.5. HRMS (FD) m/z calcd for $C_{18}H_{24}O_2^+$: $[M]^+$ 272.1776; found: 272.1774.

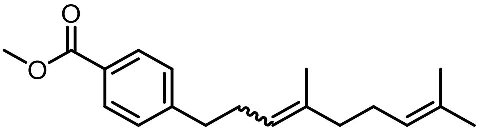

Methyl 4-(4,8-dimethylnona-3,7-dien-1-yl)benzoate (46). Prepared according to GP4 (Method B), step 1: 3 h. Purified by flash column chromatography on silica gel (Pentane : Dichloromethane 80:20) to afford the product as colourless oil (57 mg, 40%). ^{1}H NMR (300 MHz, $CDCl_3$) δ 7.98 – 7.91 (m, 2H), 7.28 – 7.22 (m, 2H), 5.20 – 5.11 (m, 1H), 5.11 – 5.03 (m, 1H), 3.90 (s, 3H), 2.74 – 2.64 (m, 2H), 2.37 – 2.26 (m, 2H), 2.11 – 1.93 (m, 4H), 1.71 – 1.65 (m, 4H), 1.61 – 1.58 (m, 3H), 1.53 (m, 2H). ^{13}C NMR (75 MHz, $CDCl_3$) δ 167.4, 167.3, 148.1, 136.4, 136.4, 131.8, 131.5, 129.8, 129.7, 128.7, 128.6, 127.8, 127.8, 124.4, 124.3, 124.0, 123.1, 52.1, 39.8, 36.5, 36.3, 32.1, 29.8, 29.6, 29.6, 26.8, 26.6, 25.9, 25.8, 23.5, 17.8, 17.8, 16.1. HRMS (EI) m/z calcd for $C_{19}H_{26}O_2^+$: $[M]^+$ 286.1933; found: 286.1938 and 286.1930.

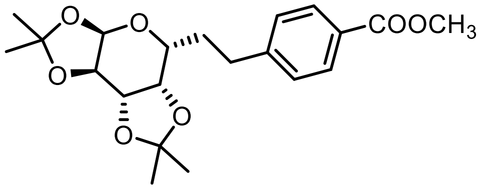

Methyl 4-(2-((3aR,5R,5aS,8aS,8bR)-2,2,7,7-tetramethyltetrahydro-5H-bis([1,3]dioxolo)[4,5-b:4',5'-d]pyran-5-yl)ethyl)benzoate (47). Prepared according to GP4 (Method B), step 1: olefin was added into a flame-dried Schlenk flask and dissolved in 0.2 mL anhydrous THF. Subsequently, 9-BBN was added dropwise at 0 °C. Purified by flash column chromatography on silica gel (Pentane : Ethyl Acetate: 90:10) to afford the product as colourless oil (70 mg, 36%, dr 25:1). ^{1}H NMR (300 MHz, $CDCl_3$) δ 8.00 – 7.86 (m, 2H), 7.35 – 7.23 (m, 2H), 5.54 (d, *J* = 5 Hz, 1H), 4.54 (dd, J_1 = 8 Hz, J_2 = 2 Hz, 1H), 4.27 (dd, J_1 = 5 Hz, J_2 = 2 Hz, 1H), 4.06 (dd, J_1 = 8 Hz, J_2 = 2 Hz, 1H), 3.88 (s, 3H), 3.64 (ddd, J_1 = 10 Hz, J_2 = 4 Hz, J_3 = 2 Hz, 1H), 2.92 – 2.66 (m, 2H), 2.18 – 1.98 (m, 1H), 1.89 – 1.73 (m, 1H), 1.45 (s, 3H), 1.32 (s, 6H), 1.29 (s, 3H). ^{13}C NMR (75 MHz, $CDCl_3$) δ 167.2, 147.5, 129.8 (2C), 128.9 (2C), 127.9, 109.1, 108.4, 96.7, 73.0, 71.1, 70.5, 66.1, 52.0, 31.6, 31.3, 26.1, 25.9, 25.0, 24.5. HRMS (EI) m/z calcd for $C_{21}H_{28}O_7^+$: $[M]^+$ 392.1835; found: 392.1847.

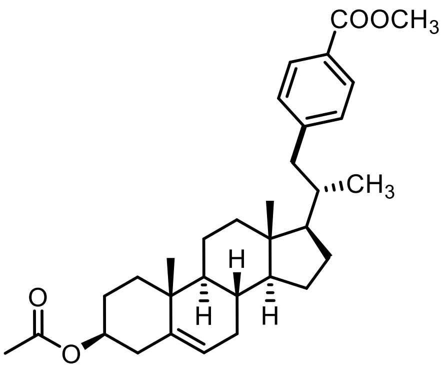

Methyl 4-((S)-2-((3S,8S,9S,10R,13R,14S,17R)-3-acetoxy-10,13-dimethyl-2,3,4,7,8,9,10,11,12,13,14,15,16,17-tetradecahydro-1H-cyclopenta[a]phenanthren-17-

yl)propyl)benzoate (48). Prepared according to GP4 (Method B), step 1: olefin was added into a flame-dried Schlenk flask and dissolved in 0.2 mL anhydrous THF. Subsequently, 9-BBN was added dropwise at 0 °C; step 2: 0.25 mmol scale of **2a.** The reaction mixture was irradiated 3 h then removed solvent, extract with ethyl acetate, dried over Na_2SO_4, filtered and concentrated under reduced pressure. Purified by flash column chromatography on silica gel (Pentane : Ethyl Acetate: 85:15) to afford the product as colourless solid (61 mg, 50%, dr 10:1). ^{1}H NMR (400 MHz, $CDCl_3$) δ 7.95 – 7.88 (m, 2H), 7.20 – 7.14 (m, 2H), 5.39 – 5.31 (m, 1H), 4.63 – 5.53 (m, 1H), 3.86 (s, 3H), 2.90 (dd, J_1 = 13 Hz, J_2 = 3 Hz, 1H), 2.34 – 2.23 (m, 2H), 2.15 – 2.07 (m, 1H), 2.03 – 1.89 (m, 6H), 1.87 – 1.78 (m, 2H), 1.71 – 1.36 (m, 8H), 1.21 – 0.86 (m, 9H), 0.78 (d, J = 6 Hz, 3H), 0.69 (s, 3H). ^{13}C NMR (101 MHz, $CDCl_3$) δ 170.5, 167.2, 147.4, 139.7, 129.5 (2C), 129.4 (2C), 127.6, 122.6, 73.9, 56.8, 56.6, 51.9, 50.0, 42.9, 42.5, 39.7, 38.6, 38.2, 37.0, 36.6, 31.9 (2C), 29.0, 27.8, 24.4, 21.5, 21.0, 19.3, 18.3, 11.9. HRMS (EI) m/z calcd for $C_{30}H_{40}O_2^+$: $[M-CH_3COOH]^+$ 432.3028; found: 432.3018.

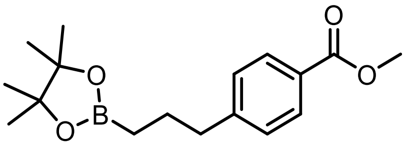

Methyl 4-(3-(4,4,5,5-tetramethyl-1,3,2-dioxaborolan-2-yl)propyl)benzoate (49). Prepared according to GP4 (Method B), 1.0 equiv. alkylborane**.** Purified by flash column chromatography on silica gel (Pentane : Ethyl Acetate: 100:0 → 95:5) to afford the product as colourless oil (71 mg, 47%). ^{1}H NMR (300 MHz, $CDCl_3$) δ 7.96 – 7.88 (m, 1H), 7.25 – 7.18 (m, 1H), 3.87 (s, 2H), 2.64 (dd, J = 9, 7 Hz, 1H), 1.72 (p, J = 8 Hz, 1H), 1.22 (s, 6H), 0.80 (t, J = 8 Hz, 1H). ^{13}C NMR (75 MHz, $CDCl_3$) δ 167.3, 148.3, 129.6, 128.6, 127.7, 83.1, 52.0, 38.6, 25.8, 24.9. The signal of the α-B-carbon was not observed. ^{11}B NMR (96 MHz, $CDCl_3$) δ 33.9. Spectroscopic data are in accordance with the literature.[30]

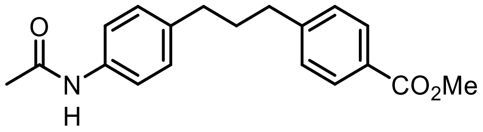

Methyl 4-(3-(4-acetamidophenyl)propyl)benzoate (50). Prepared according to literature.[33] $[Ir(dF(CF_3)ppy)_2(dtbbpy)]PF_6$ (1 mol %), 4-bromoacetanilide (0.2 mmol) and boronic ester **45** (2.0 equiv), were added to a 7 mL glass vial equipped with a magnetic stir bar and dissolved in 1 mL DMF. Morpholine (1.5 equiv) was added. In a second vial $NiCl_2$·glyme (5 mol %) and dtbbpy (5 mol %) were added and dissolved in 1 mL DMF. The mixture was sonicated for 30 seconds and heated afterwards to 100 °C (heat gun) until a clear green solution was obtained. Both mixtures were combined and the resulting reaction mixture was irradiated with UFO

reactor for 2 h. The mixture was poured into brine and extracted 3 times with ethyl acetate. The combined organic phases were dried over Na_2SO_4. After filtration, the solvent was removed under reduced pressure and the residue was purified by flash chromatography on silica gel (Pentane : Ethyl Acetate: 50:50) to afford the product as colourless solid (39 mg, 63%), m.p. 107~109 °C. ^{1}H NMR (400 MHz, $CDCl_3$) δ 7.95 (d, J = 8 Hz, 2H), 7.48 (s, 1H), 7.41 (d, J = 8 Hz, 2H), 7.23 (d, J = 8 Hz, 2H), 7.10 (d, J = 8 Hz, 2H), 3.90 (s, 3H), 2.67 (t, J = 8 Hz, 2H), 2.59 (t, J = 8 Hz, 2H), 2.15 (s, 3H), 1.94 (h, J = 8 Hz, 2H). ^{13}C NMR (101 MHz, $CDCl_3$) δ 168.5, 167.3, 147.9, 138.0, 135.9, 129.8 (2C), 129.0 (2C), 128.6 (2C), 127.9, 120.2 (2C), 52.1, 35.4, 34.8, 32.7, 24.6. HRMS (EI) m/z calcd for $C_{19}H_{21}N_1O_3^+$: $[M]^+$ 311.1521; found: 311.1512.

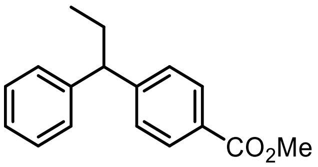

Methyl 4-(1-phenylpropyl)benzoate (38γ). Prepared according to GP3 (Method A), step 2: 6,6′-dimethyl-2,2′-dipyridyl as ligand, 16 h. Purified by flash column chromatography on silica gel (Pentane : Ethyl Acetate: 95:5, second column: Pentane : Dichloromethane: 80:20) to afford the product as colourless oil (51 mg, 40%, rr > 20:1). ^{1}H NMR (300 MHz, $CDCl_3$) δ 8.06 – 7.96 (m, 2H), 7.40 – 7.19 (m, 7H), 3.95 – 3.86 (m, 1H), 3.92 (s, 3H), 2.14 (p, J = 7 Hz, 2H), 0.95 (t, J = 7 Hz, 3H). ^{13}C NMR (75 MHz, $CDCl_3$) δ 167.1, 150.7, 144.3, 129.9 (2C), 128.6 (2C), 128.1, 128.1 (2C), 128.0 (2C), 126.4, 53.3, 52.1, 28.5, 12.8. Spectroscopic data are in accordance with the literature.[34]

2.4 NMR spectra

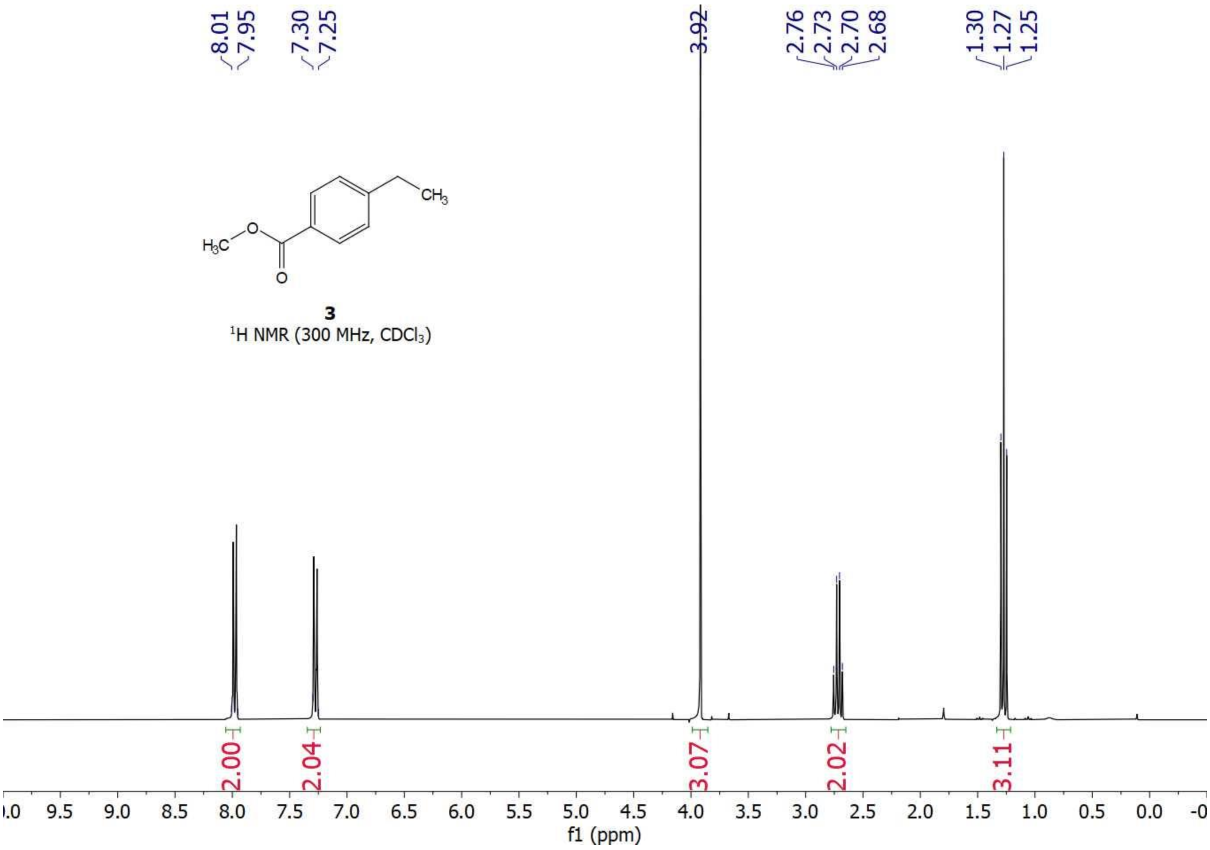

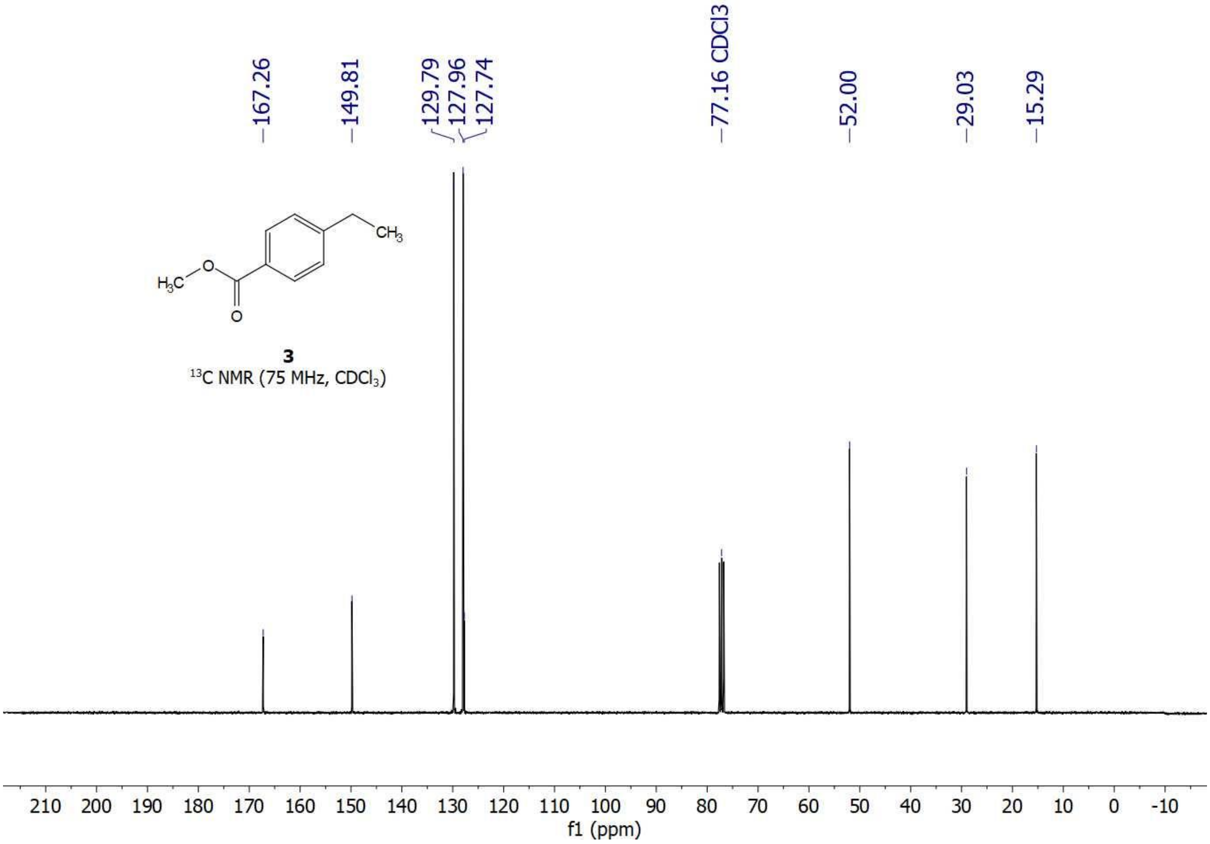

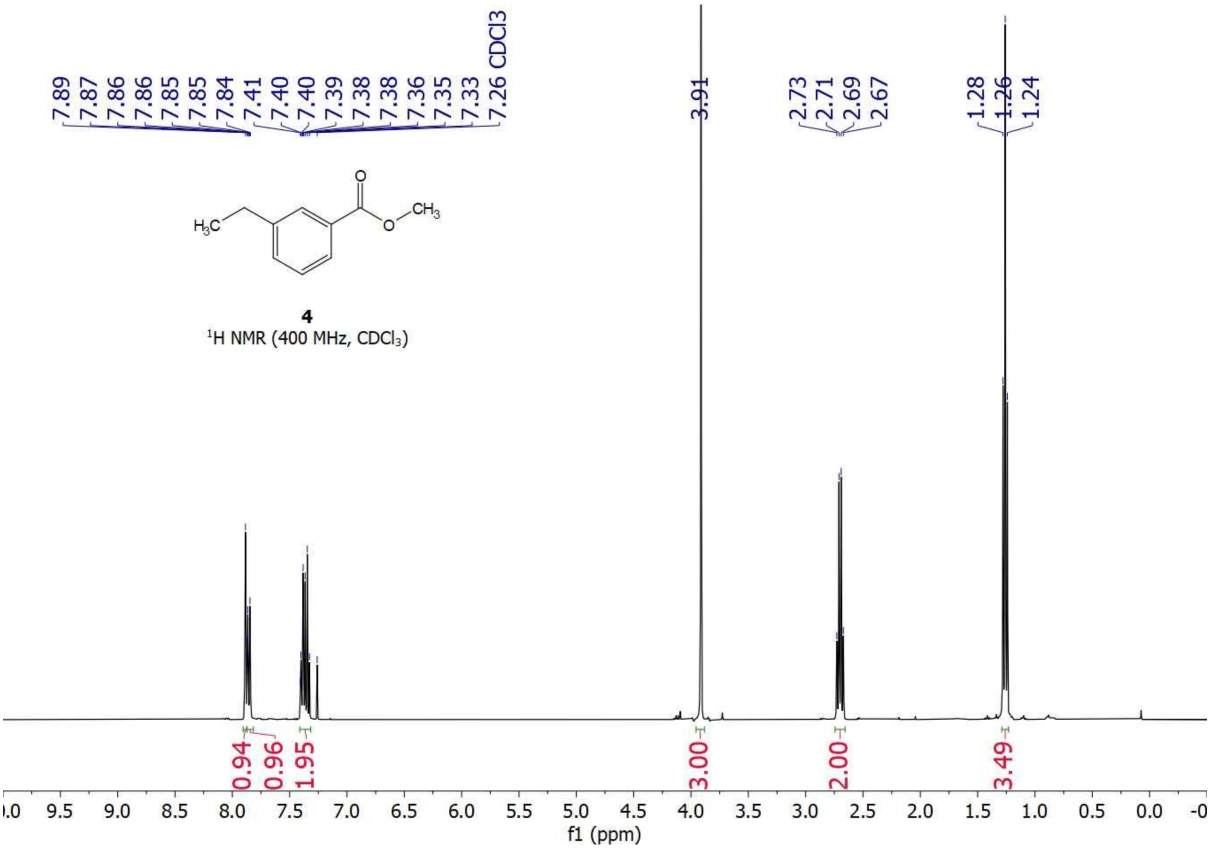

4
^{1}H NMR (400 MHz, $CDCl_3$)

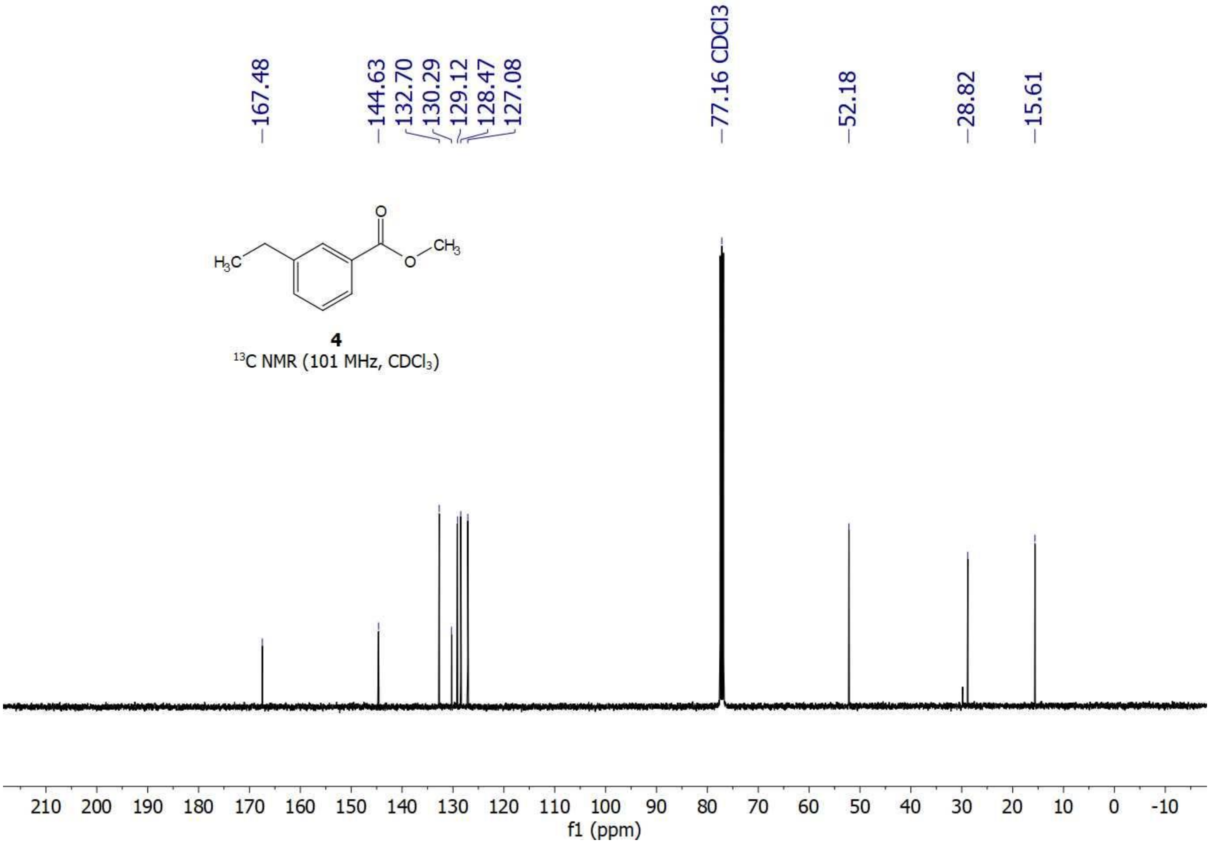

4
^{13}C NMR (101 MHz, $CDCl_3$)

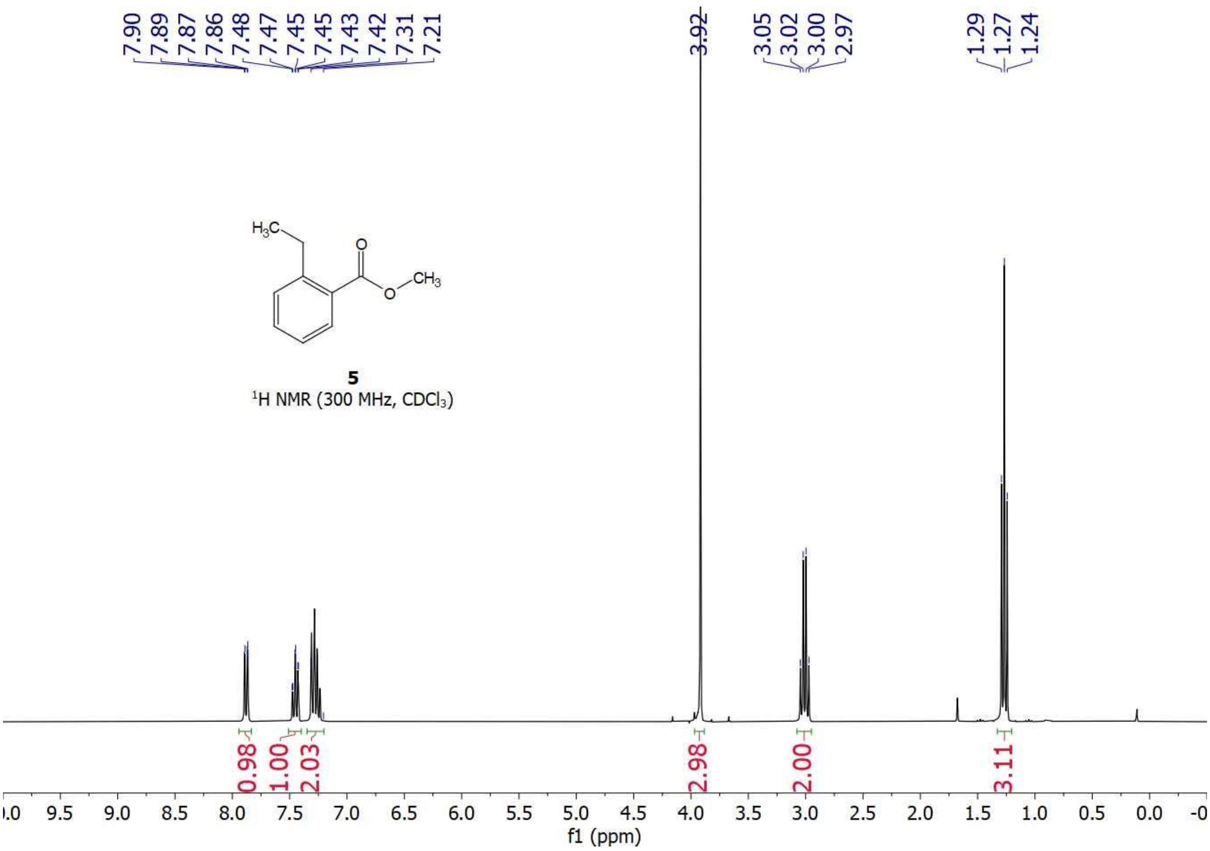
7.90
7.89
7.87
7.86
7.48
7.47
7.45
7.45
7.43
7.42
7.31
7.21
3.92
3.05
3.02
3.00
2.97
1.29
1.27
1.24
H3C
O
O
CH3
5
1H NMR (300 MHz, CDCl3)
0.98
1.00
2.03
2.98
2.00
3.11
9.5 9.0 8.5 8.0 7.5 7.0 6.5 6.0 5.5 5.0 4.5 4.0 3.5 3.0 2.5 2.0 1.5 1.0 0.5 0.0
f1 (ppm)

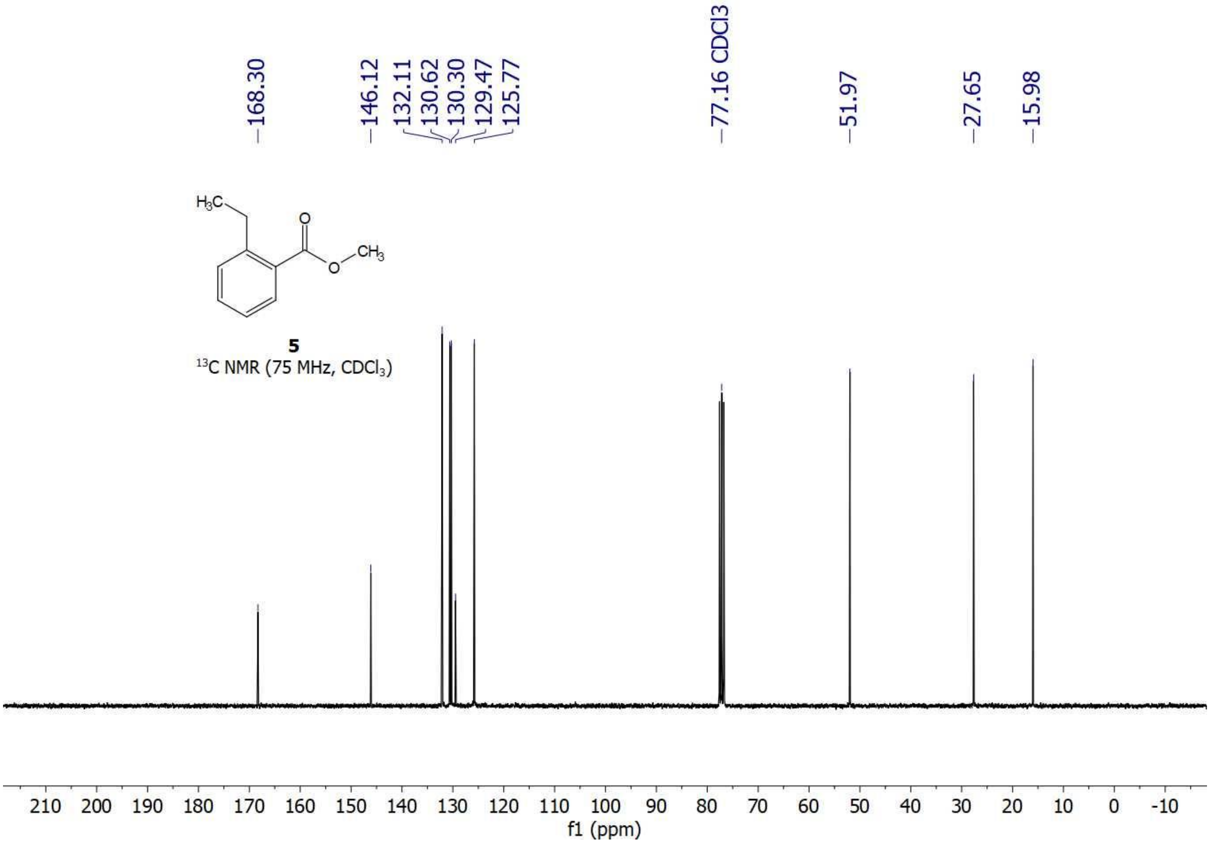
168.30
146.12
132.11
130.62
130.30
129.47
125.77
77.16 CDCl3
51.97
27.65
15.98
H3C
O
O
CH3
5
13C NMR (75 MHz, CDCl3)
210 200 190 180 170 160 150 140 130 120 110 100 90 80 70 60 50 40 30 20 10 0 -10
f1 (ppm)

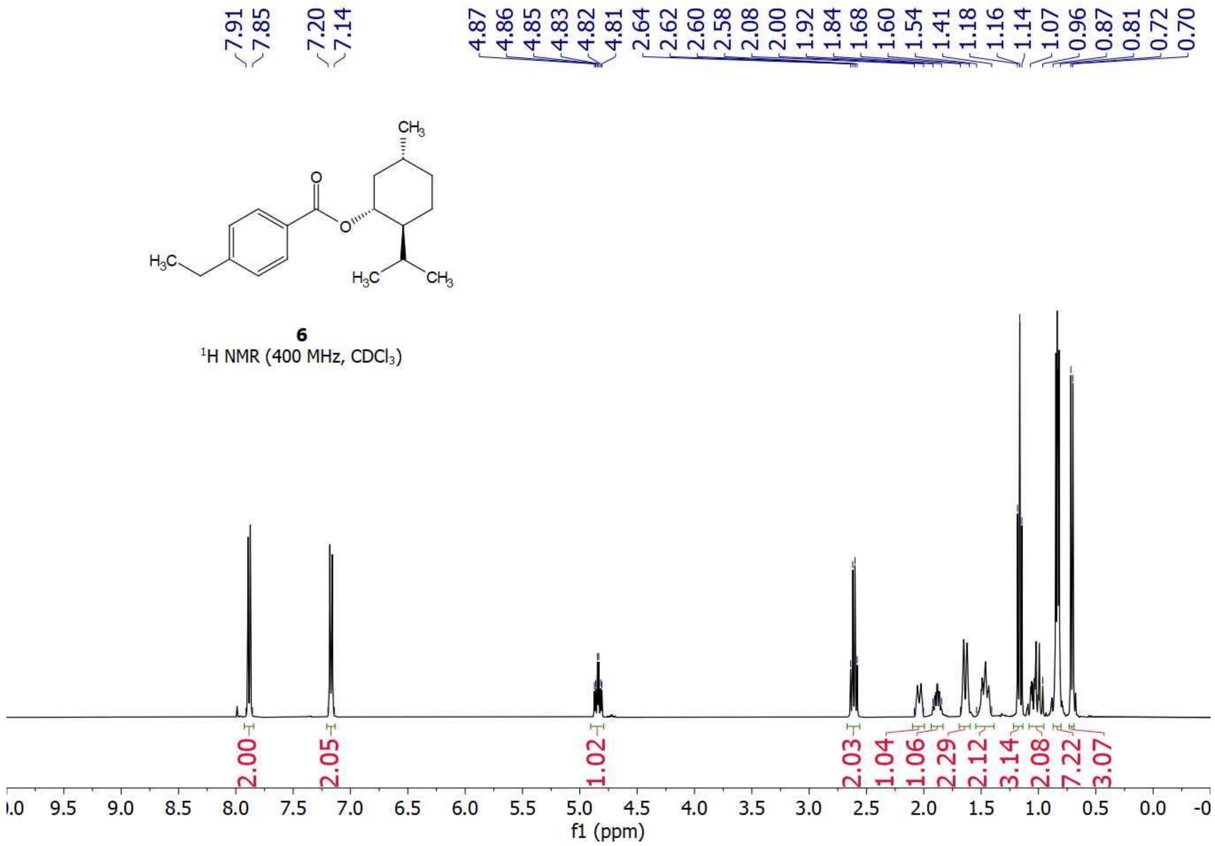
7.91
7.85
7.20
7.14
4.87
4.86
4.85
4.83
4.82
4.81
2.64
2.62
2.60
2.58
2.08
2.00
1.92
1.84
1.68
1.60
1.54
1.41
1.18
1.16
1.14
1.07
0.96
0.87
0.81
0.72
0.70
6
^{1}H NMR (400 MHz, $CDCl_3$)
2.00
2.05
1.02
2.03
1.04
1.06
2.29
2.12
3.14
2.08
7.22
3.07
f1 (ppm)

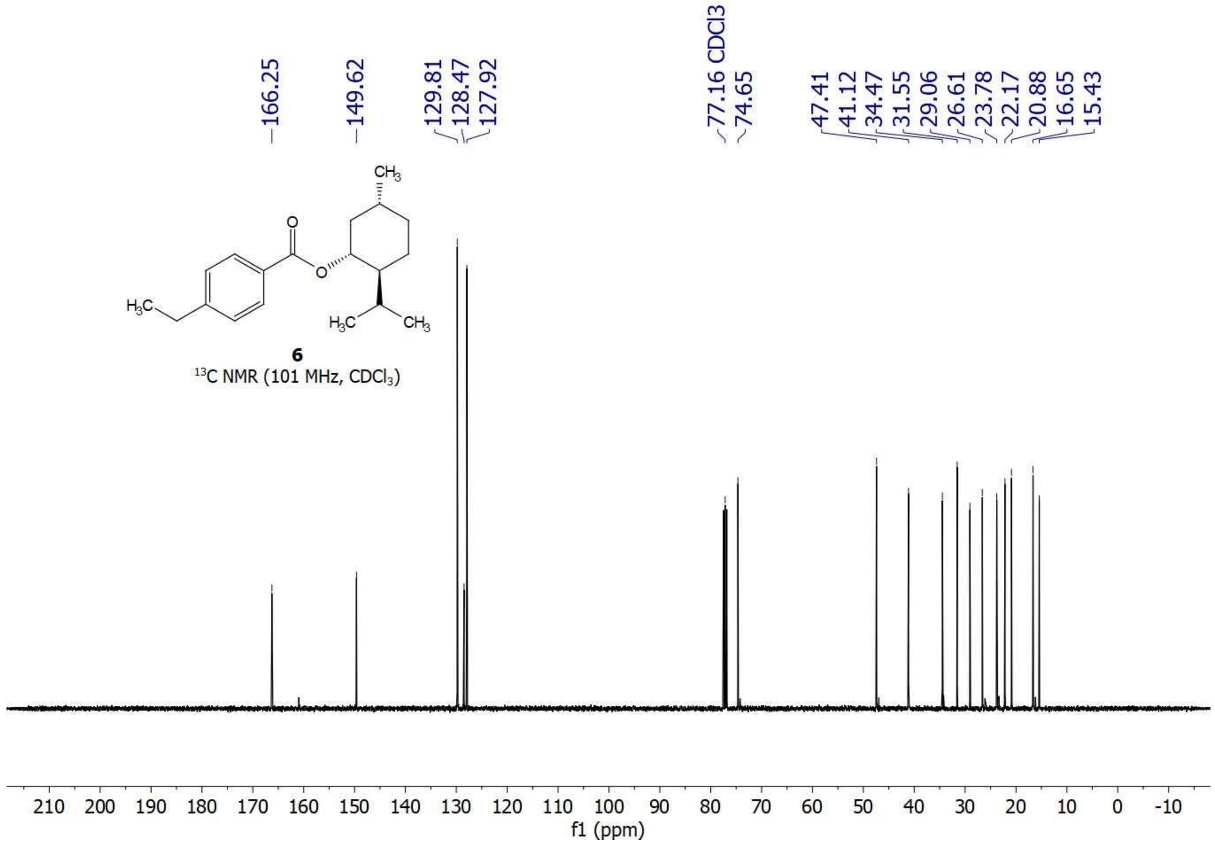
166.25
149.62
129.81
128.47
127.92
77.16 CDCl3
74.65
47.41
41.12
34.47
31.55
29.06
26.61
23.78
22.17
20.88
16.65
15.43
6
^{13}C NMR (101 MHz, $CDCl_3$)
f1 (ppm)

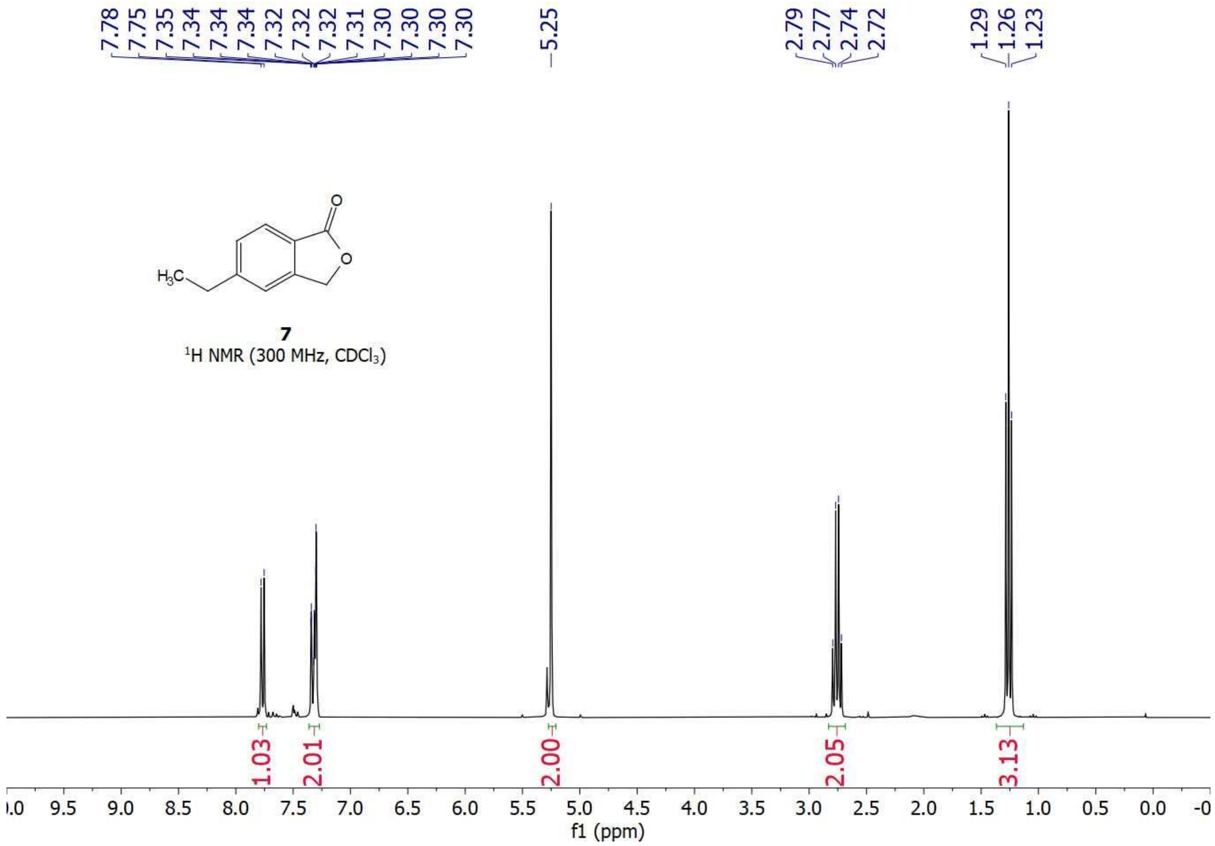

7
^{1}H NMR (300 MHz, $CDCl_3$)

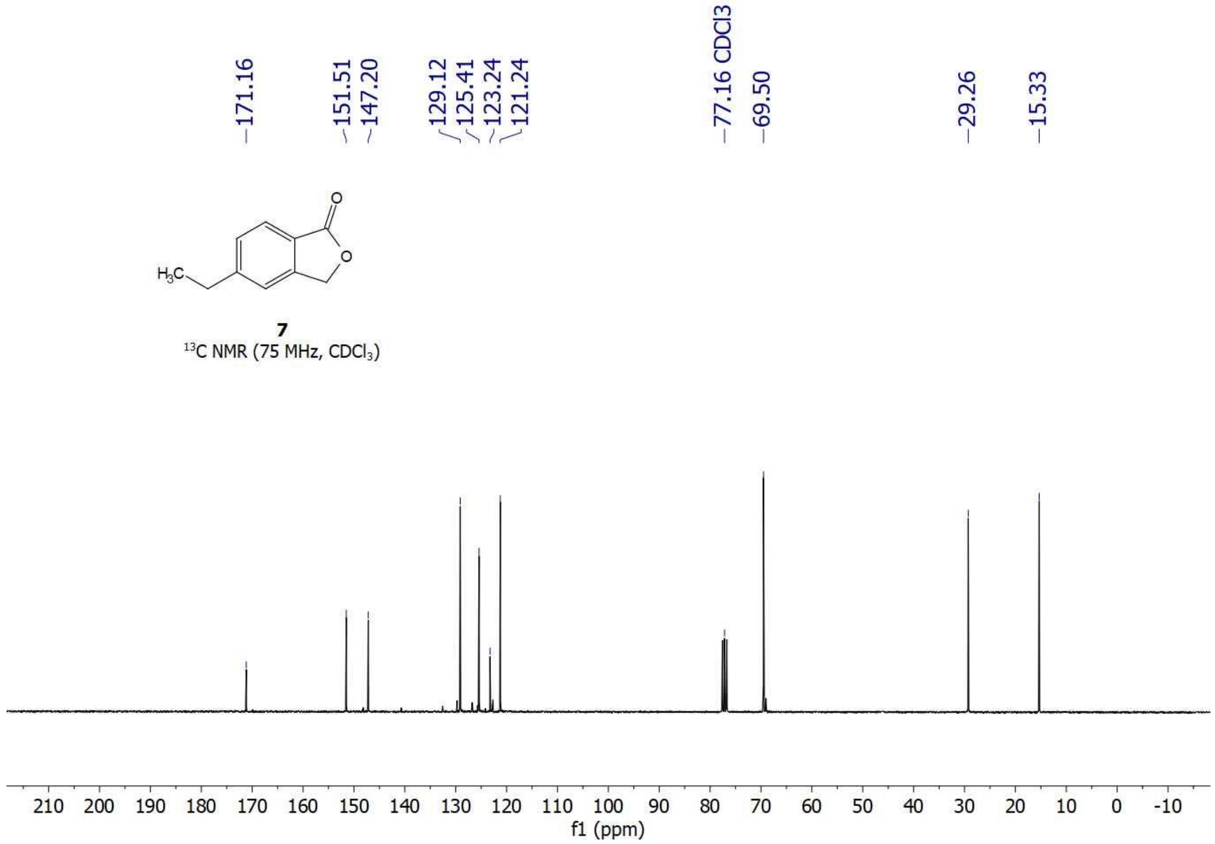

7
^{13}C NMR (75 MHz, $CDCl_3$)

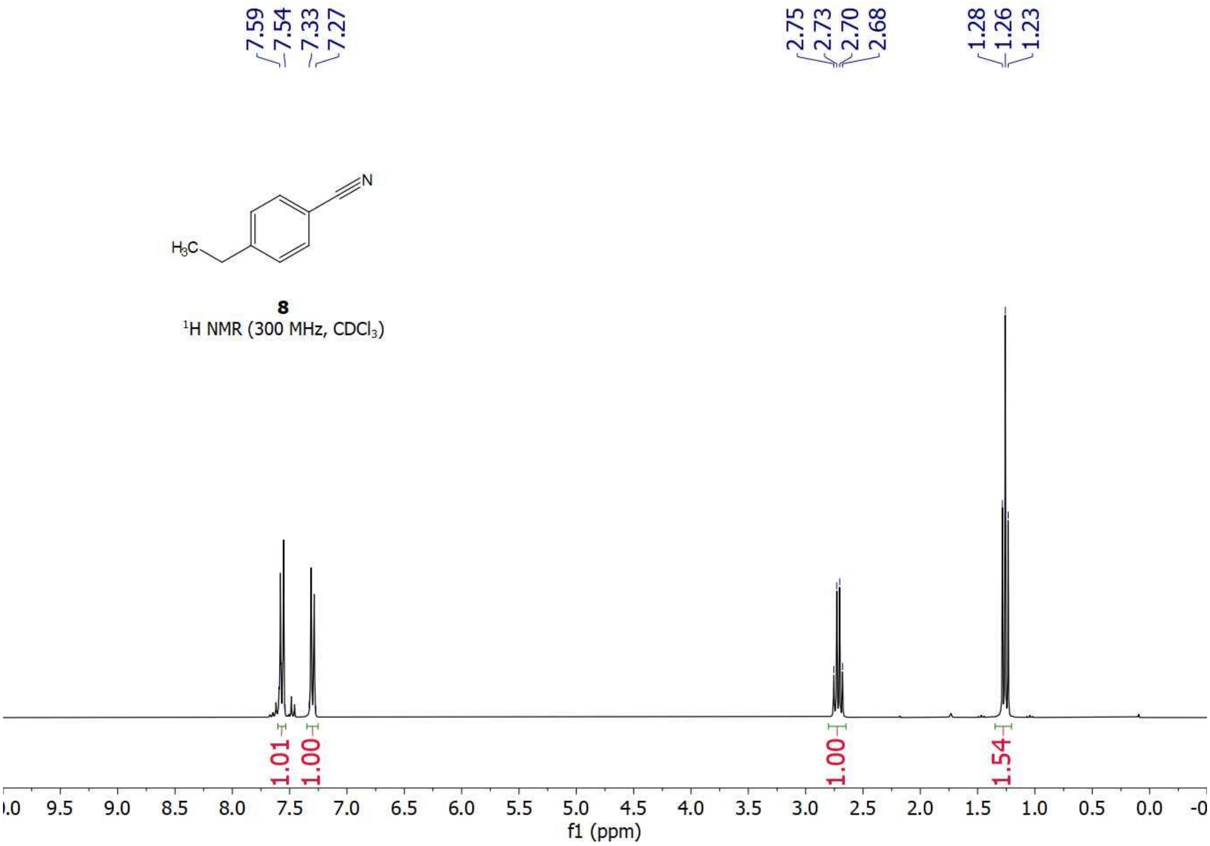

8
^{1}H NMR (300 MHz, $CDCl_3$)

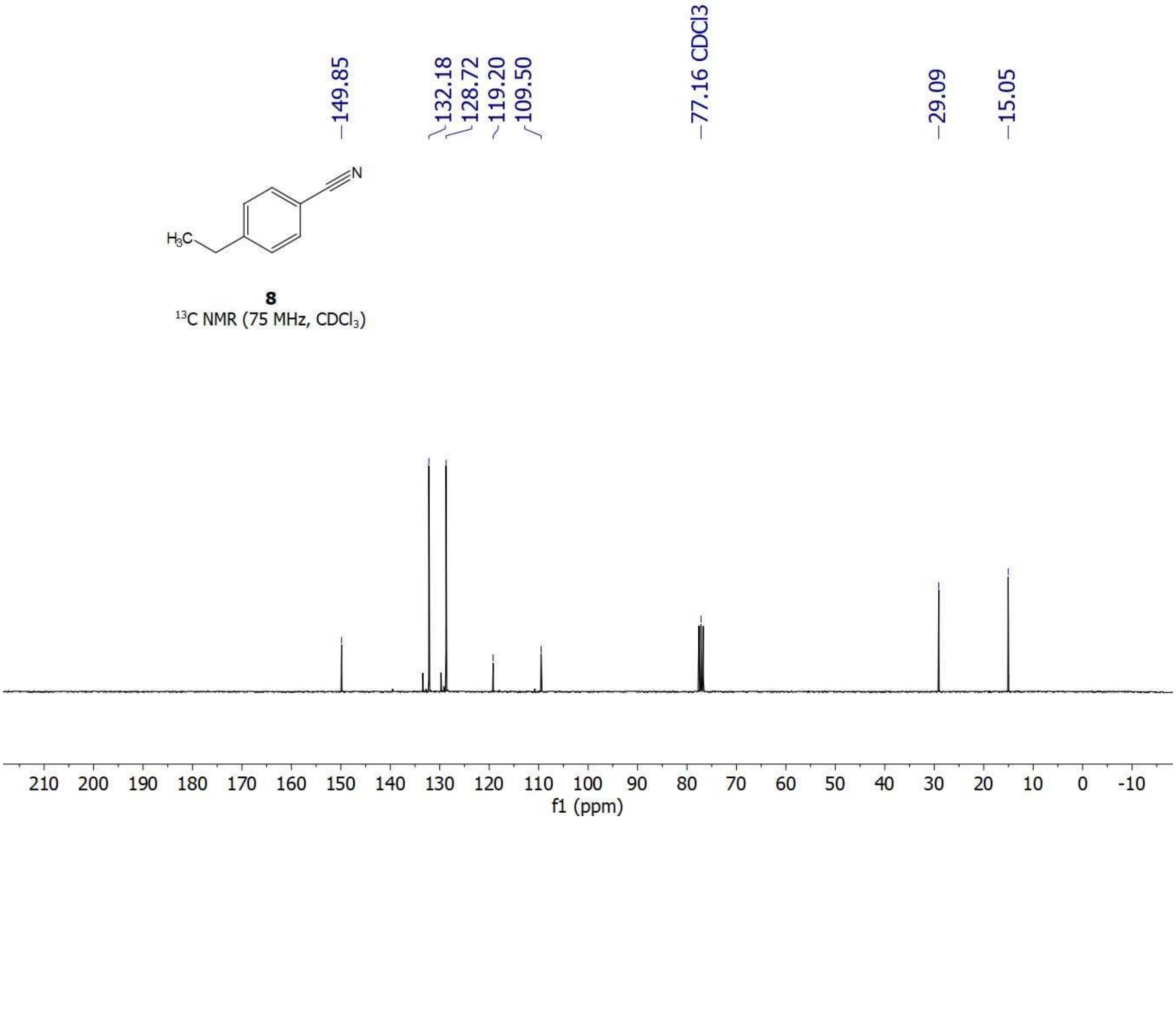

8
^{13}C NMR (75 MHz, $CDCl_3$)

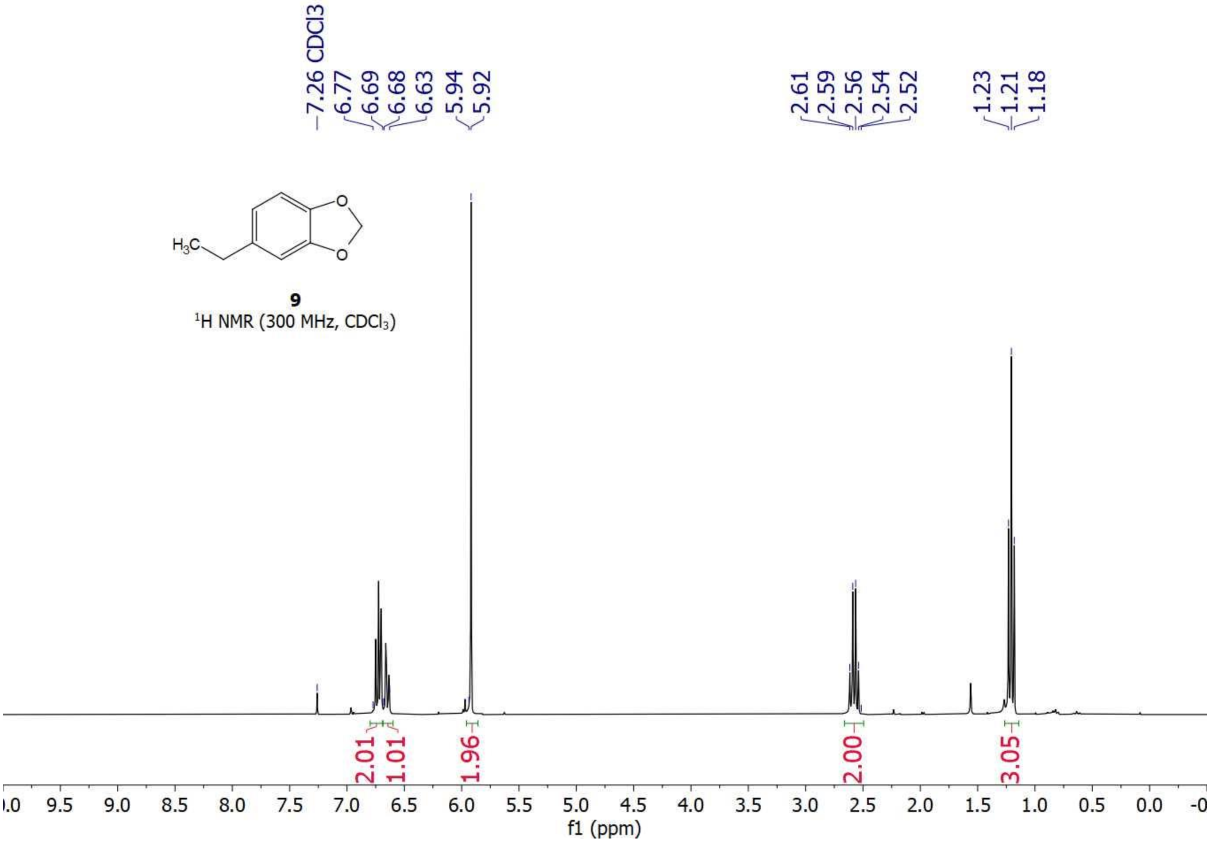
7.26 CDCl3
6.77
6.69
6.68
6.63
5.94
5.92
2.61
2.59
2.56
2.54
2.52
1.23
1.21
1.18
H3C
O
O
9
1H NMR (300 MHz, CDCl3)
2.01
1.01
1.96
2.00
3.05
f1 (ppm)

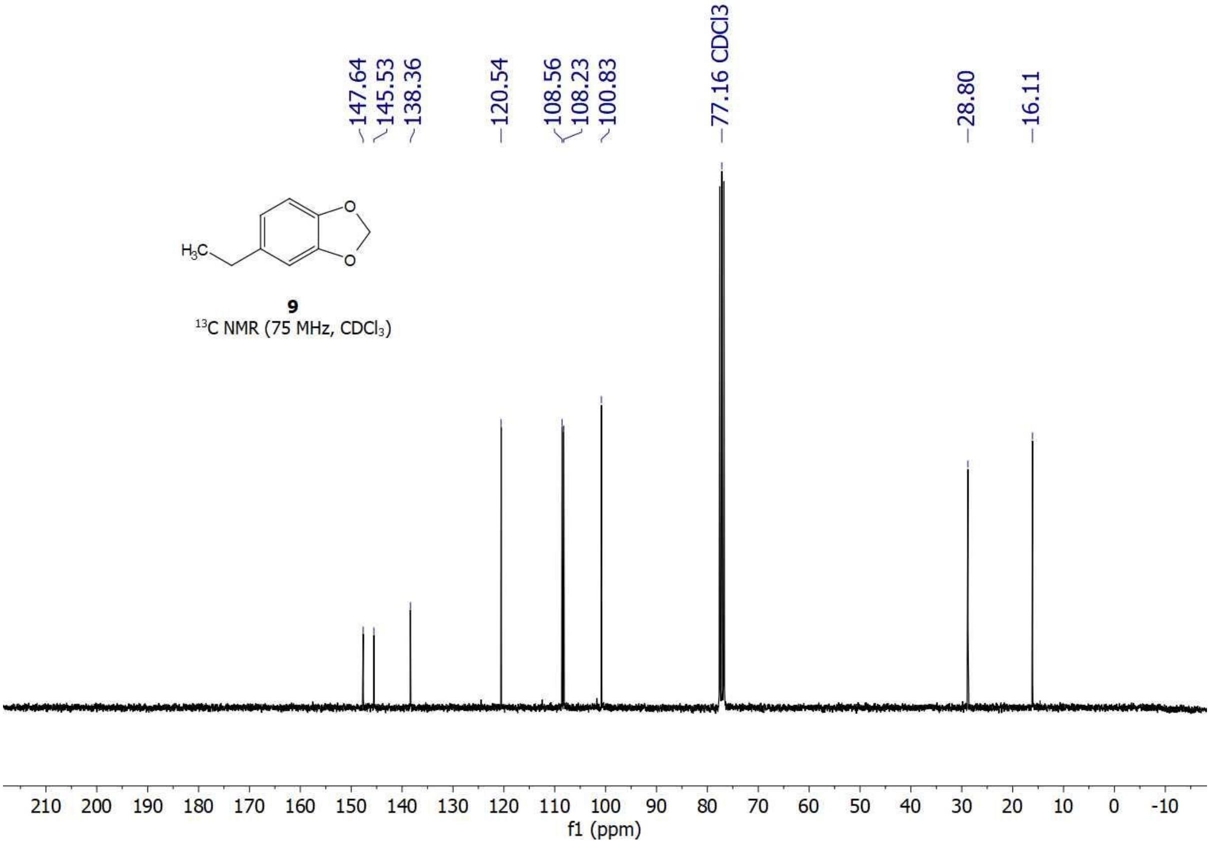
147.64
145.53
138.36
120.54
108.56
108.23
100.83
77.16 CDCl3
28.80
16.11
H3C
O
O
9
13C NMR (75 MHz, CDCl3)
f1 (ppm)

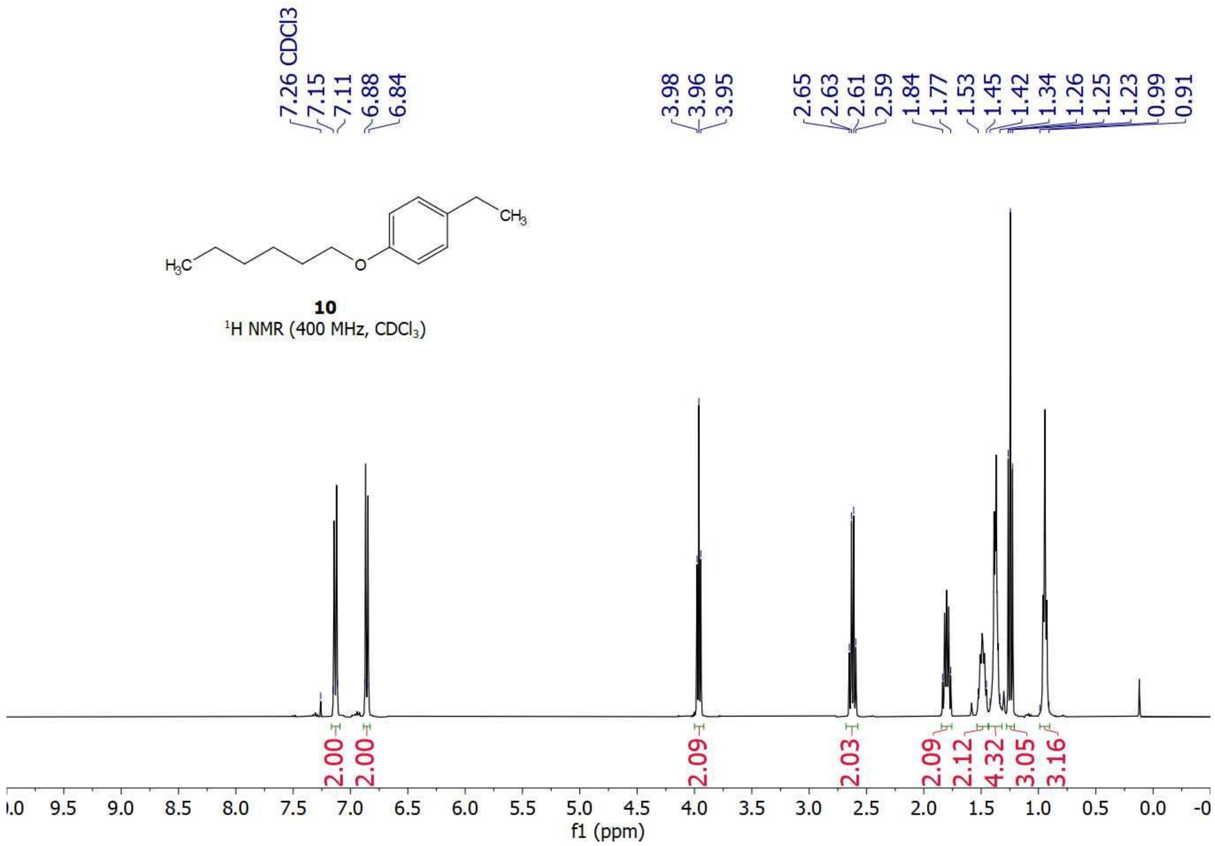
7.26 CDCl3
7.15
7.11
6.88
6.84
3.98
3.96
3.95
2.65
2.63
2.61
2.59
1.84
1.77
1.53
1.45
1.42
1.34
1.26
1.25
1.23
0.99
0.91
CH3
H3C
O
10
1H NMR (400 MHz, CDCl3)
2.00
2.00
2.09
2.03
2.09
2.12
4.32
3.05
3.16
f1 (ppm)

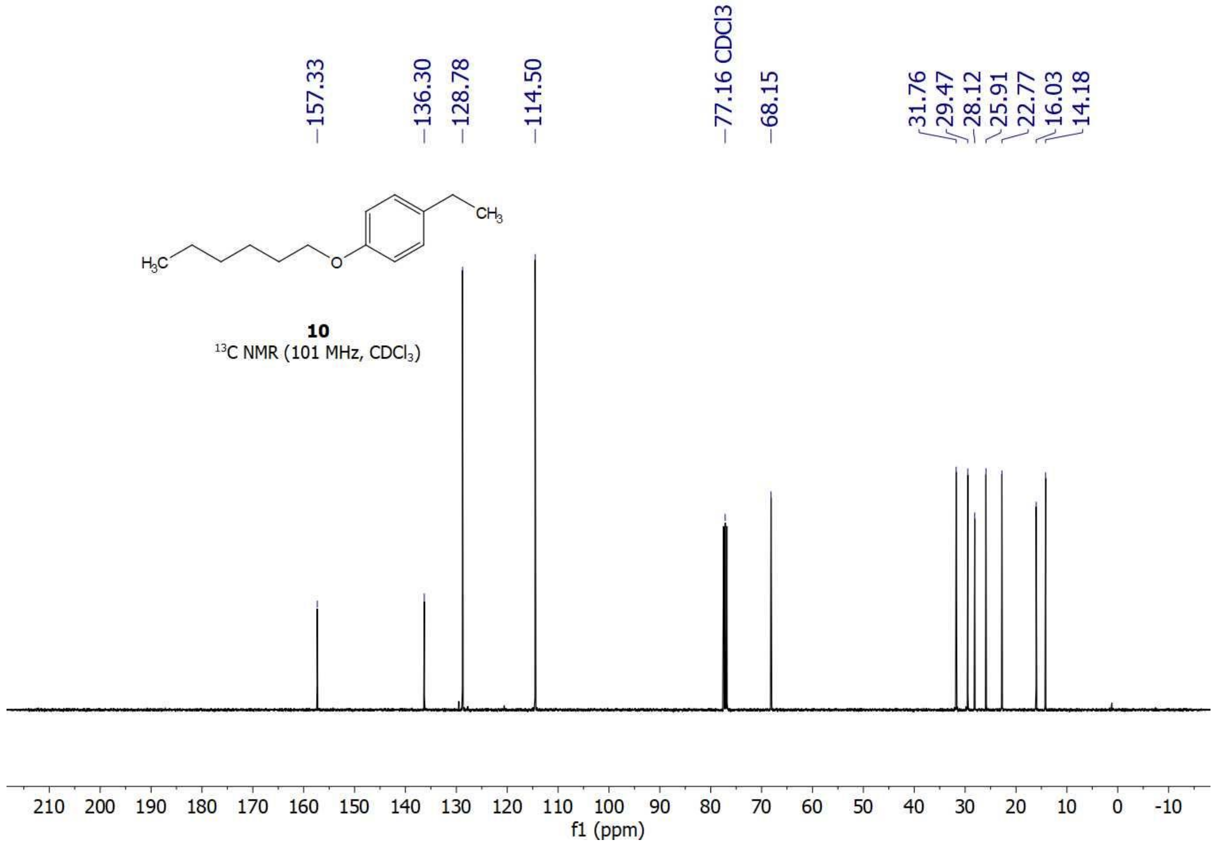
157.33
136.30
128.78
114.50
77.16 CDCl3
68.15
31.76
29.47
28.12
25.91
22.77
16.03
14.18
CH3
H3C
O
10
13C NMR (101 MHz, CDCl3)
f1 (ppm)

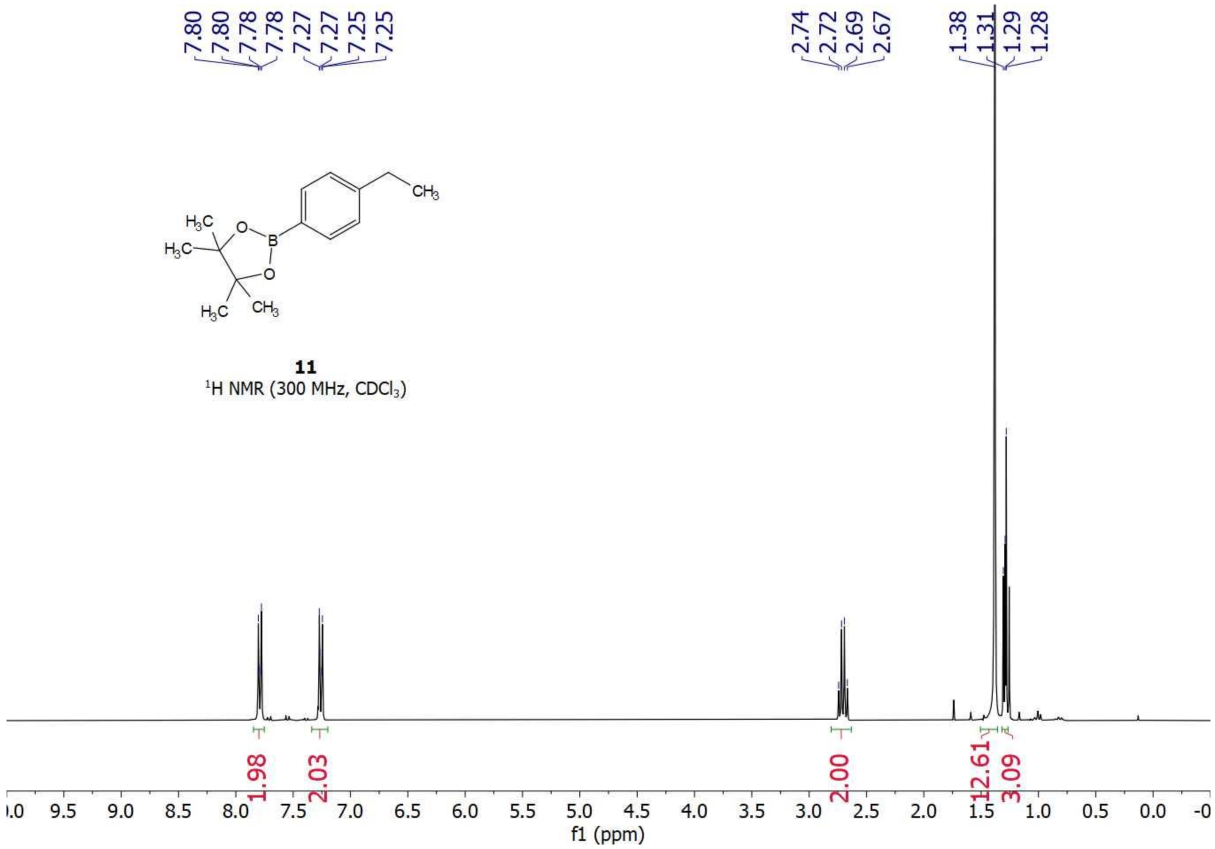

7.80
7.80
7.78
7.78
7.27
7.27
7.25
7.25
2.74
2.72
2.69
2.67
1.38
1.31
1.29
1.28
11
^{1}H NMR (300 MHz, CDCl$_3$)
1.98
2.03
2.00
12.61
3.09
f1 (ppm)

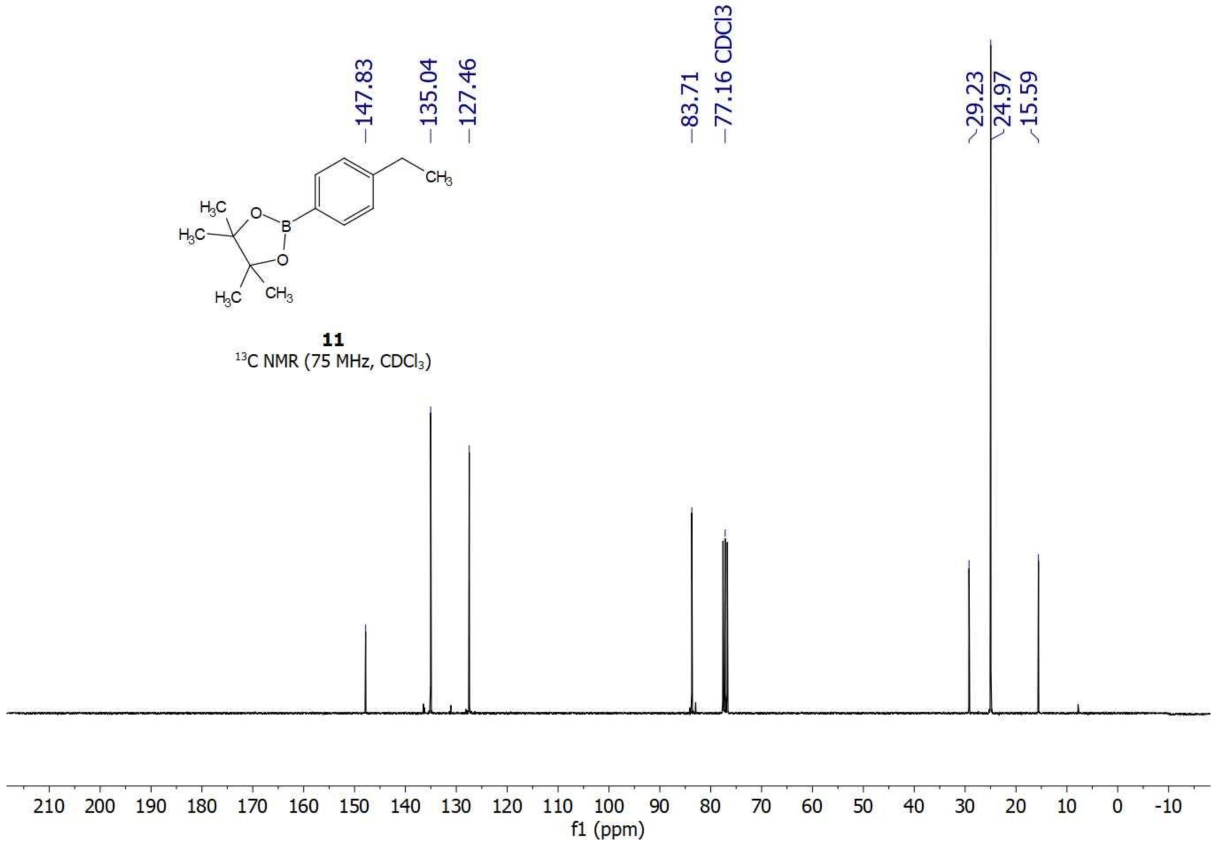

147.83
135.04
127.46
83.71
77.16 CDCl3
29.23
24.97
15.59
11
^{13}C NMR (75 MHz, CDCl$_3$)
f1 (ppm)

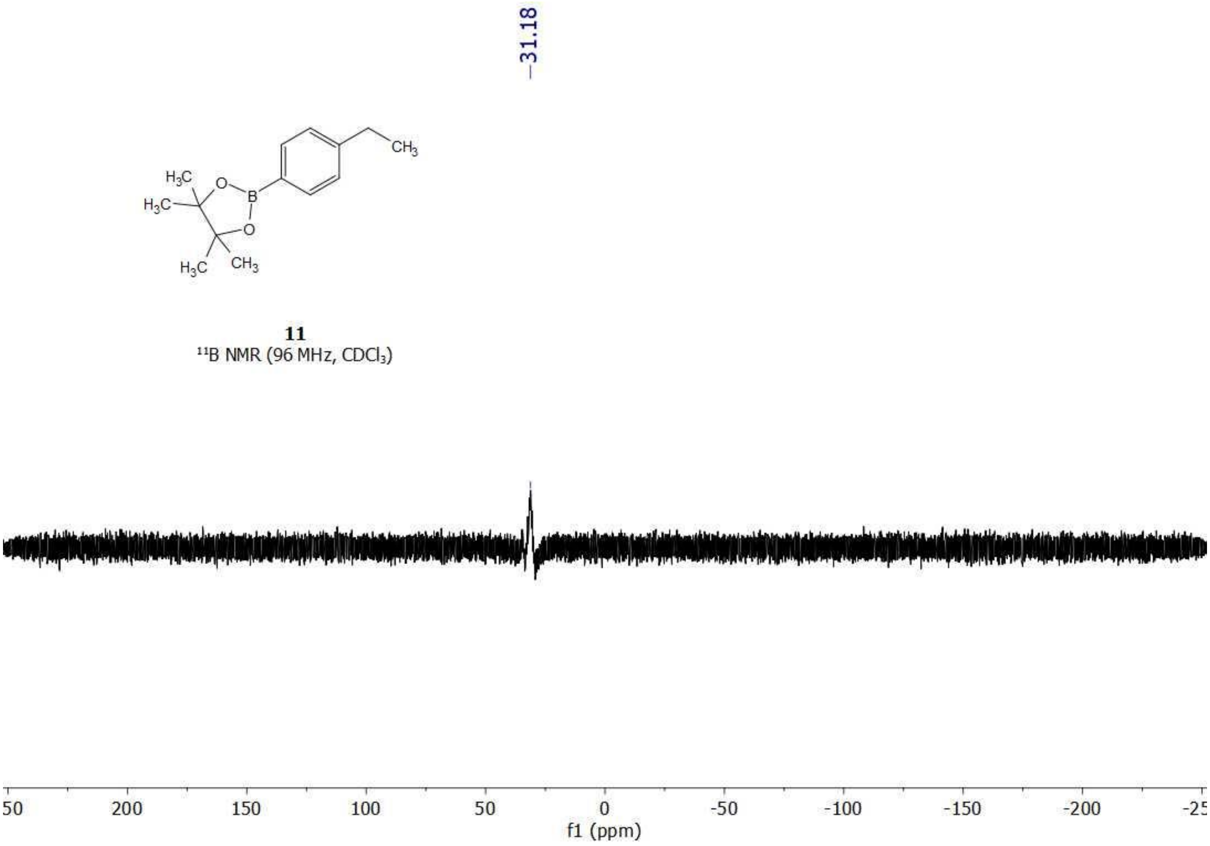

31.18
CH3
H3C
O
B
O
H3C
H3C
CH3
11
11B NMR (96 MHz, CDCl3)
50
200
150
100
50
0
-50
-100
-150
-200
f1 (ppm)

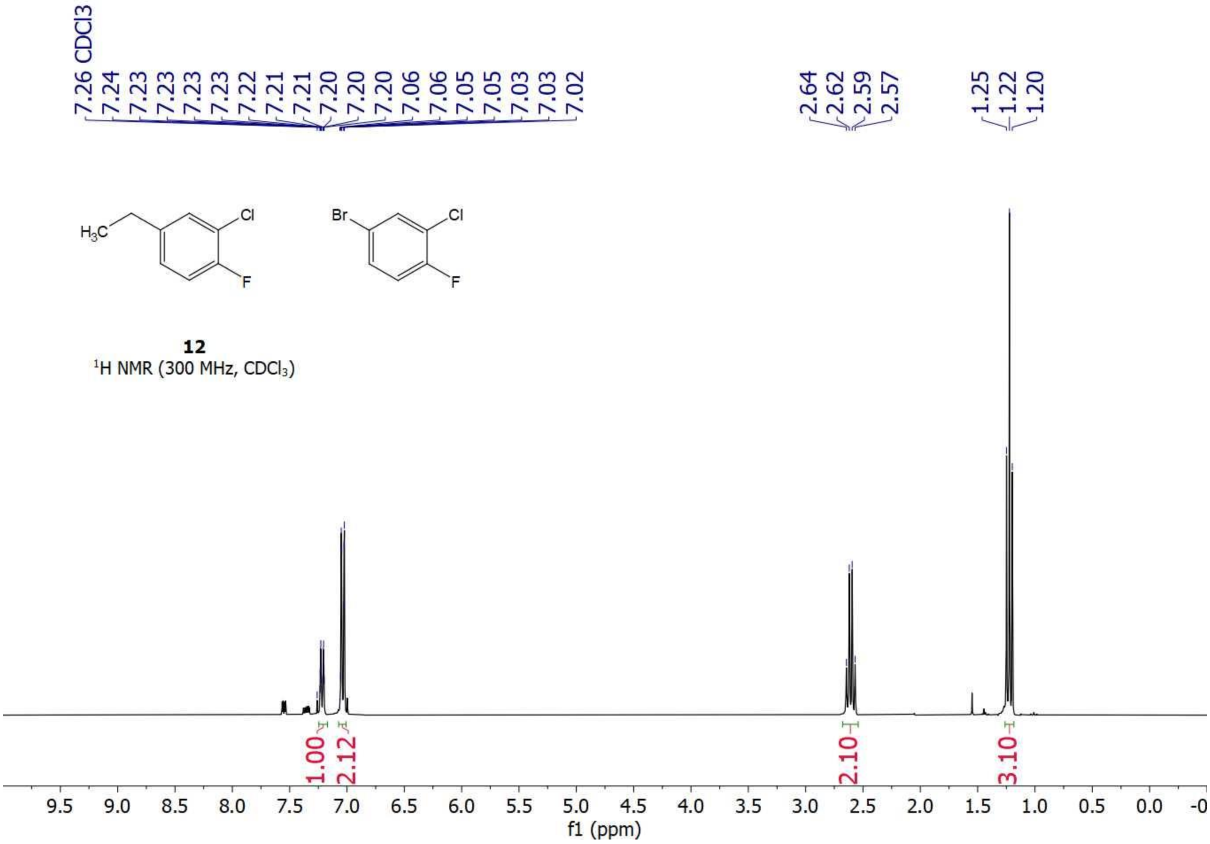

7.26 CDCl3
7.24
7.23
7.23
7.23
7.23
7.22
7.21
7.21
7.20
7.20
7.20
7.06
7.06
7.05
7.05
7.03
7.03
7.02
2.64
2.62
2.59
2.57
1.25
1.22
1.20
H3C
Cl
F
Br
Cl
F
12
1H NMR (300 MHz, CDCl3)
1.00
2.12
2.10
3.10
9.5
9.0
8.5
8.0
7.5
7.0
6.5
6.0
5.5
5.0
4.5
4.0
3.5
3.0
2.5
2.0
1.5
1.0
0.5
0.0
f1 (ppm)

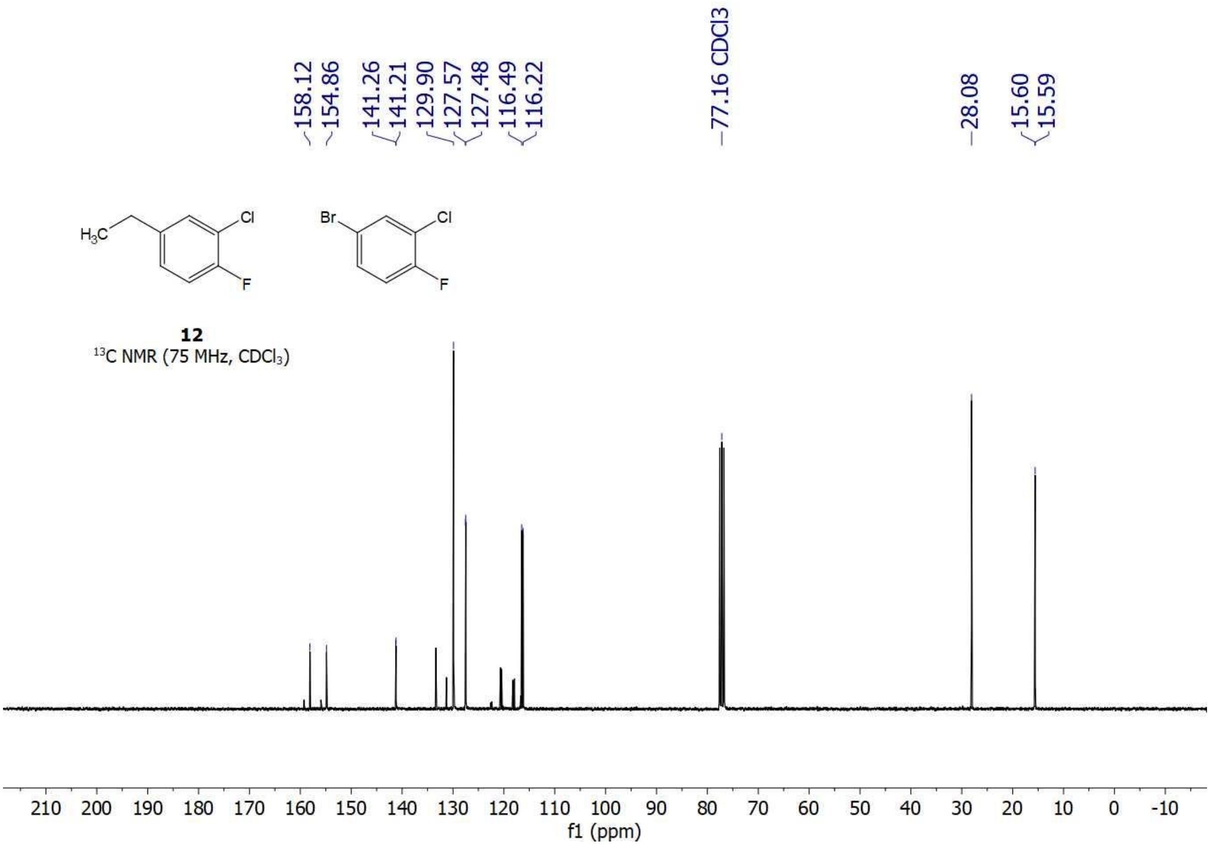

12

^{13}C NMR (75 MHz, $CDCl_3$)

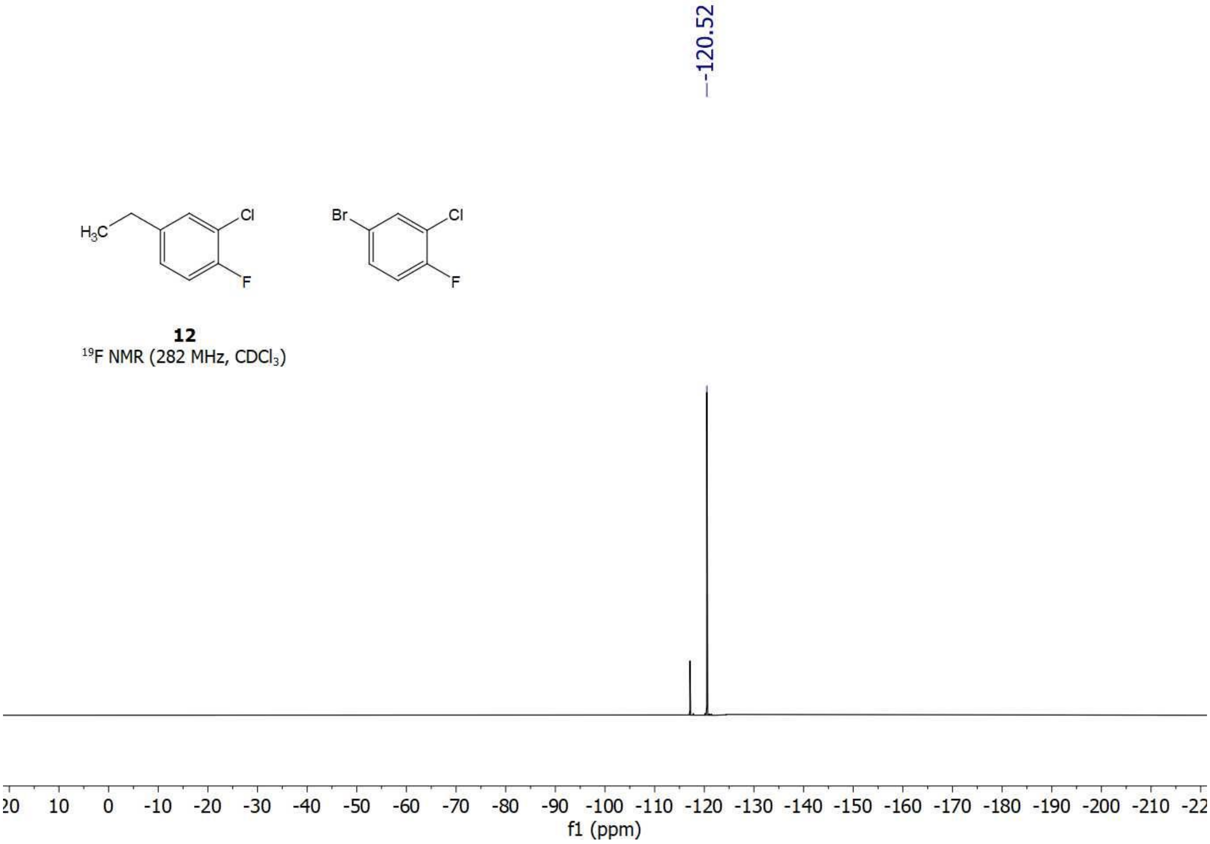

12

^{19}F NMR (282 MHz, $CDCl_3$)

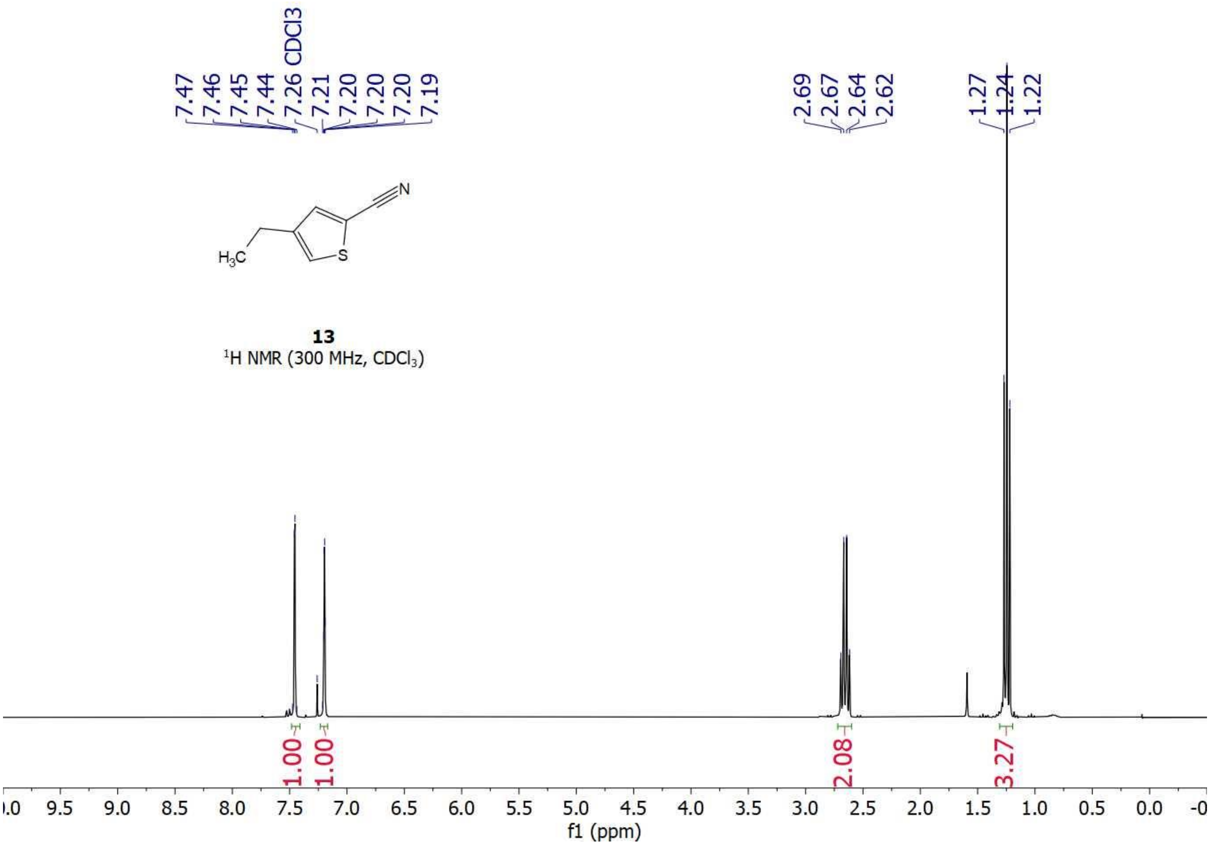

13
^{1}H NMR (300 MHz, $CDCl_3$)

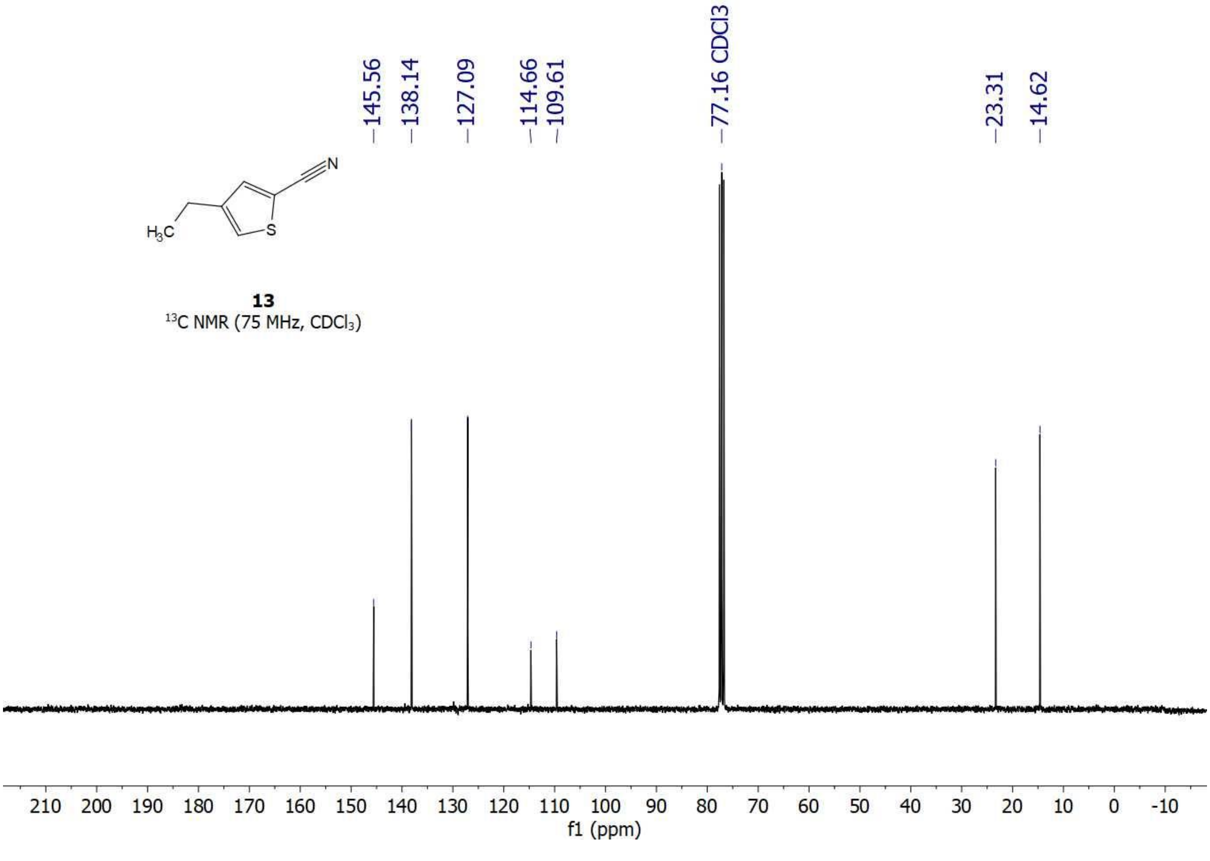

13
^{13}C NMR (75 MHz, $CDCl_3$)

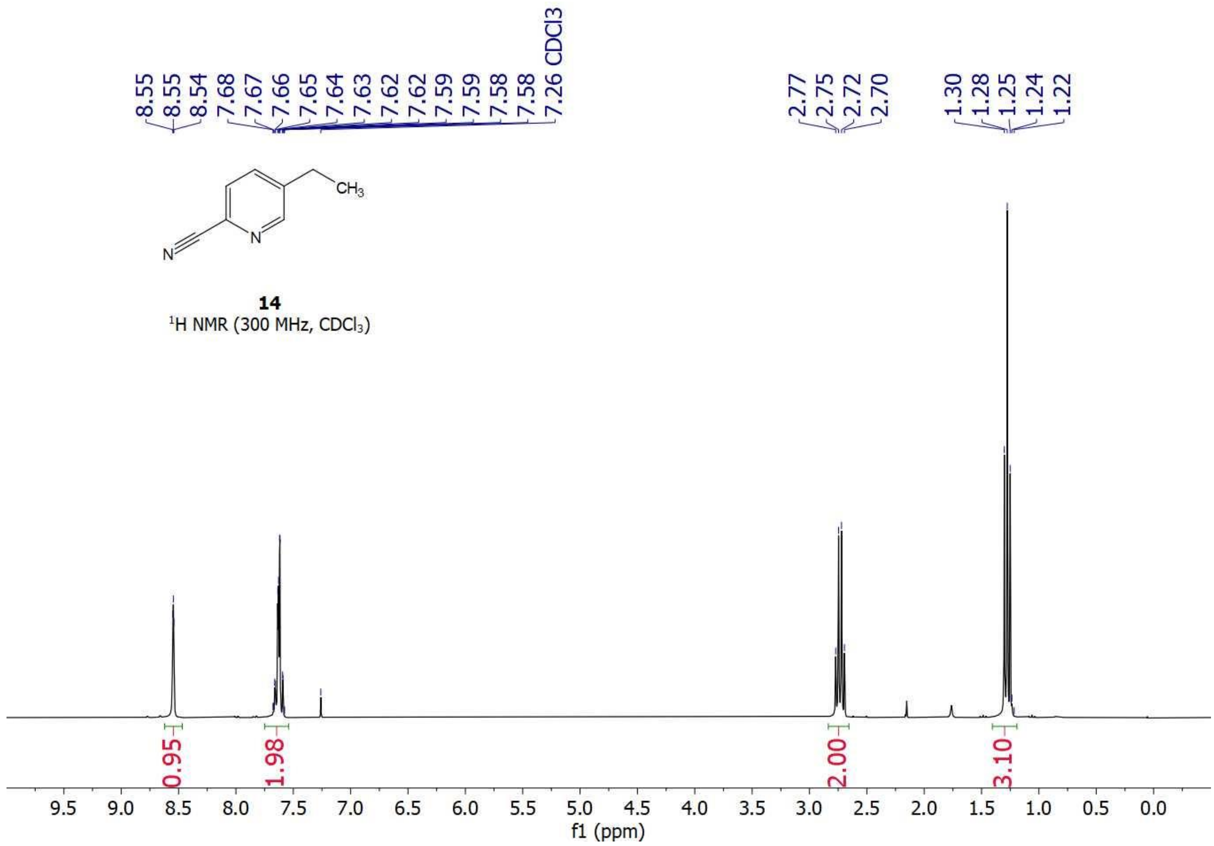
8.55
8.55
8.54
7.68
7.67
7.66
7.65
7.64
7.63
7.62
7.62
7.59
7.59
7.58
7.58
7.26 CDCl3
2.77
2.75
2.72
2.70
1.30
1.28
1.25
1.24
1.22
CH3
N
N
14
1H NMR (300 MHz, CDCl3)
0.95
1.98
2.00
3.10
9.5 9.0 8.5 8.0 7.5 7.0 6.5 6.0 5.5 5.0 4.5 4.0 3.5 3.0 2.5 2.0 1.5 1.0 0.5 0.0
f1 (ppm)

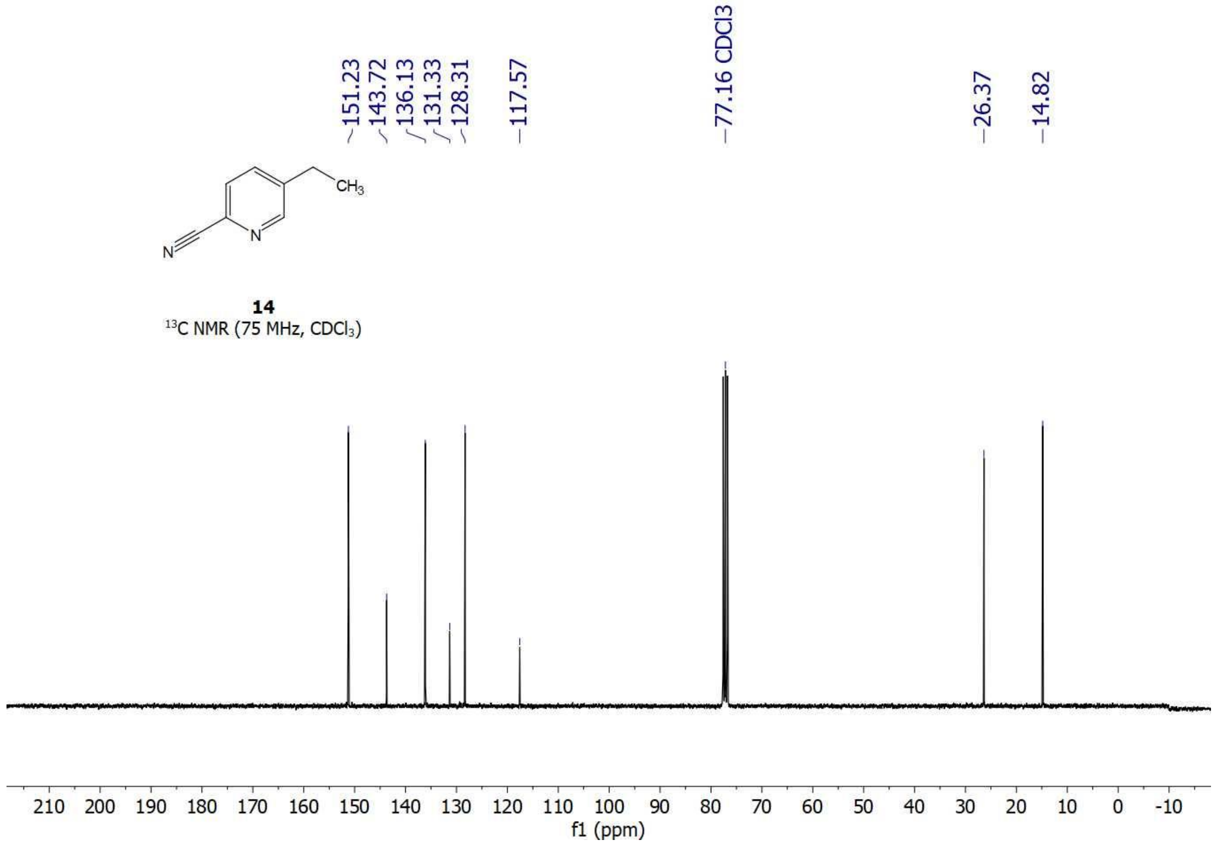
151.23
143.72
136.13
131.33
128.31
117.57
77.16 CDCl3
26.37
14.82
CH3
N
N
14
13C NMR (75 MHz, CDCl3)
210 200 190 180 170 160 150 140 130 120 110 100 90 80 70 60 50 40 30 20 10 0 -10
f1 (ppm)

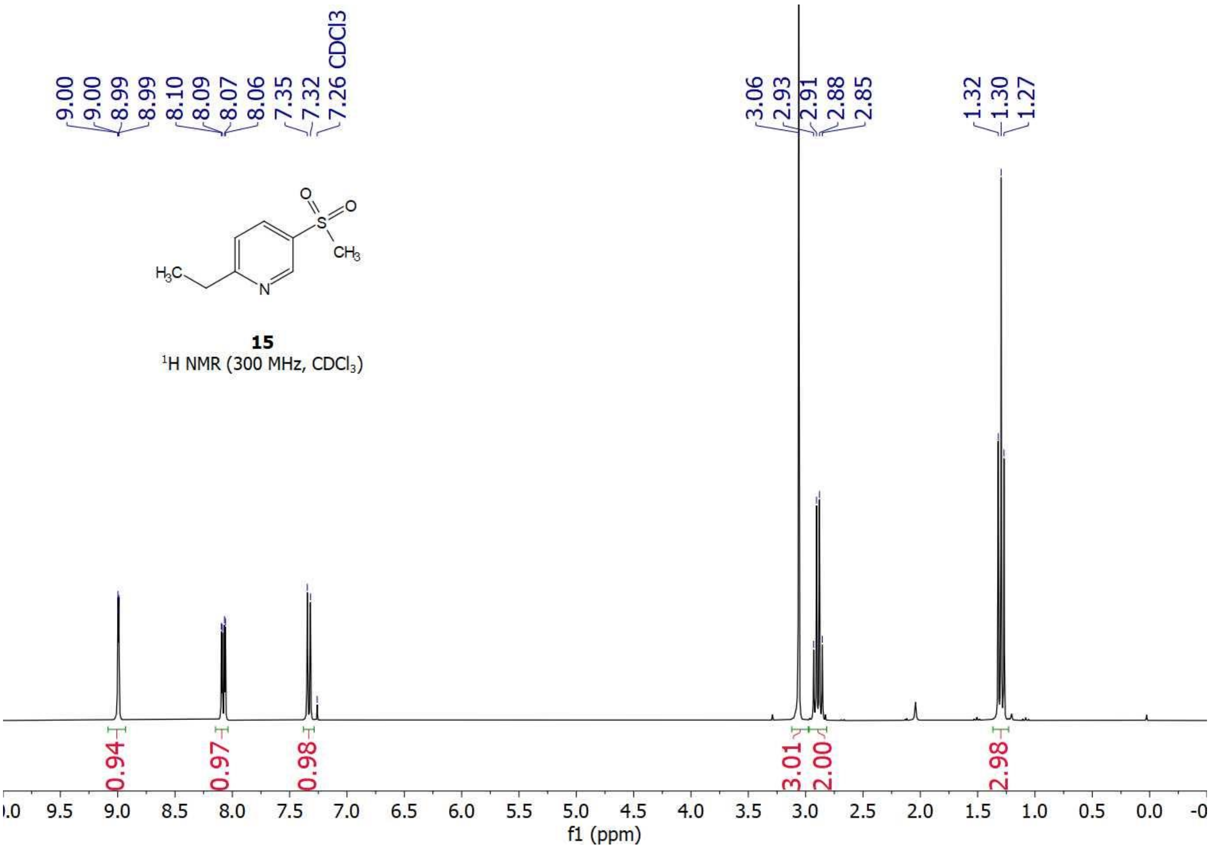
9.00
9.00
8.99
8.99
8.10
8.09
8.07
8.06
7.35
7.32
7.26 CDCl3
3.06
2.93
2.91
2.88
2.85
1.32
1.30
1.27
O
S
O
CH3
H3C
N
15
1H NMR (300 MHz, CDCl3)
0.94
0.97
0.98
3.01
2.00
2.98
f1 (ppm)

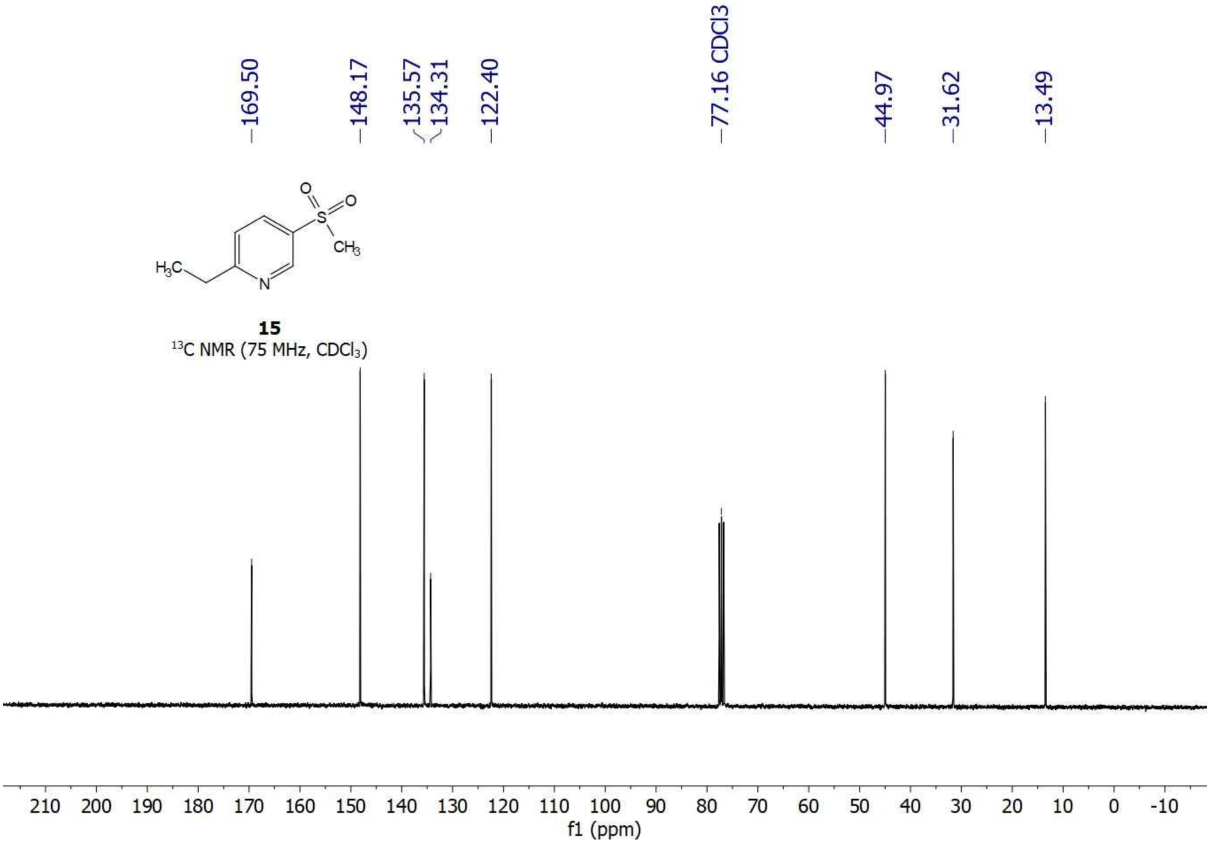
169.50
148.17
135.57
134.31
122.40
77.16 CDCl3
44.97
31.62
13.49
O
S
O
CH3
H3C
N
15
13C NMR (75 MHz, CDCl3)
f1 (ppm)

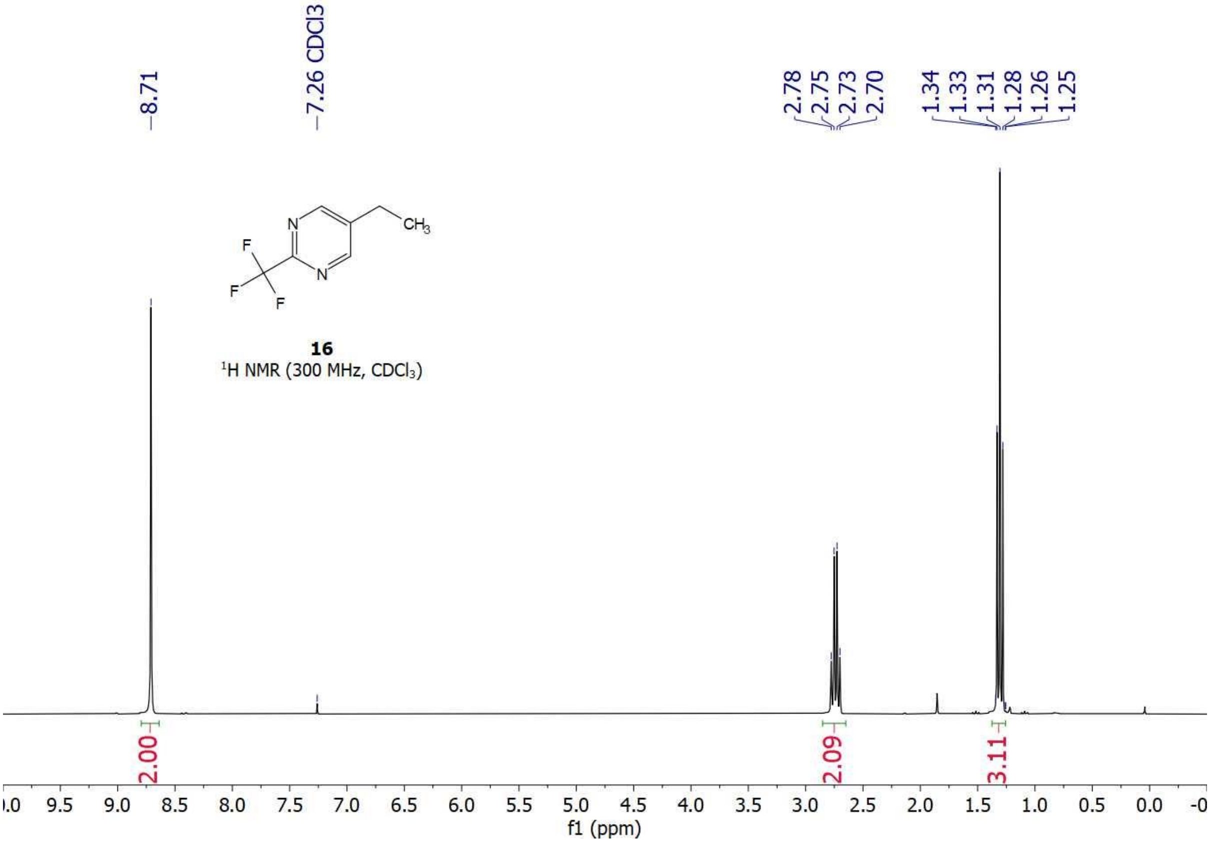
8.71
7.26 CDCl3
2.78
2.75
2.73
2.70
1.34
1.33
1.31
1.28
1.26
1.25
N
N
CH3
F
F
F
16
1H NMR (300 MHz, CDCl3)
2.00
2.09
3.11
f1 (ppm)

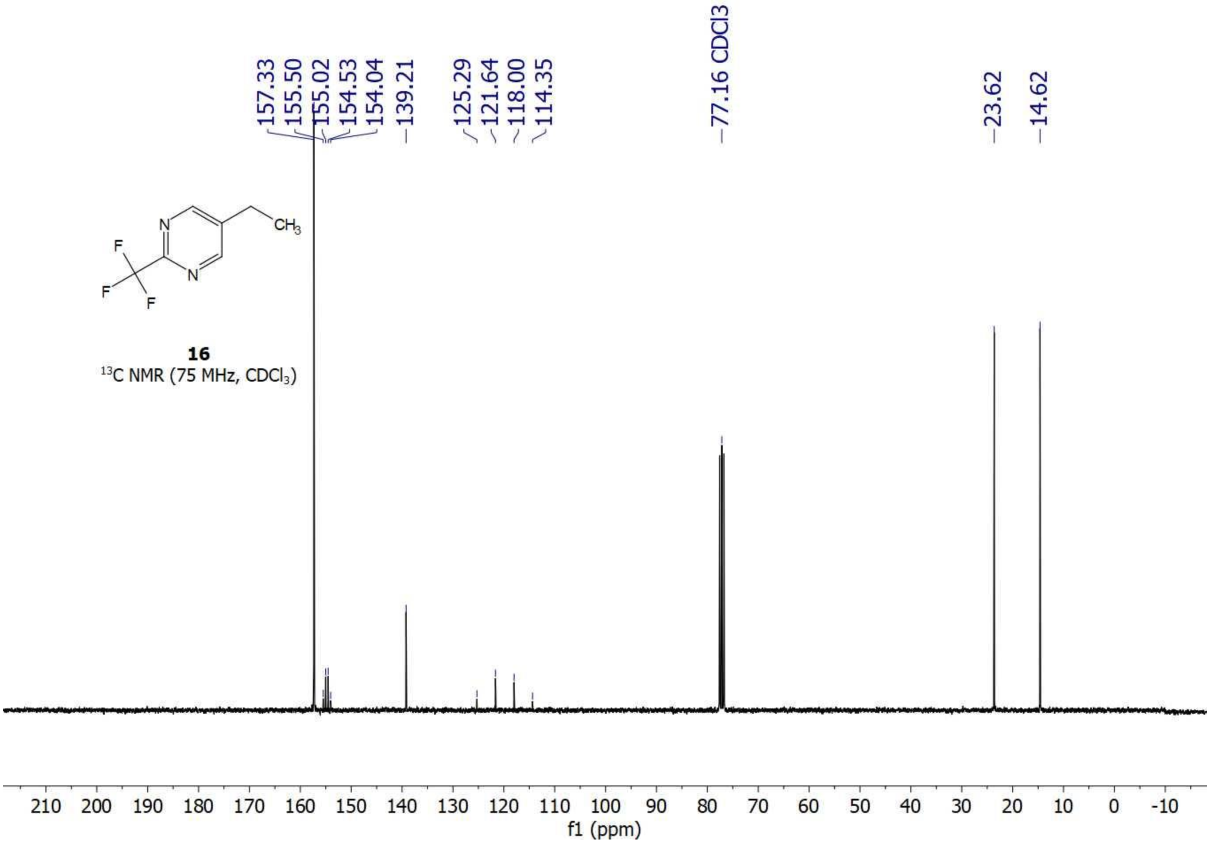
157.33
155.50
155.02
154.53
154.04
139.21
125.29
121.64
118.00
114.35
77.16 CDCl3
23.62
14.62
N
N
CH3
F
F
F
16
13C NMR (75 MHz, CDCl3)
f1 (ppm)

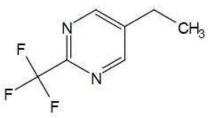

16
^{19}F NMR (282 MHz, $CDCl_3$)

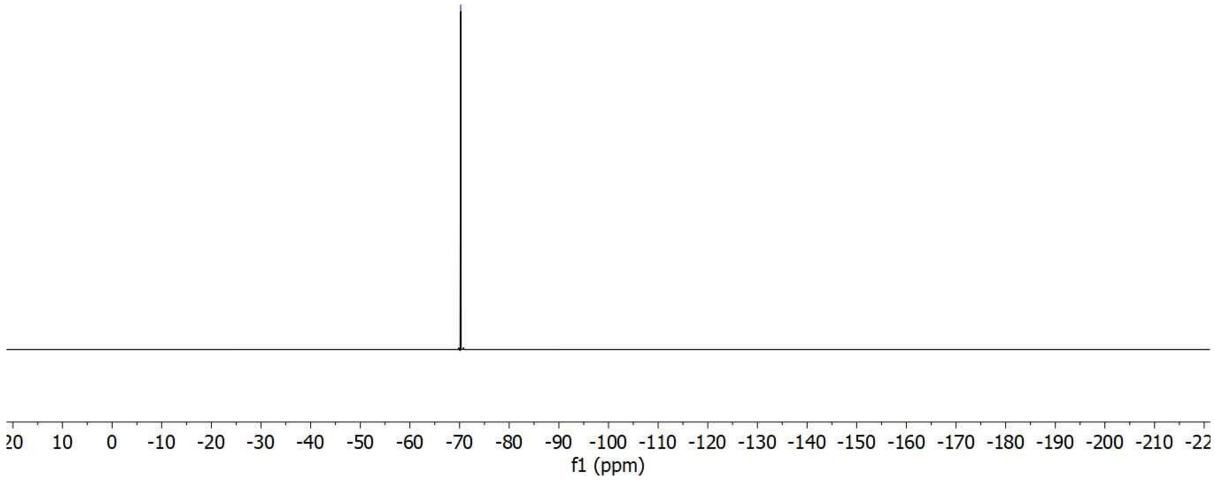

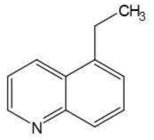

17
^{1}H NMR (400 MHz, $CDCl_3$)

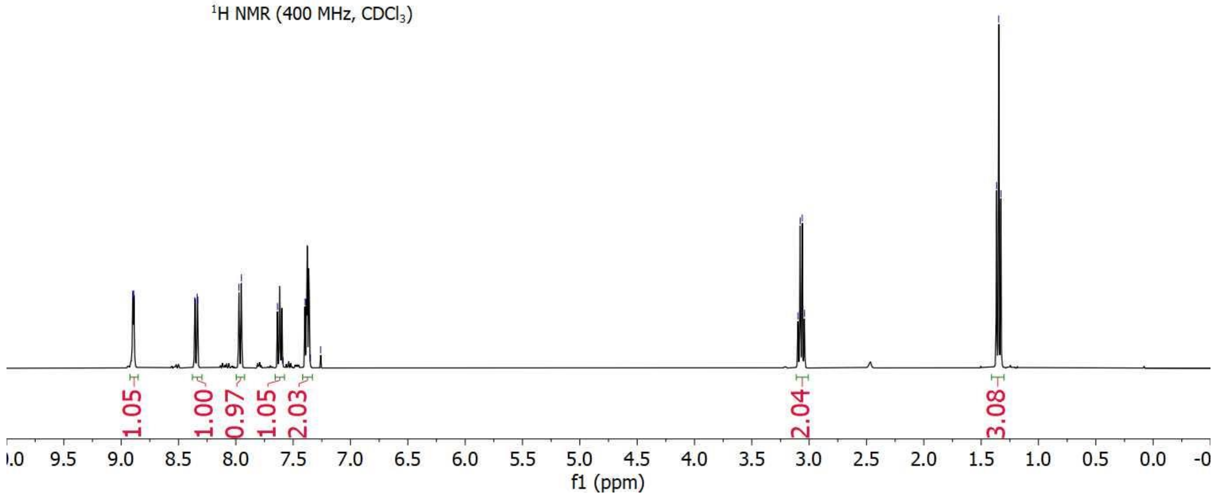

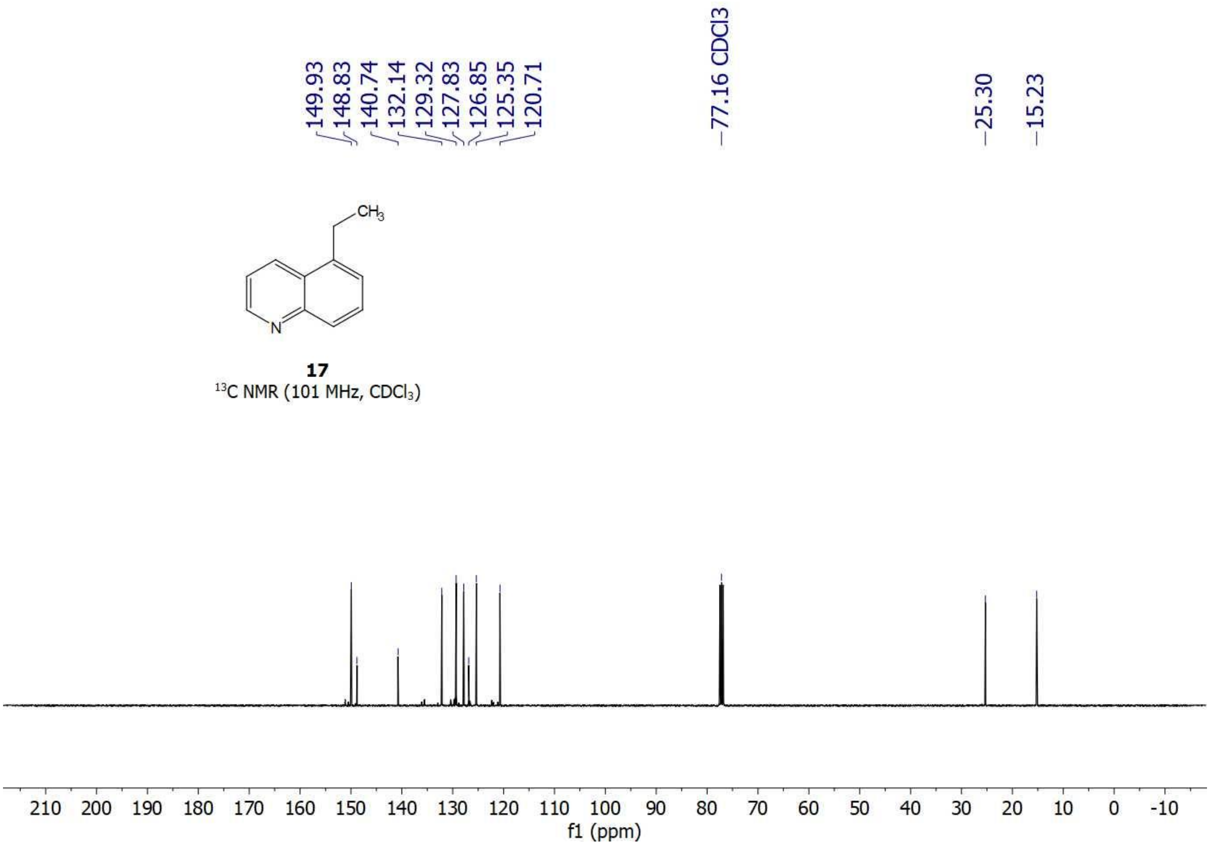
149.93
148.83
140.74
132.14
129.32
127.83
126.85
125.35
120.71
77.16 CDCl3
25.30
15.23
CH3
N
17
13C NMR (101 MHz, CDCl3)
210 200 190 180 170 160 150 140 130 120 110 100 90 80 70 60 50 40 30 20 10 0 -10
f1 (ppm)

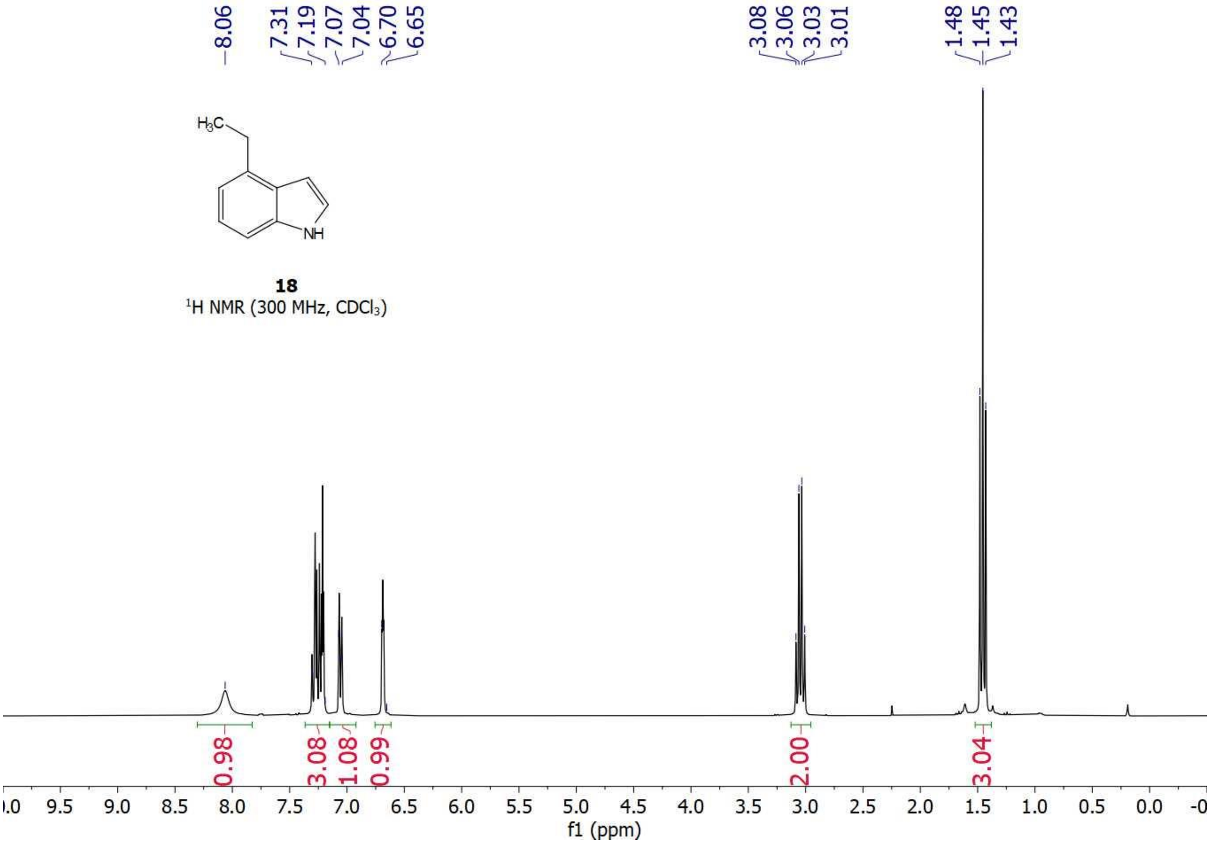
8.06
7.31
7.19
7.07
7.04
6.70
6.65
3.08
3.06
3.03
3.01
1.48
1.45
1.43
H3C
NH
18
1H NMR (300 MHz, CDCl3)
0.98
3.08
1.08
0.99
2.00
3.04
9.5 9.0 8.5 8.0 7.5 7.0 6.5 6.0 5.5 5.0 4.5 4.0 3.5 3.0 2.5 2.0 1.5 1.0 0.5 0.0
f1 (ppm)

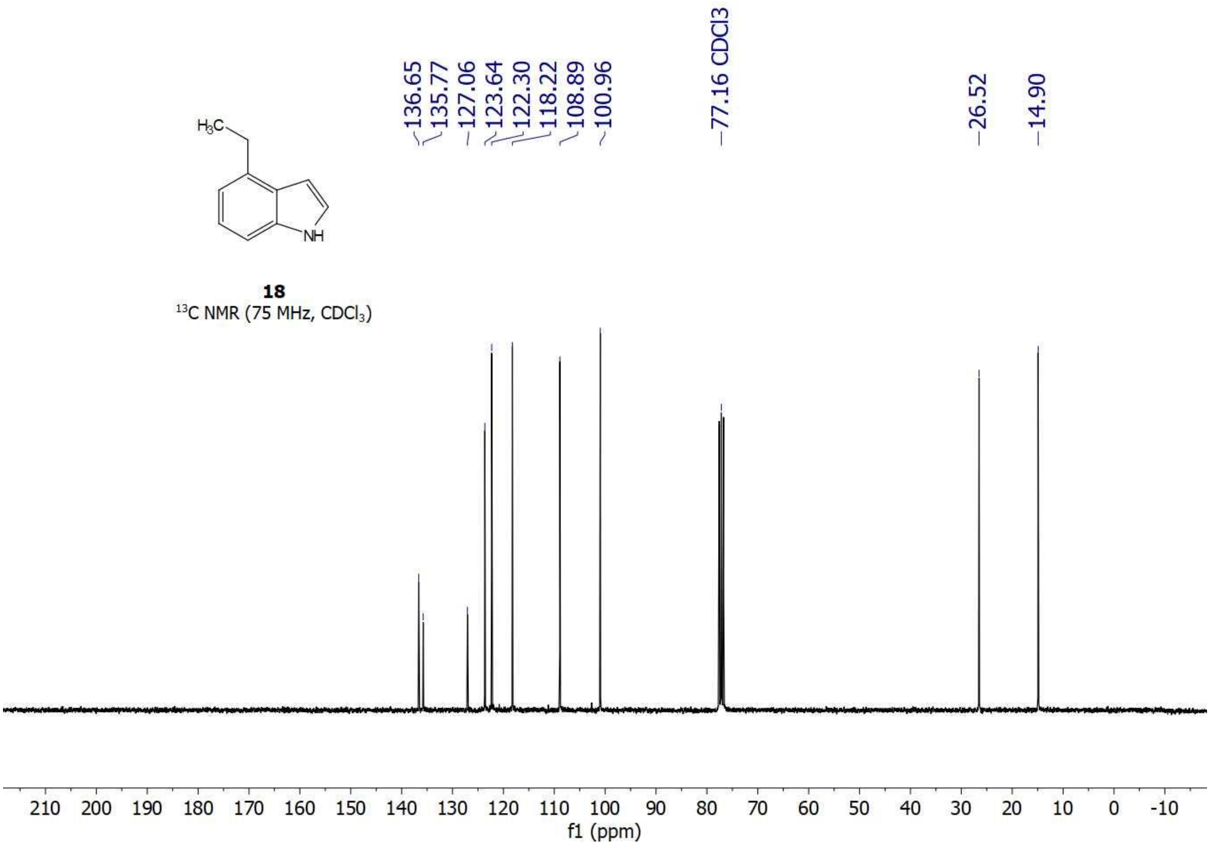

18
^{13}C NMR (75 MHz, $CDCl_3$)

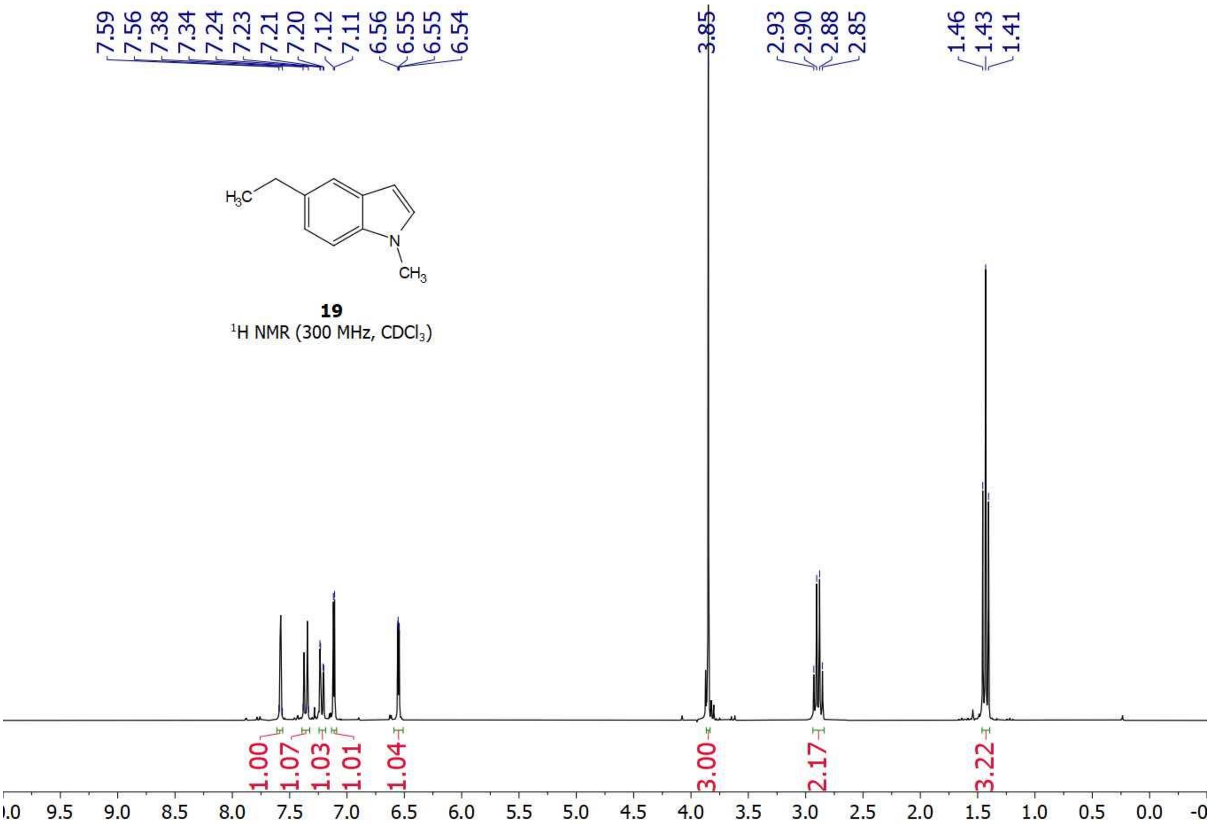

19
^{1}H NMR (300 MHz, $CDCl_3$)

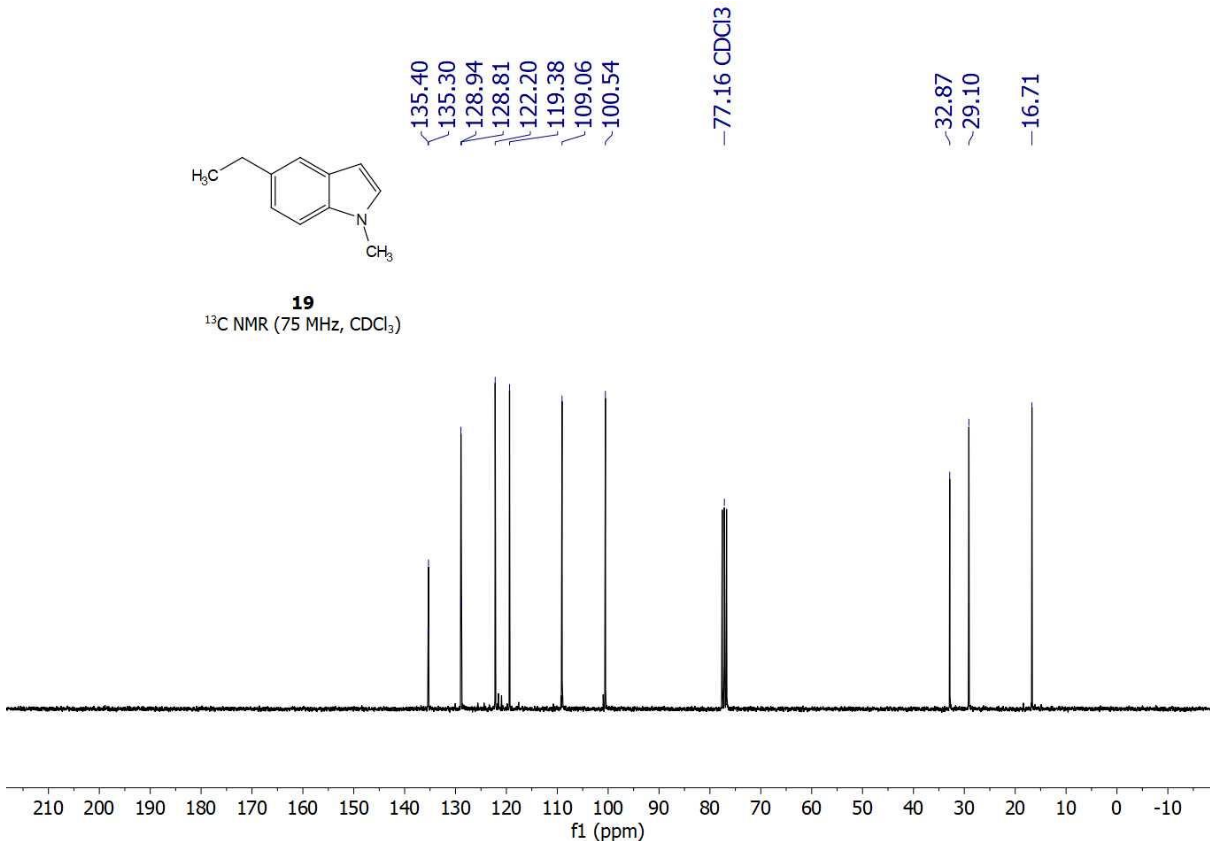
135.40
135.30
128.94
128.81
122.20
119.38
109.06
100.54
77.16 CDCl3
32.87
29.10
16.71
H_3C
N
CH_3
19
^{13}C NMR (75 MHz, $CDCl_3$)
210 200 190 180 170 160 150 140 130 120 110 100 90 80 70 60 50 40 30 20 10 0 -10
f1 (ppm)

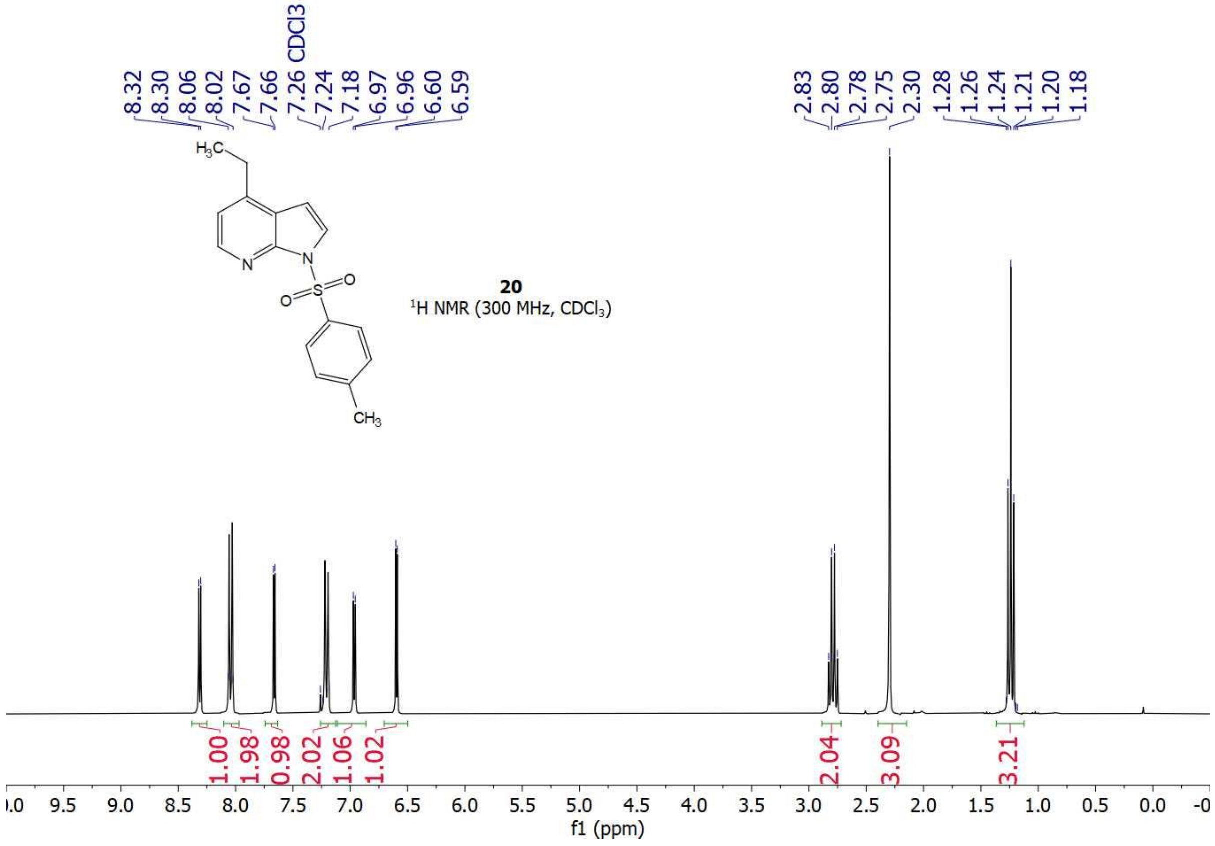
8.32
8.30
8.06
8.02
7.67
7.66
7.26 CDCl3
7.24
7.18
6.97
6.96
6.60
6.59
2.83
2.80
2.78
2.75
2.30
1.28
1.26
1.24
1.21
1.20
1.18
H_3C
N
N
S
O
O
CH_3
20
^{1}H NMR (300 MHz, $CDCl_3$)
1.00
1.98
0.98
2.02
1.06
1.02
2.04
3.09
3.21
9.5 9.0 8.5 8.0 7.5 7.0 6.5 6.0 5.5 5.0 4.5 4.0 3.5 3.0 2.5 2.0 1.5 1.0 0.5 0.0
f1 (ppm)

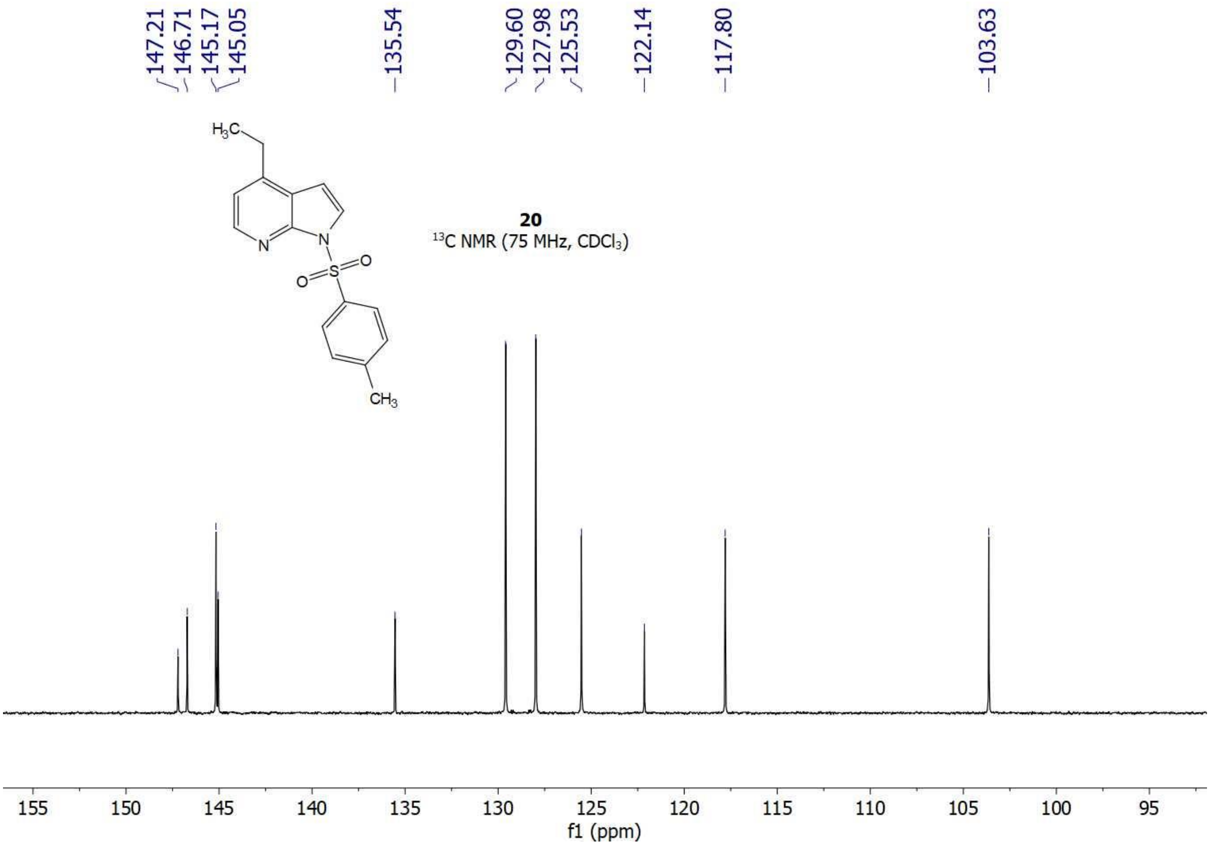
147.21
146.71
145.17
145.05
135.54
129.60
127.98
125.53
122.14
117.80
103.63
H3C
N
N
S
O
O
CH3
20
13C NMR (75 MHz, CDCl3)
155 150 145 140 135 130 125 120 115 110 105 100 95
f1 (ppm)

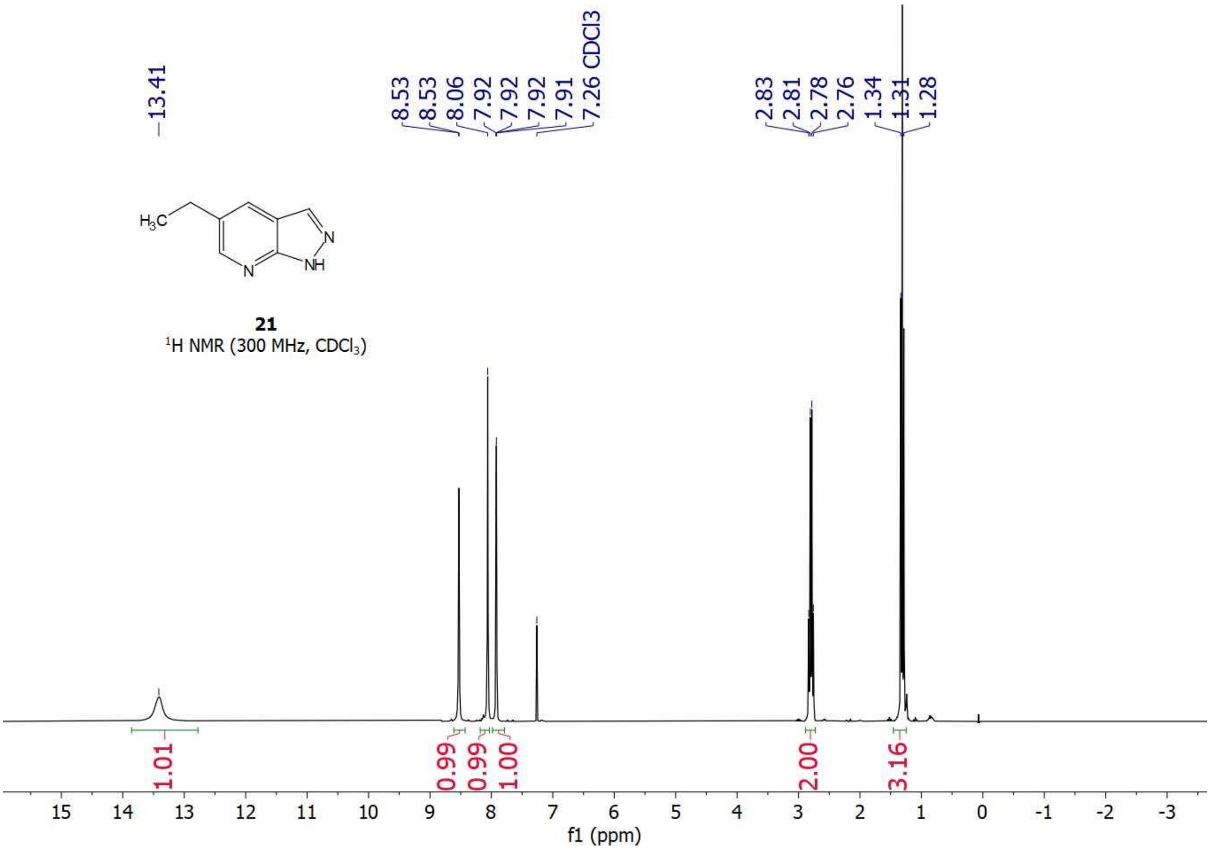
13.41
8.53
8.53
8.06
7.92
7.92
7.92
7.91
7.26 CDCl3
2.83
2.81
2.78
2.76
1.34
1.31
1.28
H3C
N
N
NH
21
1H NMR (300 MHz, CDCl3)
1.01
0.99
0.99
1.00
2.00
3.16
15 14 13 12 11 10 9 8 7 6 5 4 3 2 1 0 -1 -2 -3
f1 (ppm)

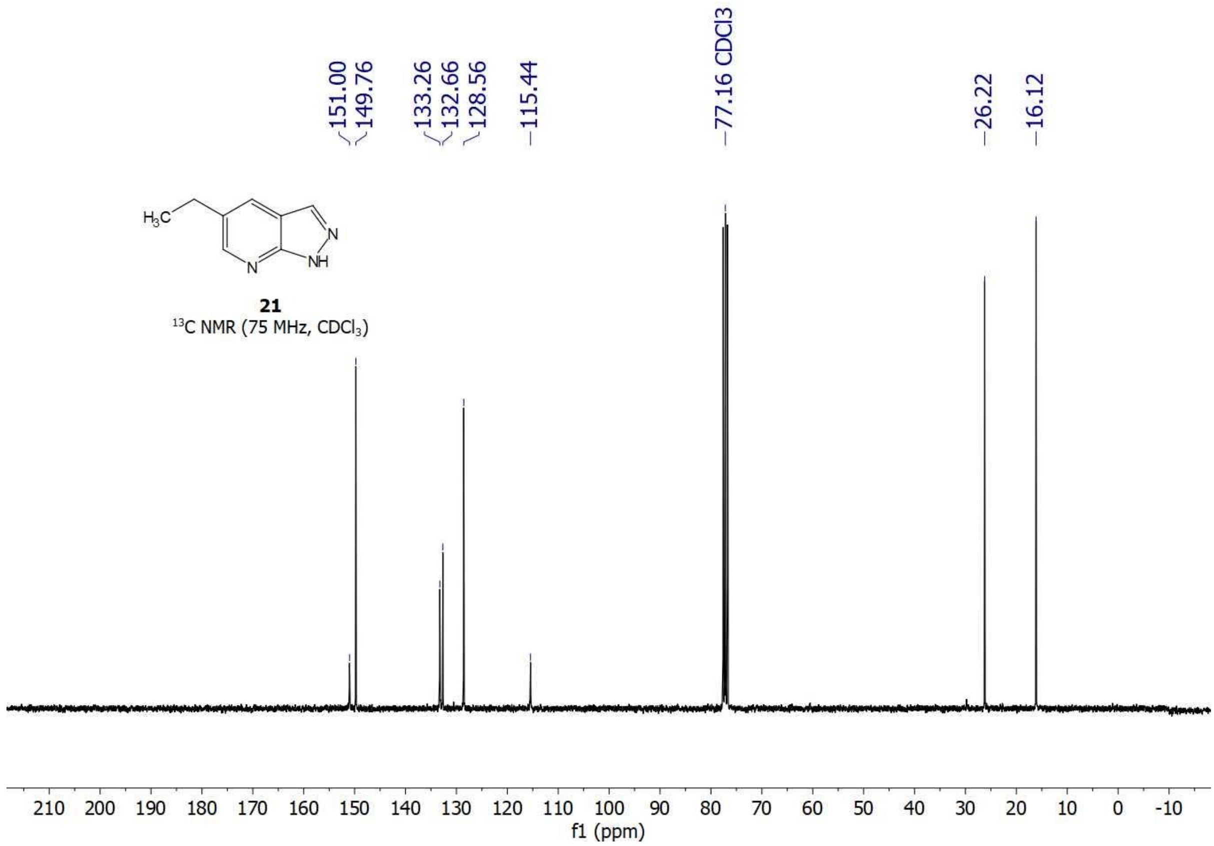
151.00
149.76
133.26
132.66
128.56
115.44
77.16 CDCl3
26.22
16.12
H3C
N
NH
21
13C NMR (75 MHz, CDCl3)
210 200 190 180 170 160 150 140 130 120 110 100 90 80 70 60 50 40 30 20 10 0 -10
f1 (ppm)

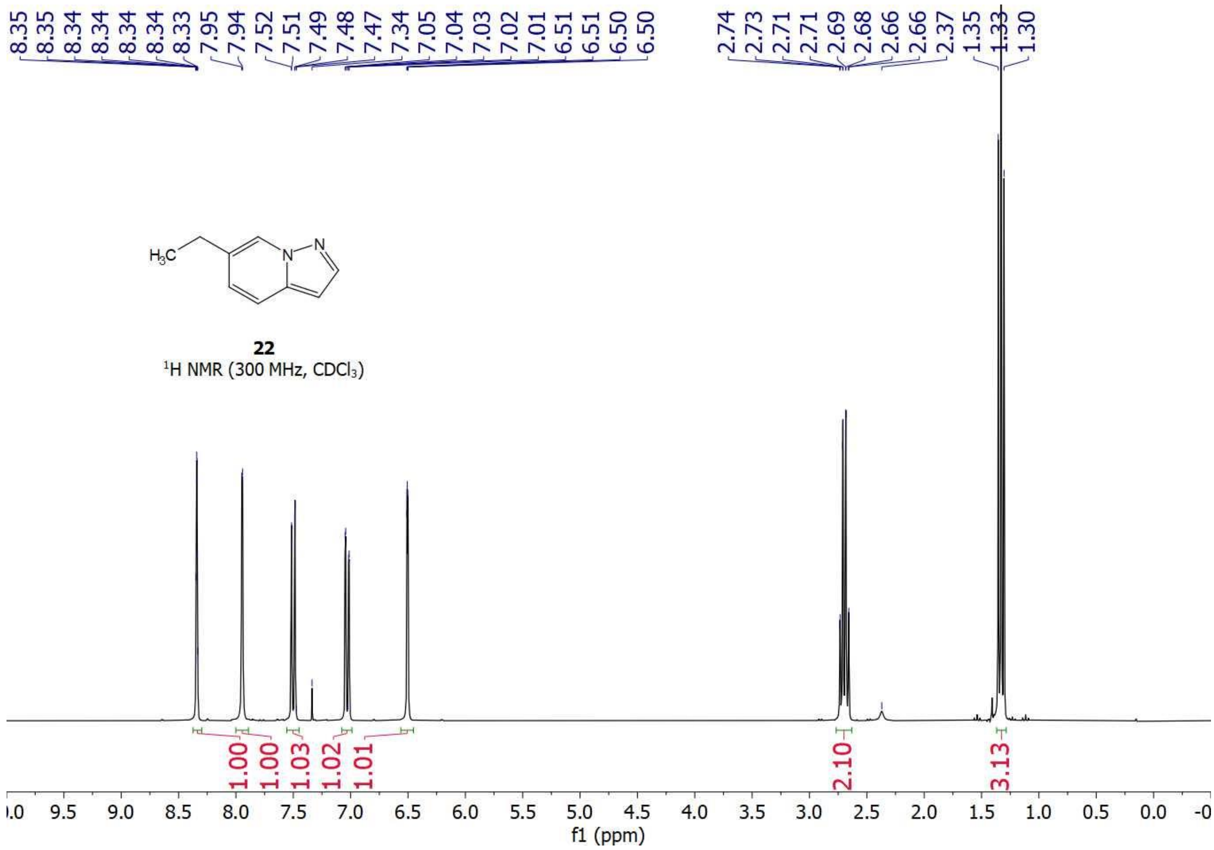
8.35 8.35 8.34 8.34 8.34 8.34 8.33 7.95 7.94 7.52 7.51 7.49 7.48 7.47 7.34 7.05 7.04 7.03 7.02 7.01 6.51 6.51 6.50 6.50
2.74 2.73 2.71 2.71 2.69 2.68 2.66 2.66 2.37 1.35 1.33 1.30
H3C
N
N
22
1H NMR (300 MHz, CDCl3)
1.00 1.00 1.03 1.02 1.01
2.10
3.13
9.5 9.0 8.5 8.0 7.5 7.0 6.5 6.0 5.5 5.0 4.5 4.0 3.5 3.0 2.5 2.0 1.5 1.0 0.5 0.0
f1 (ppm)

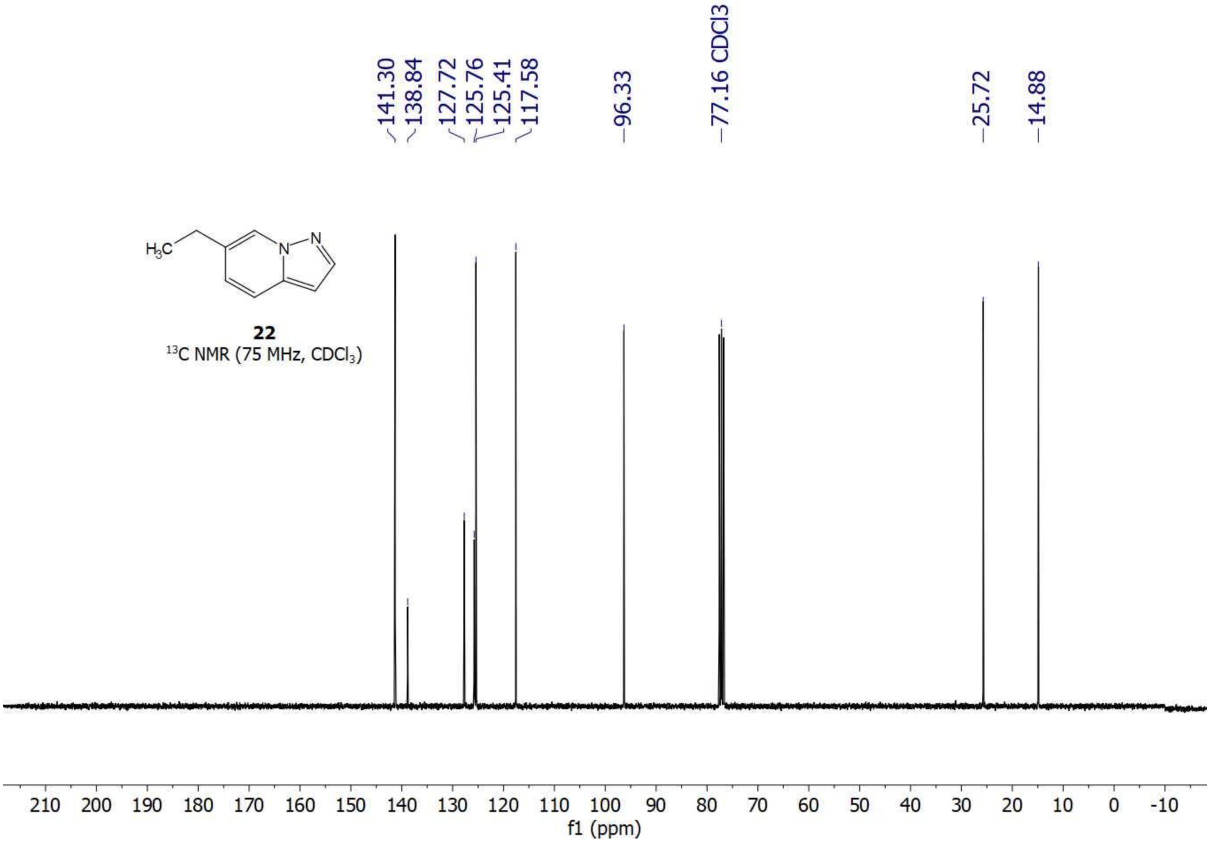

22
^{13}C NMR (75 MHz, $CDCl_3$)

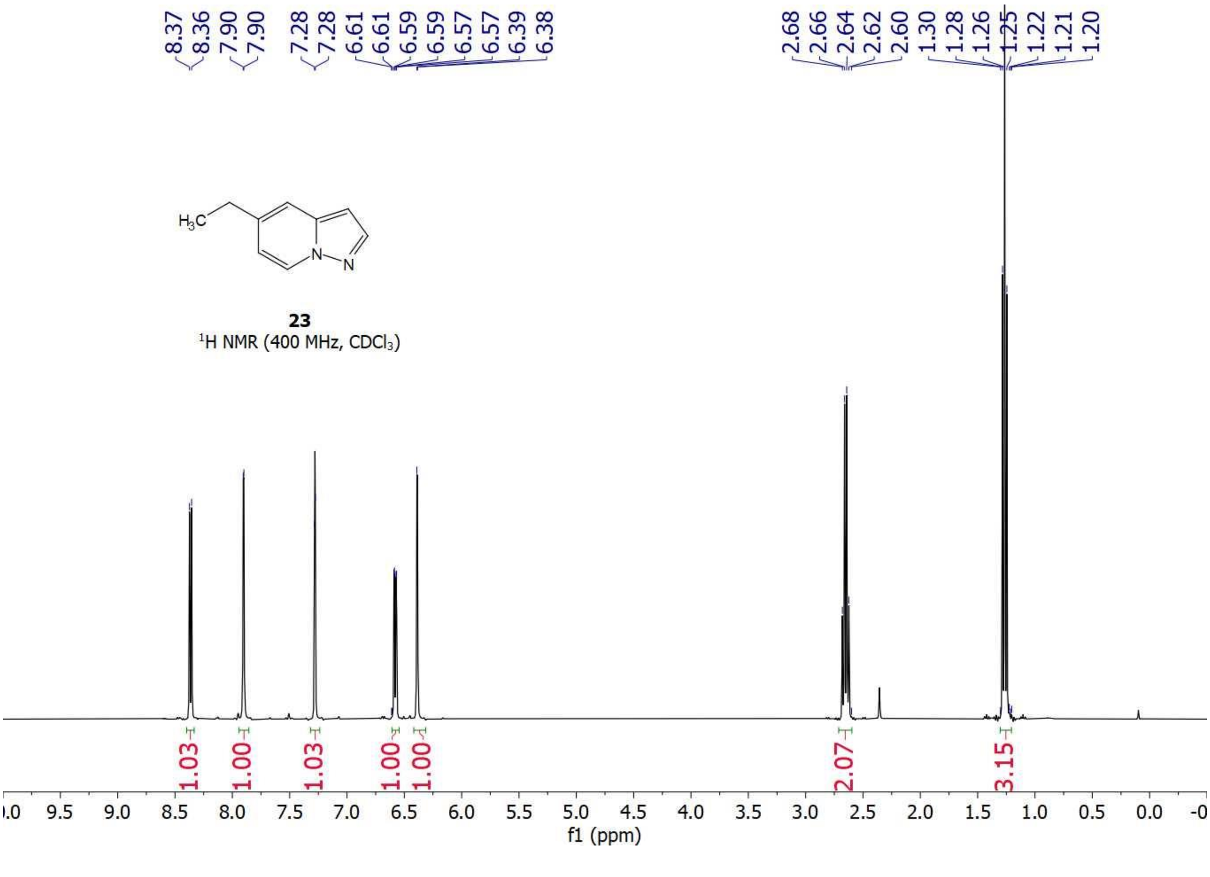

23
^{1}H NMR (400 MHz, $CDCl_3$)

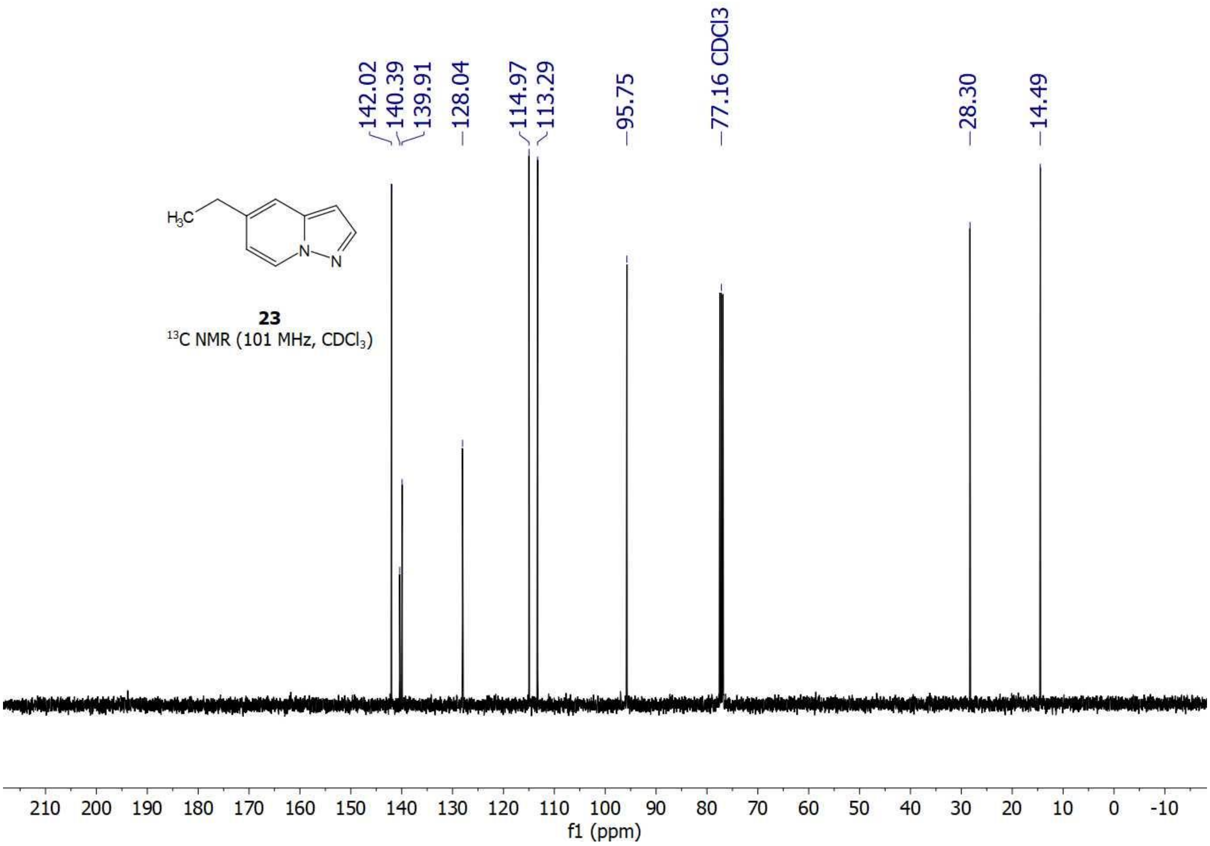
142.02
140.39
139.91
128.04
114.97
113.29
95.75
77.16 CDCl3
28.30
14.49
H_3C
N
N
23
^{13}C NMR (101 MHz, $CDCl_3$)
210 200 190 180 170 160 150 140 130 120 110 100 90 80 70 60 50 40 30 20 10 0 -10
f1 (ppm)

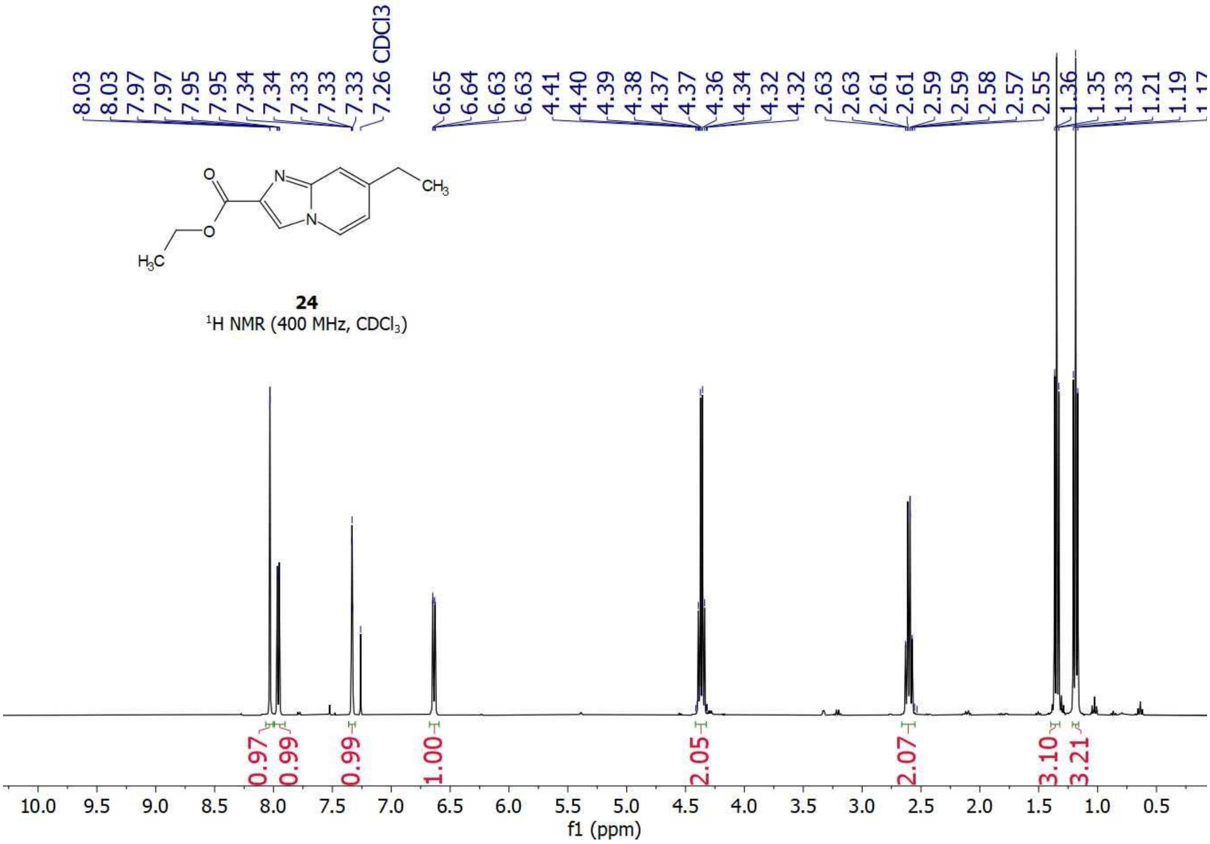
8.03
8.03
7.97
7.97
7.95
7.95
7.34
7.34
7.33
7.33
7.33
7.26 CDCl3
6.65
6.64
6.63
6.63
4.41
4.40
4.39
4.38
4.37
4.37
4.36
4.34
4.32
4.32
2.63
2.63
2.61
2.61
2.59
2.59
2.58
2.57
2.55
1.36
1.35
1.33
1.21
1.19
O
N
N
O
CH_3
H_3C
24
^1H NMR (400 MHz, $CDCl_3$)
0.97
0.99
0.99
1.00
2.05
2.07
3.10
3.21
10.0 9.5 9.0 8.5 8.0 7.5 7.0 6.5 6.0 5.5 5.0 4.5 4.0 3.5 3.0 2.5 2.0 1.5 1.0 0.5
f1 (ppm)

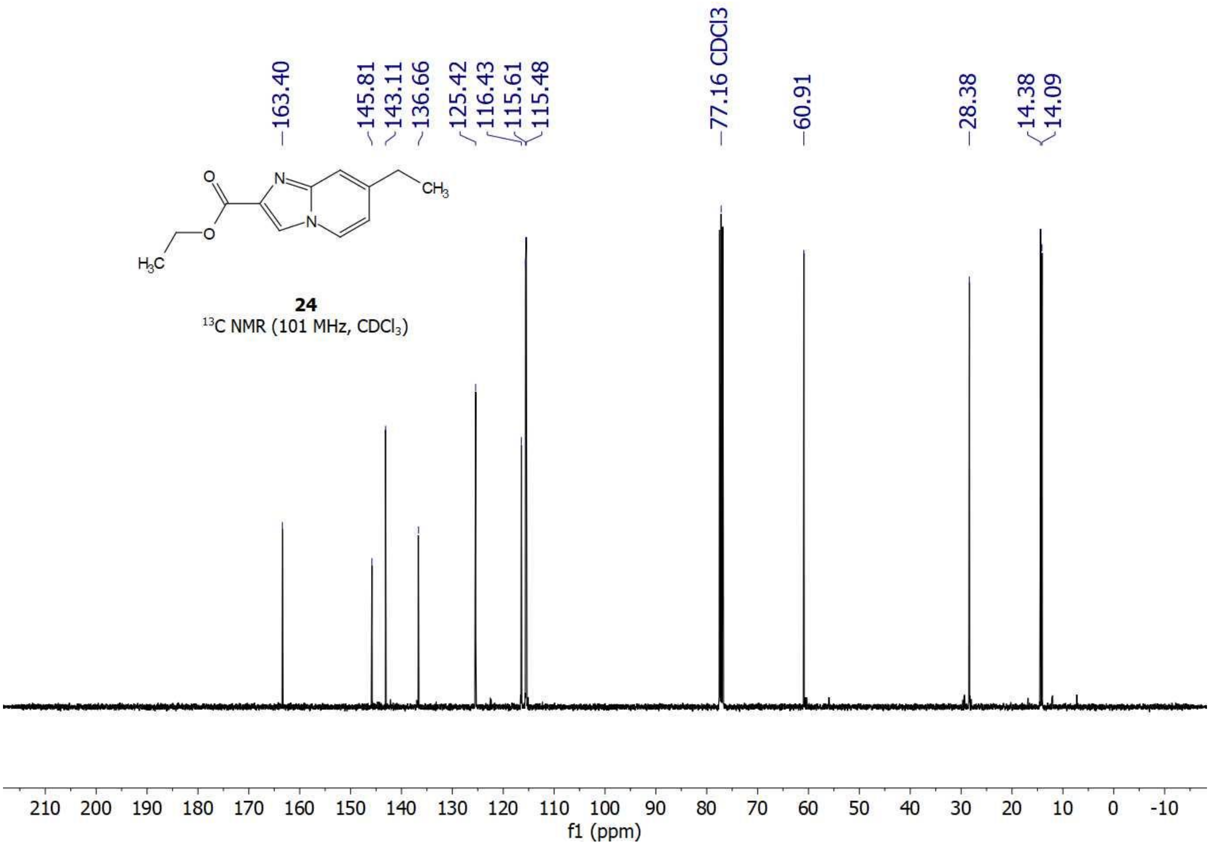

24
^{13}C NMR (101 MHz, $CDCl_3$)

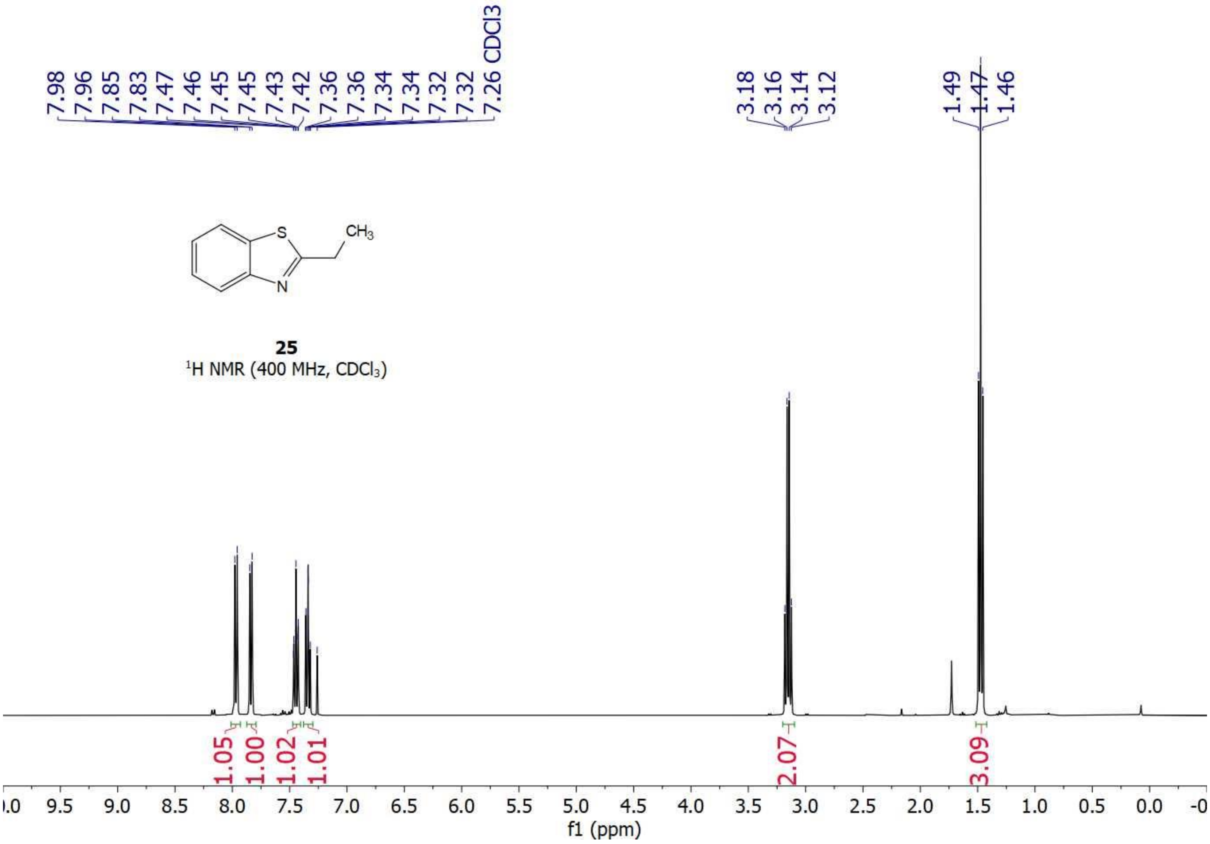

25
^{1}H NMR (400 MHz, $CDCl_3$)

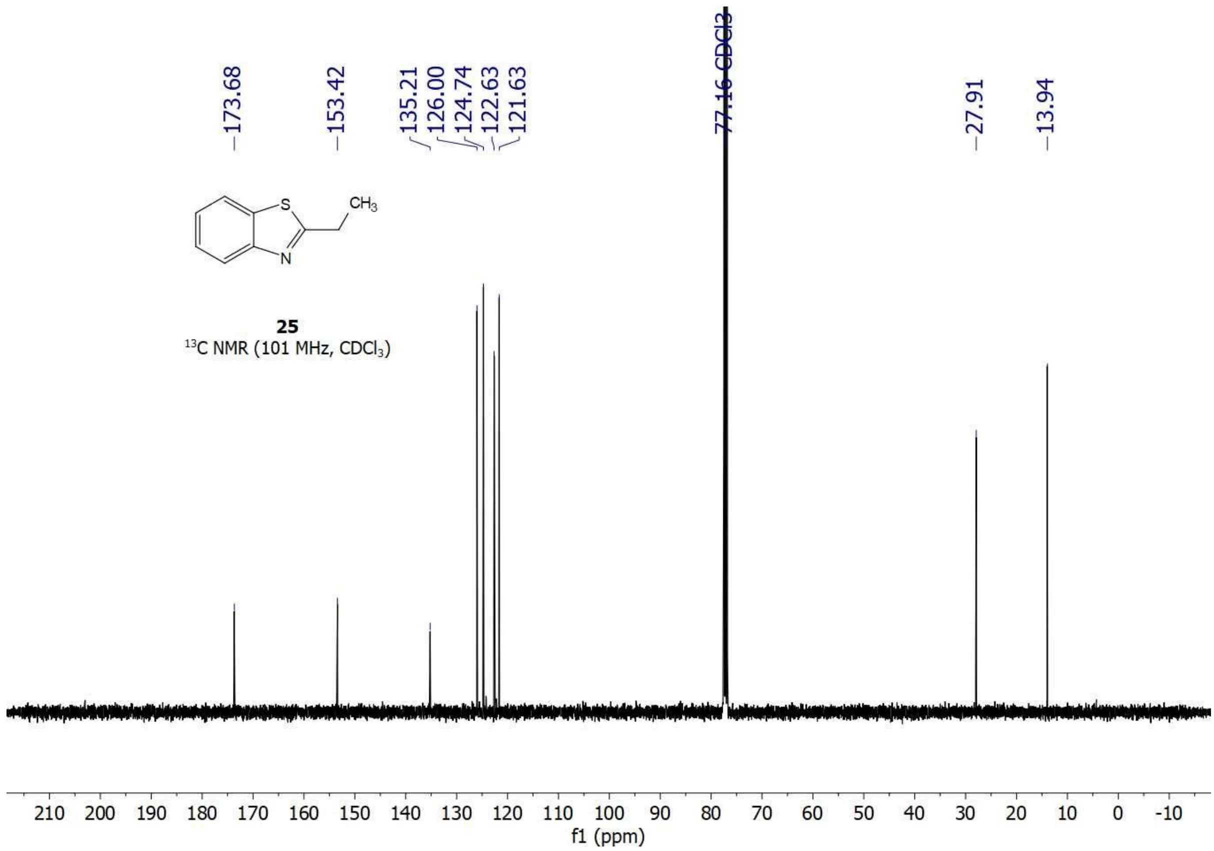
173.68
153.42
135.21
126.00
124.74
122.63
121.63
77.16 CDCl3
27.91
13.94
S
N
CH3
25
13C NMR (101 MHz, CDCl3)
210 200 190 180 170 160 150 140 130 120 110 100 90 80 70 60 50 40 30 20 10 0 -10
f1 (ppm)

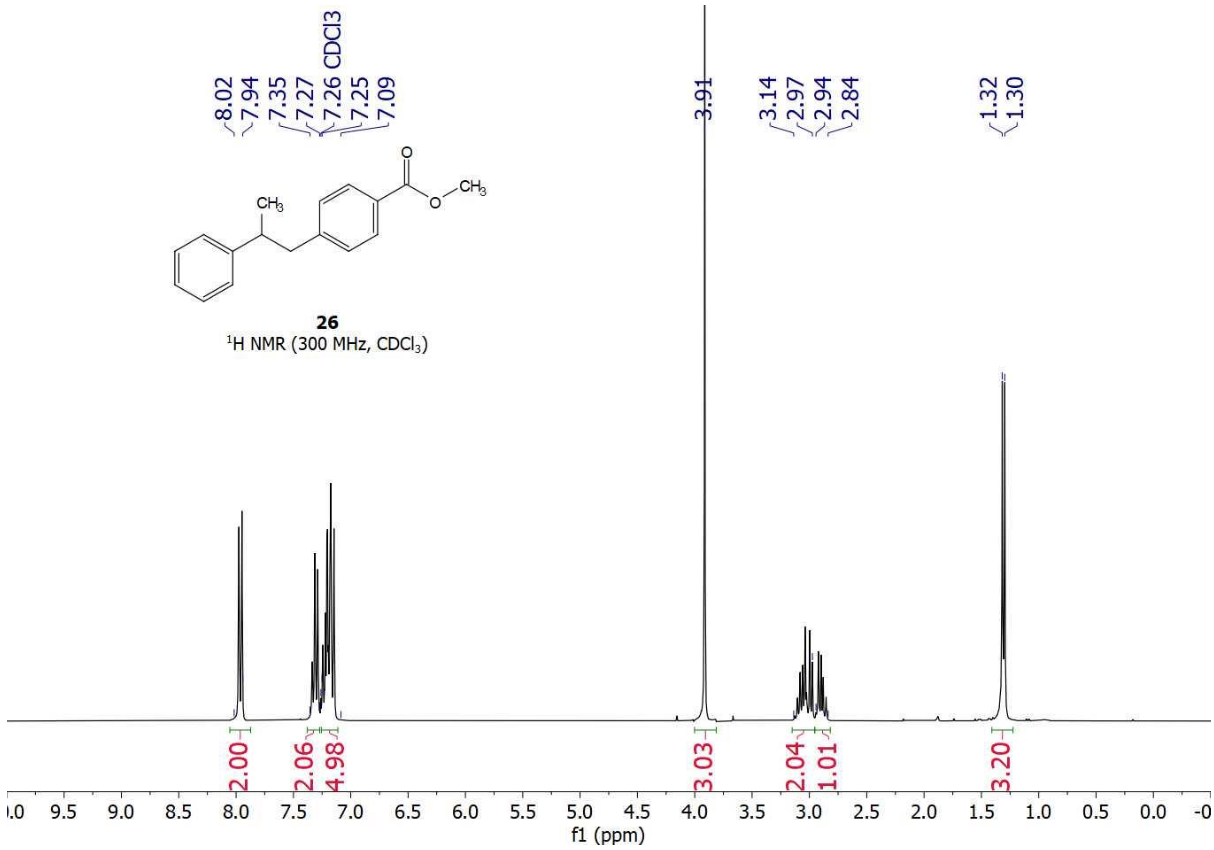
8.02
7.94
7.35
7.27
7.26 CDCl3
7.25
7.09
3.91
3.14
2.97
2.94
2.84
1.32
1.30
O
CH3
26
1H NMR (300 MHz, CDCl3)
2.00
2.06
4.98
3.03
2.04
1.01
3.20
9.5 9.0 8.5 8.0 7.5 7.0 6.5 6.0 5.5 5.0 4.5 4.0 3.5 3.0 2.5 2.0 1.5 1.0 0.5 0.0
f1 (ppm)

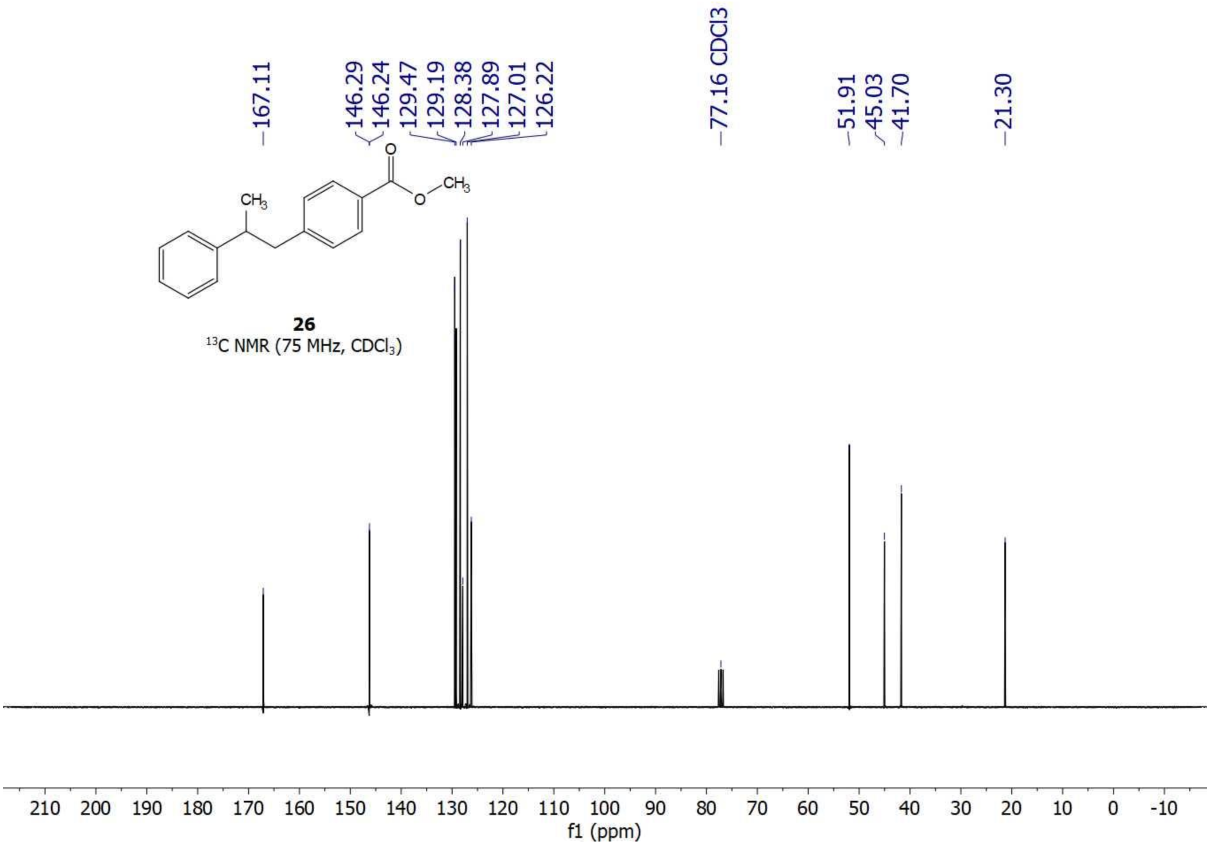

167.11
146.29
146.24
129.47
129.19
128.38
127.89
127.01
126.22
77.16 CDCl3
51.91
45.03
41.70
21.30
CH3
O
O
CH3
26
^{13}C NMR (75 MHz, $CDCl_3$)
210 200 190 180 170 160 150 140 130 120 110 100 90 80 70 60 50 40 30 20 10 0 -10
f1 (ppm)

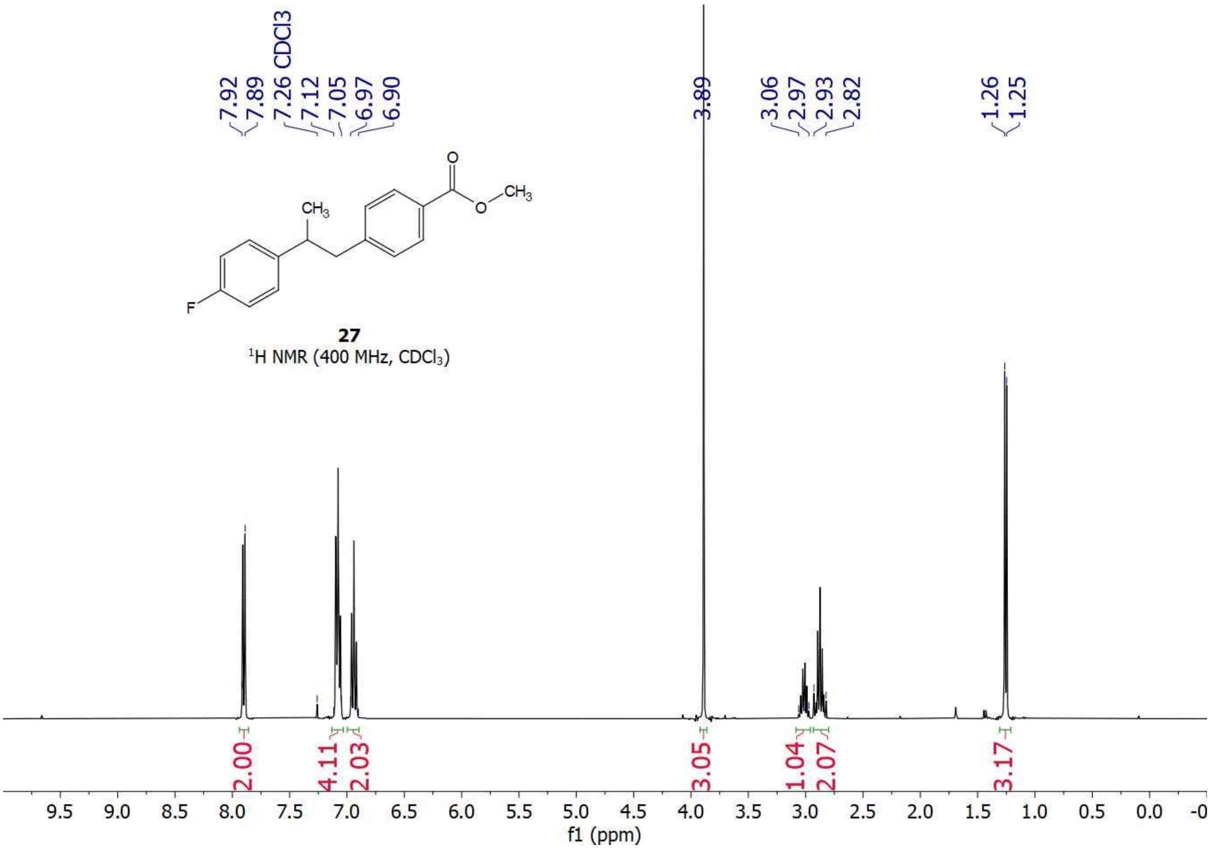

7.92
7.89
7.26 CDCl3
7.12
7.05
6.97
6.90
3.89
3.06
2.97
2.93
2.82
1.26
1.25
O
CH3
O
CH3
F
27
^{1}H NMR (400 MHz, $CDCl_3$)
2.00
4.11
2.03
3.05
1.04
2.07
3.17
9.5 9.0 8.5 8.0 7.5 7.0 6.5 6.0 5.5 5.0 4.5 4.0 3.5 3.0 2.5 2.0 1.5 1.0 0.5 0.0 -0
f1 (ppm)

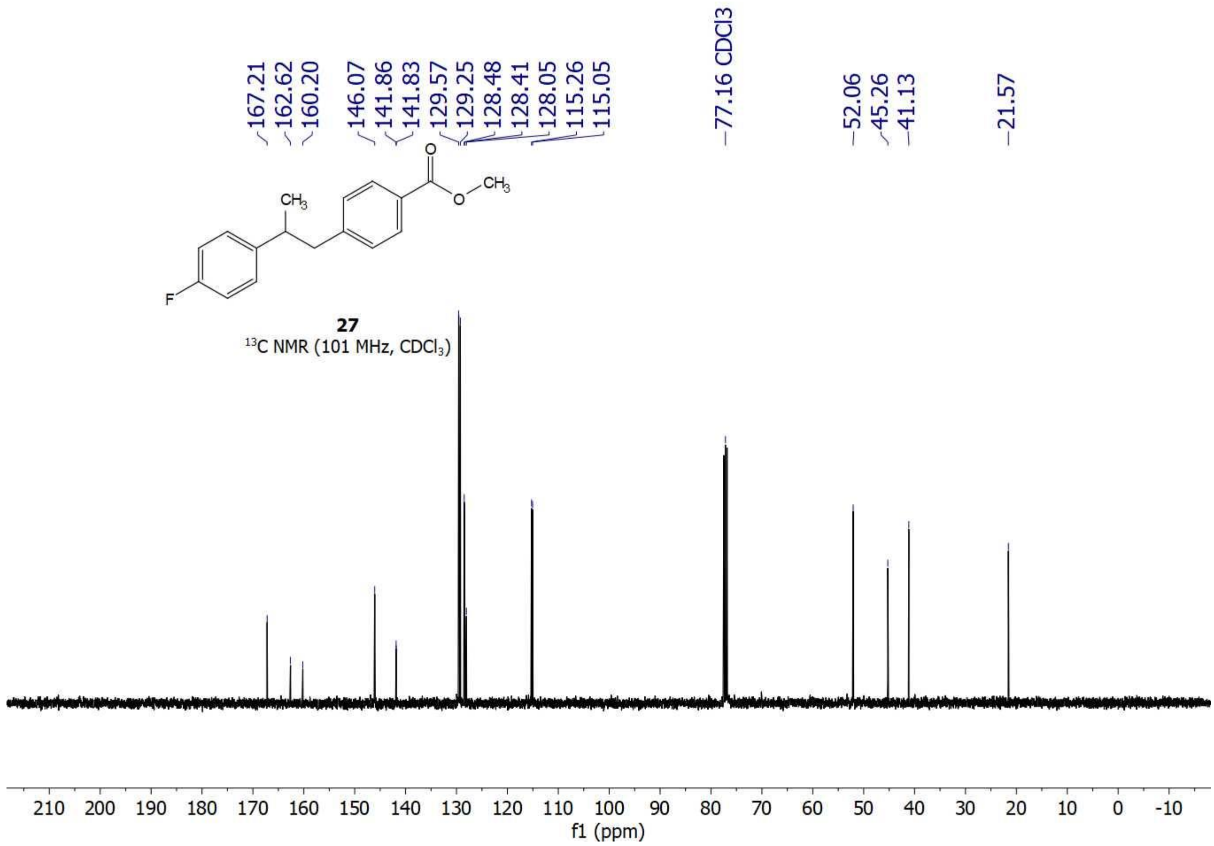
167.21
162.62
160.20
146.07
141.86
141.83
129.57
129.25
128.48
128.41
128.05
115.26
115.05
77.16 CDCl3
52.06
45.26
41.13
21.57
CH3
O
O
CH3
F
27
13C NMR (101 MHz, CDCl3)
210 200 190 180 170 160 150 140 130 120 110 100 90 80 70 60 50 40 30 20 10 0 -10
f1 (ppm)

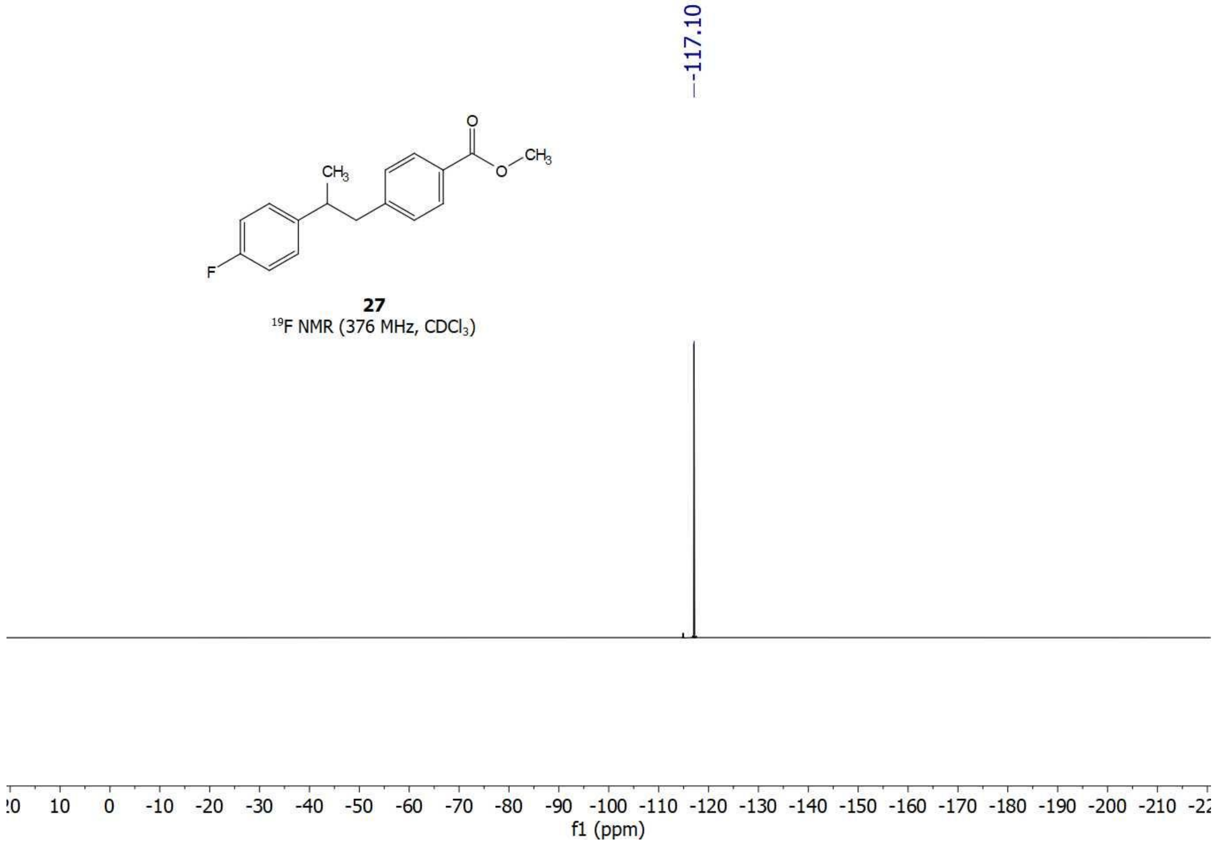
-117.10
CH3
O
O
CH3
F
27
19F NMR (376 MHz, CDCl3)
10 0 -10 -20 -30 -40 -50 -60 -70 -80 -90 -100 -110 -120 -130 -140 -150 -160 -170 -180 -190 -200 -210
f1 (ppm)

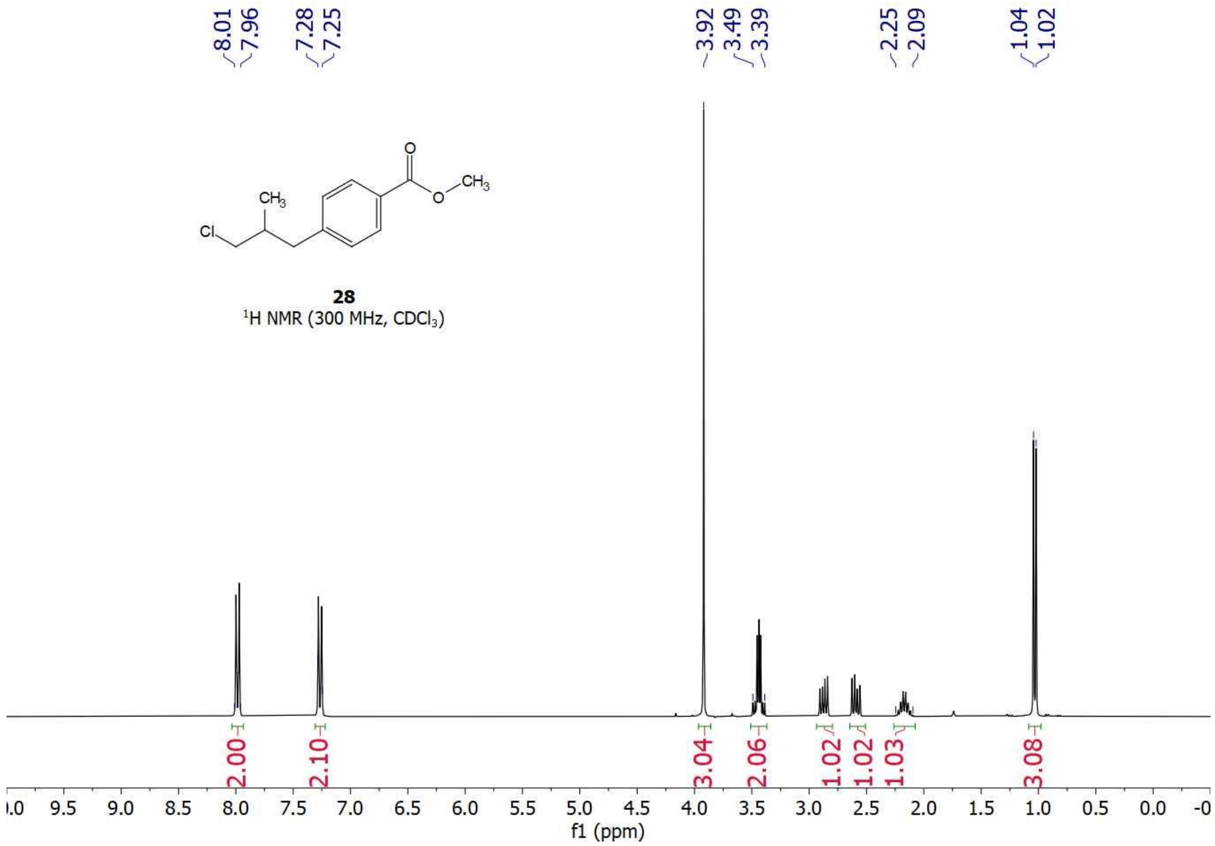

8.01
7.96
7.28
7.25
3.92
3.49
3.39
2.25
2.09
1.04
1.02
CH3
O
O
CH3
Cl
28
1H NMR (300 MHz, CDCl3)
2.00
2.10
3.04
2.06
1.02
1.02
1.03
3.08
f1 (ppm)

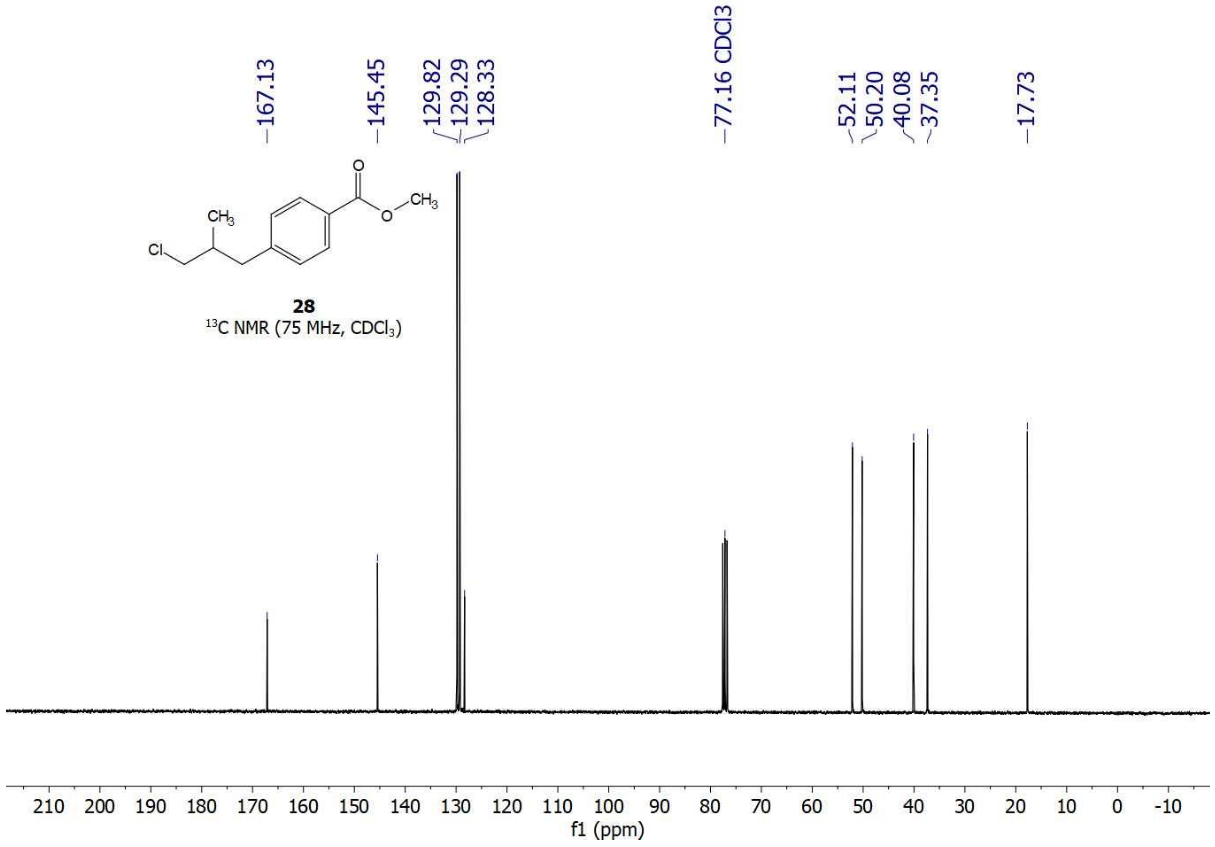

167.13
145.45
129.82
129.29
128.33
77.16 CDCl3
52.11
50.20
40.08
37.35
17.73
CH3
O
O
CH3
Cl
28
13C NMR (75 MHz, CDCl3)
f1 (ppm)

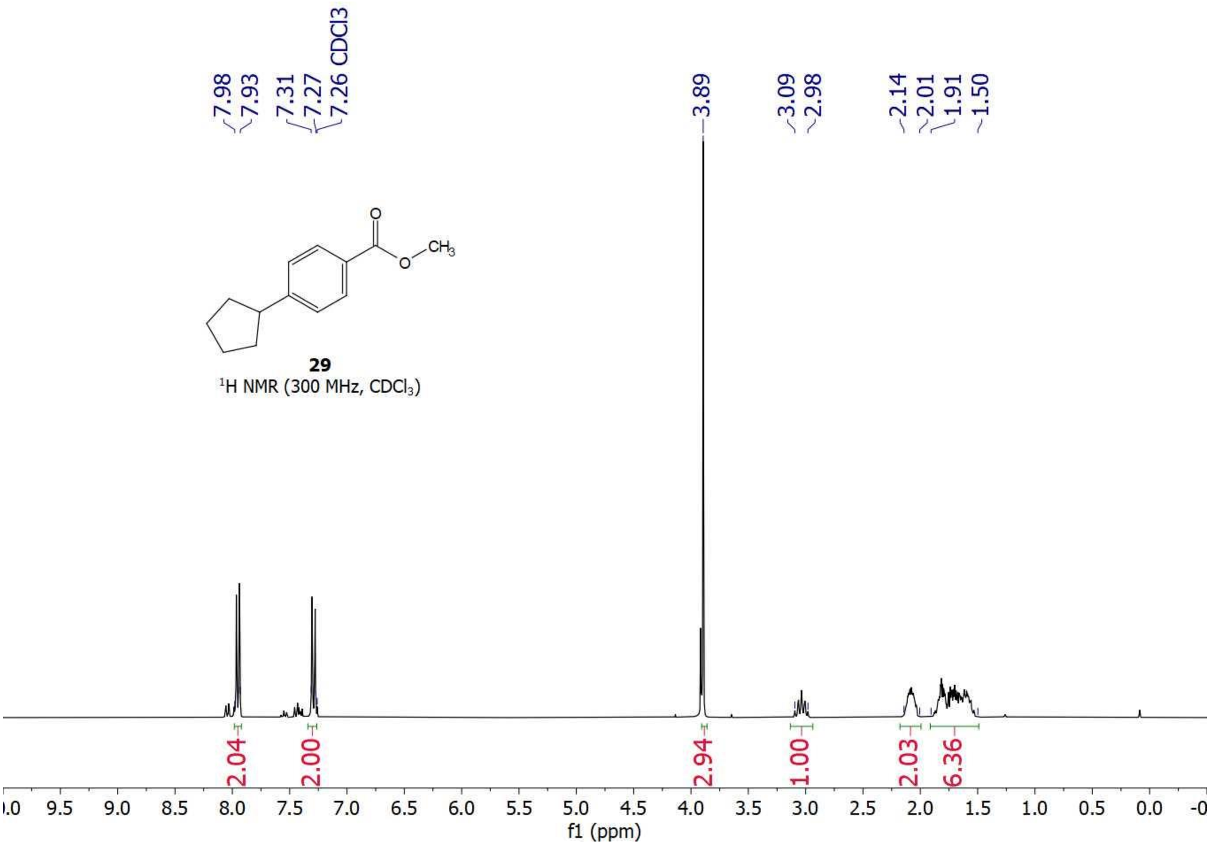

29
^{1}H NMR (300 MHz, $CDCl_3$)

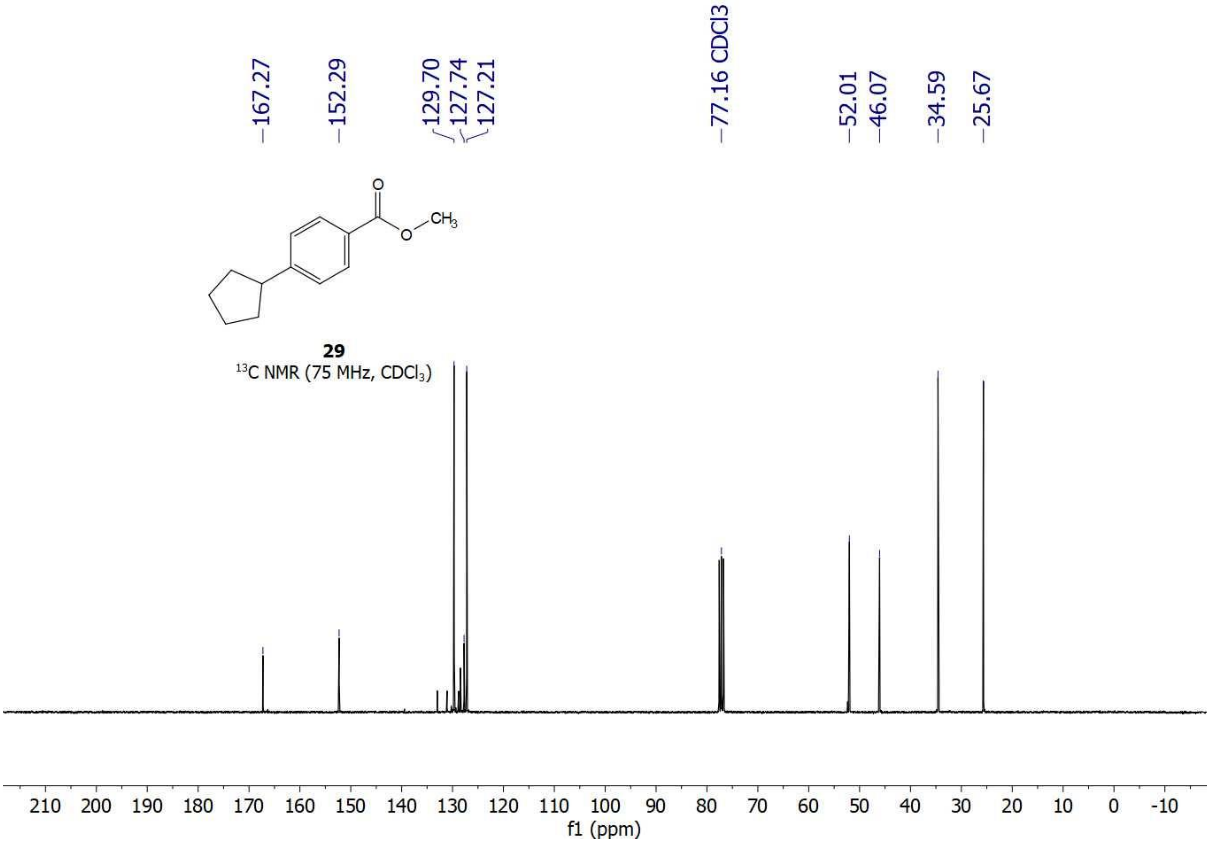

29
^{13}C NMR (75 MHz, $CDCl_3$)

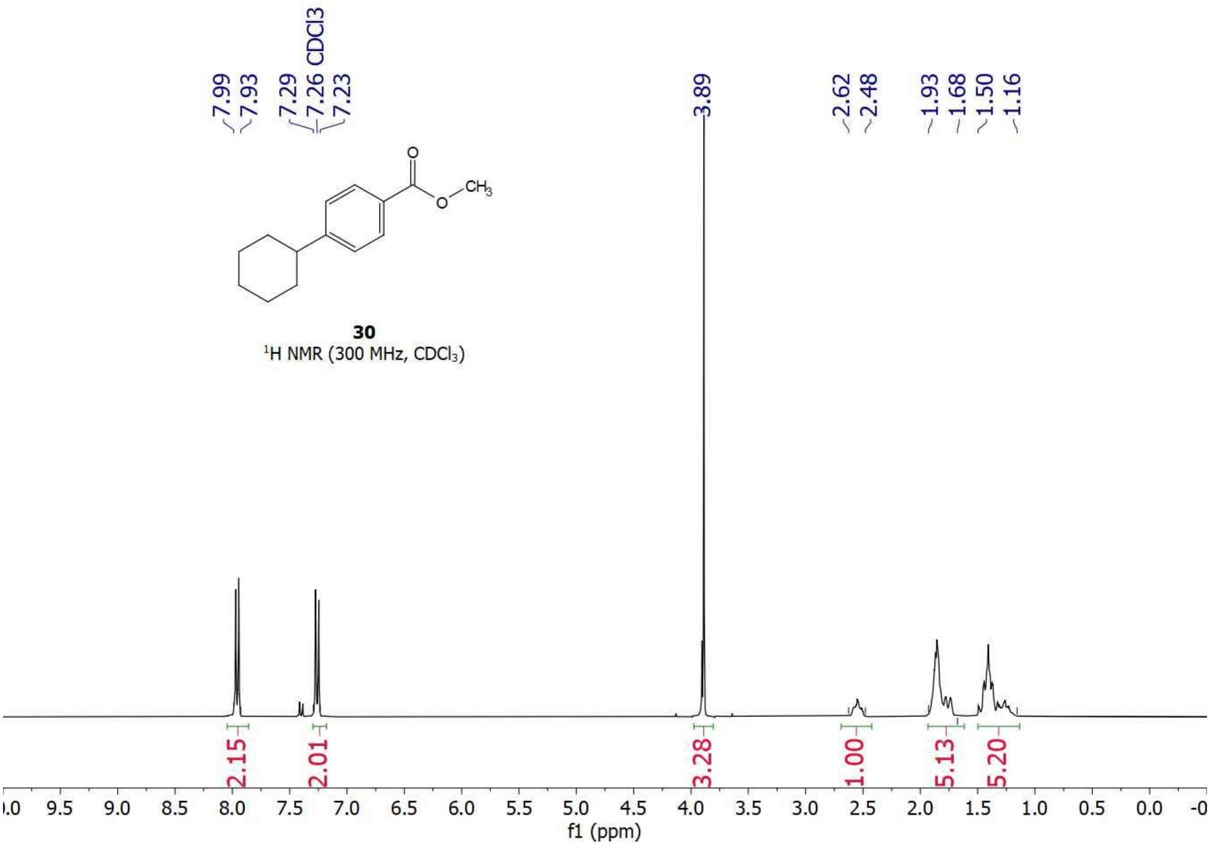
7.99
7.93
7.29
7.26 CDCl3
7.23
3.89
2.62
2.48
1.93
1.68
1.50
1.16
CH3
O
O
30
1H NMR (300 MHz, CDCl3)
2.15
2.01
3.28
1.00
5.13
5.20
f1 (ppm)

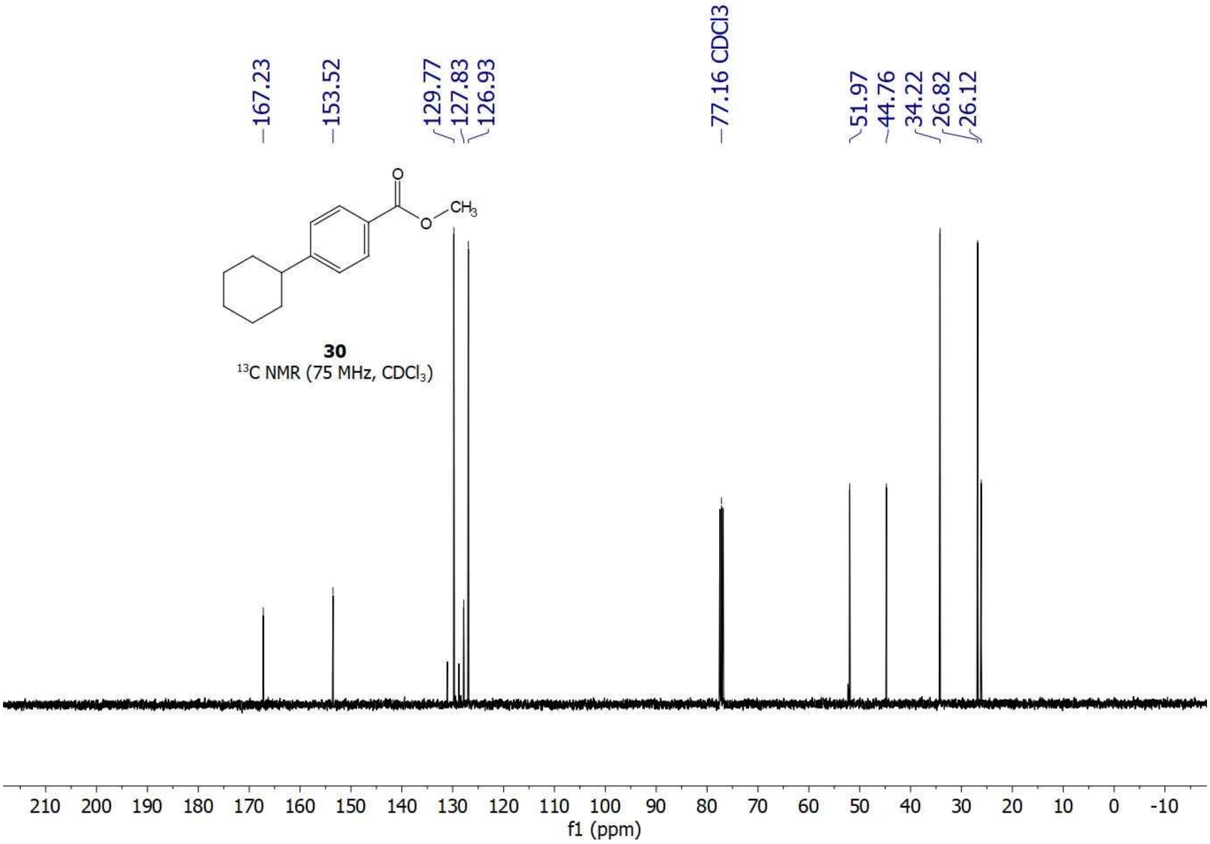
167.23
153.52
129.77
127.83
126.93
77.16 CDCl3
51.97
44.76
34.22
26.82
26.12
CH3
O
O
30
13C NMR (75 MHz, CDCl3)
f1 (ppm)

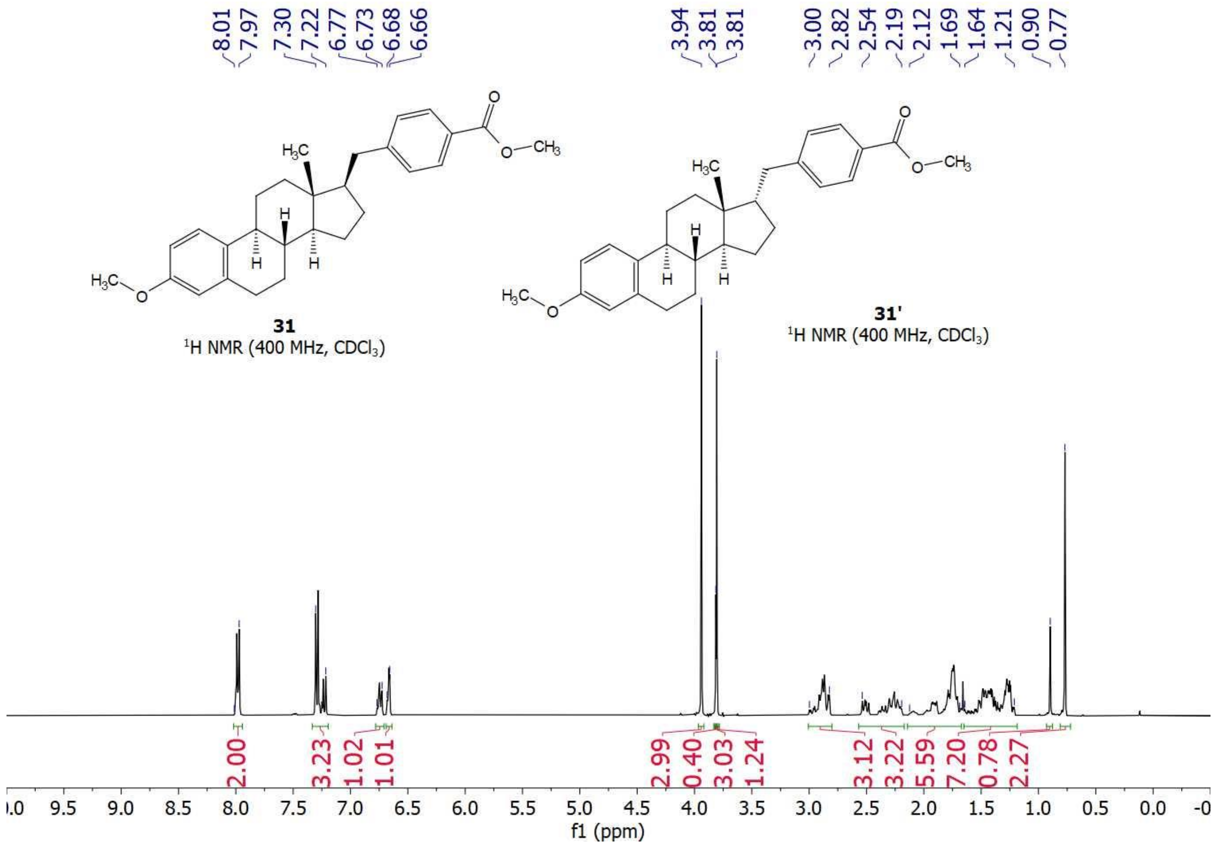
8.01 7.97 7.30 7.22 6.77 6.73 6.68 6.66
3.94 3.81 3.81
3.00 2.82 2.54 2.19 2.12 1.69 1.64 1.21 0.90 0.77
31
^{1}H NMR (400 MHz, CDCl$_3$)
31'
^{1}H NMR (400 MHz, CDCl$_3$)
2.00 3.23 1.02 1.01 2.99 0.40 3.03 1.24 3.12 3.22 5.59 7.20 0.78 2.27
f1 (ppm)

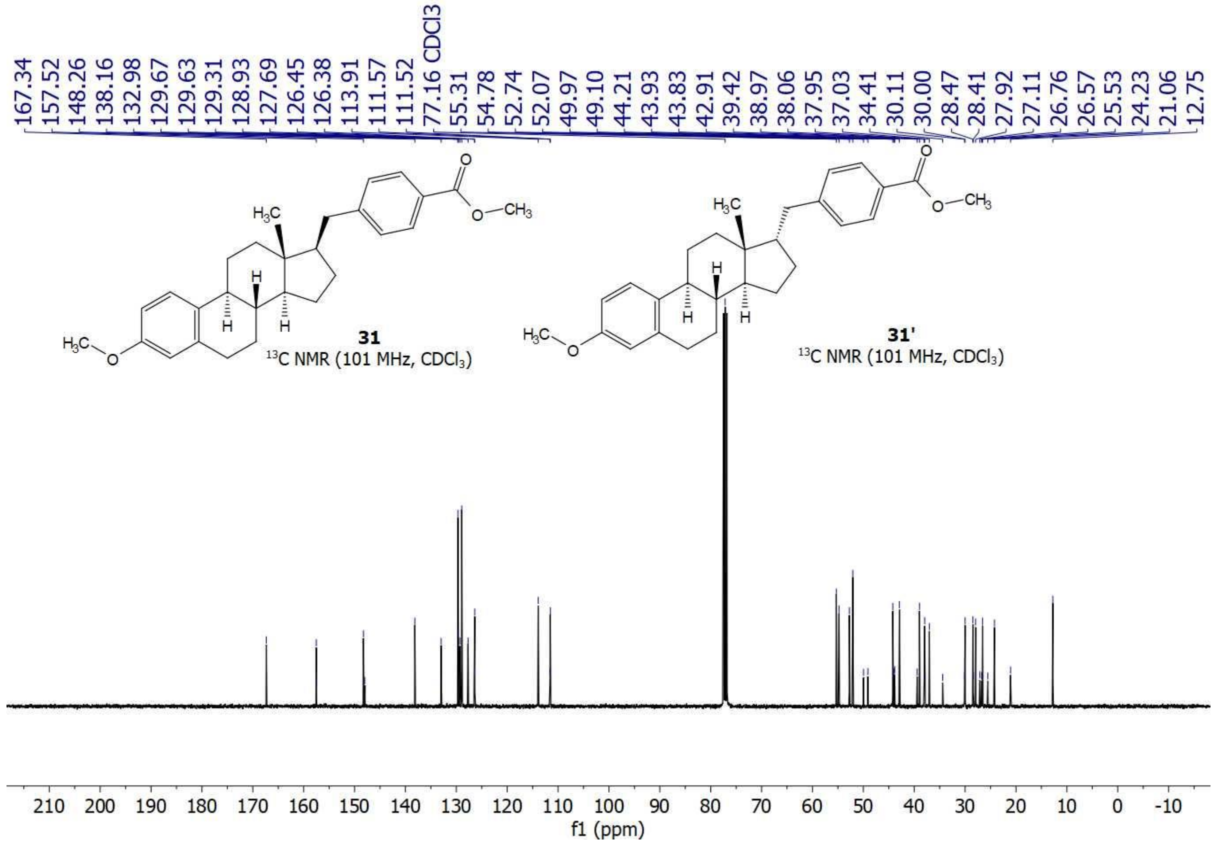
167.34 157.52 148.26 138.16 132.98 129.67 129.63 129.31 128.93 127.69 126.45 126.38 113.91 111.57 111.52 77.16 CDCl3 55.31 54.78 52.74 52.07 49.97 49.10 44.21 43.93 43.83 42.91 39.42 38.97 38.06 37.95 37.03 34.41 30.11 30.00 28.47 28.41 27.92 27.11 26.76 26.57 25.53 24.23 21.06 12.75
31
^{13}C NMR (101 MHz, CDCl$_3$)
31'
^{13}C NMR (101 MHz, CDCl$_3$)
f1 (ppm)

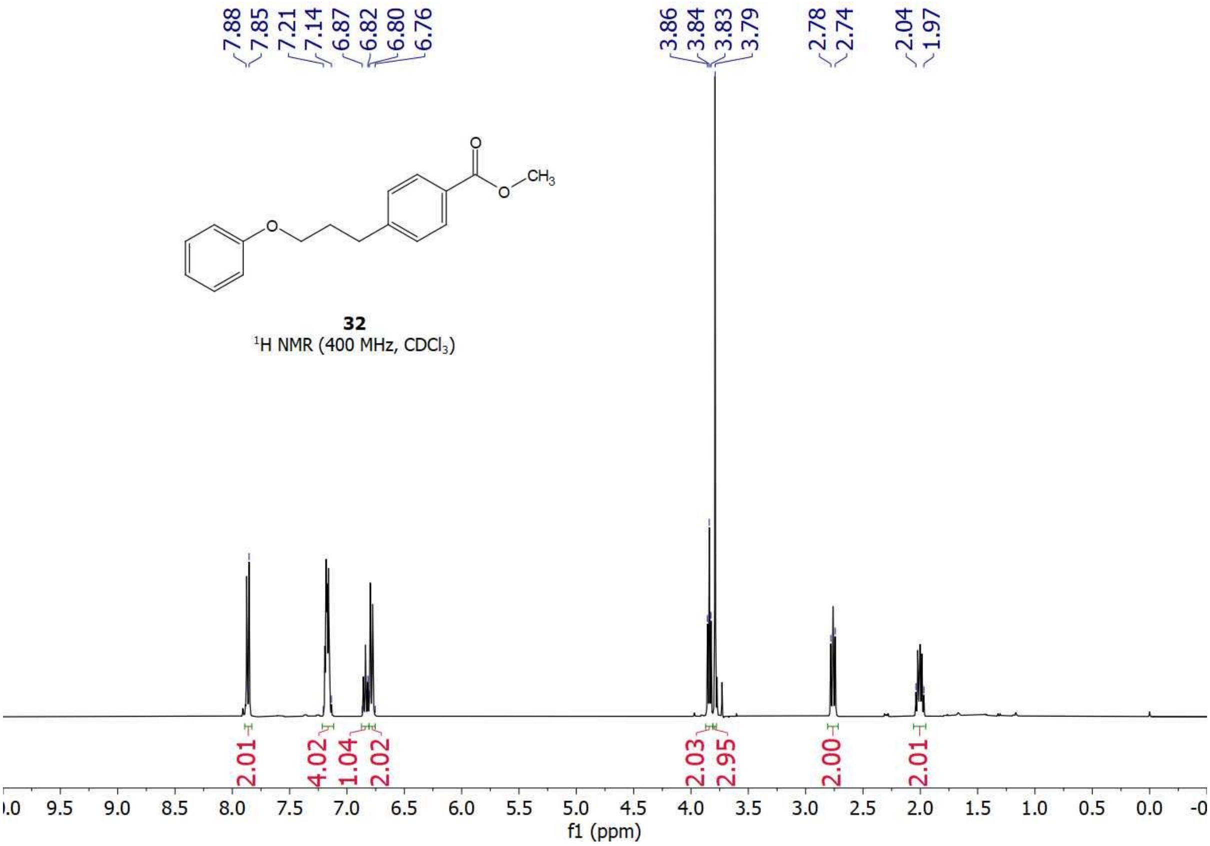
7.88
7.85
7.21
7.14
6.87
6.82
6.80
6.76
3.86
3.84
3.83
3.79
2.78
2.74
2.04
1.97
O
O
CH3
O
32
1H NMR (400 MHz, CDCl3)
2.01
4.02
1.04
2.02
2.03
2.95
2.00
2.01
9.5 9.0 8.5 8.0 7.5 7.0 6.5 6.0 5.5 5.0 4.5 4.0 3.5 3.0 2.5 2.0 1.5 1.0 0.5 0.0
f1 (ppm)

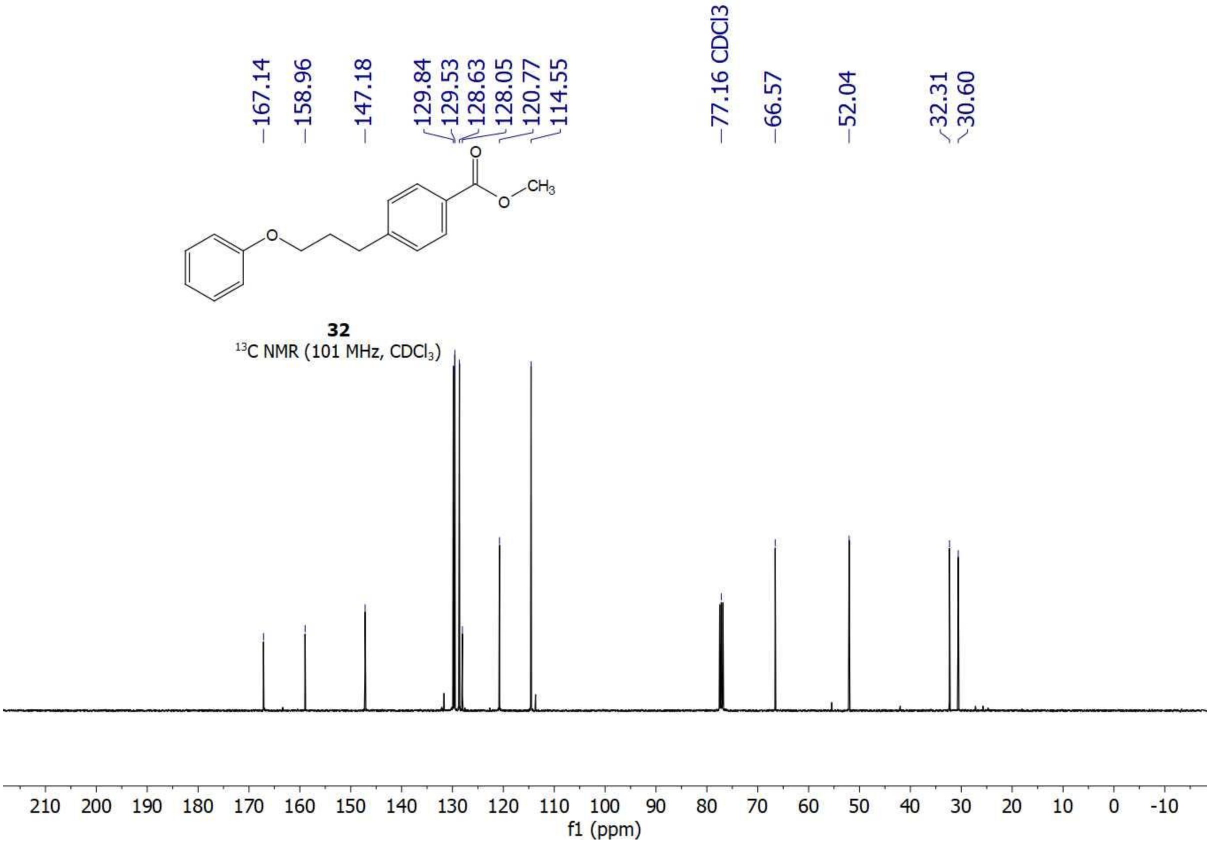
167.14
158.96
147.18
129.84
129.53
128.63
128.05
120.77
114.55
77.16 CDCl3
66.57
52.04
32.31
30.60
O
O
CH3
O
32
13C NMR (101 MHz, CDCl3)
210 200 190 180 170 160 150 140 130 120 110 100 90 80 70 60 50 40 30 20 10 0 -10
f1 (ppm)

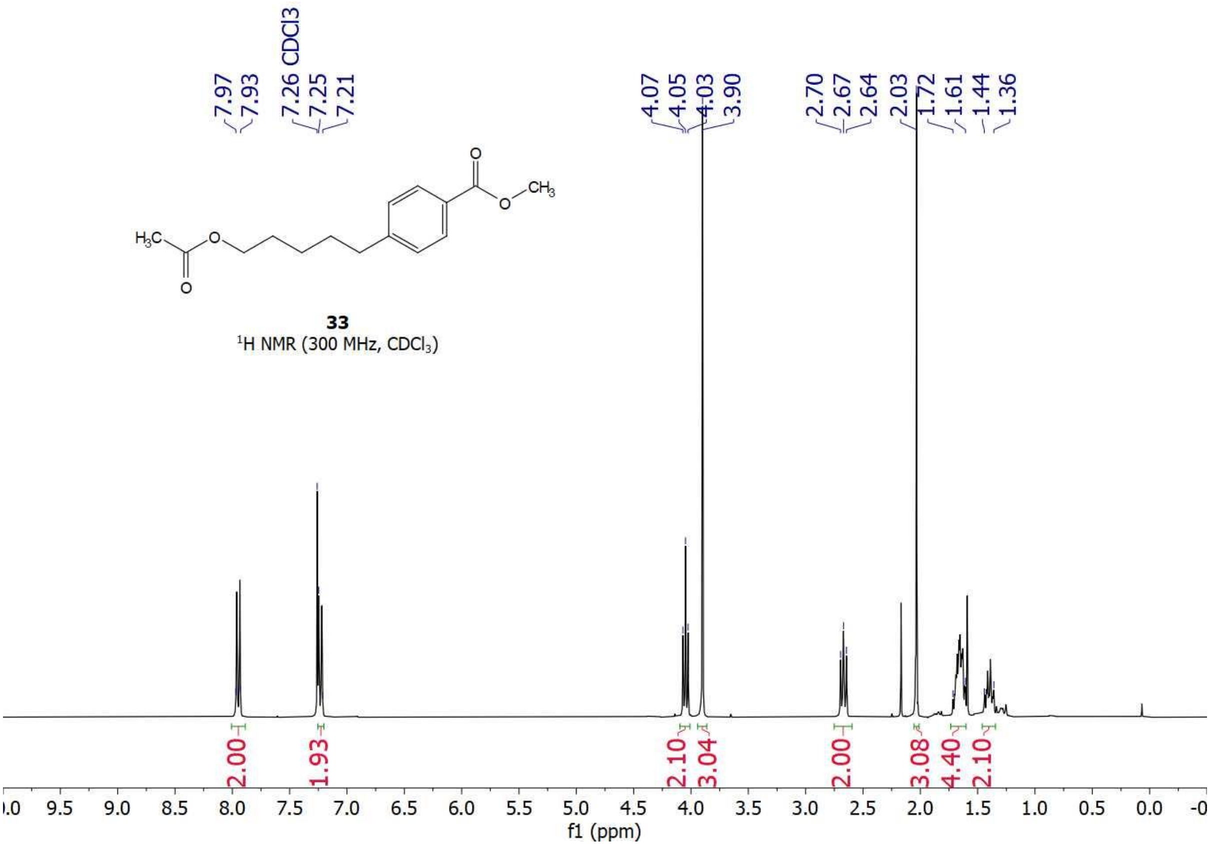
7.97
7.93
7.26 CDCl3
7.25
7.21
4.07
4.05
4.03
3.90
2.70
2.67
2.64
2.03
1.72
1.61
1.44
1.36
33
^{1}H NMR (300 MHz, $CDCl_3$)
2.00
1.93
2.10
3.04
2.00
3.08
4.40
2.10
f1 (ppm)

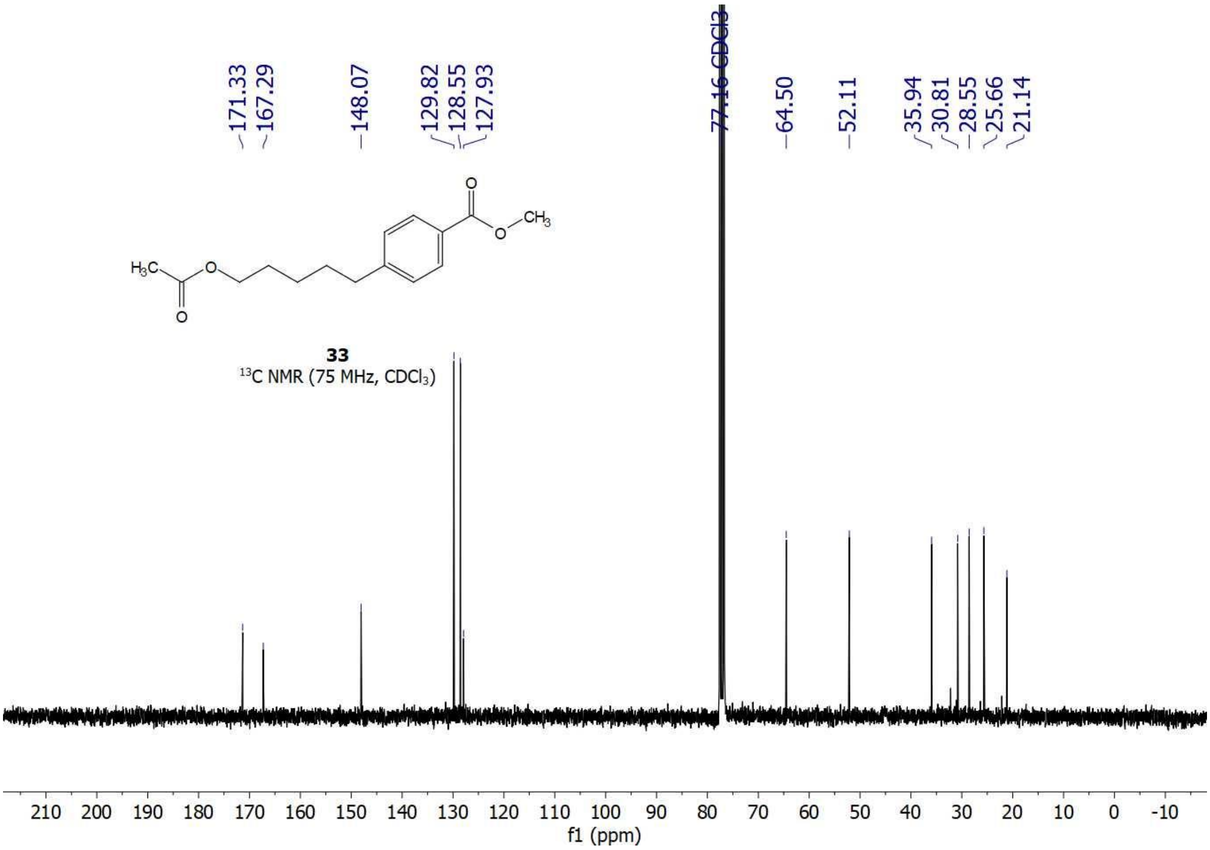
171.33
167.29
148.07
129.82
128.55
127.93
77.16 CDCl3
64.50
52.11
35.94
30.81
28.55
25.66
21.14
33
^{13}C NMR (75 MHz, $CDCl_3$)
f1 (ppm)

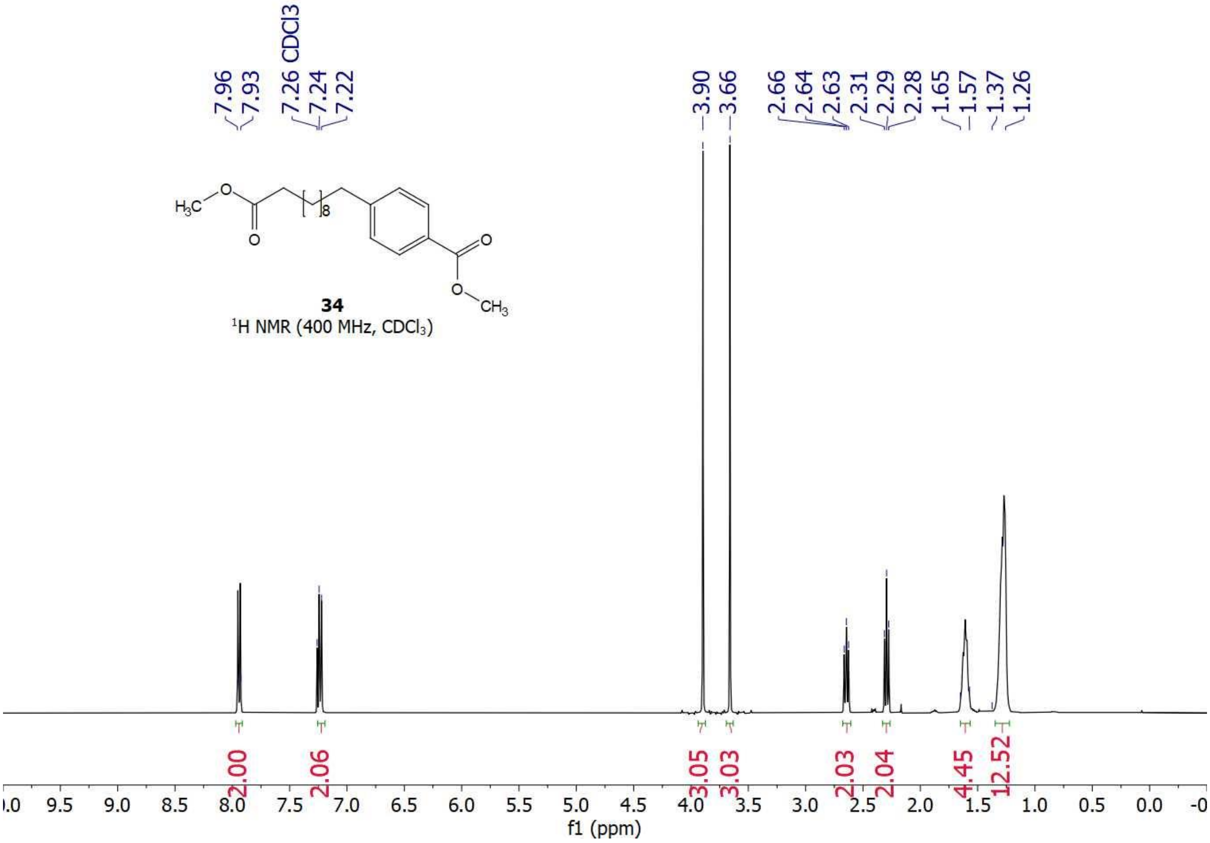
7.96
7.93
7.26 CDCl3
7.24
7.22
3.90
3.66
2.66
2.64
2.63
2.31
2.29
2.28
1.65
1.57
1.37
1.26
34
^{1}H NMR (400 MHz, $CDCl_3$)
2.00
2.06
3.05
3.03
2.03
2.04
4.45
12.52
f1 (ppm)

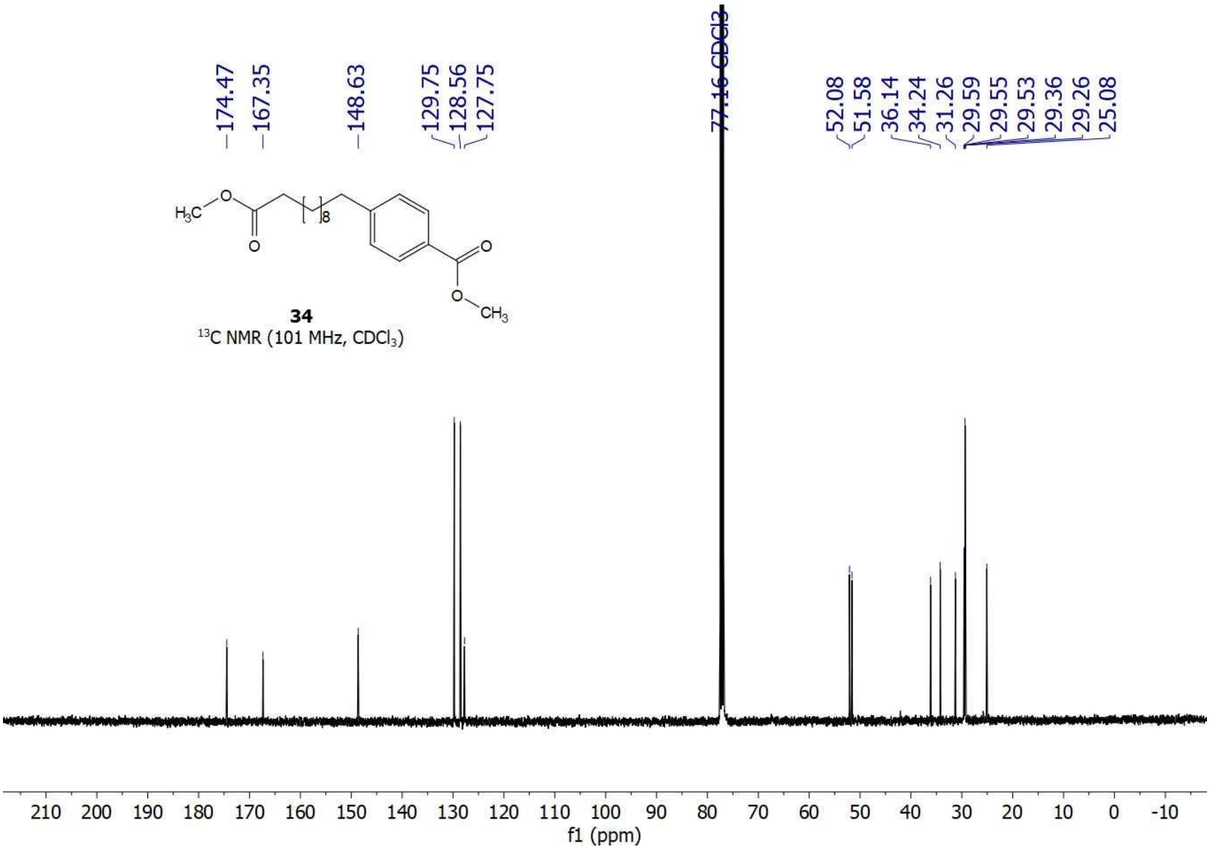
174.47
167.35
148.63
129.75
128.56
127.75
77.16 CDCl3
52.08
51.58
36.14
34.24
31.26
29.59
29.55
29.53
29.36
29.26
25.08
34
^{13}C NMR (101 MHz, $CDCl_3$)
f1 (ppm)

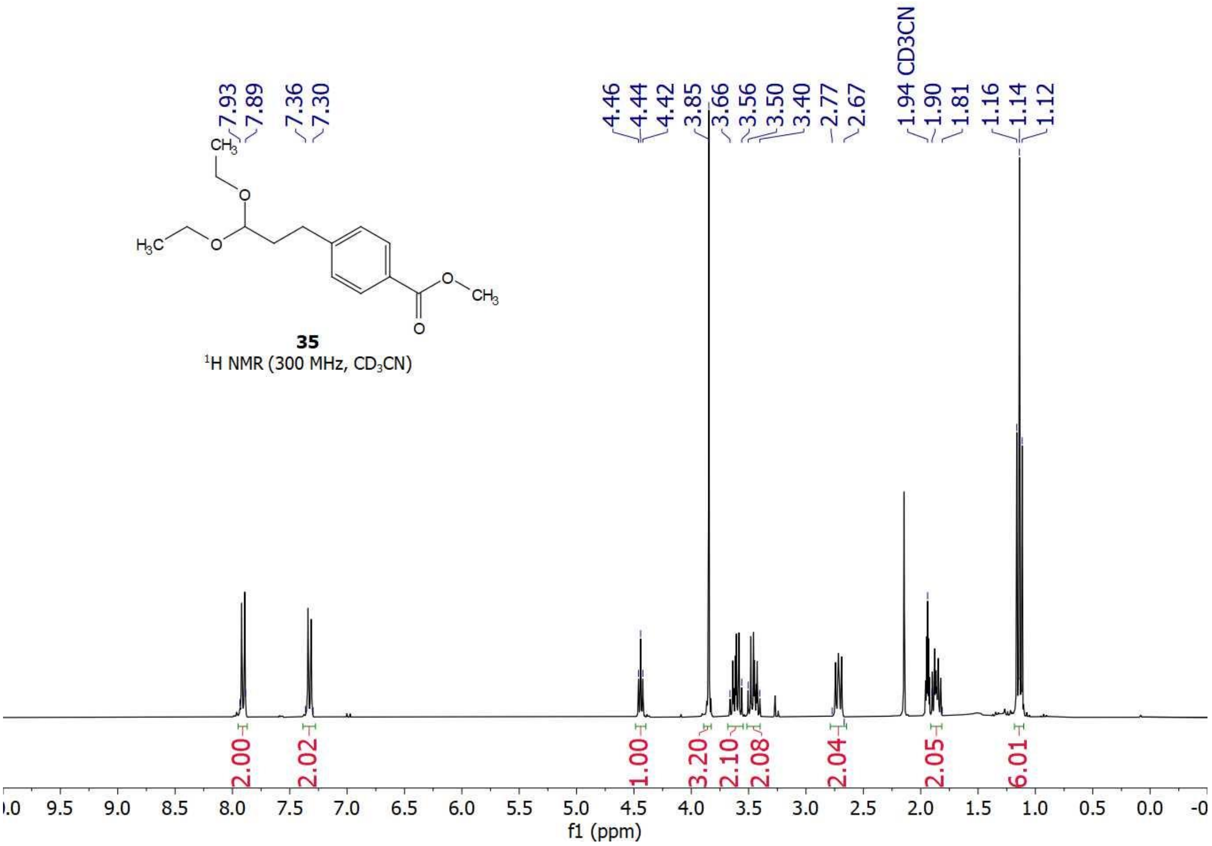
7.93
7.89
7.36
7.30
4.46
4.44
4.42
3.85
3.66
3.56
3.50
3.40
2.77
2.67
1.94 CD3CN
1.90
1.81
1.16
1.14
1.12
35
^{1}H NMR (300 MHz, CD$_3$CN)
2.00
2.02
1.00
3.20
2.10
2.08
2.04
2.05
6.01
f1 (ppm)

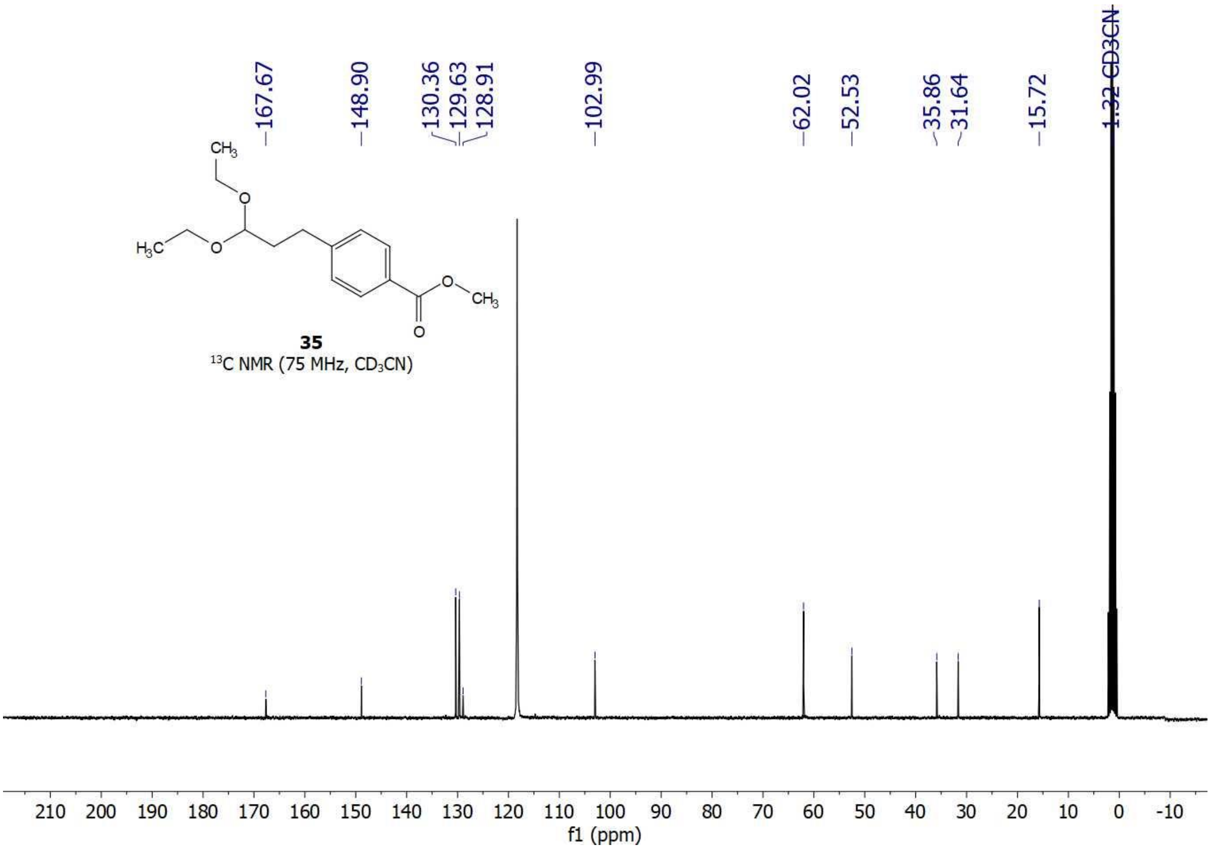
167.67
148.90
130.36
129.63
128.91
102.99
62.02
52.53
35.86
31.64
15.72
1.32 CD3CN
35
^{13}C NMR (75 MHz, CD$_3$CN)
f1 (ppm)

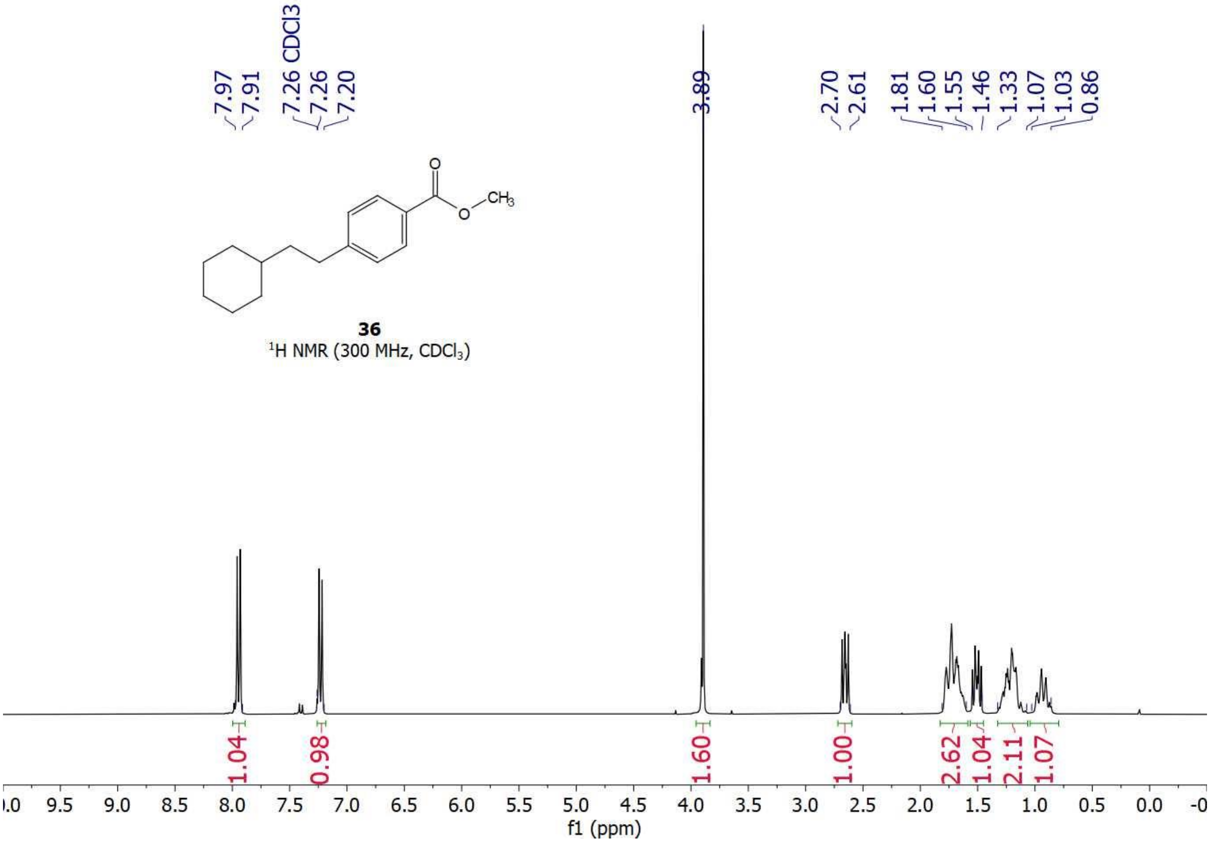
7.97
7.91
7.26 CDCl3
7.26
7.20
3.89
2.70
2.61
1.81
1.60
1.55
1.46
1.33
1.07
1.03
0.86
36
1H NMR (300 MHz, CDCl3)
1.04
0.98
1.60
1.00
2.62
1.04
2.11
1.07
f1 (ppm)

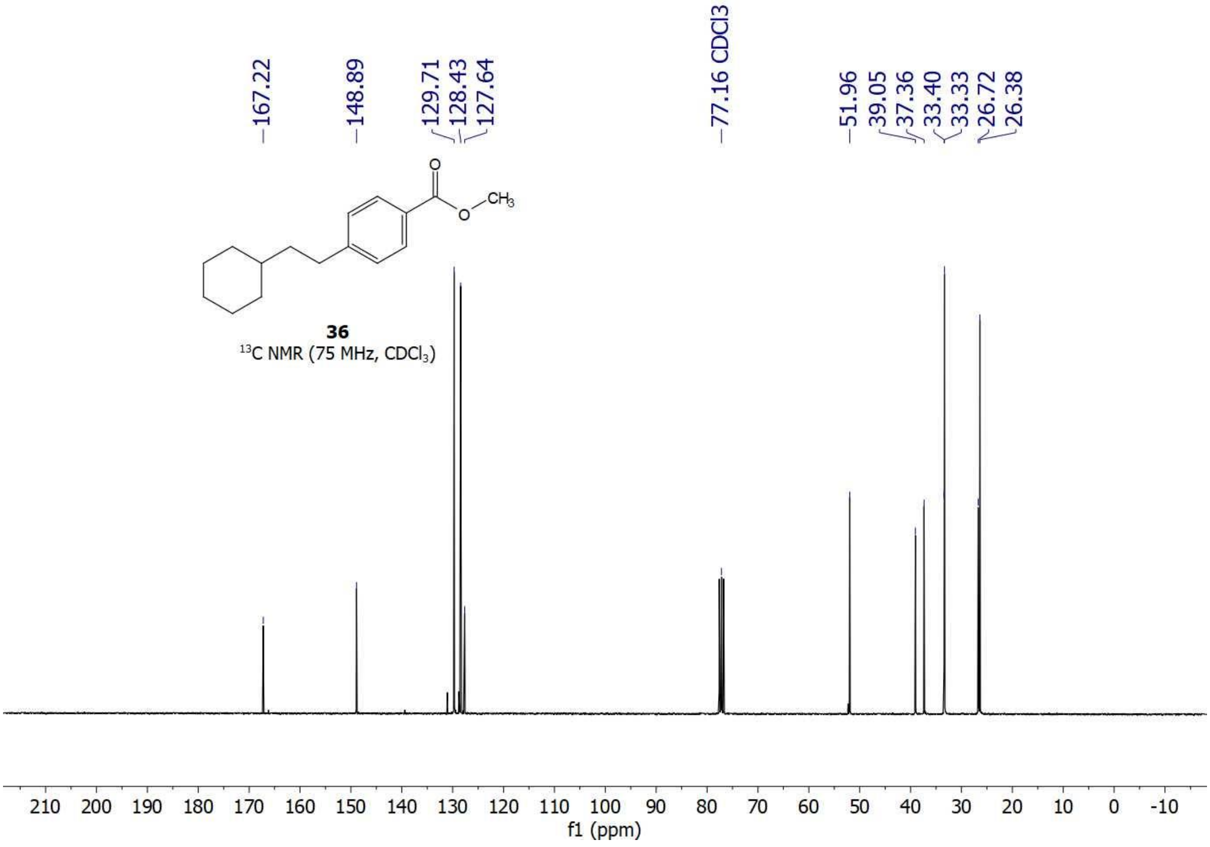
167.22
148.89
129.71
128.43
127.64
77.16 CDCl3
51.96
39.05
37.36
33.40
33.33
26.72
26.38
36
13C NMR (75 MHz, CDCl3)
f1 (ppm)

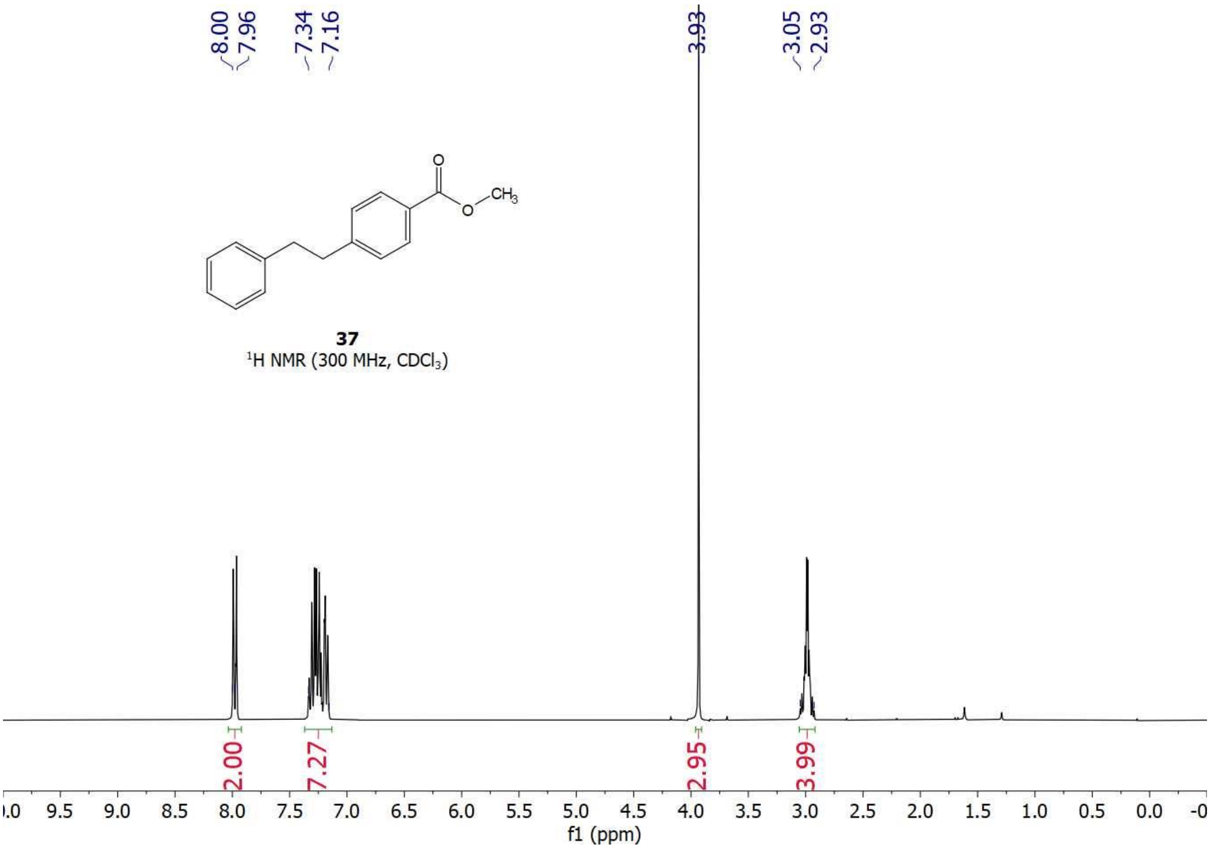
8.00
7.96
7.34
7.16
3.93
3.05
2.93
O
O
CH3
37
1H NMR (300 MHz, CDCl3)
2.00
7.27
2.95
3.99
f1 (ppm)

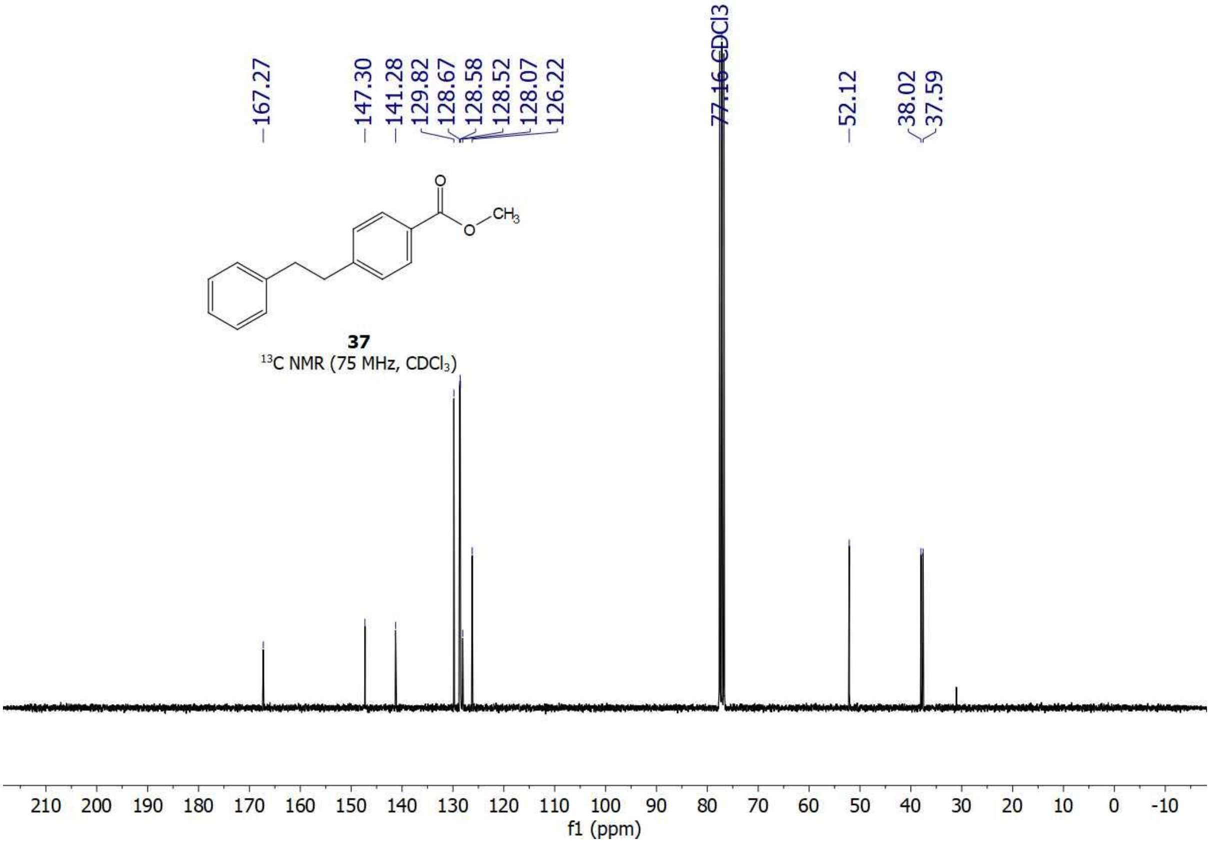
167.27
147.30
141.28
129.82
128.67
128.58
128.52
128.07
126.22
77.16 CDCl3
52.12
38.02
37.59
O
O
CH3
37
13C NMR (75 MHz, CDCl3)
f1 (ppm)

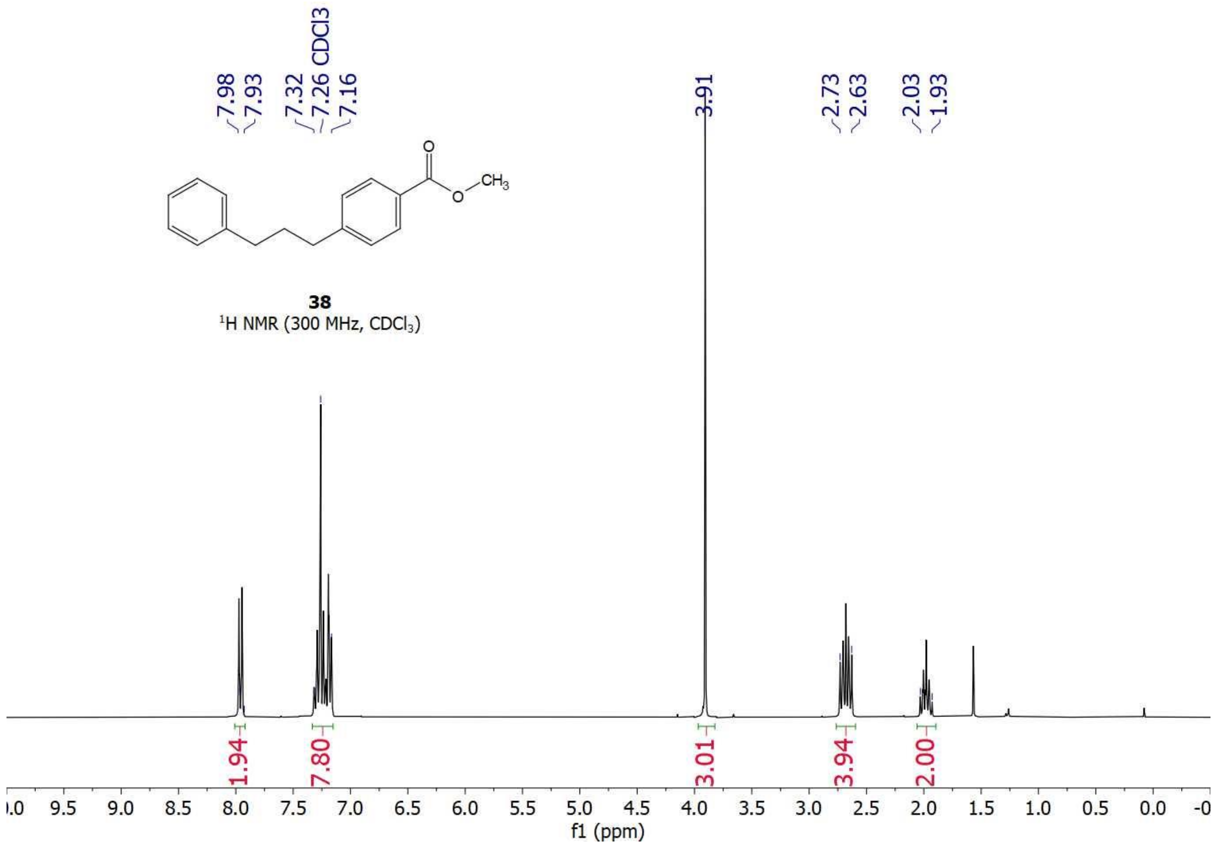
7.98
7.93
7.32
7.26 CDCl3
7.16
3.91
2.73
2.63
2.03
1.93
CH3
O
O
38
1H NMR (300 MHz, CDCl3)
1.94
7.80
3.01
3.94
2.00
f1 (ppm)

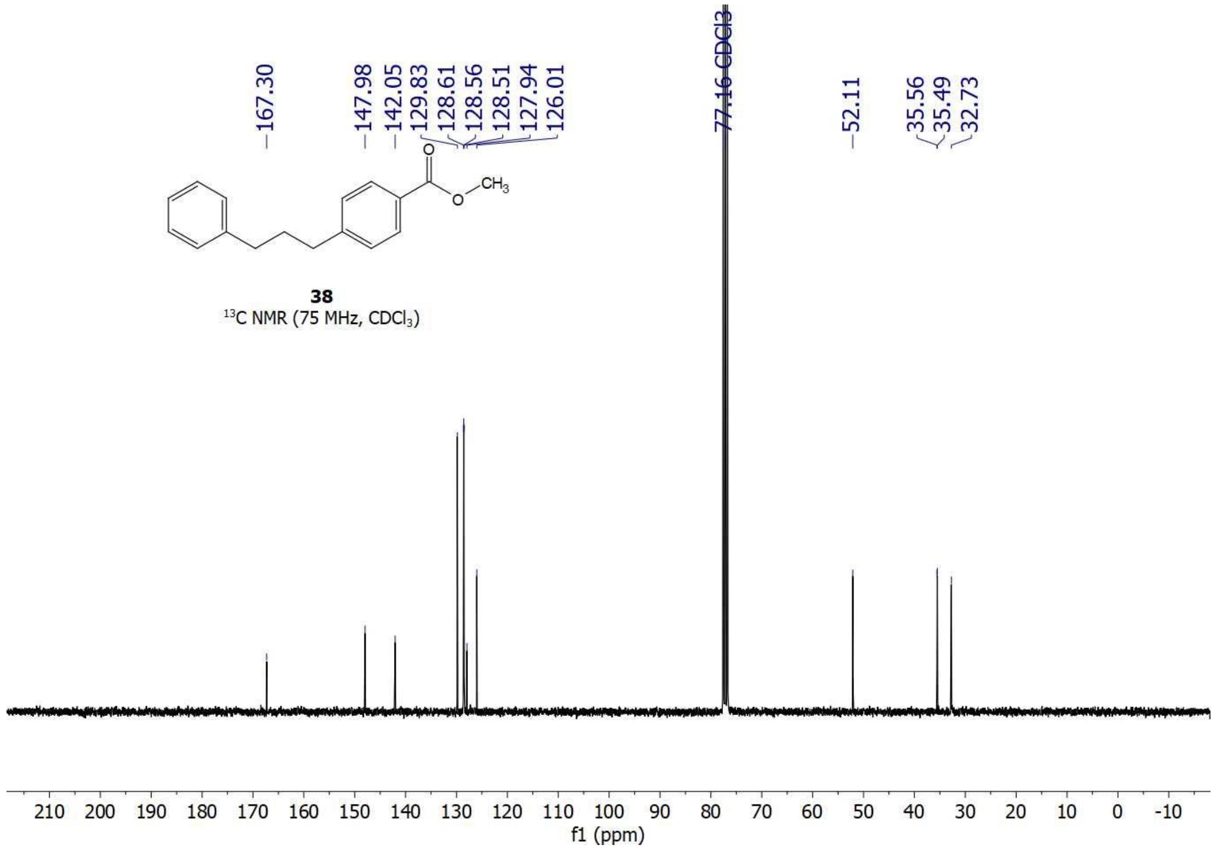
167.30
147.98
142.05
129.83
128.61
128.56
128.51
127.94
126.01
77.16 CDCl3
52.11
35.56
35.49
32.73
CH3
O
O
38
13C NMR (75 MHz, CDCl3)
f1 (ppm)

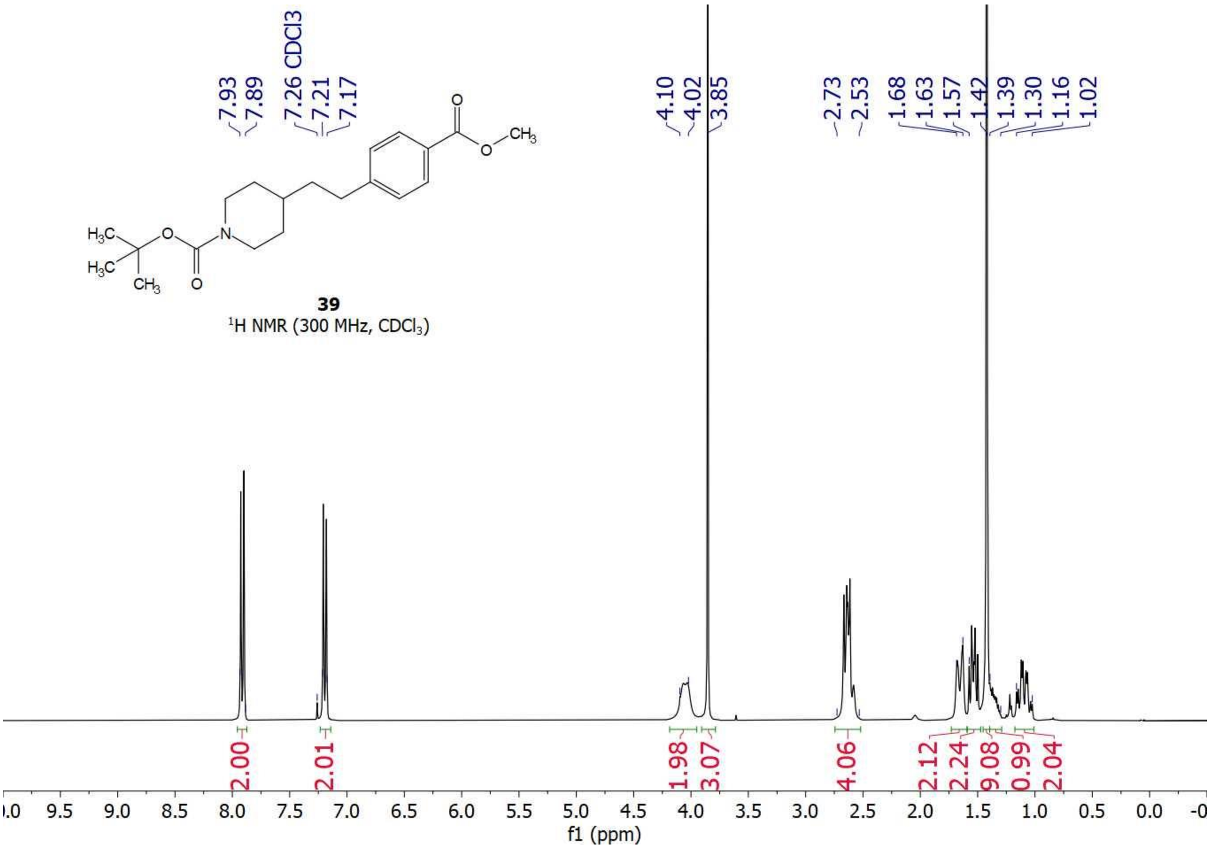
7.93
7.89
7.26 CDCl3
7.21
7.17
4.10
4.02
3.85
2.73
2.53
1.68
1.63
1.57
1.42
1.39
1.30
1.16
1.02
H_3C
H_3C
CH_3
O
N
O
O
CH_3
39
^{1}H NMR (300 MHz, $CDCl_3$)
2.00
2.01
1.98
3.07
4.06
2.12
2.24
9.08
0.99
2.04
f1 (ppm)

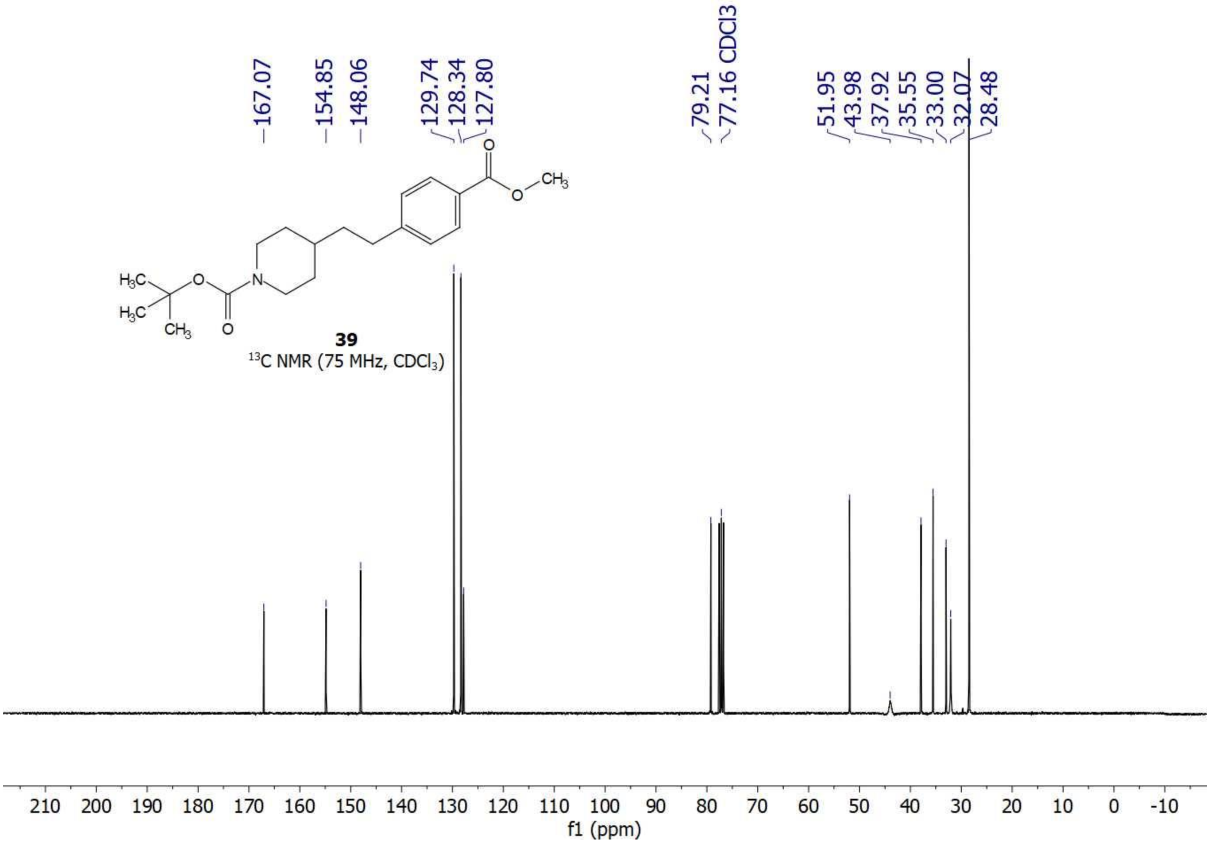
167.07
154.85
148.06
129.74
128.34
127.80
79.21
77.16 CDCl3
51.95
43.98
37.92
35.55
33.00
32.07
28.48
H_3C
H_3C
CH_3
O
N
O
O
CH_3
39
^{13}C NMR (75 MHz, $CDCl_3$)
f1 (ppm)

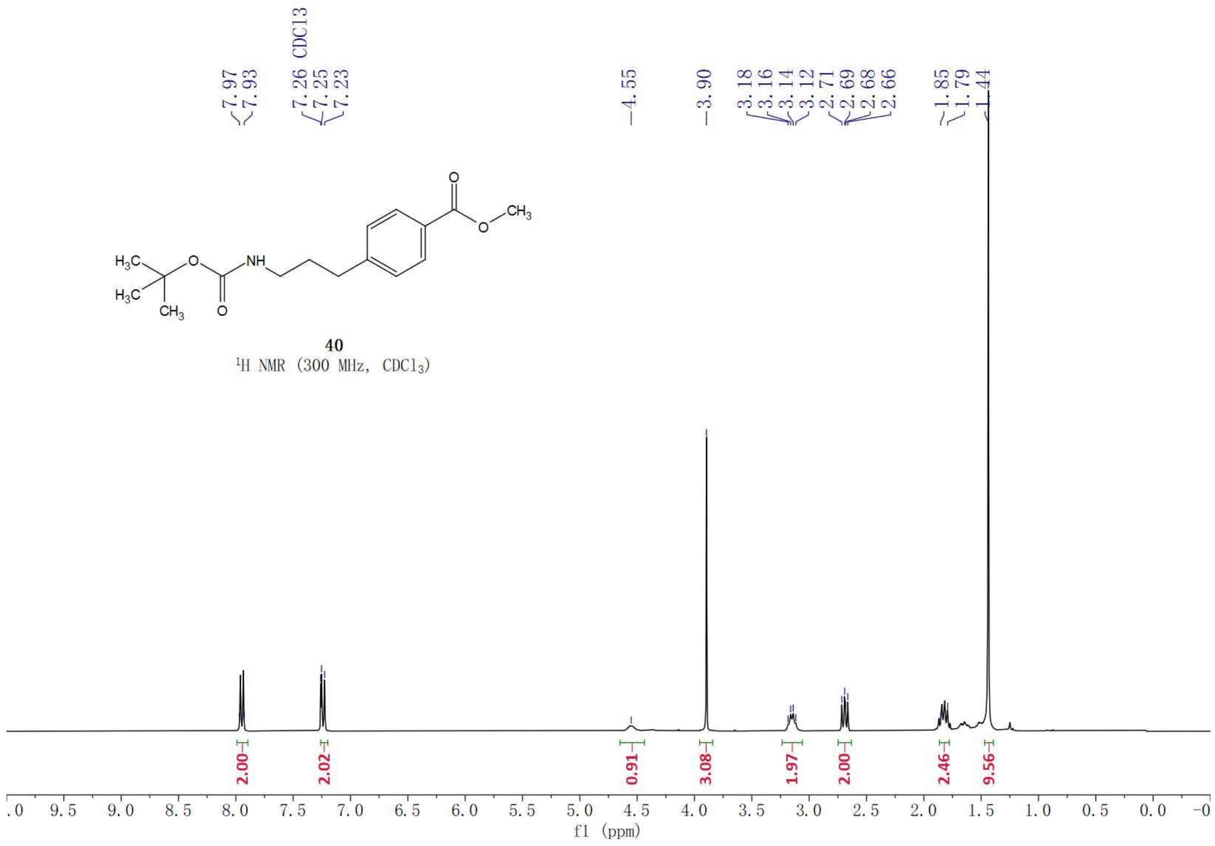
7.97
7.93
7.26 CDCl3
7.25
7.23
4.55
3.90
3.18
3.16
3.14
3.12
2.71
2.69
2.68
2.66
1.85
1.79
1.44
40
^1H NMR (300 MHz, $CDCl_3$)
2.00
2.02
0.91
3.08
1.97
2.00
2.46
9.56
f1 (ppm)

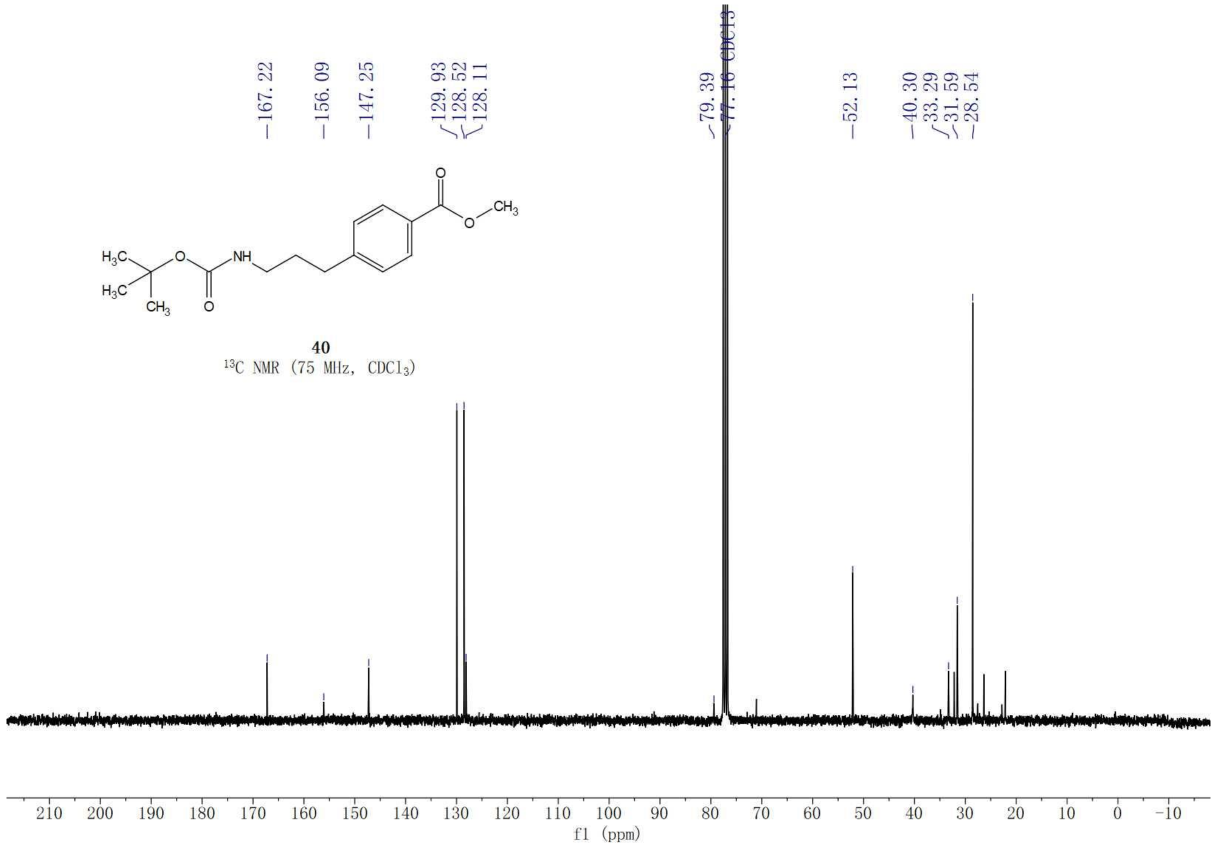
167.22
156.09
147.25
129.93
128.52
128.11
79.39
77.16 CDCl3
52.13
40.30
33.29
31.59
28.54
40
^{13}C NMR (75 MHz, $CDCl_3$)
f1 (ppm)

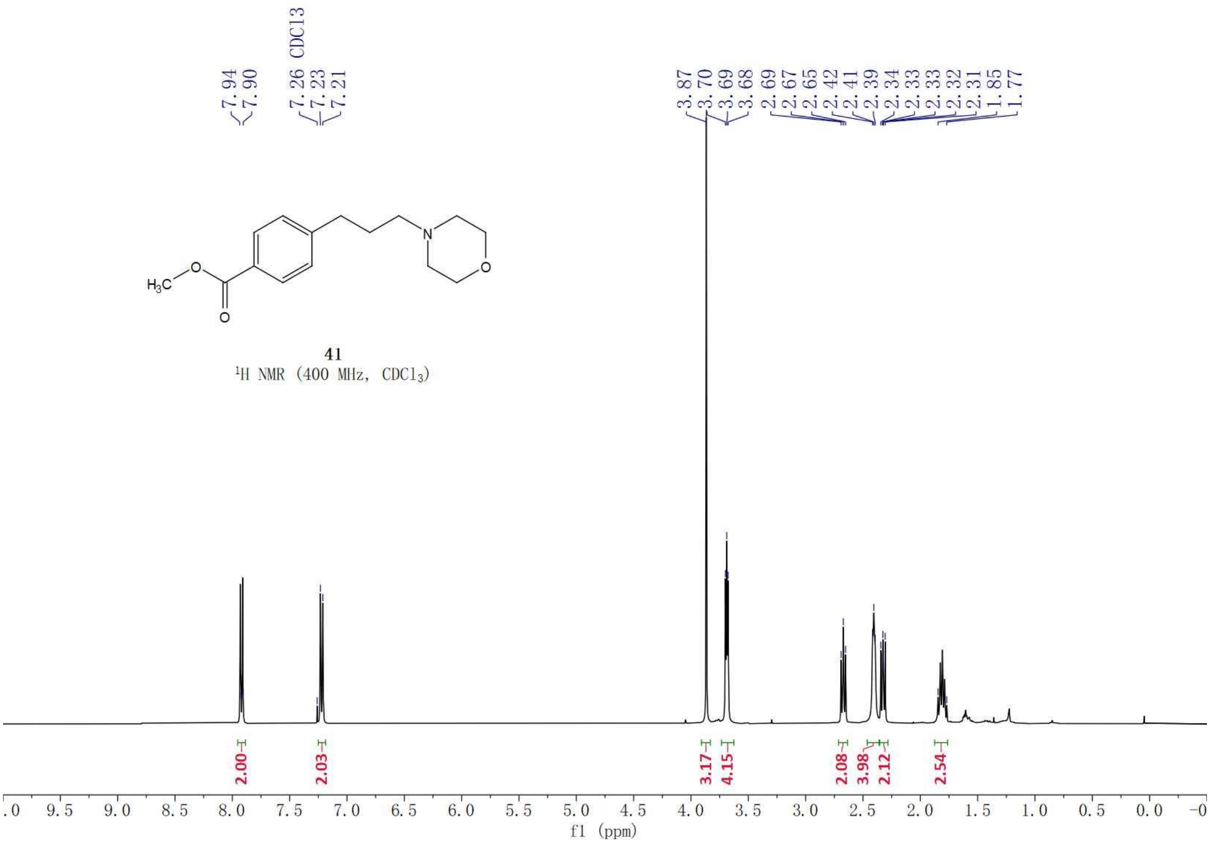
7.94
7.90
7.26 CDCl3
7.23
7.21
3.87
3.70
3.69
3.68
2.69
2.67
2.65
2.42
2.41
2.39
2.34
2.33
2.33
2.32
2.31
1.85
1.77
H3C
O
O
N
O
41
1H NMR (400 MHz, CDCl3)
2.00
2.03
3.17
4.15
2.08
3.98
2.12
2.54
.0 9.5 9.0 8.5 8.0 7.5 7.0 6.5 6.0 5.5 5.0 4.5 4.0 3.5 3.0 2.5 2.0 1.5 1.0 0.5 0.0 −0
f1 (ppm)

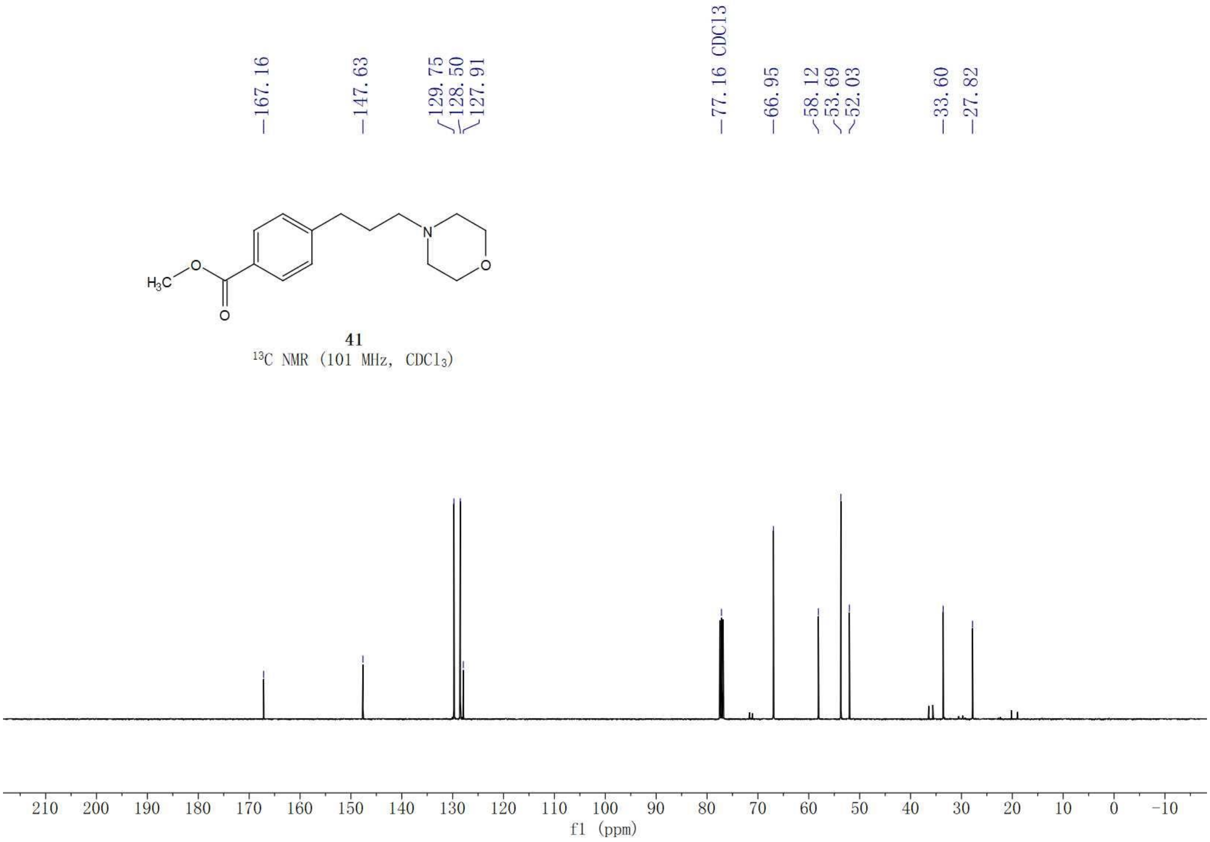
167.16
147.63
129.75
128.50
127.91
77.16 CDCl3
66.95
58.12
53.69
52.03
33.60
27.82
H3C
O
O
N
O
41
13C NMR (101 MHz, CDCl3)
210 200 190 180 170 160 150 140 130 120 110 100 90 80 70 60 50 40 30 20 10 0 −10
f1 (ppm)

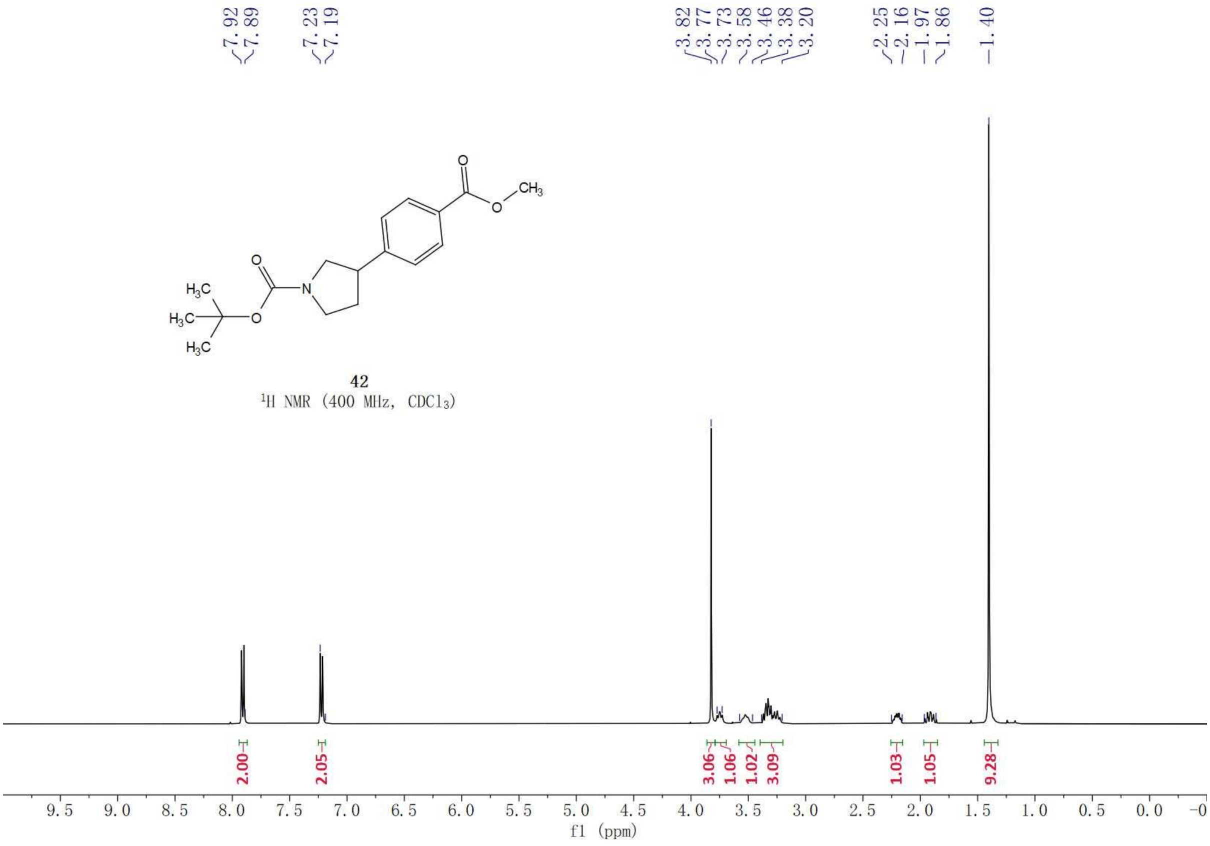
7.92
7.89
7.23
7.19
3.82
3.77
3.73
3.58
3.46
3.38
3.20
2.25
2.16
1.97
1.86
1.40
42
^{1}H NMR (400 MHz, CDCl$_3$)
2.00
2.05
3.06
1.06
1.02
3.09
1.03
1.05
9.28
f1 (ppm)

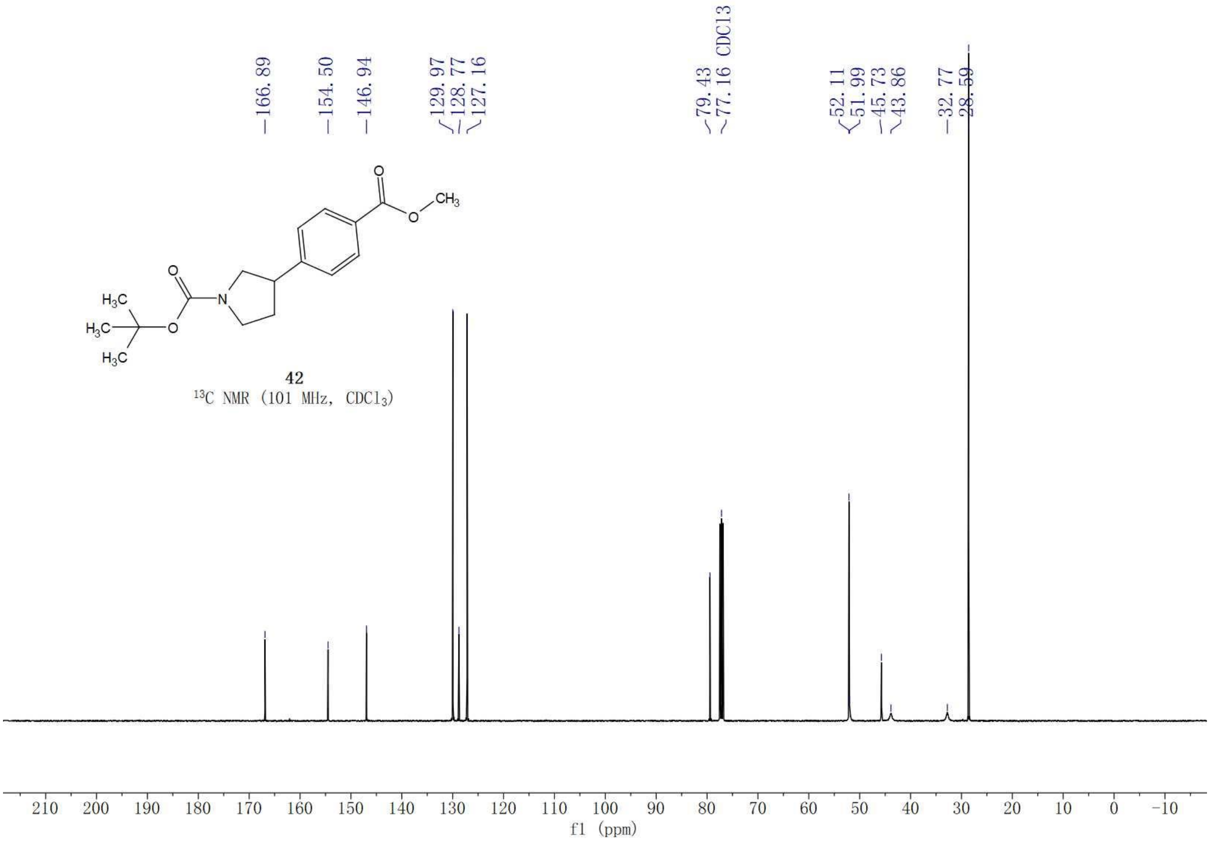
166.89
154.50
146.94
129.97
128.77
127.16
79.43
77.16 CDCl3
52.11
51.99
45.73
43.86
32.77
28.59
42
^{13}C NMR (101 MHz, CDCl$_3$)
f1 (ppm)

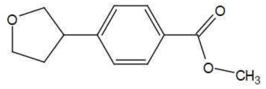

43
^{1}H NMR (300 MHz, CDCl$_3$)

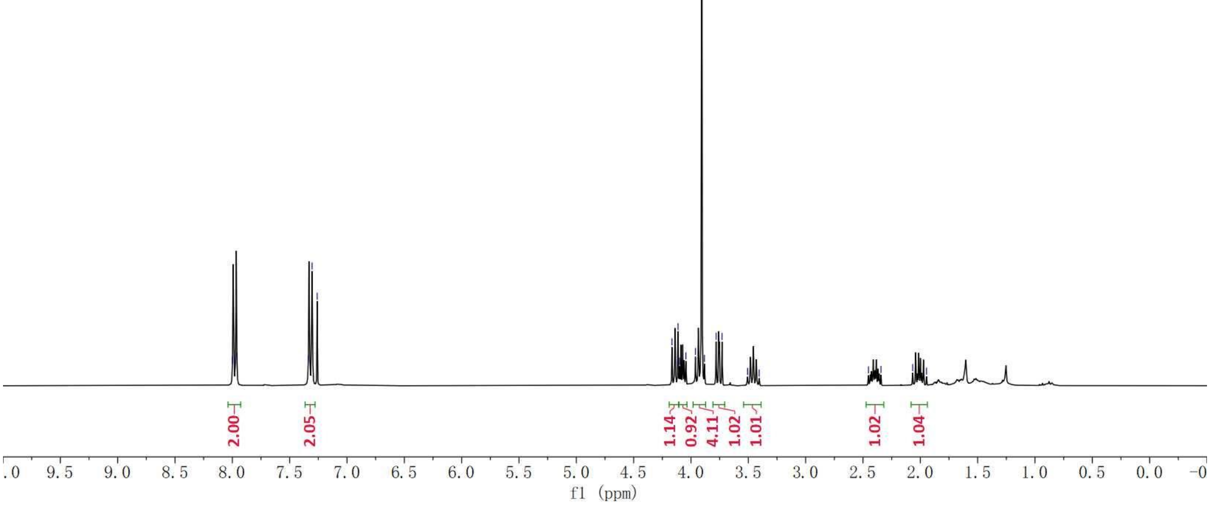

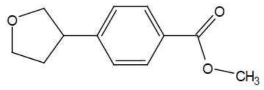

43
^{13}C NMR (75 MHz, CDCl$_3$)

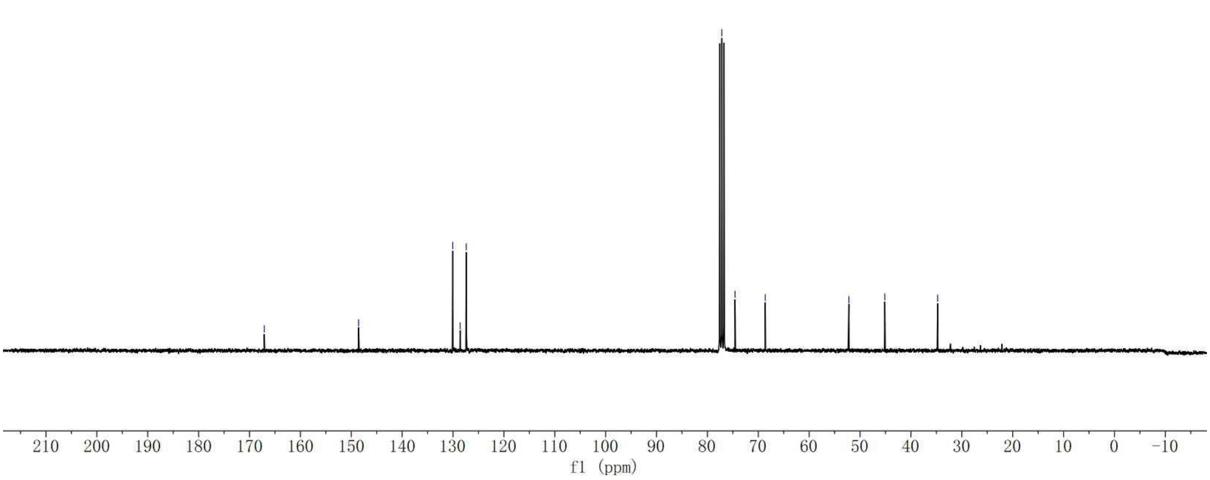

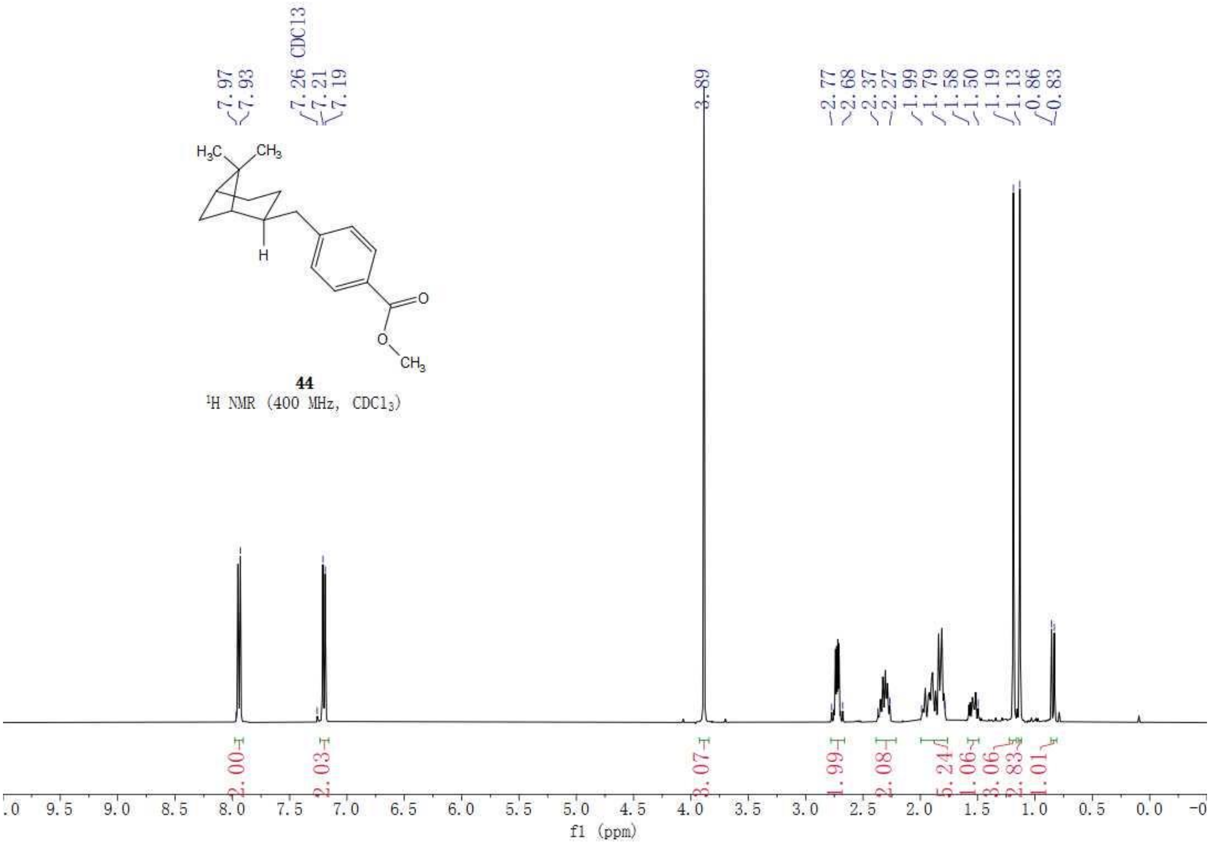

44
^{1}H NMR (400 MHz, $CDCl_3$)

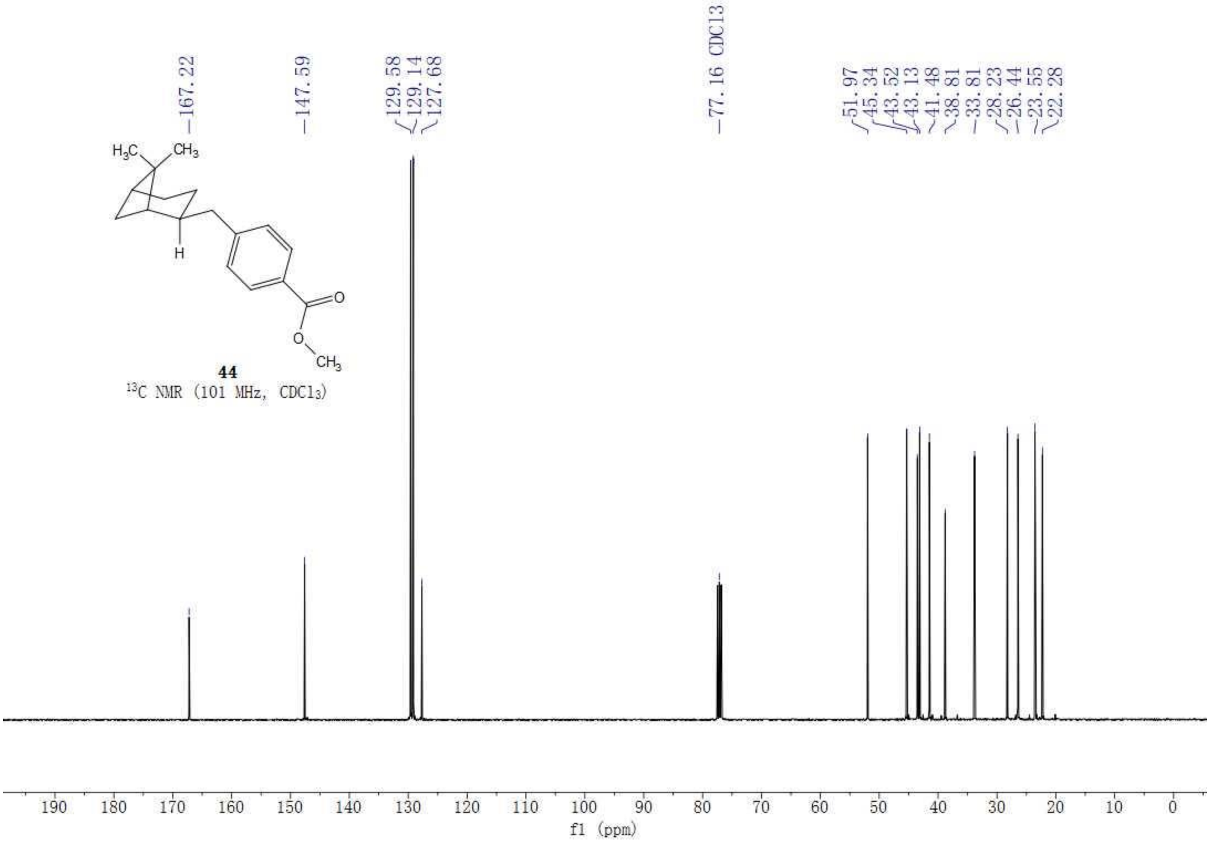

44
^{13}C NMR (101 MHz, $CDCl_3$)

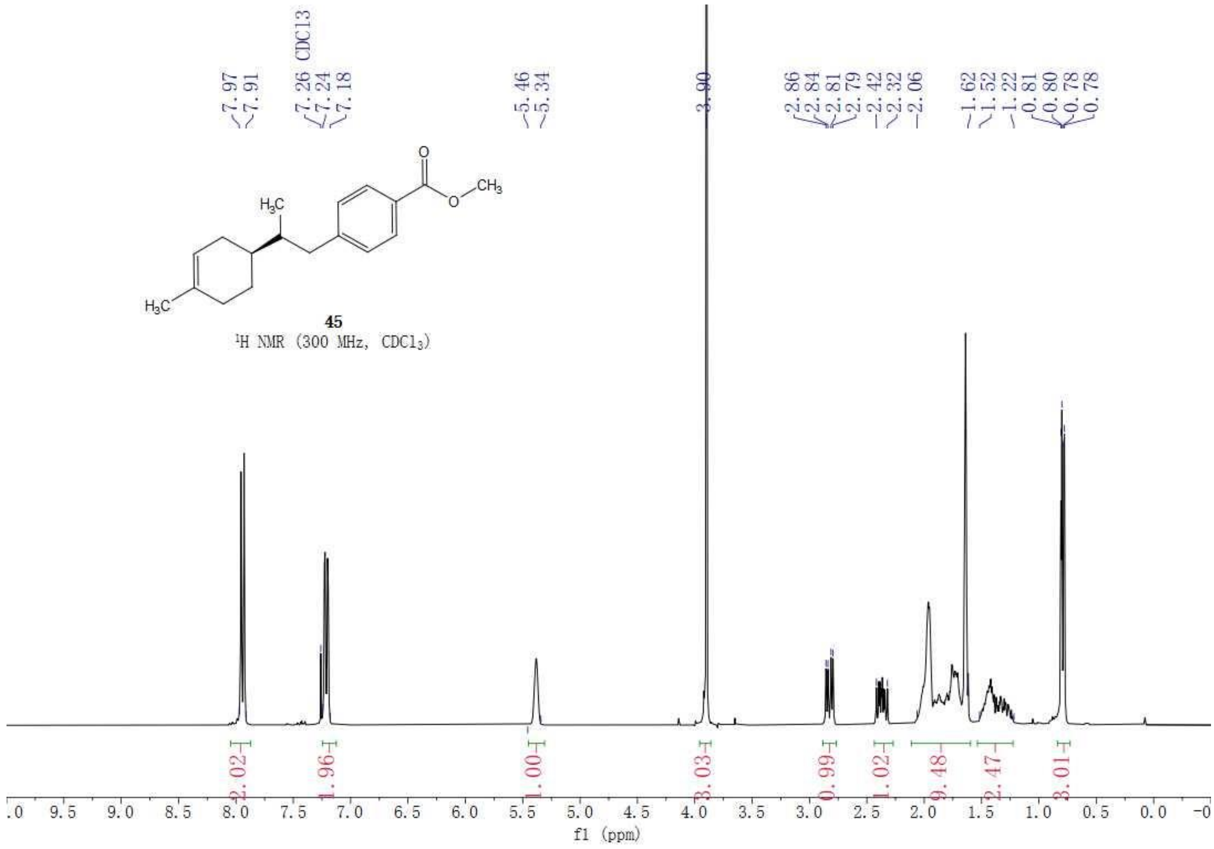
7.97
7.91
7.26 CDCl3
7.24
7.18
5.46
5.34
3.90
2.86
2.84
2.81
2.79
2.42
2.32
2.06
1.62
1.52
1.22
0.81
0.80
0.78
0.78
O
O
CH3
H3C
H3C
45
1H NMR (300 MHz, CDCl3)
2.02
1.96
1.00
3.03
0.99
1.02
9.48
2.47
3.01
f1 (ppm)

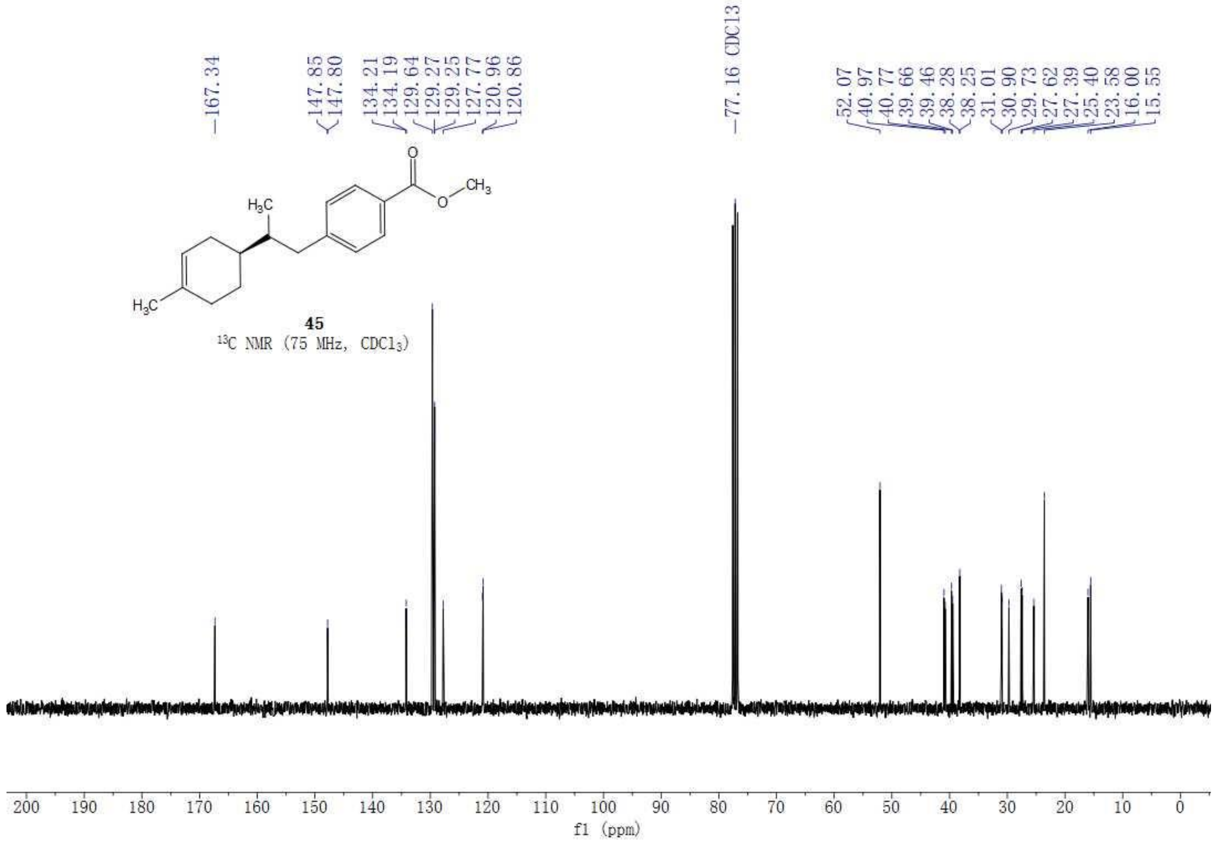
167.34
147.85
147.80
134.21
134.19
129.64
129.27
129.25
127.77
120.96
120.86
77.16 CDCl3
52.07
40.97
40.77
39.66
39.46
38.28
38.25
31.01
30.90
29.73
27.62
27.39
25.40
23.58
16.00
15.55
O
O
CH3
H3C
H3C
45
13C NMR (75 MHz, CDCl3)
f1 (ppm)

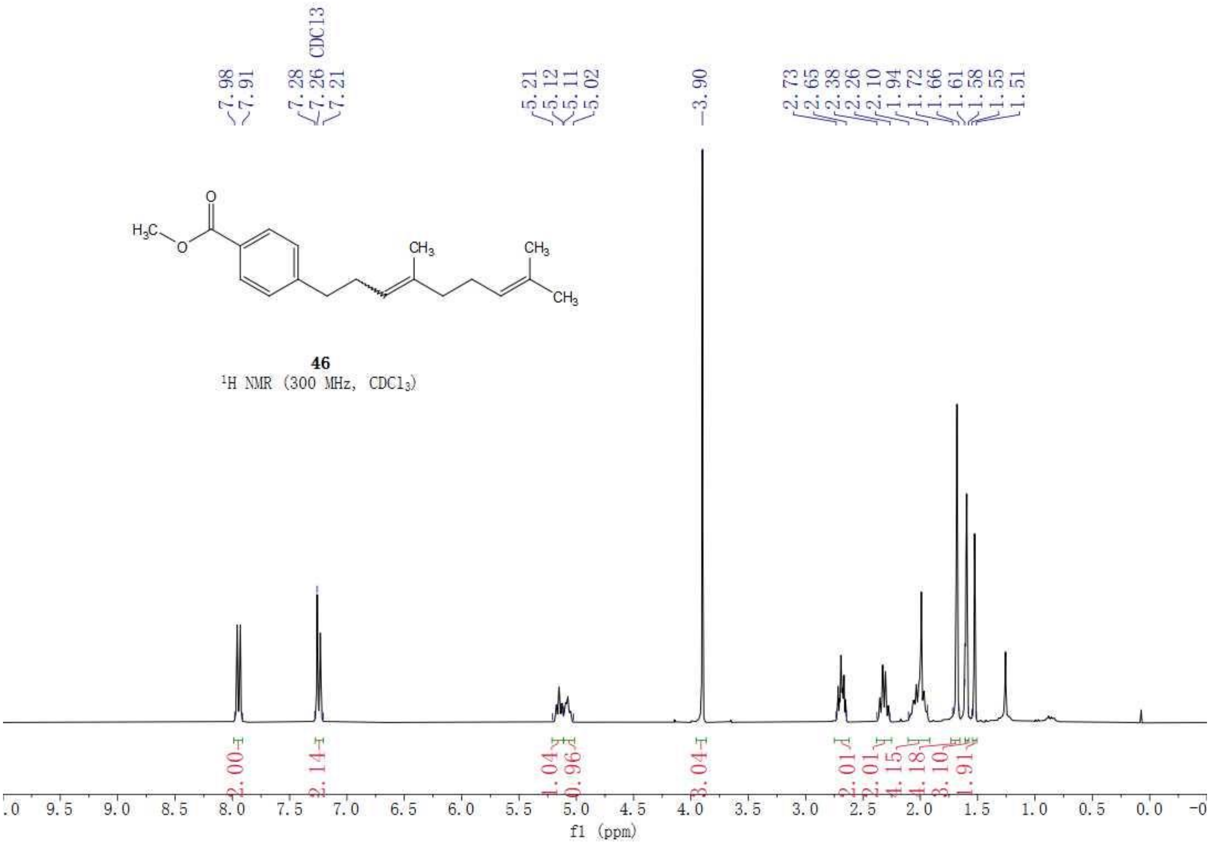

46

^{1}H NMR (300 MHz, $CDCl_3$)

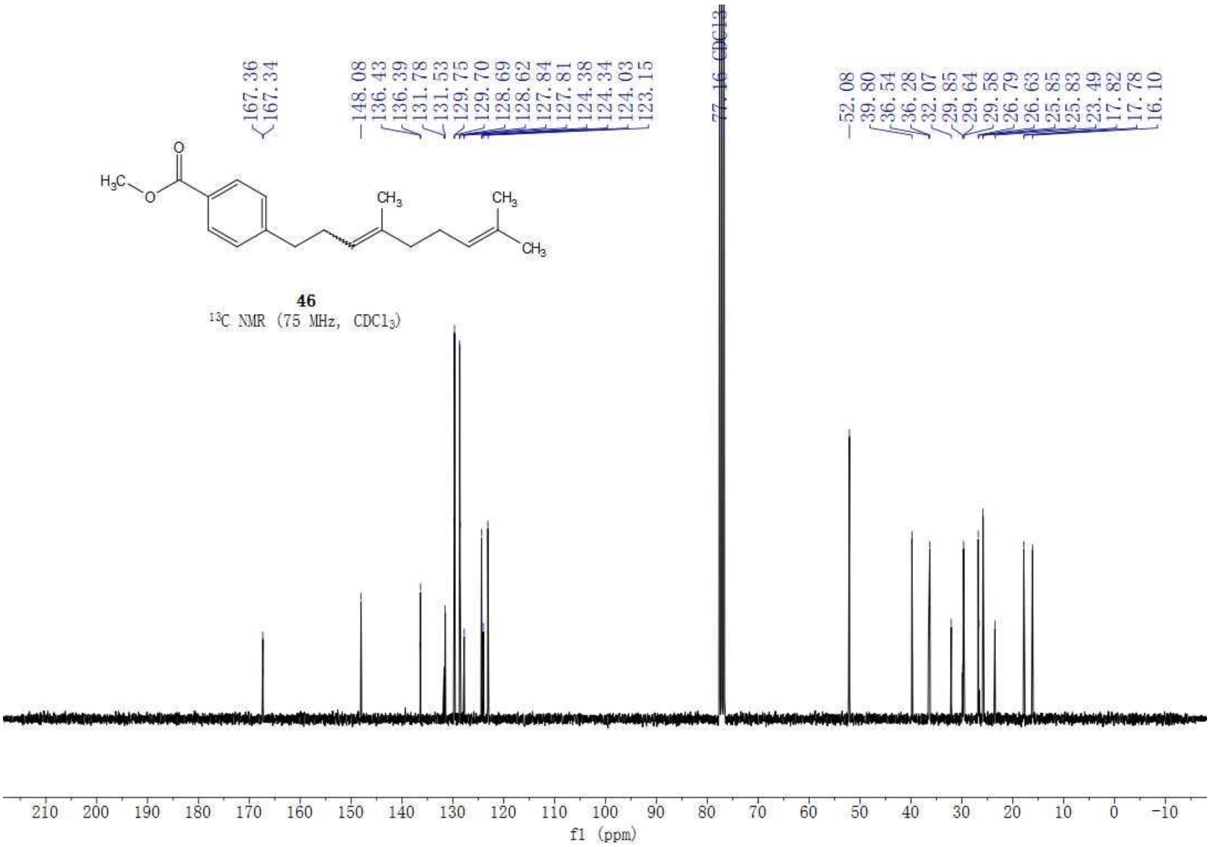

46

^{13}C NMR (75 MHz, $CDCl_3$)

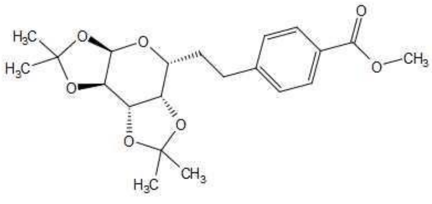

47
^{1}H NMR (300 MHz, $CDCl_3$)

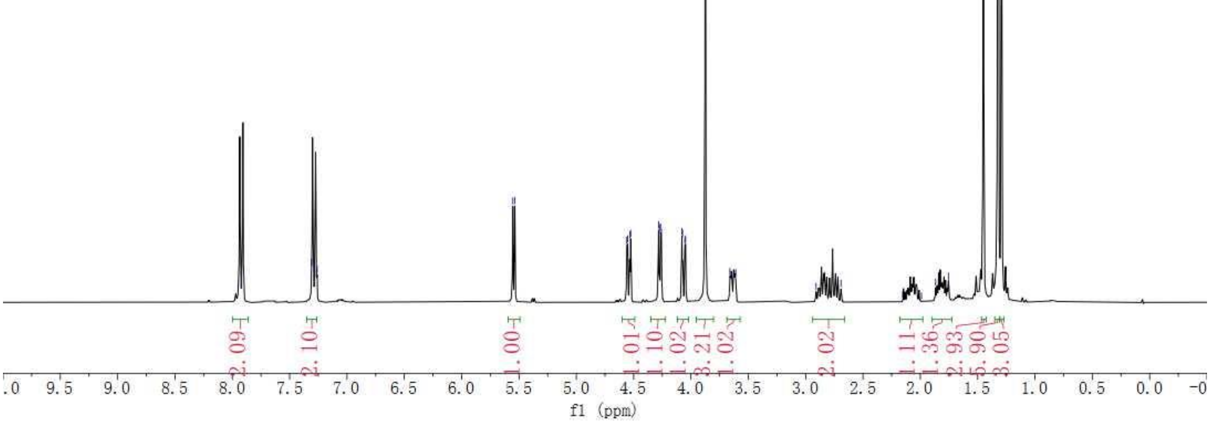

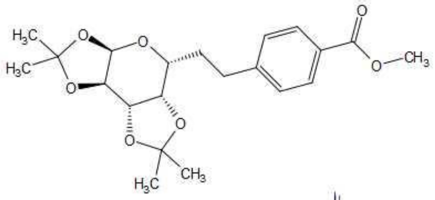

47
^{13}C NMR (75 MHz, $CDCl_3$)

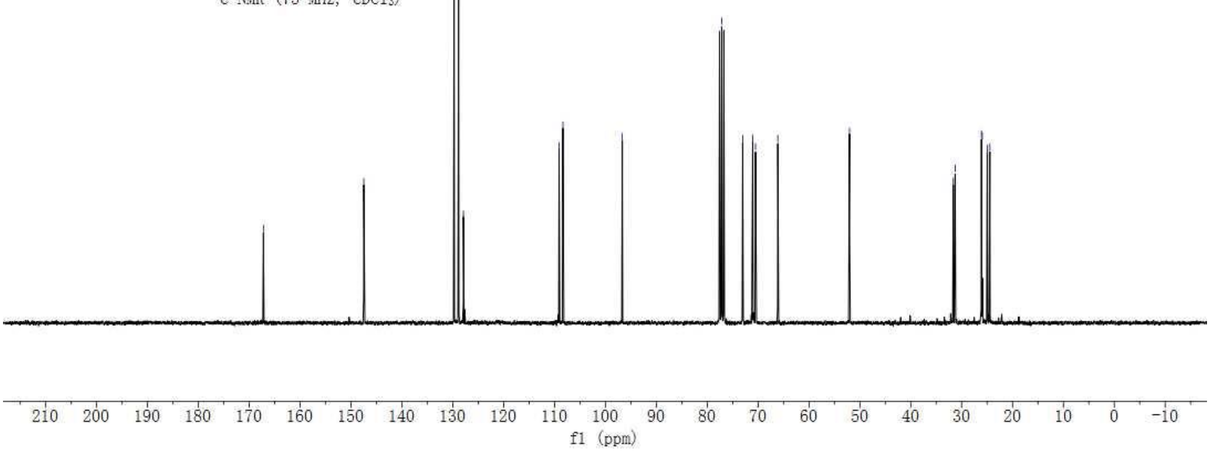

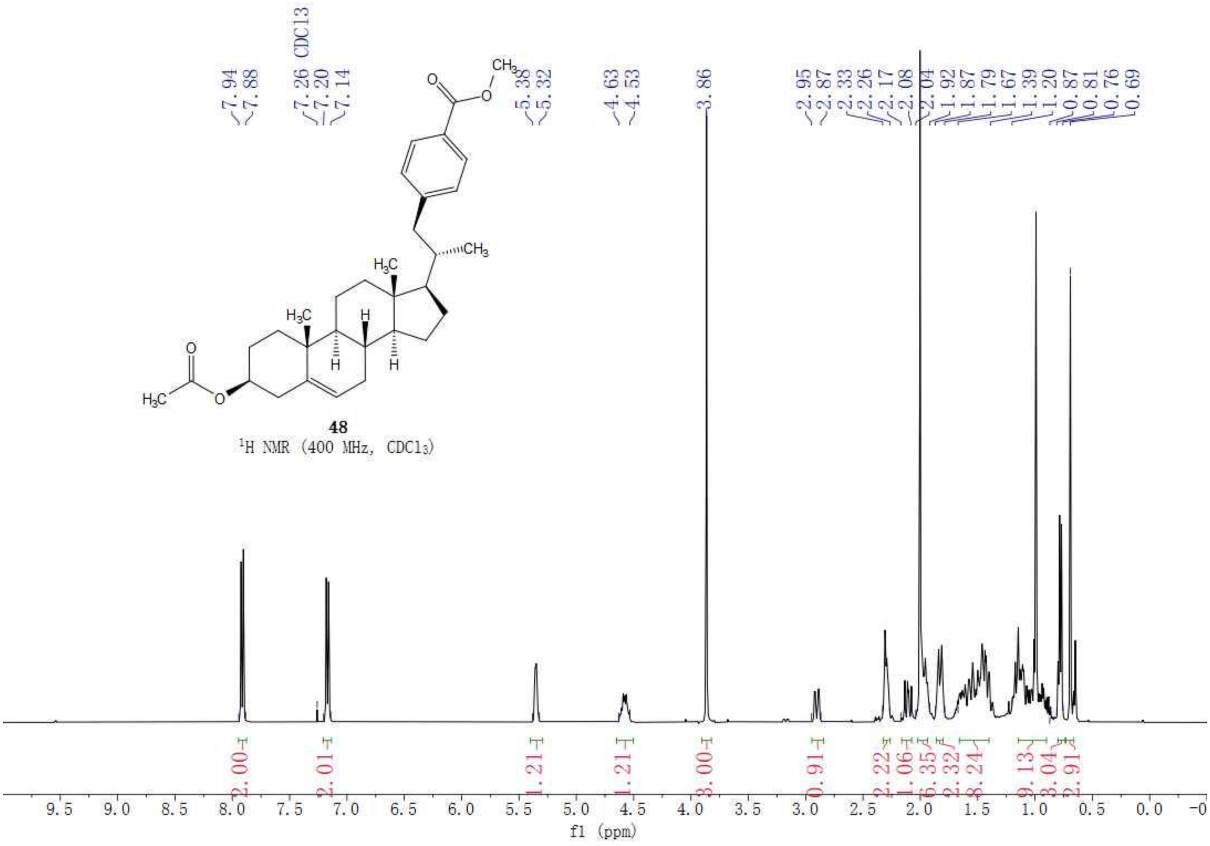
48
^{1}H NMR (400 MHz, CDCl$_3$)
7.94 7.88 7.26 CDCl3 7.20 7.14 5.38 5.32 4.63 4.53 3.86 2.95 2.87 2.33 2.26 2.17 2.08 2.04 1.92 1.87 1.79 1.67 1.39 1.20 0.87 0.81 0.76 0.69
2.00 2.01 1.21 1.21 3.00 0.91 2.22 1.06 6.35 2.32 8.24 9.13 3.04 2.91
f1 (ppm)

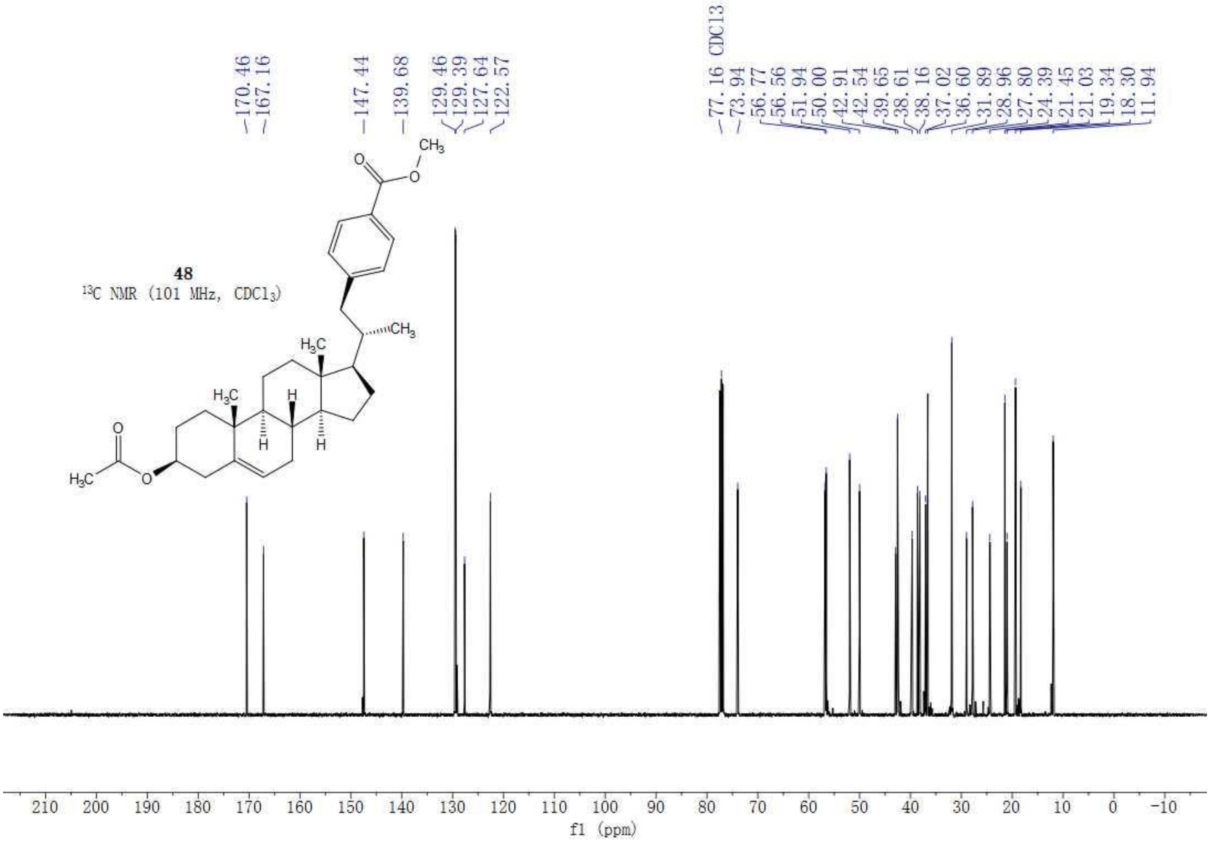
48
^{13}C NMR (101 MHz, CDCl$_3$)
170.46 167.16 147.44 139.68 129.46 129.39 127.64 122.57 77.16 CDCl3 73.94 56.77 56.56 51.94 50.00 42.91 42.54 39.65 38.61 38.16 37.02 36.60 31.89 28.96 27.80 24.39 21.45 21.03 19.34 18.30 11.94
f1 (ppm)

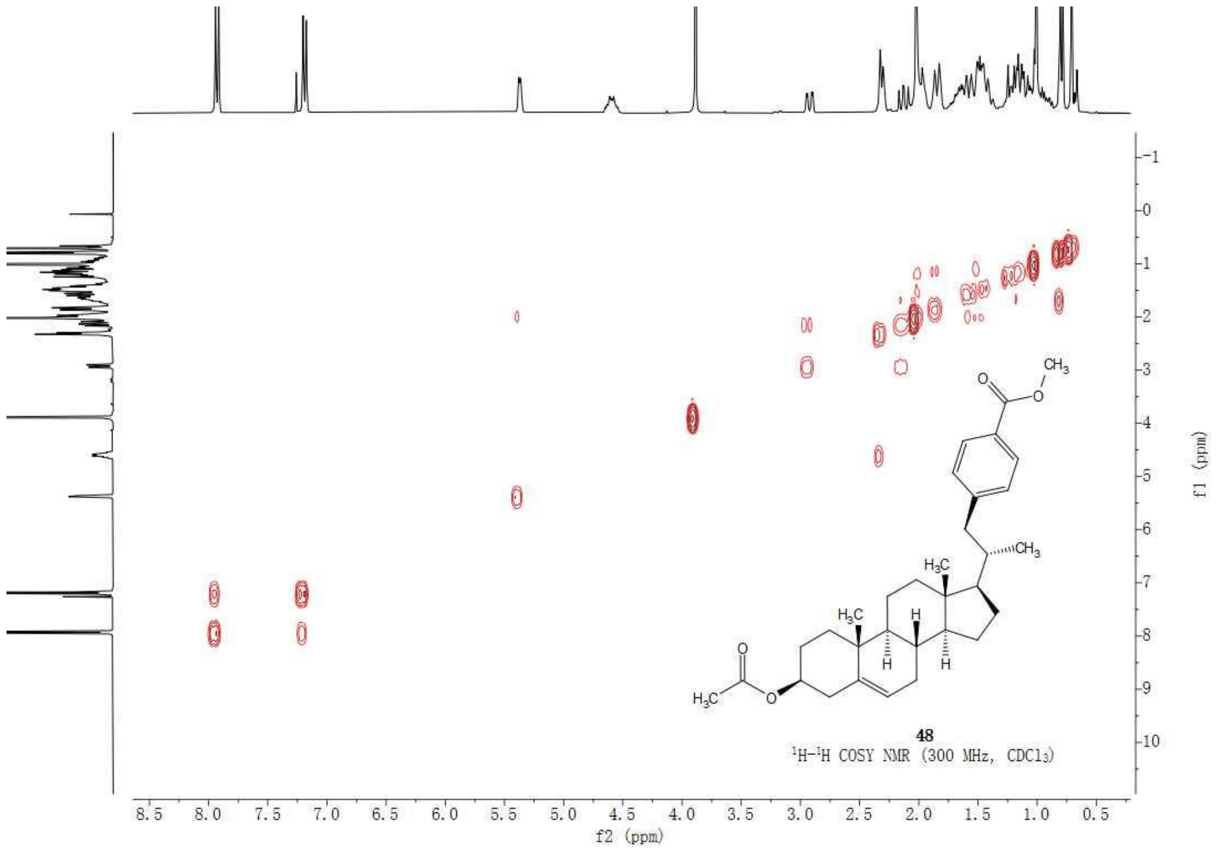
48
1H-1H COSY NMR (300 MHz, CDCl3)
f2 (ppm)
f1 (ppm)

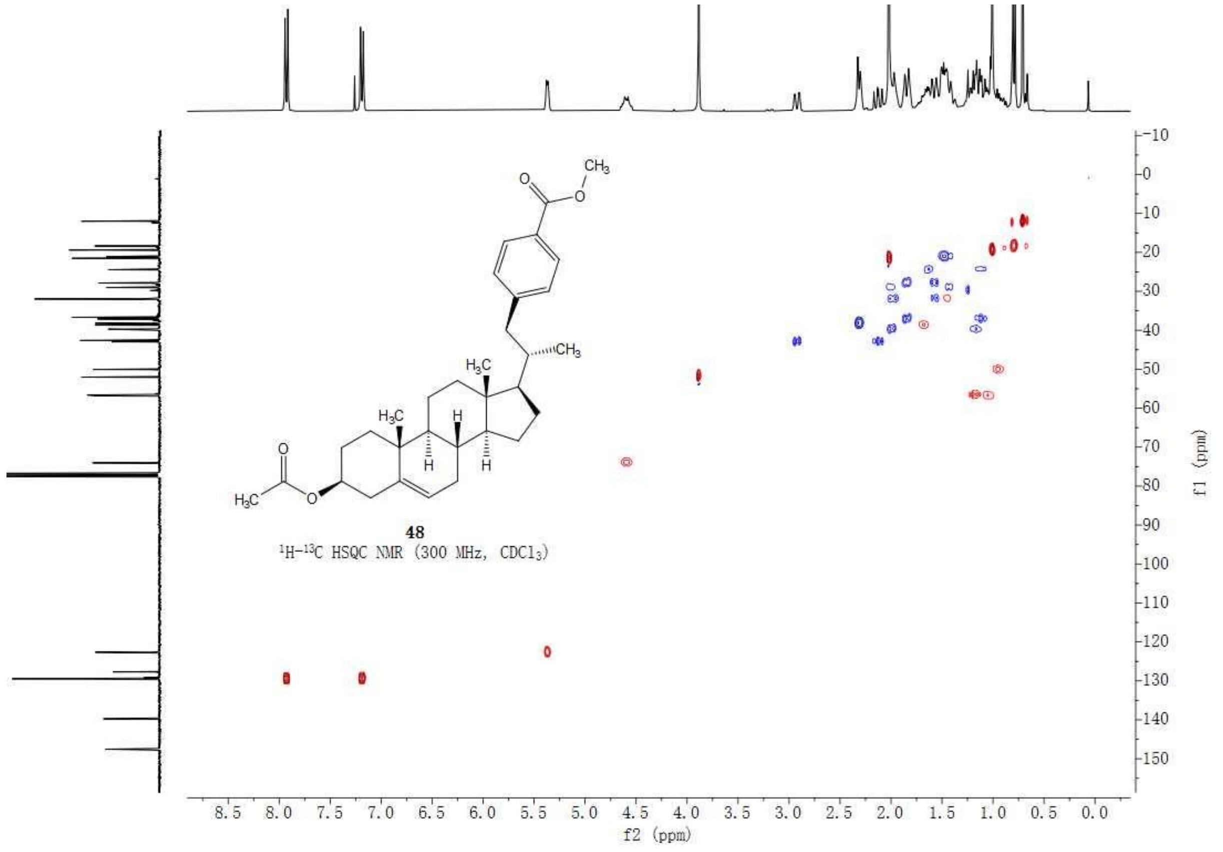
48
1H-13C HSQC NMR (300 MHz, CDCl3)
f2 (ppm)
f1 (ppm)

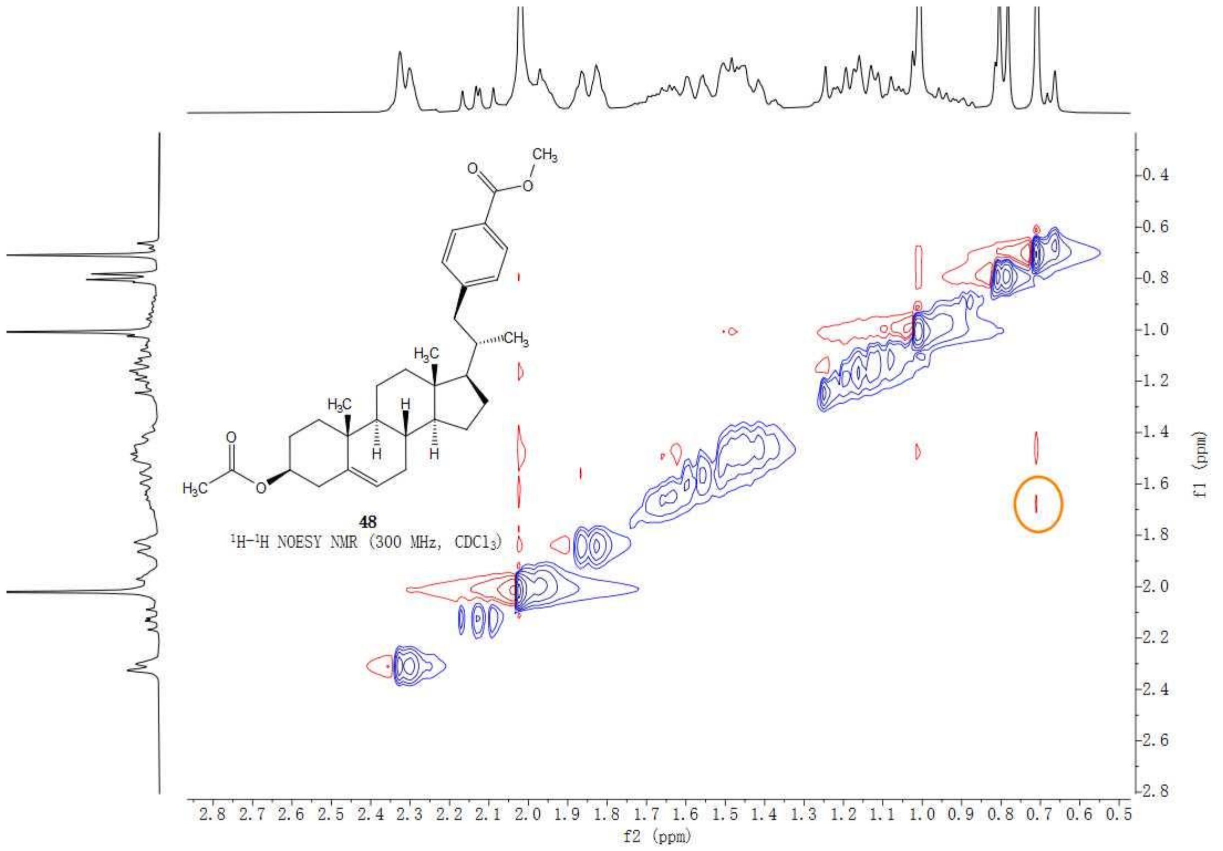

CH3
H3C
CH3
H3C
H
H
H
O
O
O
O
H3C
48
1H-1H NOESY NMR (300 MHz, CDCl3)
f2 (ppm)
f1 (ppm)

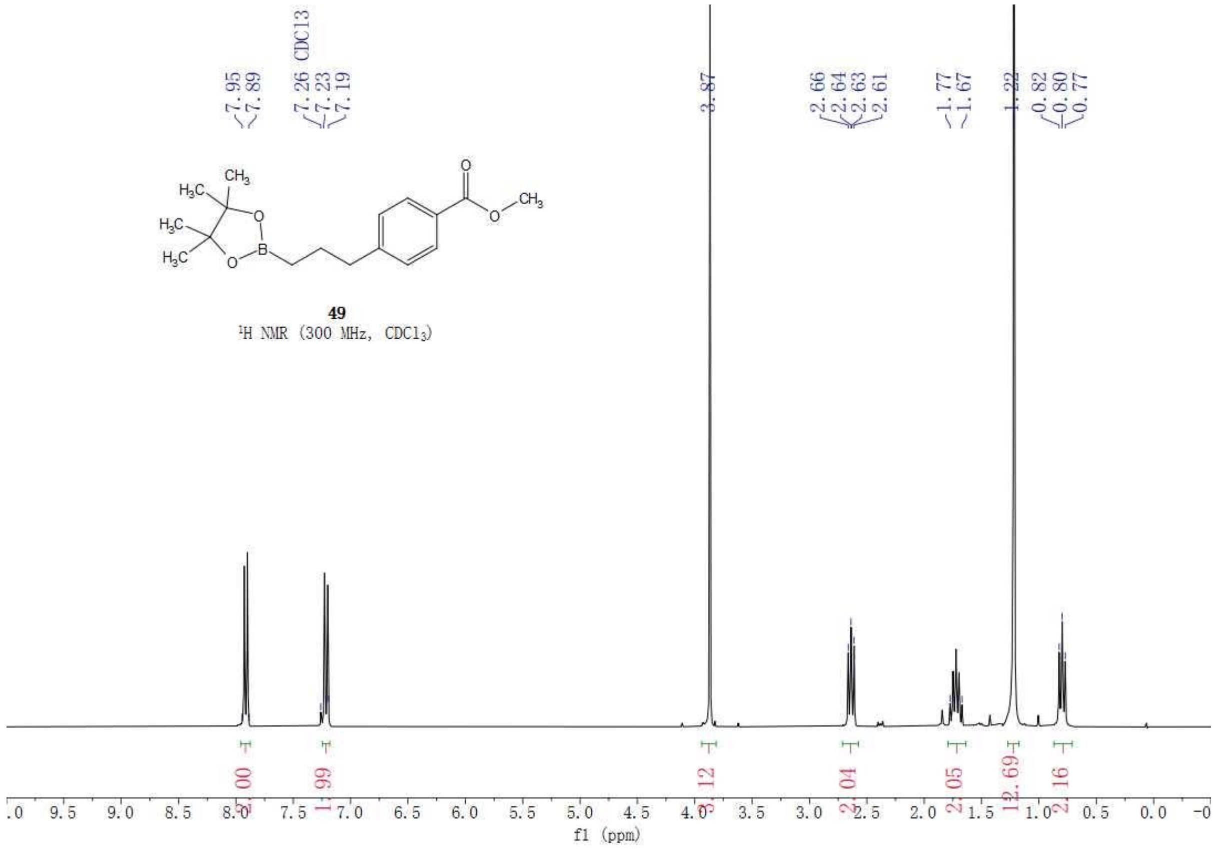

7.95
7.89
7.26 CDCl3
7.23
7.19
3.87
2.66
2.64
2.63
2.61
1.77
1.67
1.22
0.82
0.80
0.77
H3C
CH3
H3C
H3C
O
B
O
O
O
CH3
49
1H NMR (300 MHz, CDCl3)
2.00
1.99
3.12
2.04
2.05
12.69
2.16
f1 (ppm)

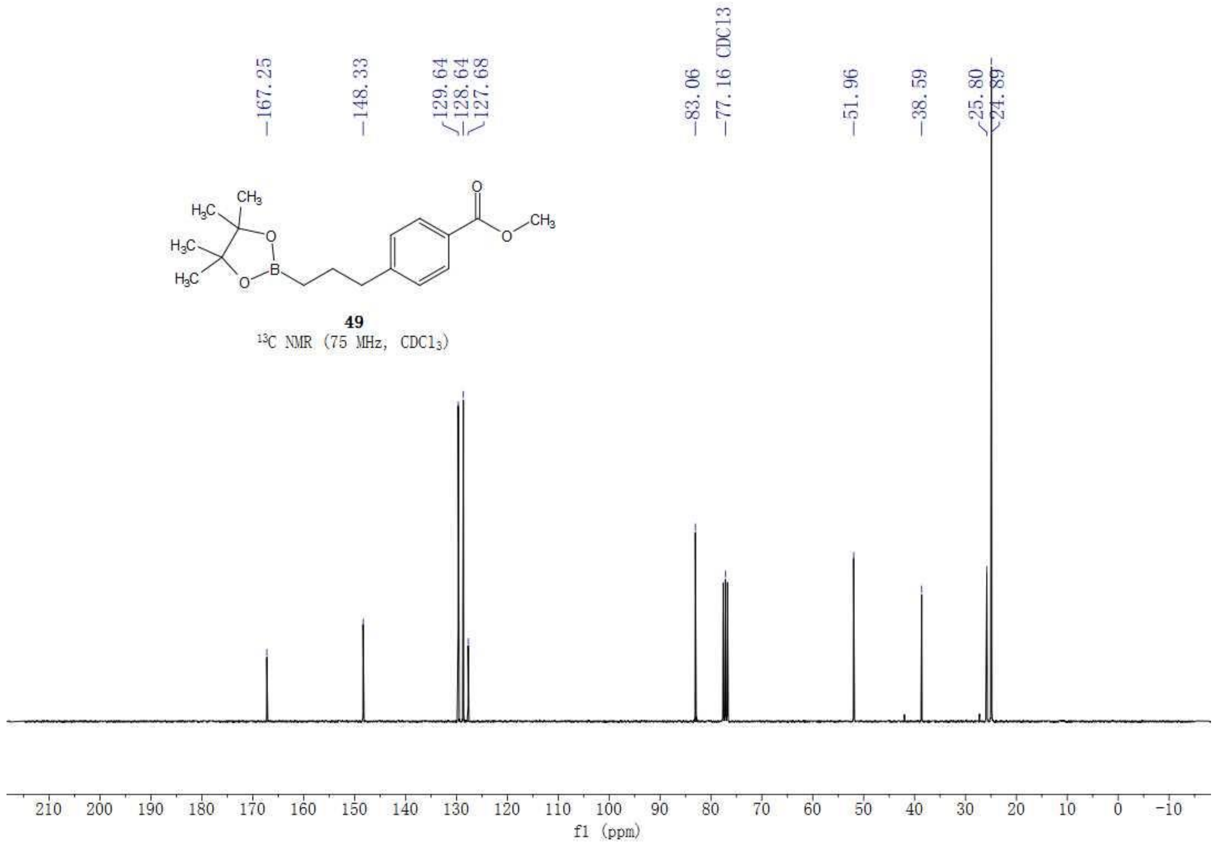

49

^{13}C NMR (75 MHz, $CDCl_3$)

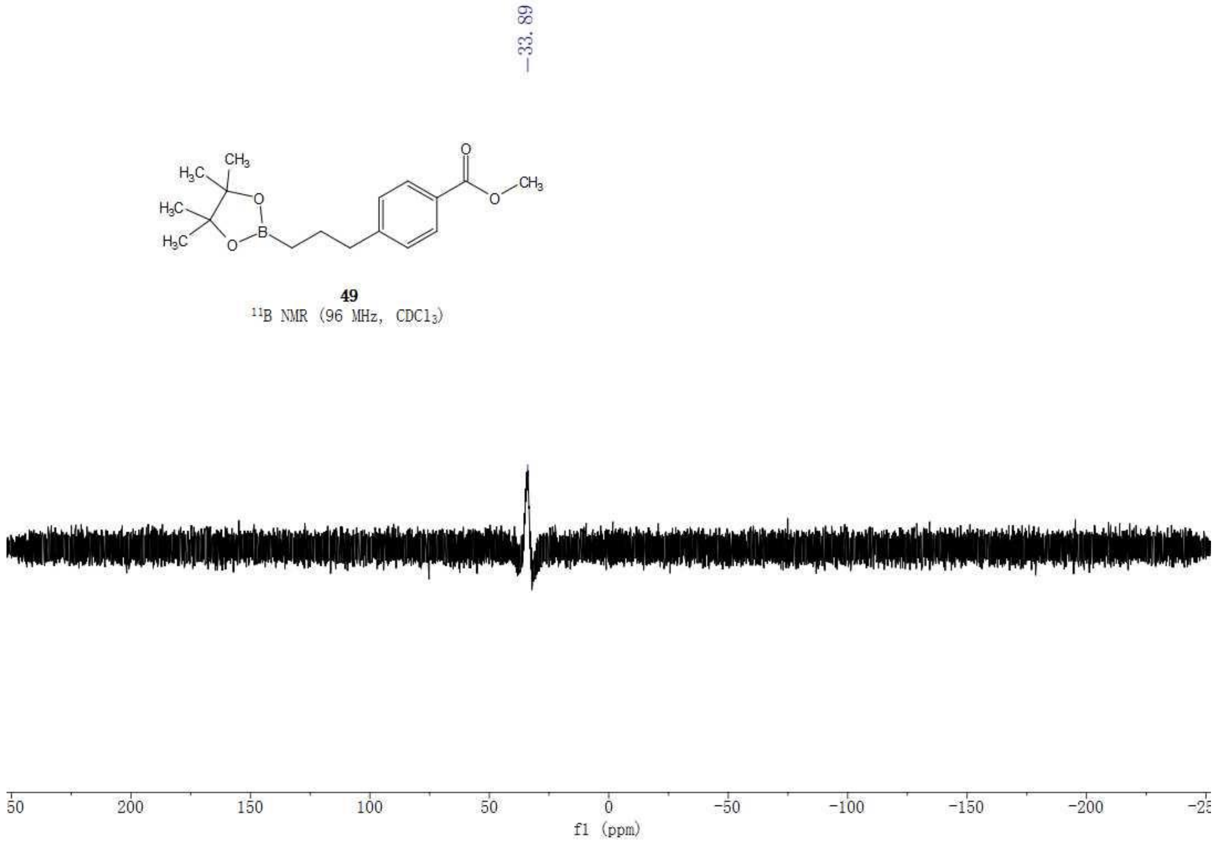

49

^{11}B NMR (96 MHz, $CDCl_3$)

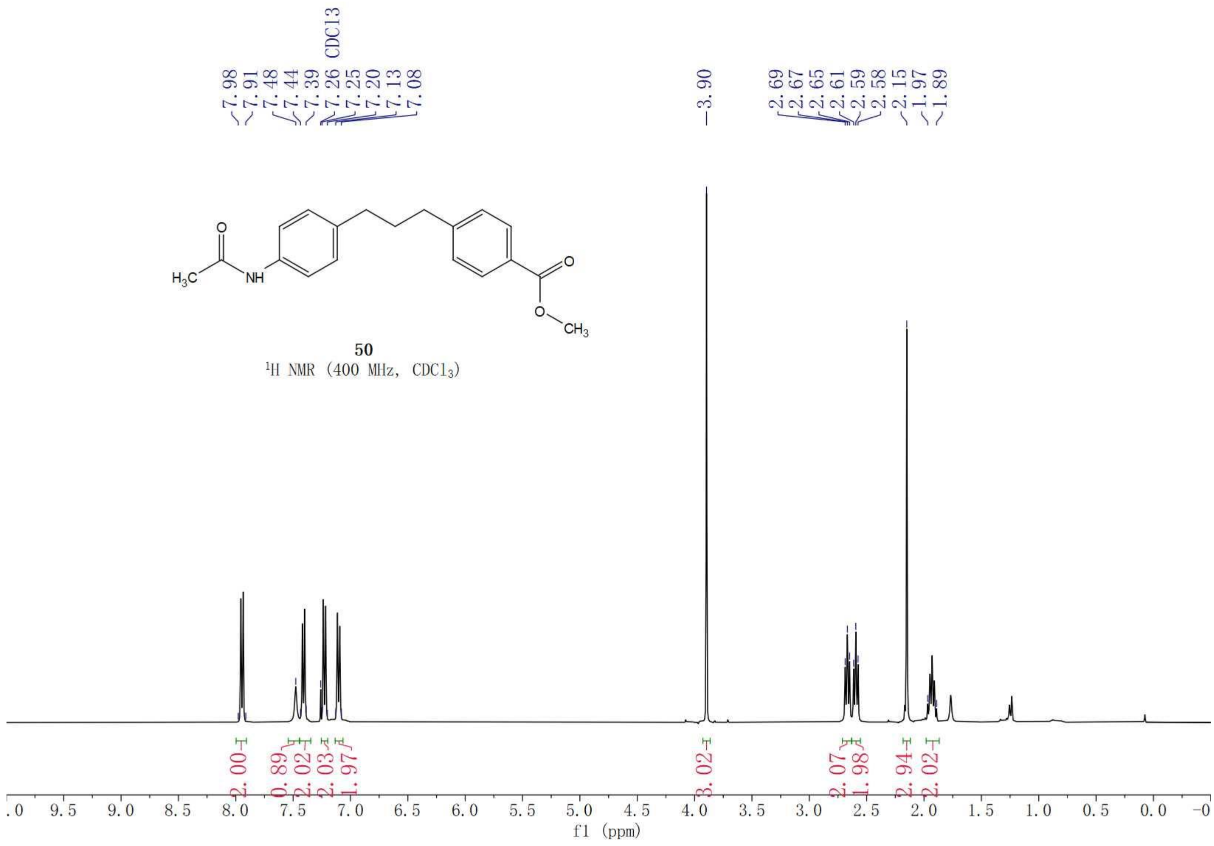
7.98
7.91
7.48
7.44
7.39
7.26 CDCl3
7.25
7.20
7.13
7.08
3.90
2.69
2.67
2.65
2.61
2.59
2.58
2.15
1.97
1.89
H3C
O
NH
O
O
CH3
50
1H NMR (400 MHz, CDCl3)
2.00
0.89
2.02
2.03
1.97
3.02
2.07
1.98
2.94
2.02
f1 (ppm)

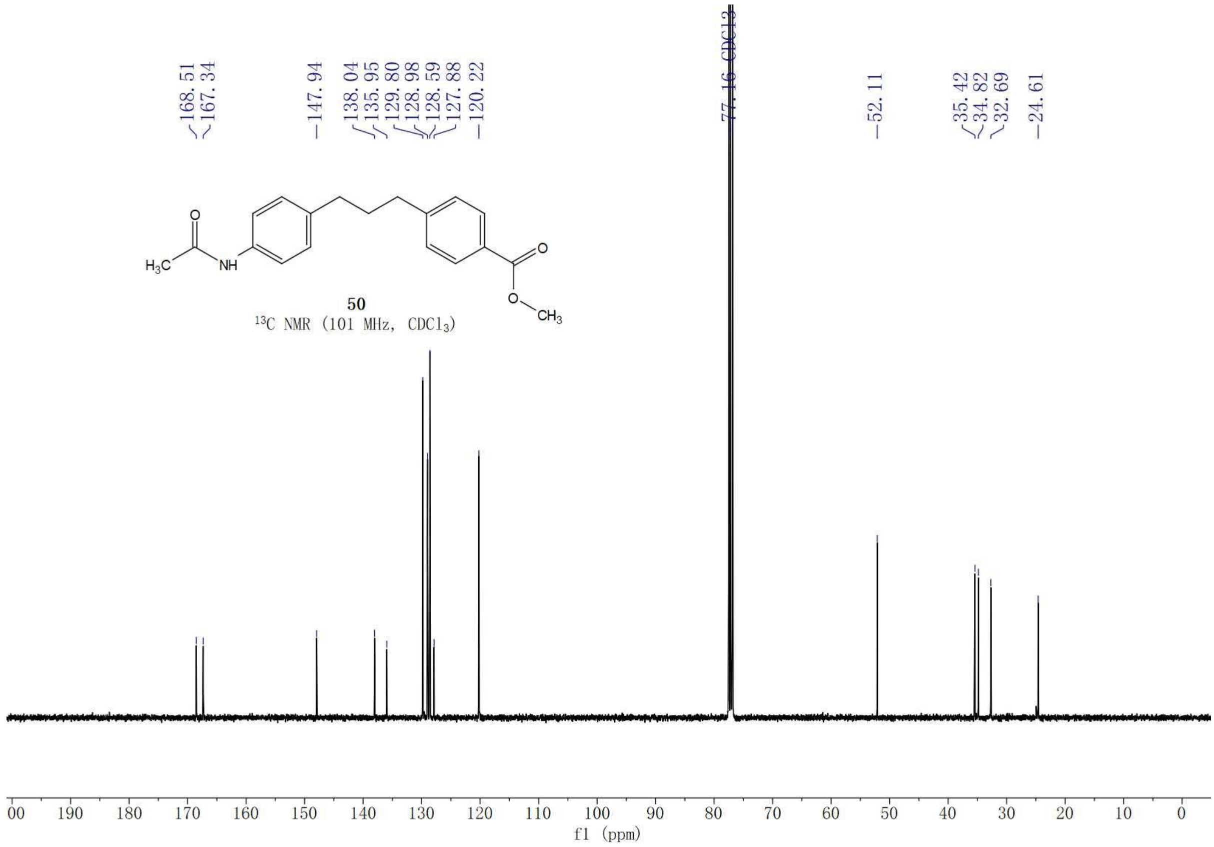
168.51
167.34
147.94
138.04
135.95
129.80
128.98
128.59
127.88
120.22
77.16 CDCl3
52.11
35.42
34.82
32.69
24.61
H3C
O
NH
O
O
CH3
50
13C NMR (101 MHz, CDCl3)
f1 (ppm)

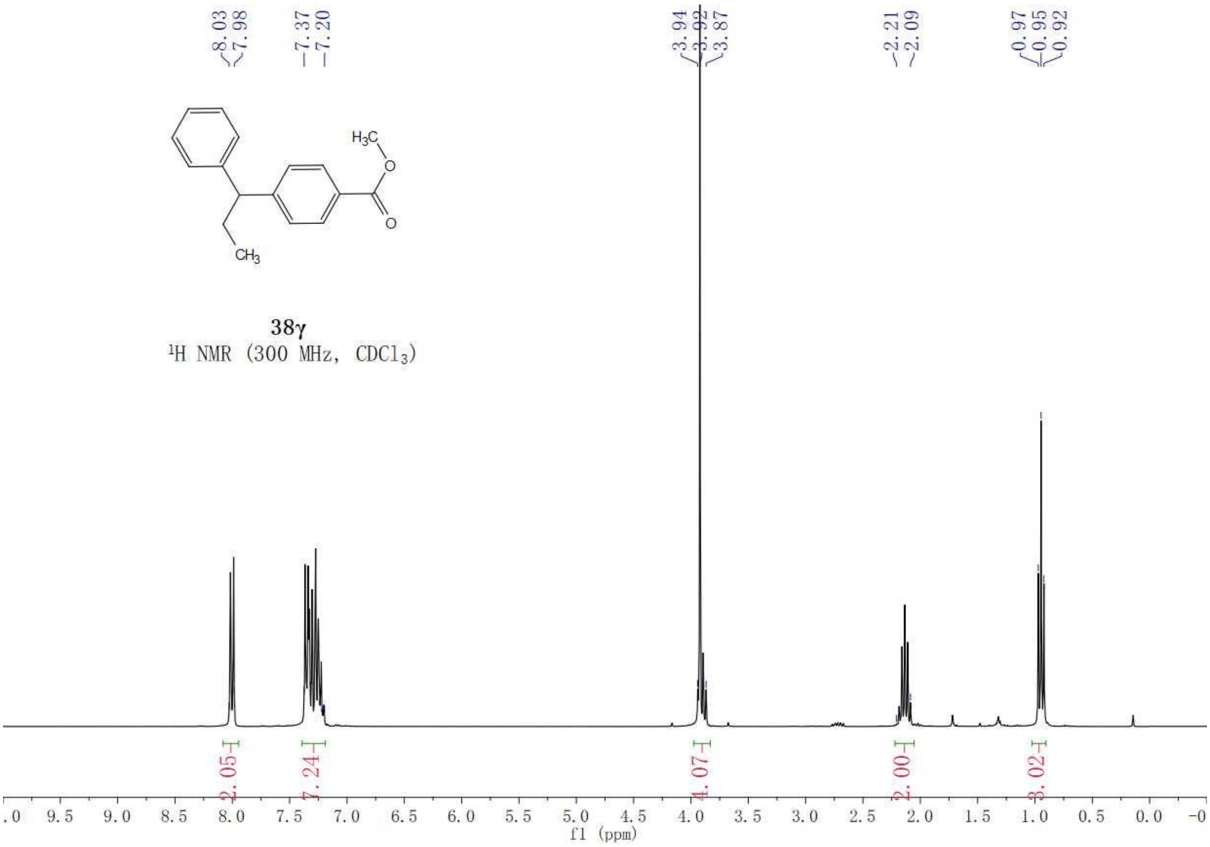
8.03
7.98
7.37
7.20
3.94
3.92
3.87
2.21
2.09
0.97
0.95
0.92
H_3C
O
O
CH_3
38γ
^1H NMR (300 MHz, $CDCl_3$)
2.05
7.24
4.07
2.00
3.02
.0 9.5 9.0 8.5 8.0 7.5 7.0 6.5 6.0 5.5 5.0 4.5 4.0 3.5 3.0 2.5 2.0 1.5 1.0 0.5 0.0 -0
f1 (ppm)

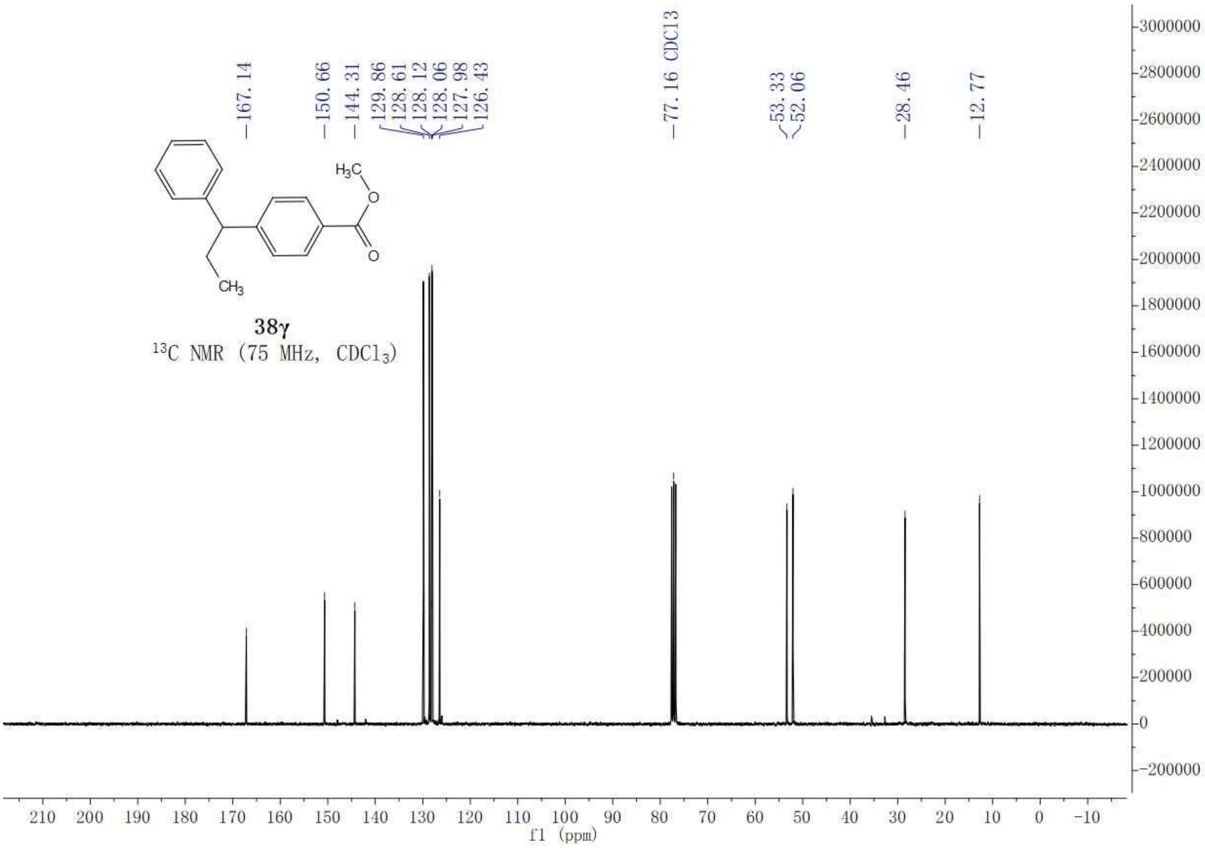
167.14
150.66
144.31
129.86
128.61
128.12
128.06
127.98
126.43
77.16 CDCl3
53.33
52.06
28.46
12.77
H_3C
O
O
CH_3
38γ
^{13}C NMR (75 MHz, $CDCl_3$)
3000000
2800000
2600000
2400000
2200000
2000000
1800000
1600000
1400000
1200000
1000000
800000
600000
400000
200000
0
-200000
210 200 190 180 170 160 150 140 130 120 110 100 90 80 70 60 50 40 30 20 10 0 -10
f1 (ppm)

3. Supplementary References

1 Norden, S., Bender, M., Rullkötter, J. & Christoffers, J. Androstanes with Modified Carbon Skeletons. *Eur. J. Org. Chem.* **2011**, 4543-4550, doi:https://doi.org/10.1002/ejoc.201100390 (2011).

2 Zhou, L. *et al.* Direct Nitration of Vinylcycles with Copper Nitrate. *Org. Lett.* **25**, 1415-1419, doi:10.1021/acs.orglett.3c00138 (2023).

3 Zhou, X., Xu, Y. & Dong, G. Olefination via Cu-Mediated Dehydroacylation of Unstrained Ketones. *J. Am. Chem. Soc.* **143**, 20042-20048, doi:10.1021/jacs.1c09587 (2021).

4 Zhang, P., Hevey, R. & Ling, C.-C. Total Synthesis of β-d-ido-Heptopyranosides Related to Capsular Polysaccharides of Campylobacter jejuni HS:4. *J. Org. Chem.* **82**, 9662-9674, doi:10.1021/acs.joc.7b01752 (2017).

5 Bo, Z. & Schlüter, A. D. Synthesis of Low-Generation, Aryl-/Alkyl-Type, Nonpolar Dendrons Carrying Protected Hydroxyalkyl Groups in the Periphery. *J. Org. Chem.* **67**, 5327-5332, doi:10.1021/jo025742k (2002).

6 Zhao, H.-Y., Zhou, M. & Zhang, X. Palladium-Catalyzed Carbonylative Cross-Coupling of Difluoroalkyl Halides with Alkylboranes under 1 atm of CO. *Org. Lett.* **23**, 9106-9111, doi:10.1021/acs.orglett.1c03396 (2021).

7 Hansch, C., Leo, A. & Taft, R. W. A survey of Hammett substituent constants and resonance and field parameters. *Chem. Rev.* **91**, 165-195, doi:10.1021/cr00002a004 (1991).

8 Chemler, S. R., Trauner, D. & Danishefsky, S. J. The B-Alkyl Suzuki–Miyaura Cross-Coupling Reaction: Development, Mechanistic Study, and Applications in Natural Product Synthesis. *Angew. Chem. Int. Ed.* **40**, 4544-4568, doi:https://doi.org/10.1002/1521-3773(20011217)40:24<4544::AID-ANIE4544>3.0.CO;2-N (2001).

9 Cismesia, M. A. & Yoon, T. P. Characterizing chain processes in visible light photoredox catalysis. *Chem. Sci.* **6**, 5426-5434, doi:10.1039/C5SC02185E (2015).

10 *Proc. R, Soc. London, Ser. A. Math. Phys. Sci.* **235**, 518-536 (1956).

11 Xu, J. *et al.* Unveiling Extreme Photoreduction Potentials of Donor–Acceptor Cyanoarenes to Access Aryl Radicals from Aryl Chlorides. *J. Am. Chem. Soc.* **143**, 13266-13273, doi:10.1021/jacs.1c05994 (2021).

12 Stoll, S. & Schweiger, A. EasySpin, a comprehensive software package for spectral simulation and analysis in EPR. *J. Magn. Reson.* **178**, 42-55, doi:https://doi.org/10.1016/j.jmr.2005.08.013 (2006).

13 Diccianni, J. B., Hu, C. & Diao, T. Binuclear, High-Valent Nickel Complexes: Ni− Ni Bonds in Aryl–Halogen Bond Formation. *Angew. Chem.* **129**, 3689-3693 (2017).

14 Zheng, B. *et al.* Organometallic Nickel(III) Complexes Relevant to Cross-Coupling and Carbon–Heteroatom Bond Formation Reactions. *J. Am. Chem. Soc.* **136**, 6499-6504, doi:10.1021/ja5024749 (2014).

15 Griego, L., Chae, J. B. & Mirica, L., doi:10.26434/chemrxiv-2023-fpf1n (2023).

16 Lee, H., Börgel, J. & Ritter, T. Carbon–Fluorine Reductive Elimination from Nickel(III) Complexes. *Angew. Chem. Int. Ed.* **56**, 6966-6969, doi:https://doi.org/10.1002/anie.201701552 (2017).

17 Guo, L., Song, F., Zhu, S., Li, H. & Chu, L. syn-Selective alkylarylation of terminal alkynes via the combination of photoredox and nickel catalysis. *Nat. Commun.* **9**, 4543, doi:10.1038/s41467-018-06904-9 (2018).

18 Donabauer, K. *et al.* Photocatalytic carbanion generation - benzylation of aliphatic aldehydes to secondary alcohols. *Chem Sci* **10**, 5162-5166, doi:10.1039/c9sc01356c (2019).

19 Lane, C. F. The reaction between trialkylboranes and copper(II) salts. An unusual electron-transfer oxidation-reduction. *J. Organometal. Chem.* **31**, 421-431, doi:https://doi.org/10.1016/S0022-328X(00)86250-6 (1971).

20 Bour, J. R. *et al.* Carbon–Carbon Bond-Forming Reductive Elimination from Isolated Nickel(III) Complexes. *J. Am. Chem. Soc.* **138**, 16105-16111, doi:10.1021/jacs.6b10350 (2016).

21 Tellis, J. C., Primer, D. N. & Molander, G. A. Single-electron transmetalation in organoboron cross-coupling by photoredox/nickel dual catalysis. *Science* **345**, 433-436, doi:10.1126/science.1253647 (2014).

22 Zuo, Z. *et al.* Merging photoredox with nickel catalysis: Coupling of α-carboxyl sp³-carbons with aryl halides. *Science* **345**, 437-440, doi:doi:10.1126/science.1255525 (2014).

23 Xue, G., Xie, F., Liang, H., Chen, G. & Dai, W. Copper-Catalyzed Oxidative C–C Bond Cleavage of Alkyl-(Hetero)arenes Enabling Direct Access to Nitriles. *Org. Lett.* **24**, 5590-5595, doi:10.1021/acs.orglett.2c02238 (2022).

24 Yang, W., Gao, L., Lu, J. & Song, Z. Chemoselective deoxygenation of ether-substituted alcohols and carbonyl compounds by B(C6F5)3-catalyzed reduction with (HMe2SiCH2)2. *Chem. Commun.* **54**, 4834-4837, doi:10.1039/C8CC01163J (2018).

25 Wang, X., Cui, P., Xia, C. & Wu, L. Catalytic Boration of Alkyl Halides with Borane without Hydrodehalogenation Enabled by Titanium Catalyst. *Angew. Chem. Int. Ed.* **60**, 12298-12303, doi:https://doi.org/10.1002/anie.202100569 (2021).

26 Wu, Q. *et al.* Pd-Catalyzed Alkylation of (Iso)quinolines and Arenes: 2-Acylpyridine Compounds as Alkylation Reagents. *Org. Lett.* **20**, 6345-6348, doi:10.1021/acs.orglett.8b02498 (2018).

27 Li, H. *et al.* Visible-light-induced C(sp3)–C(sp3) bond formation via radical/radical cross-coupling. *Chem. Commun.* **59**, 1205-1208, doi:10.1039/D2CC05840E (2023).

28 Schirmer, T. E. *et al.* Mesoporous Graphitic Carbon Nitride as a Heterogeneous Organic Photocatalyst in the Dual Catalytic Arylation of Alkyl Bis(catecholato)silicates. *Org. Lett.* **24**, 2483-2487, doi:10.1021/acs.orglett.2c00529 (2022).

29 Mazzarella, D., Pulcinella, A., Bovy, L., Broersma, R. & Noël, T. Rapid and Direct Photocatalytic C(sp3)−H Acylation and Arylation in Flow. *Angew. Chem. Int. Ed.* **60**, 21277-21282, doi:https://doi.org/10.1002/anie.202108987 (2021).

30 Luridiana, A. *et al.* The Merger of Benzophenone HAT Photocatalysis and Silyl Radical-Induced XAT Enables Both Nickel-Catalyzed Cross-Electrophile Coupling and 1,2-Dicarbofunctionalization of Olefins. *ACS Catal.* **12**, 11216-11225, doi:10.1021/acscatal.2c03805 (2022).

31 Wu, Y., Chen, Y.-Q., Liu, T., Eastgate, M. D. & Yu, J.-Q. Pd-Catalyzed γ-C(sp3)–H Arylation of Free Amines Using a Transient Directing Group. *J. Am. Chem. Soc.* **138**, 14554-14557, doi:10.1021/jacs.6b09653 (2016).

32 Zhang, X. & MacMillan, D. W. C. Alcohols as Latent Coupling Fragments for Metallaphotoredox Catalysis: sp3–sp2 Cross-Coupling of Oxalates with Aryl Halides. *J. Am. Chem. Soc.* **138**, 13862-13865, doi:10.1021/jacs.6b09533 (2016).

33 Speckmeier, E. & Maier, T. C. ART—An Amino Radical Transfer Strategy for C(sp2)–C(sp3) Coupling Reactions, Enabled by Dual Photo/Nickel Catalysis. *J. Am. Chem. Soc.* **144**, 9997-10005, doi:10.1021/jacs.2c03220 (2022).

34 Liu, J., Gong, H. & Zhu, S. BH3 · Me2S: An Alternative Hydride Source for NiH-Catalyzed Reductive Migratory Hydroarylation and Hydroalkenylation of Alkenes. *Eur. J. Org. Chem.* **2021**, 1543-1546, doi:https://doi.org/10.1002/ejoc.202100005 (2021).
